# Reduced replication origin licensing selectively kills KRAS-mutant colorectal cancer cells via mitotic catastrophe

**DOI:** 10.1038/s41419-020-2704-9

**Published:** 2020-07-01

**Authors:** Bastian Gastl, Kathleen Klotz-Noack, Bertram Klinger, Sylvia Ispasanie, Krenoula Hani Fouad Salib, Johannes Zuber, Soulafa Mamlouk, Natalie Bublitz, Nils Blüthgen, David Horst, Markus Morkel, Reinhold Schäfer, Christine Sers

**Affiliations:** 1 Institute of Pathology, Charité Universitätsmedizin Berlin, Charitéplatz 1, 10117 Berlin, Germany; 2 BSIO—Berlin School of Integrative Oncology, 13353 Berlin, Germany; 3 German Cancer Consortium (DKTK), German Cancer Research Center, Partner Site Berlin, 69120 Heidelberg, Germany; 4 IRI Life Sciences and Institute for Theoretical Biology, Humboldt University Berlin, Philippstr. 13/Haus 18, 10115 Berlin, Germany; 5 Research Institute of Molecular Pathology, Vienna Biocenter, 1030 Vienna, Austria

**Keywords:** Oncogenes, Origin firing

## Abstract

To unravel vulnerabilities of KRAS-mutant CRC cells, a shRNA-based screen specifically inhibiting MAPK pathway components and targets was performed in CaCo2 cells harboring conditional oncogenic KRAS^G12V^. The custom-designed shRNA library comprised 121 selected genes, which were previously identified to be strongly regulated in response to MEK inhibition. The screen showed that CaCo2 cells expressing KRAS^G12V^ were sensitive to the suppression of the DNA replication licensing factor minichromosome maintenance complex component 7 (MCM7), whereas KRAS^wt^ CaCo2 cells were largely resistant to MCM7 suppression. Similar results were obtained in an isogenic DLD-1 cell culture model. Knockdown of MCM7 in a KRAS-mutant background led to replication stress as indicated by increased nuclear RPA focalization. Further investigation showed a significant increase in mitotic cells after simultaneous MCM7 knockdown and KRAS^G12V^ expression. The increased percentage of mitotic cells coincided with strongly increased DNA damage in mitosis. Taken together, the accumulation of DNA damage in mitotic cells is due to replication stress that remained unresolved, which results in mitotic catastrophe and cell death. In summary, the data show a vulnerability of KRAS-mutant cells towards suppression of MCM7 and suggest that inhibiting DNA replication licensing might be a viable strategy to target KRAS-mutant cancers.

## Introduction

*RAS* genes constitute the most commonly mutated oncogenes in human malignancies and serve as drivers of cellular transformation and tumor maintenance^1^. Even though *RAS* genes were the first oncogenes to be discovered, no targeted therapy for KRAS, NRAS, or HRAS mutant cancers has made its way to clinical application. This failure was not only due to the particularly high affinity of RAS proteins for the cofactor GTP, rendering its displacement by competing drugs inefficient, but also due to an incomplete understanding of the biochemical properties and individual functions of different RAS isoforms^2^. Only recently, selective inhibitors targeting the KRAS^G12C^ mutation, which occurs in a small subset of KRAS-mutant cancer patients, were identified and further developed^3,4^.

RAS proteins activate downstream signaling pathways via different effectors including the RAF proteins, RAL-GDS, and PIK3CA among others. The two most prominent effector pathways, the RAF–MEK–ERK and the PI3K–AKT–mTOR pathway, impinge on multiple cellular functions (reviewed in ref. ^5^). RAS proteins drive proliferation through cyclin and CDK activation^6,7^, interfere with apoptotic pathways^8^ and affect DNA replication and cell cycle checkpoint control^9,10^. Moreover, RAS proteins deregulate cellular metabolism by promoting glucose import and consumption^11,12^. The diversity of RAS-dependent regulation of cellular processes potentially offers a broad spectrum of potential intervention targets among the RAS effector pathways. Currently, pathway inhibitors acting on the RAS downstream effectors RAF and MEK are the furthermost developed therapeutic compounds^13^. Trametinib and cobimetinib, selective inhibitors against the effector kinases MEK1/2, have been clinically approved and are used in combination with selective BRAF^V600E^ inhibitors in BRAF-driven malignant melanoma^14^. In contrast, MEK or RAF inhibitors turned out to be surprisingly ineffective in RAS mutant cancer patients. This is due to paradoxical and feed-back dependent re-activation of the MEK/ERK and the PI3K/AKT axis in an EGFR-dependent manner^15^. To overcome these limitations, combinatorial inhibition of MEK/ERK and PI3K/AKT pathways was envisaged as a rational solution, however, the high level of toxicity in cancer patients enforced rapid termination of clinical trials^2^.

In recent years, functional genomic and synthetic lethality screens using shRNA and CRISPR/Cas9 technology have provided a new avenue for searching targetable structures in RAS mutant tumor cells (reviewed in ref. ^16^). Such screening efforts revealed a broad spectrum of genes required for cellular survival and transformation mediated by mutant KRAS, NRAS or HRAS genes. For example, the apoptosis inhibitor BCL-XL^17^ was among the factors identified to be essential for KRAS mutant colorectal cancer cells, as well as the DNA replication licensing factor CDC6^18^. Additionally, a critical role of the proteasome was noticed in such screens multiple times^18,19^, indicating its functional alliance with KRAS.

Here we describe a synthetic lethality screen based on a focused shRNA library targeting transcription factors, DNA binding proteins and other nuclear proteins. These factors were previously retrieved by gene expression profiling as being up-regulated via MAPK signaling in KRAS mutant colorectal cancer cells^20^ as well as in mesenchymal and epithelial cells transformed by HRAS and KRAS oncogenes, respectively^21,22^. We transduced the library into an isogenic model system based on the colorectal cancer cell line CaCo2, harboring a conditional mutant KRAS^G12V^ transgene. This approach revealed that suppression of the minichromosome maintenance complex (MCM) subunit MCM7 is synthetic lethal with mutated KRAS. The MCM complex plays a central role in DNA replication via licensing of replication origins and governance of replication speed. The essential function of MCM7 in KRAS mutant cells is discussed.

## Results

### Suppression of MCM7 is synthetic lethal with KRAS mutant colorectal cancer cells

**Fig. 1:**
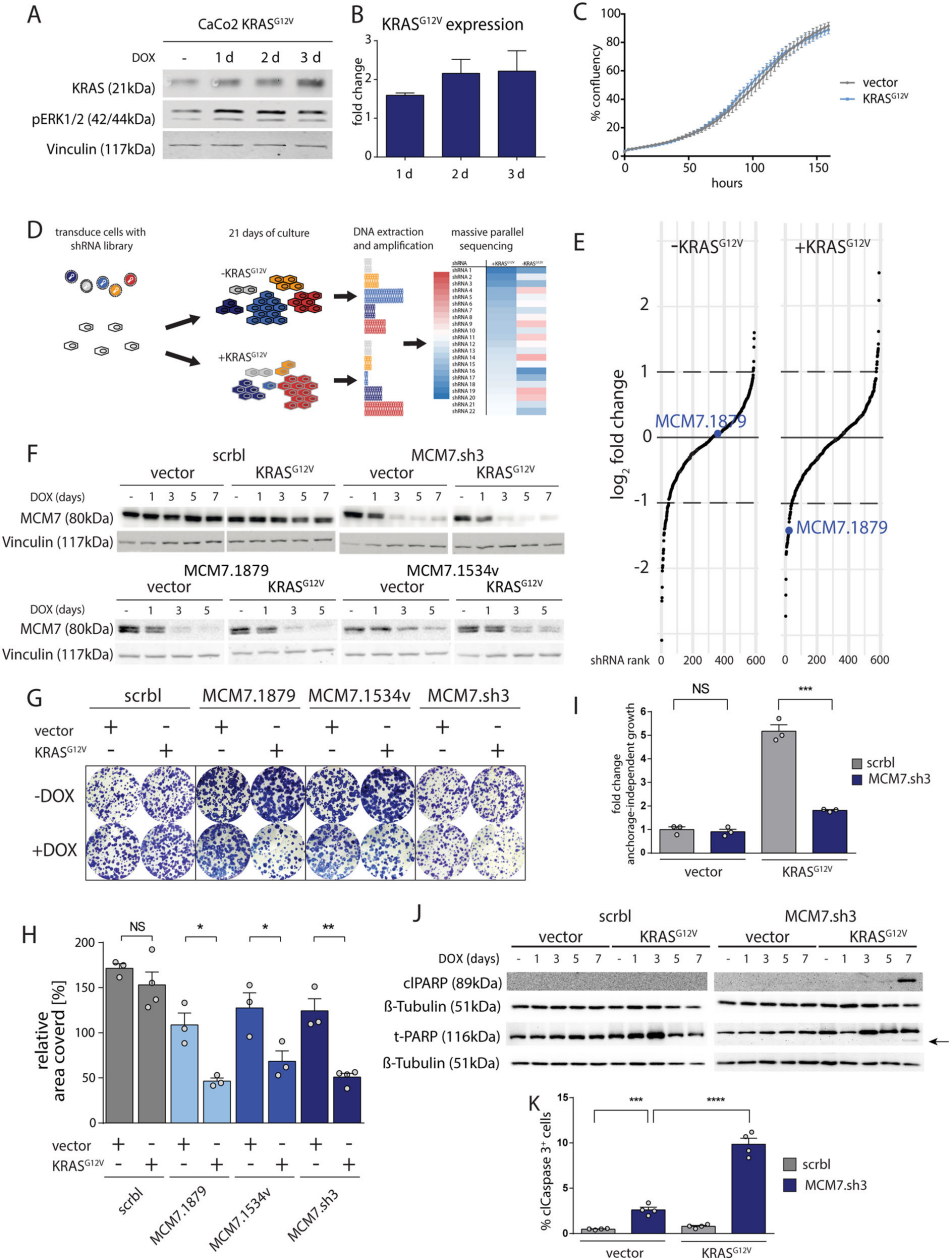
KRAS mutant cells are sensitive to MCM7 suppression. **a** Western blot analysis shows that KRAS^G12V^ overexpression is at physiological levels and potent to induce downstream activation of ERK. Vinculin serves as a loading control. **b** Quantification of KRAS^G12V^ protein expression after 1, 2, and 3 days of doxycycline induction. Mean expression ± SEM is shown (*n* = 2–3 per group). **c** Representative growth curve of CaCo2 cells after KRAS^G12V^ induction shows no difference in growth with or without KRAS^G12V^ expression (*n* = 6 technical replicates; error bars = SEM). **d** Graphical outline of the RNAi screen to identify genetic interactors of mutant KRAS. **e** Change of shRNA abundance in the shRNA screen after 21 days of culture with and without doxycycline as determined by massive parallel sequencing. Each dot represents a shRNA. The *y*-axis depicts the log_2_ fold change of shRNA abundance. The *x*-axis shows each shRNA ranked by their fold change. The shRNA MCM7.1879 is specifically depleted when co-expressed with doxycycline induced KRAS^G12V^. **f** Western blots showing MCM7 expression after knockdown. KRAS^G12V^ and shRNA expression were induced with doxycycline for 0, 1, 3, 5, or 7 days. MCM7 knockdown was efficient after 3 days of shRNA expression. Vinculin serves as loading control. **g**, **h** Clonogenic assays of three non-overlapping shRNAs targeting MCM7 show increased sensitivity of KRAS^G12V^ expressing CaCo2 cells to MCM7 knockdown. Mean percentage of the relative area covered by cells ± SEM is shown (*n* = 3–4 per group). Student’s *t*-test (two-sided); NS = not significant; *p < 0.05; **p < 0.01. **i** Soft agar assays showing anchorage-independent growth in CaCo2 cells ± induced KRAS^G12V^ and ± MCM7.sh3. KRAS^G12V^ expression increases colony formation in CaCo2 cells about five fold. MCM7 knockdown has no detectable effect on cells expressing an empty control vector, whereas cells expressing additionally KRAS^G12V^ reduce colony formation significantly. Mean fold change of colonies formed after five weeks of culture is shown ± SEM (*n* = 3 per group). Student’s *t*-test (two-sided); NS = not significant; ***p < 0.001. **j** Western blots show an increase in cleaved PARP (clPARP) staining after five and seven days of MCM7 knockdown specifically in CaCo2 cells expressing KRAS^G12V^. ß-Tubulin and total PARP serve as a loading control, the cleaved PARP is indicated by an arrow. **k** FACS analysis of cleaved Caspase 3 (clCaspase 3) reveals a significant increase of apoptosis in cells co-expressing KRAS^G12V^ and MCM7.sh3 compared to cells expressing MCM7.sh3 alone. Mean percentage of clCaspase 3^+^ cells ± SEM is shown (*n* = 4 per group). Student’s *t*-test (two-sided); ***p < 0.001; ****p < 0.0001.

For transduction of the shRNA library, we established an isogenic colorectal cell culture model by introducing conditional KRAS^G12V^ into the CaCo2 cell line that forms moderately well-differentiated adenocarcinomas in nude mice and exhibits the capacity to differentiate into enterocytes in vitro^23^. Doxycycline induced expression of KRAS^G12V^ in Caco2 cultures revealed elevated RAS levels about two-fold higher than endogenous KRAS levels. This near physiological expression level was sufficient to potently activate downstream MAPK signaling (Fig. 1a, b). While there were no differences in growth between non-induced Caco2 and KRAS^G12V^ expressing Caco2 cells in monolayer culture, the oncogene-transformed cells exhibited increased anchorage-independence (Figs. 1c, S1A). We transduced the cells by retroviral transfer with a custom microRNA-based shRNA library comprising 625 shRNAs targeting previously identified MAPK pathway-regulated target genes (Supplementary Table 1). Three days after transduction, a control sample was taken and the remaining cells were split into two samples, which were separately cultured for 21 more days with or without doxycycline induction, respectively. Cells were then harvested, genomic DNA isolated, and the shRNA cassettes amplified via PCR. The relative abundance of each shRNA in both populations compared to the control sample was determined by massive parallel sequencing (Fig. 1d).

We identified the shRNA MCM7.1879 to be specifically suppressed in the cell population expressing KRAS^G12V^, whereas cells without mutant KRAS did not show a reduction of MCM7.1879 abundance (Fig. 1e). To verify this finding, we transfected CaCo2 cells harboring either inducible KRAS^G12V^ or a control vector with four different doxycycline-inducible shRNAs. We tested the shRNA from the screen (MCM7.1879), two additional independent shRNAs directed against MCM7 (MCM7.1543v, MCM7.sh3), and a non-targeting control shRNA (scrbl) on their knockdown efficiencies and in colony formation assays. MCM7.1879 and MCM7.sh3 lead to an efficient knockdown of MCM7 in cells expressing KRAS^G12V^ or an empty vector, whereas MCM7.1534v was less effective (Fig. 1f). Knockdown of MCM7 expression by three independent shRNAs resulted in reduced colony formation and growth in semi-solid agar medium (Figs. 1g–i, S1A). To address the mechanism underlying growth reduction in KRAS^G12V^ cells following MCM7 knockdown, we assessed the presence of cleaved poly (ADP-ribose) polymerase (clPARP) and cleaved Caspase 3 as indicators for apoptosis. Cells with reduced MCM7 levels in a KRAS^wt^ background showed only a modest cleavage of PARP and Caspase 3. In contrast, KRAS^G12V^ expressing cells with reduced MCM7 levels exhibited an increase in clPARP and in cleaved Caspase 3 positive cells after seven days (Figs. 1j, k, and S1B, C).

**Fig. 2:**
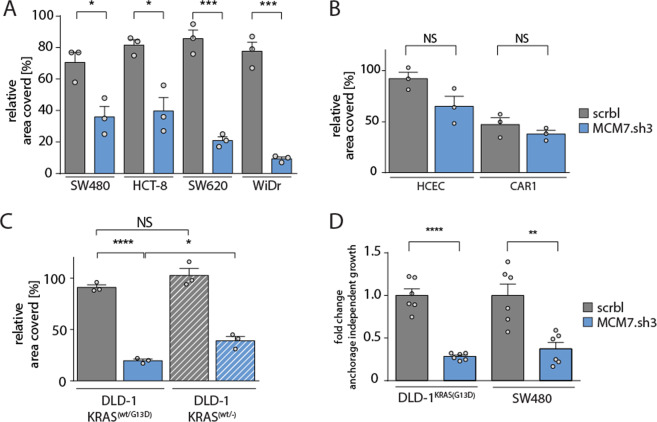
Consistent growth reduction of MCM7-suppressed colorectal cancer cell lines. **a** Clonogenic assay quantification of SW480 (KRAS^G12V^), HCT-8 (KRAS^G13A^), HT-29 (BRAF^V600E^), WiDr (BRAF^V600E^). All cell lines showed decreased growth after doxycycline induction of MCM7.sh3 compared to the control shRNA (scrbl). Mean percentage of the relative area covered by cells ± SEM is shown (*n* = 3 per group). Student’s *t*-test (two-sided); *p < 0.05; ***p < 0.001. **b** Clonogenic assay quantification of DLD1^KRAS(wt/−)^ and its parental cell line DLD1^KRAS(wt/G13D)^. Both cell lines are sensitive to MCM7 suppression, but DLD1 cells without mutated KRAS become more resistant to low levels of MCM7. Mean percentage of the relative area covered by cells ± SEM is shown (*n* = 3 per group). Student’s *t*-test (two-sided); NS = not significant; *p < 0.05; ****p < 0.0001. **c** Quantification of soft agar assays confirmed decreased anchorage-independent colony formation in KRAS mutated DLD1 and SW480 cells. Mean fold change of colonies ± SEM is shown (*n* = 6 per group). Student’s *t*-test (two-sided); **p < 0.01; ****p < 0.0001.

Next, we investigated the impact of MCM7 knockdown in other colorectal cancer cell lines harboring KRAS or BRAF mutations. We observed reduced colony formation of SW480, HCT-8, HT-29, and WiDR cell lines upon MCM7 knockdown (Fig. 2a). In contrast, the effect of MCM7 knock-down in KRAS/BRAF wildtype cells HCEC and CAR1 was not significantly different from the scrbl-control shRNA effect and only low levels of cleaved Caspase-3 (>5%) were measured (Figs. 2b, S2E). The comparison of MCM7 suppression in DLD-1 cells (KRAS^wt/G13D^) and its isogenic derivative line lacking the mutated KRAS allele (KRAS^wt/−^) showed a clear growth reduction in both cell lines however, DLD-1 KRAS^wt/G13D^ cells were more sensitive to reduced MCM7 levels than the KRAS-knockout cell line (Fig. 2c). The reason for the high sensitivity of DLD-1 KRAS^wt^ cells is unclear, however, these cells likely have adapted following KRAS^G13D^ depletion and thus might not represent a true wildtype situation. MCM7-suppressed DLD-1 KRAS^wt/G13D^ and SW480 KRAS^G12V^ cells additionally showed strongly reduced anchorage-independent proliferation (Fig. 2d). DLD-1 KRAS^wt/-^ cells exhibited higher levels of pERK as compared to DLD-1 KRAS^wt/G13D^ cells (Fig. S2B). Thus, we investigated the impact of MEK inhibition onto the sensitivity of these cells towards MCM7 suppression. Already low levels of MEK1,2 inhibitor AZD6244, which did not significantly influence cell growth and colony formation, dramatically lowered the sensitivity of both KRAS^wt/−^ and KRAS^wt/G13D^ DLD-1 cells towards MCM7 suppression (Fig. S2B). This indicates that even low activation levels of pERK increase the need for colorectal cancer cells of a high amount of the replication origin MCM complex.

### Mutant KRAS expression causes replicative stress in cells with MCM7 suppression

**Fig. 3:**
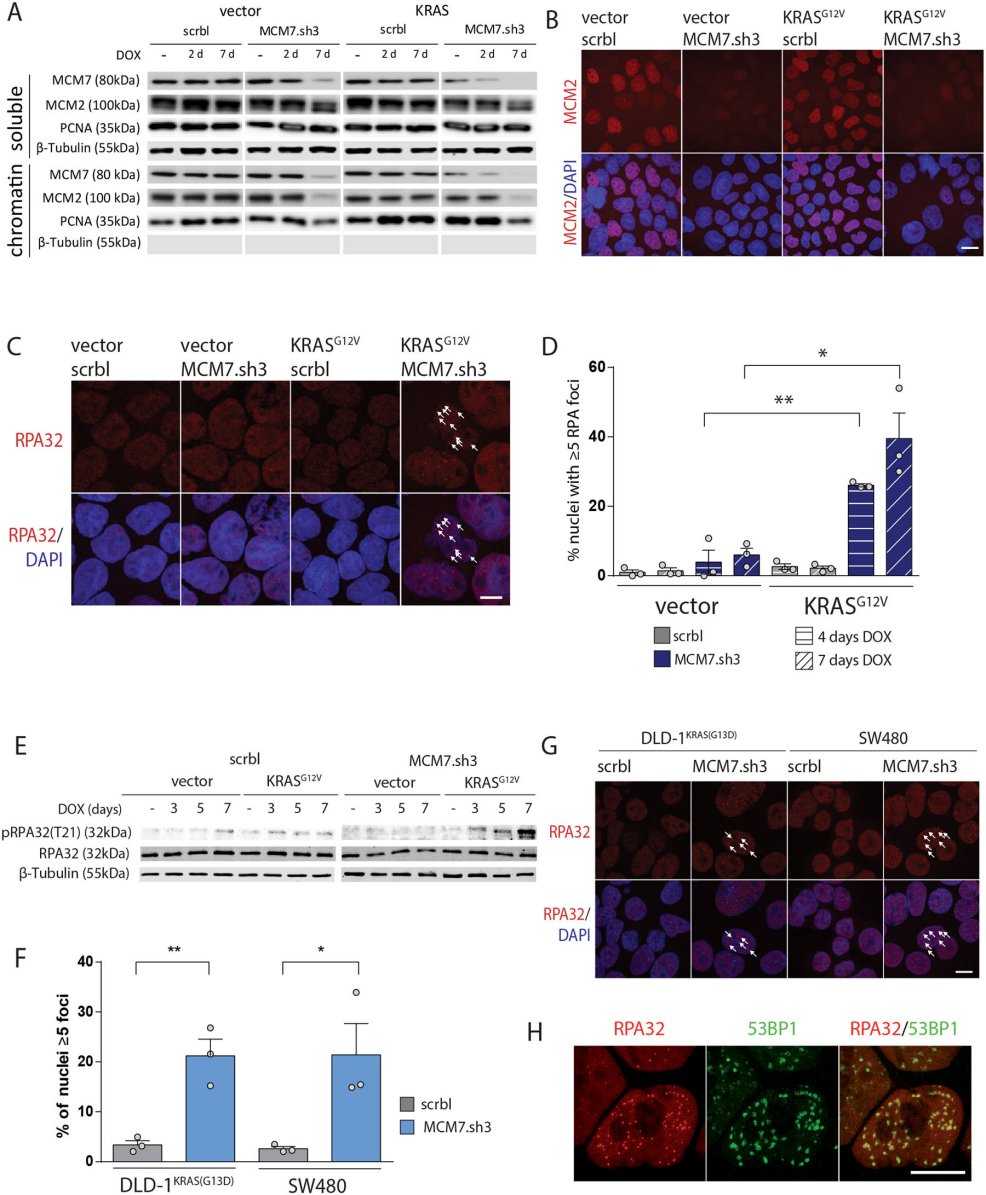
Cells with MCM7 knockdown show increased replication stress after KRAS^G12V^ expression. **a** Protein fractionation and subsequent western blot analysis showed a potent knockdown of MCM7 of the soluble as well as the chromatin-bound protein fraction. MCM2 levels are exclusively depleted from the chromatin-bound fraction. β-tubulin and PCNA serve as loading controls for the soluble and chromatin-bound fraction, respectively. **b** In situ chromatin fractionation of adherent cells followed by immunofluorescence staining shows a robust depletion of MCM2 from the chromatin after 7 days of MCM7 knockdown. Scale bar = 20 µm. **c** Immunofluorescence after 7 days of doxycycline induction showed RPA32 foci formation in CaCo2 cells after simultaneous KRAS^G12V^ and MCM7.sh3 expression. Arrows indicate RPA foci. Scale bar = 10 µm. **d** Quantification of nuclei with 5 or more RPA32 foci showed a significant increase of RPA32 focalization only when KRAS^G12V^ and MCM7.sh3 were co-expressed in CaCo2 cells. After 4 and 7 days, ∼26% and ∼40% of cells exhibited 5 or more RPA32 foci, respectively. Mean percentage of cells with 5 or more RPA32 foci ± SEM is shown (*n* = 3 per group). At least 50 cells per experiment were counted. Student’s *t*-test (two-sided); *p < 0.05, **p < 0.01. **e** Western blot shows specific phosphorylation of RPA32 in KRAS^G12V^ expressing CaCo2 cells after MCM7 knockdown. β-tubulin serves as a loading control. **f** Quantification of immunofluorescence reveals an increase of cells with RPA foci after four days of MCM7 knockdown in DLD1 and SW480 cells. Mean percentage of cells with ≥5 RPA foci ± SEM is shown (*n* = 3 per group). At least 50 cells per experiment were counted. Student’s *t*-test (two-sided); *p < 0.05; **p < 0.01. **g** Immunofluorescence after 4 days of doxycycline induction showed RPA32 foci formation in DLD-1 and SW480 cells after MCM7.sh3 expression. Arrows indicate RPA foci. Scale bar = 10 µm. **h** CaCo2 cells expressing KRAS^G12V^ and MCM7.sh3 show colocalization of RPA and 53BP1 foci after 7 days of doxycycline induction. Scale bar = 10 µm.

MCM7 is an essential component of the replicative helicase MCM2-7. The hexameric MCM2-7 protein complex ensures correct DNA replication licensing and accurate duplication of the genome in a tightly regulated multistep process^24^. First, loading of MCM2-7 to the origins of replication confers replication licensing prior to S phase^25–27^. This process is strictly limited to late M and G1 to prevent DNA re-replication during S phase^28,29^. Inhibition of this DNA replication origin licensing via depletion of MCM subunits or other licensing factors has been suggested to activate a licensing checkpoint that prevents cells from entering S-phase with an under-licensed genome^30,31^. Upon entry into S phase, origin firing, which initiates DNA replication, is facilitated by phosphorylation of the MCM complex by the DFb4-dependent CDC7 kinase (DDK) and cyclin-dependent kinases CDK, most likely CDK2^24^. Activated oncogenes are known to interfere with replication initiation and its regulation. Increased replication origin firing and altered fork progression can result in senescence or apoptosis^9^ (reviewed in ref. ^32^). To understand the consequences of MCM7 suppression in CaCo2 KRAS^G12V^ cells, we investigated the integrity of the MCM2-7 complex and sought for evidence of enhanced replication stress. Following introduction of MCM7.sh3 into CaCo2 cells, we observed efficient depletion of MCM7 in the soluble and chromatin-bound fractions prepared from cellular lysates. MCM7 suppression further resulted in the loss of MCM2 in the chromatin fraction, but not in the soluble fraction (Figs. 3a, b). This suggests that the knockdown was specific for MCM7 but the suppression of MCM7 led to a disruption of proper complex formation or recruitment to the DNA as it was previously described^33^.

Replication Protein A (RPA) focalization serves as a hallmark of replication stress. RPA focalization occurs when stretches of non-replicated, single-stranded DNA are bound upon replication fork stalling^34–36^. Following MCM7 knockdown, visualization of the RPA32 subunit by immunofluorescence revealed a substantial increase in RPA focus formation in approximately 26% of CaCo2 cells at day 4 and 40% at day 7 post KRAS^G12V^ induction. Neither KRAS^G12V^ expression nor MCM7 suppression alone resulted in a significant increase of cells with RPA foci (Fig. 3c, d). In line with this, RPA32 was increasingly phosphorylated after MCM7 knockdown specifically in KRAS^G12V^ CaCo2 cells (Fig. 3e). Increased RPA focus formation was also observed in KRAS mutant DLD1 (KRAS^wt/G13D^) and SW480 (KRAS^G12V/G12V^) cells following MCM7 suppression (Fig. 3f, g). Interestingly, RPA foci co-localized with 53BP1, which is one of the major players in double-strand break repair and has been implicated in the repair of under-replicated DNA after mitosis^37,38^ (Fig. 3h). In agreement with the observed replicative stress, increased CHK1 activation was detected specifically in CaCo2 cells with simultaneous KRAS^G12V^ expression and MCM7 knockdown. In contrast, CHK2 was activated after MCM7 suppression, yet was independent of KRAS^G12V^ expression (Fig. S3A).

The source of mutant KRAS-generated replication stress in the background of low replication licensing is still elusive. Previously, expression of oncogenic RAS has been linked to replication stress due to an increase in replication origin firing^9,32^. To test the influence of mutant KRAS on origin firing, we inhibited CDC7 pharmacologically with PHA 767491 in two isogenic cell lines. We found that KRAS mutant cells were partially able to counteract the CDC7 inhibition and thus showed increased resistance to CDC7 inhibition (Fig. S3B–F), indicating that mutant KRAS indeed promotes origin firing potentially by activating CDC7.

### Replication stress in mutant KRAS cancer cells leads to DNA damage in mitosis followed by mitotic catastrophe in MCM7-suppressed cells

**Fig. 4:**
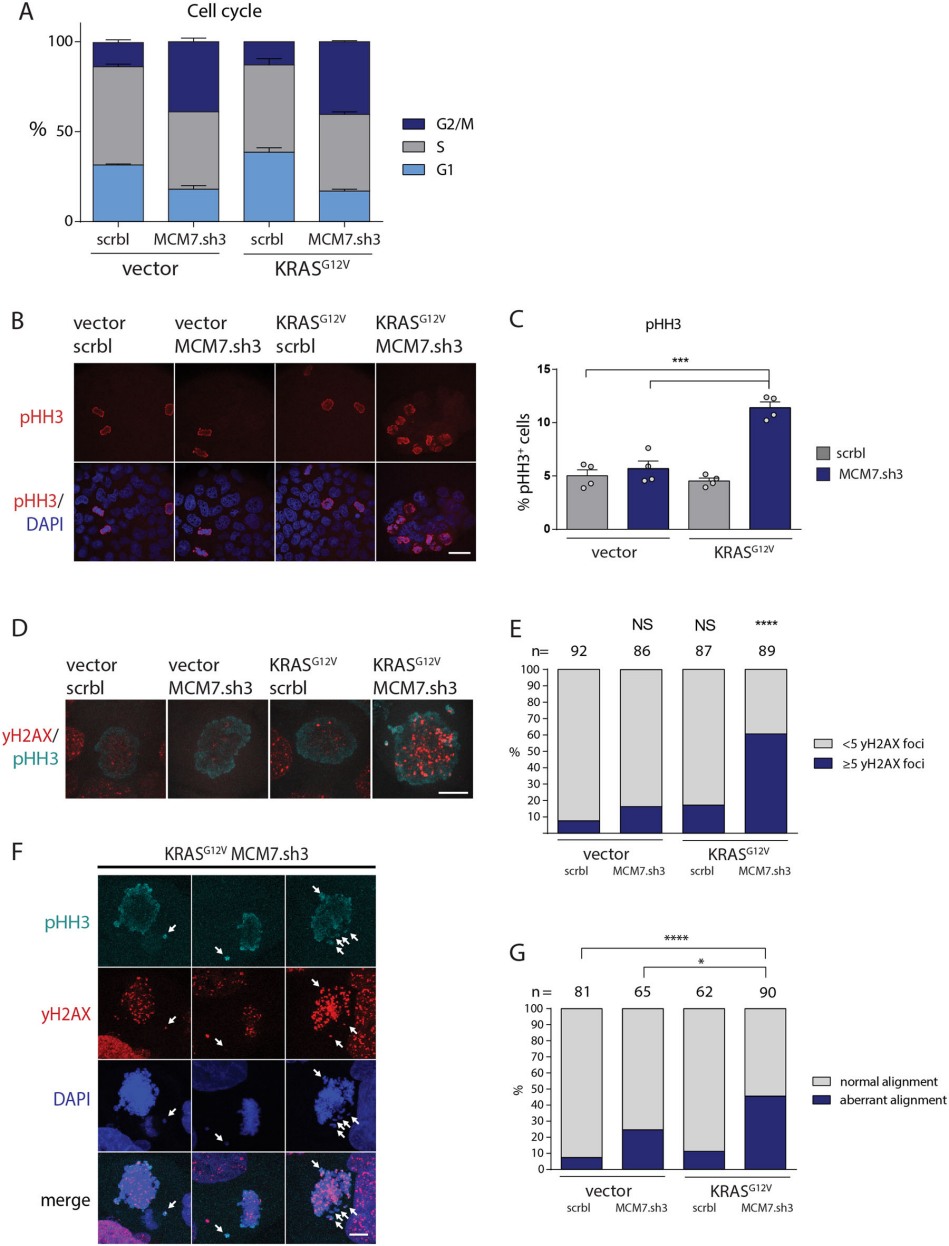
Induction of mutant KRAS expression and MCM7 suppression drives cells into mitotic catastrophe. **a** Expression of KRAS^G12V^ and MCM7.sh3 in CaCo2 cells were induced with doxycycline for 7 days. BrdU was added to the cells 30 min before they were harvested and stained with propidium iodide (PI) and an anti-BrdU-AF488 antibody. FACS analysis reveals increased persistence of CaCo2 cells with low levels of MCM7 in G2/M phase independently of KRAS^G12V^ seven days after induction. (*n* = 2 per group). **b**, **c** Cells were stained for phospho-Histone H3 (pHH3) and counted. About 5% of all cells stained positive for pHH3 after 7 days of induction except for cells co-expressing KRAS^G12V^ and MCM7.sh3, which had an increased mitotic cell number of over 11%. Scale bar = 40 µm. Mean percentage of pHH3^+^ cells ± SEM is shown (*n* = 4 per group). At least 200 cells per experiment were counted. Student’s *t*-test (two-sided); ***p < 0.001. **d**, **e** Co-staining of pHH3 and γH2AX revealed that the majority of mitotic CaCo2 cells exhibit DNA damage in mitosis after co-expression of KRAS^G12V^ and MCM7.sh3, whereas KRAS^G12V^ or MCM7.sh3 expression alone, did not cause a significant increase of mitotic cells with 5 or more γH2AX foci. A total of at least 86 cells per group were counted through the course of 5 independent experiments. Fisher’s exact test; ****p < 0.0001. **f**, **g** CaCo2 cells show an increased number of cells with DNA damage and randomly distributed chromosomes throughout the cell during mitosis after 7 days of KRAS^G12V^ expression and MCM7 knockdown. Arrows indicate misaligned chromosomes. Scale bar = 10 µm. Fisher’s exact test; *p < 0.05; ****p < 0.0001.

To assess how KRAS mutation and MCM7 suppression affect the regulation of the licensing checkpoints, we analyzed the cell cycle distribution in CaCo2 cells seven days after MCM7 knockdown with or without KRAS^G12V^ expression by flow cytometry. While KRAS^G12V^ expression alone did not significantly change cell cycle distribution (Fig. 4a), MCM7-suppressed CaCo2 cells accumulated in G2/M, however, this was independent of KRAS^G12V^ expression. Interestingly, knockdown of MCM7 did not lead to accumulation in G1, indicating that the licensing checkpoint is not being activated in CaCo2 cells. This is in line with previous reports that cancer cells accumulate in G2 after suppression of licensing factors^33^. To further characterize the point of arrest, we analyzed phosphorylation of Histone H3 (pHH3) at serine 10, which is specific for mitotic cells^39^. Remarkably, MCM7-suppressed KRAS^G12V^ expressing cells showed a robust increase of cells in mitosis (Figs. 4b, c, S4A). In contrast, KRAS^G12V^ expression or knockdown of MCM7 alone had no major influence on the number of cells in mitosis even after seven days of KRAS^G12V^ activation. This result indicates that lowering MCM7 levels halted cells in G2, yet cells entered mitosis eventually.

Premature entry into mitosis in the presence of DNA perturbations such as under-replicated DNA followed by a mitotic arrest are hallmarks of mitotic catastrophe^40–42^. Therefore, we hypothesized that the underlying mechanism of oncogenic KRAS-dependent cell death in MCM7-suppressed cells might be mitotic catastrophe. To test this, KRAS^G12V^ and MCM7.sh3 expression was induced for seven days and pHH3-positive mitotic cells were analyzed for DNA damage by determining the phosphorylation of histone H2AX at Ser139 (γH2AX). We found a striking increase in DNA damage during mitosis in cells with simultaneous KRAS^G12V^ expression and MCM7 knockdown (Figs. 4d, e, S4B). In contrast, cells with either KRAS^G12V^ expression or MCM7 knockdown alone showed no significant increase of *γ*H2AX foci in mitosis as compared to control cells. Simultaneous KRAS^G12V^ expression and MCM7 knockdown also induced massive chromosomal aberrations during mitosis and random distribution of condensed chromosomes throughout the cell - a characteristic for cells undergoing mitotic catastrophe^41^ (Figs. 4f, g, S4C). These data show that cells harboring mutant KRAS are specifically sensitive to decreased replication origin licensing by MCM7 suppression, which results in mitotic catastrophe and apoptosis.

### KRAS mutant cells are more sensitive towards agents causing under-replicated DNA

To assess whether KRAS mutant cells are generally sensitive towards under-replication, we exposed them to hydroxyurea and oxaliplatin, both of which perturb DNA synthesis albeit through different mechanisms. Hydroxyurea depletes the dNTP pool by inhibiting ribonucleotide reductase and during DNA replication prevents the incorporation of nucleotides leading to stalled replication forks^43,44^. Oxaliplatin inhibits DNA replication by generating inter- and intra-strand crosslinks^45^. We indeed observed an increased sensitivity of CaCo2 cells towards hydroxyurea and oxaliplatin when KRAS^G12V^ was expressed (Fig. S5), further suggesting increased sensitivity of KRAS mutant cells to therapeutically induced replication stress.

## Discussion

KRAS is one of the most commonly mutated oncogenes, but clinically effective anti-RAS therapies remain elusive^1,2^. The concept of synthetic lethality is expected to help identifying specific vulnerabilities of cells expressing this oncogene. Here we demonstrate that the suppression of MCM7 is synthetic lethal in KRAS mutated colorectal cancer cells, which specifically rely on high MCM7 levels. In contrast, KRAS wild-type cells only exhibited minimal sensitivity to MCM7 depletion.

Forced oncogenic RAS expression has previously been associated with replication stress via depletion of deoxy-ribonucleotide triphosphate levels^46,47^, production of large amounts of the reactive oxygen species superoxide^48,49^, or aberrant origin firing^9^. We find that endogenous or weak ectopic expression of mutant KRAS did not cause detectable replication stress by itself, suggesting that cells harboring endogenous mutant KRAS levels have adapted to oncogene-induced stress by activation of DNA damage repair^50^. Our results support the notion that even low levels of oncogenic KRAS in a KRAS wild-type background prime the cells for susceptibility to additional stress in the form of decreased replication origin licensing upon disruption of the MCM2-7 complex. Earlier reports have shown that oncogenic RAS expression decreases the distance between active replicons, an indication that RAS directly drives firing of otherwise dormant origins^9,32^. This is further supported by our observation that KRAS mutant cells exhibited increased resistance towards pharmacological inhibition of CDC7. The CDC7 kinase mediates MCM2-7 complex phosphorylation prior to DNA replication. Presumably mutant KRAS is able to trigger DNA replication directly through stimulation of CDC7 kinase activity. CDC7 forms a complex with its regulatory subunit ASK/Dfb4 that facilitates ATP binding and plays an essential role in the initiation of DNA replication by phosphorylation of MCM2 and MCM4. ERK and CK2α were recently found to promote DNA replication in an EGFR dependent manner in human glioblastoma cells by decreasing an ADP-dependent negative feed-back regulation acting on CDC7/ASK^51^. The mechanism of how an increase in origin licensing is mediated via oncogenic KRAS in colorectal cancer cells remains elusive. However, several lines of evidence suggest that also in colorectal cancer cells KRAS^G12V^-induced MAPK signaling is likely to exert an influence on DNA replication by targeting several components of the DNA replication machinery at different levels. First, we found that treatment of cells with low MEK inhibitor concentrations dramatically reduced the sensitivity towards MCM depletion, indicating a direct effect of MEK or ERK kinases. Additionally, MCM7 itself is a known MAPK target^20^. Furthermore, MCM3 and the ATR kinase, which phosphorylates MCM3 upon replication stress, have been identified as (indirect) ERK targets^52^. Thus, it remains to be determined to what extend activated RAS–MEK–ERK signaling in colorectal cancer cells impinges on both expression and phosphorylation-dependent activation of replication complex members to foster origin firing.

Aberrant or increased origin licensing and firing in mutant KRAS-expressing cells potentially reduces the number of dormant origins, which comprise the vast majority of licensed origins. Dormant origins serve as a back-up during S-phase to resolve perturbations such as replication fork stalling^33,53–57^. KRAS^G12V^ expression upon MCM7 depletion increased the fraction of mitotic cells, which at the same time exhibited DNA damage. This effect indicated unresolved replication stress in KRAS mutant cells as a consequence of under-replicated stretches of DNA, which occurred in S-phase exhibiting low MCM levels. The presence of under-replicated DNA during mitosis is known to hinder proper chromosome segregation during mitosis and to promote mitotic arrest followed by mitotic catastrophe^58,59^. Mutant RAS could further contribute to an increased entry into mitosis despite the presence of under-replicated DNA by overwriting the G2/M checkpoint via ERK-mediated activation of p90^RSK^ ERK^10,60^. However, we did not find evidence for this mechanism as no significant increase in p90^RSK^ was detected in KRAS^G12V^ cells upon depletion of MCM7 (data not shown). Alternatively, the observed phenotype could be due to the stimulation of transcription through mutant RAS. Recent evidence has suggested that strong overexpression of HRAS^G12V^ promoted the formation of R-loops, which interfered with replication fork progression and caused replication stress^61^. However, whether a similar mechanism is active in cells exhibiting low KRAS^G12V^ levels warrants further investigation.

Specifically targeting cancer cells by taking advantage of a unique vulnerability was first clinically manifested by the PARP inhibitor olaparib^62,63^. Despite substantial efforts related to RAS synthetic lethality screens, the identified targets in various KRAS-expressing models did not overlap or were not successfully replicated in vivo^16^. Yet, there is previous evidence that perturbing DNA replication licensing might be an attractive strategy to target KRAS mutated cells. HCT116 colorectal cancer cells were sensitive to the suppression of CDC6, the protein involved in the recruitment of the MCM complex to the origin of replication^64^. While our data further support the concept that KRAS mutant cells show increased sensitivity towards perturbations of DNA replication, it became clear that pharmacological inhibition of DNA replication initiation, e.g. by inhibition of CDC7 in cells harboring a conditionally expressed oncogene, does not suffice for induction of cell death in KRAS mutant cancer cells. Currently, CDC7 inhibitors are used as monotherapy for solid cancer in clinical trials (clinicaltrials.gov, identifiers: NCT03096054, NCT02699749). It is tempting to speculate that the inhibitors are less effective in KRAS mutant cancers due to their increased resistance.

MCM proteins are being regarded as prognostic tumor markers due to their frequent up-regulation in various cancer types^65,66^. Inappropriate expression of MCM2-7 and other pre-replicative complex proteins have been linked to pre-malignant dysplasia and cancer^67–70^. For example, patients with Meier-Gorline syndrome, which is caused by mutations in proteins required for replication licensing, show a primordial dwarfism phenotype but no other severe health compromising symptoms^71–73^. Notably, all MCM2-7 components are core essential genes^74^. However, MCM2-7 levels can be decreased several-fold in normal human cells without major effects, likely because most licensed origins remain dormant during S phase (∼80–90%) and serve as a back-up to resolve perturbations such as stalled replication forks^33,53–57^. This might predict a low toxicity of therapeutic compounds targeting replication licensing towards normal cells, an effect that became evident when we used KRAS and BRAF wildtype cells. Yet, targeting replication licensing might enable cancer cell-specific killing due to enhancement of replication stress in tumors expressing RAS oncogenes or other mutated components of RAS-mediated pathways. Additionally, the presence of the licensing checkpoint might prevent normal healthy cells from entering S-phase before the genome is fully licensed^30^.

In conclusion, we provide evidence that oncogenic KRAS expression sensitizes colorectal cancer cells to inhibition of DNA replication origin licensing by suppression of the replication factor MCM7. This concept is important to be considered in therapeutic approaches targeting licensing and replication components in KRAS mutant cancers yet is likely to provide a vulnerability for therapies inducing replication stress.

## Materials and methods

### Plasmids

KRAS^G12V^ expression was facilitated using a pSIN-TRE3G-KRAS^G12V^-IRES-TagBFP-PGK-HyroR plasmid. The shRNA library used in the screen was cloned into pMSCV-miRE-PGK-NeoR-IRES-mCherry. Subsequent validation of MCM7 was performed in pSIN-TRE3G-turboGFP-miRE-PGK-Neo^75^. To increase retrovirus production efficiency in Plat-E cells, gagpol containing viral group antigens and reverse transcriptase was used.

### shRNA library preparation

The library was comprised of 635 shRNAs targeting 121 genes and additional 6 control genes (Supplementary Table 1). The shRNAs were designed based on optimized design algorithms^76^. The constructs were synthesized as 136 bp oligomers on a 55K Agilent custom array by CustomArray Inc. and contained the respective shRNA sequence, an EcoR1 restriction site with miRE linker DNA, and a library-specific primer sequence (TTGTCCGCGTCGATCCTAGG). The shRNA sequences were amplified using 5′ miRE-XhoI forward (5′ TACAATACTCGAGAAGGTATATTGCTGTTGACAGTGAGCG 3′) and 3′ library-specific reverse primer (5′ TTGTCCGCGTCGATCCTAGG 3′) (1.0 ng template DNA, 1.5 µl dNTPs, 1.5 µl each primer (10 mM), 1.5 µl DMSO, 10 µl Phusion HF reaction buffer, 0.5 µl Phusion DNA polymerase in 50 µl reaction volume) (cycle conditions: 98 °C 1 min, 22 × [98 °C 10 s, 54 °C 30 s, 68 °C 25 s], 68 °C 5 min). The PCR products of 40 separate reactions were column purified (PCR purification kit, Qiagen), digested with EcoRI-HF and XhoI (4 h at 37 °C) and gel extracted, skipping the QG wash, instead washing with 750 µl and 500 µl PE (QiaQuick Gel extraction, Qiagen). The backbone (5ug) pMSCV-miRE-PGK-NeoR-IRES-mCherry was digested with EcoRI-HF and XhoI for 3 h at 37 °C, heat inactivated for 20 min 65 °C, dephosphorylated with 1 µl of calf intestinal alkaline phosphatase (New England Biolabs) to the reaction for 1 h at 37 °C and gel extracted (QiaQuick Gel extraction, Qiagen). Inserts and backbone were quantified and ligated overnight at 15 °C in a molar insert to backbone ratio of 2.5:1. Ligation product was then phenol extracted and NaOAc/EtOH precipitated at −20 °C overnight. DNA was recovered in 8 µl EB and 2.0 µl of the ligation product was transformed four times into 20 *μ*l MegaX DH10B T1 electrocompetent cells (Invitrogen) by electroporation (2.0 kV, time constant 4–5). Electrocompetent cells were subsequently plated on 4 × 15 cm dishes overnight. Cells were washed off the plates, incubated in 4×500 ml LB-Ampicillin at 37 °C for 4–6 h. The plasmids were then isolated using Qiagen Plasmid Maxi kit. Virus production and cell transduction was performed according to methods part “Retrovirus production and transduction”.

### RNAi screen

CaCo2 cells with doxycycline-inducible KRAS^G12V^ were transfected by retroviral transduction with a custom-designed shRNA library containing 625 shRNAs against 121 different targets (Supplementary Table 1; purchased from Integrated DNA Technologies) with less than 20% transduction efficiency to predominantly integrate a single shRNA construct per cell. This was achieved by diluting the retrovirus library 1:7. Three days after transfection and G418 selection, a fraction of cells was sorted for mCherry^+^ cells (shRNA^+^) using a FACSAria II and frozen down as the baseline control. The remaining cells were split in half and cultured in the presence or absence of doxycycline 21 more days before the two fractions were sorted for mCherry^+^ cells and frozen down. Before cell sorting, the cell populations were filtered through a 30 µm cell filter. The shRNA representation was kept over 1 000x at any given time.

To isolate the genomic DNA, the sorted cell pellets were resuspended in 400 µl DNA extraction buffer (10 mM Tris-HCl pH = 8.0, 150 mM NaCl, 10 mM EDTA). Then, 4 µl 10% SDS and 4 µl proteinase K (20 mg/ml stock) (Ambion) were added to the samples. After each addition, the samples were vortexed. Next, the samples were incubated at 55 °C in a shaker (800 rpm) for 16.5 h. The samples were then mixed with 400 µl phenol, transferred to PhaseLock tubes, and centrifuged at maximum speed for 8 min. The DNA containing aqueous solution was transferred to a fresh tube while leaving 30 µl of the aqueous in the tube to avoid contamination with the phenol soluble fraction. The phenol extraction was repeated two more times while adjusting for the phenol volumes according to the current volume of the aqueous fraction. To precipitate the genomic DNA, 31 µl 3 M sodium acetate pH = 5.2 (${\textstyle{1 \over {10}}}$ the volume of the aqueous solution), 930 µl 96% ethanol (3 volumes of the aqueous solution), and 1 µl Pellet Paint Co-Precipitant were added and mixed. The DNA was then left to precipitate at −20 °C overnight. On the next day, the DNA samples were centrifuged at maximum speed at 4 °C for 30 min. The resulting DNA pellet was then washed with 200 µl 70% ethanol and centrifuged for 5 min at 4 °C. The supernatant was discarded, and the DNA pellet was air dried for 5 min at 55 °C before it was resuspended in 40 µl elution buffer (EB, provided in QIAGEN Plasmid Mini Kit). To facilitate resuspension, the DNA solution was incubated for 1.5 h at 55 °C while shaking at 800 rpm. The final DNA concentrations across all samples were then equilibrated to 0.5 µg/µl.

Samples were then PCR amplified and barcoded (adapter primers: p5 SOLEXA adapter AATGATACGGCGACCACCGATGGATGTGGAATGTGTGCGAGG, p7loop_Barcode: CAAGCAGAAGACGGCATACGAXYXYTAGTGAAGCCACAGATGT, XYXY = barcoding nucleotides) (template DNA [0.5 µg/µl] 1.0 µl, H_2_O 36.5 µl, 10× PCR buffer 5.0 µl, MgCl_2_ 3.0 µl, dNTPs (2 mM each) 1.0 µl, p7loop_XYXY [10 µM] 1.5 µl, p5 SOLEXA adapter [10 µM] 1.5 µl, Amplitaq Gold 0.5 µl; cycling conditions: 10 min 95 °C, 33 × [30 s 95 °C, 45 s 52 °C, 60 s 72 °C], 7 min 72 °C). To prevent amplification artifacts, each sample was amplified in 10 separate reactions and the resulting PCR products were mixed.

To purify the amplified DNA, one round of Phenol extraction was performed. The DNA pellet was resuspended in 30 µl elution buffer (EB, provided in QIAGEN Plasmid Mini Kit) and run on a 2% agarose gel. The correct band at 345 bp was excised and purified using a QIAGEN QIAquick gel extraction kit according to the manufacturer’s instructions with following adjustments: samples were incubated for 10 min after addition of 1 volume isopropanol to increase precipitation, the optional washing step with QG buffer was skipped, samples were washed a second time with 0.5 ml PE buffer, and the DNA was eluted in 30 µl EB after 15 min of incubation at ambient temperature. The isolated DNA was then diluted to obtain a concentration of 100 nM (∼22.77 ng/µl for 345 bp) per sample. Deep sequencing was performed by the sequencing unit of the Campus Science Support Facilities (now Vienna BioCenter Core Facilities GmbH).

Raw sequencing reads measured by Illumina HiSeq 2500 were provided as binary aligned map (BAM) files. BAM files were converted into FASTQ files using BEDtools2 v2.2.3^77^. Counts were created by perfectly matching inverted reverse complement sequences for hairpin and barcode using the process Amplicon function of edgeR v3.8.6^78^. Samples were normalized by scaling the total count of each sample to the average total count of all samples. All processing steps following FASTQ file generation were conducted in R v3.2.1.

### Cell lines, antibodies, and inhibitors

Cell lines and respective culture conditions are specified in Supplementary Table 2. Antibodies used are listed in Supplementary Table 3. Hydroxyurea, Oxaliplatin, and PHA 767491 (CDC7i) were purchased from Sigma-Aldrich. Cell line identity was regularly validated by STR profiling (CLS Cell Lines Service GmbH, Eppelheim, Germany). HCET1T cells were additionally sequenced to proof absence of KRAS and BRAF mutations using an in-house colorectal cancer-specific gene panel as described recently^79^. Variants were called using the Torrent Variant Caller (TVC; Life Technologies) using the strict setting as specified by the IonTorrent Suite. A threshold of quality Q50 was applied to reduce false positive results and the in-house tool SoFIA was used for analyses^79^.

### Cell culture

Cell lines were cultured in their respective medium with antibiotic selection as indicated in Supplementary Table 2 with 10% fetal calf serum (PAN Biotech, P30-1506, Lot No. P130902) and 1% penicillin/streptomycin (Biochrom) at 37 °C and 5% CO_2_. Transgene expression was induced with 2 µg/ml of doxycycline (Applichem) where indicated. For drug treatment experiments, KRAS transgene expression was induced immediately after seeding and drugs were added one night after seeding the cells (PHA 767491, hydroxyurea, oxaliplatin—all Sigma-Aldrich).

### Retrovirus production and transduction

20 µg of the target plasmid and 7.5 µg of a plasmid encoding gag and pol were diluted in a total of 440 µl H_2_O and incubated at 37 °C for 5 h while shaking. Plat-E cells (HEK293T) were seeded at a confluence of 4 × 10^6^ cells in a 10 cm dish in 9 ml medium and cultured for at least 4 h. When cells were approx. 75% confluent, 10 µl PO_4_ solution (70 mM Na_2_HPO_4_, 70 mM NaH_2_PO_4_—all Sigma-Aldrich) was added to 0.5 ml 2× HBS (41.96 mM Hepes, 273.78 mM NaCl, pH to 7.15—all Sigma-Aldrich). 60 µl 2 M CaCl_2_ was mixed with the DNA solution and then added dropwise to the 2x HBS-PO_4_ solution while gently shaking. The mixture was incubated 1 min at room temperature and then dispersed dropwise onto the cells. 20 h after the transfection the medium was exchanged and reduced to 5 ml per plate. The virus-containing supernatant was harvested 34 h after transfection. The virus-containing supernatant was diluted 1:1 with medium and polybrene (Merck Millipore) was added in a dilution of 1:1200 to the final volume before the mixture was added to cell lines previously transduced with a lentivirus containing an ecotropic receptor for 24 h.

### Colony formation assays

Approximately 5 × 10^3^–1 × 10^4^ cells/well were seeded in 6-well plates and cultured with or without 2 µg/ml doxycycline or additional drugs as indicated in 2 ml medium. Culture medium was changed after approximately six days or every three to four days when using drugs. After 10–13 days cells were fixed and stained with 0.2% methylene blue (Applichem) in 50% methanol (J.T. Baker) for 20 min before washing the plates by submerging them in ambient temperature tap water. The percentage of the well area covered by colonies was quantified using a custom pipeline in CellProfiler^80^. Results were normalized to each respective control (no doxycycline or no drug).

### Soft agar assays

10^5^ CaCo2 cells or 10^3^ SW480/DLD1 cells were suspended in one part 37 °C warm 2× DMEM (CaCo2, SW480) or 2× RPMI-1640 (DLD1) and mixed with one part 42 °C warm 0.4% Noble Agar (BD Bioscience) in T25 flasks. After mixing, the flasks were incubated on ice for 30 min and subsequently incubated for 3 weeks (SW480, DLD1) or 5 weeks (CaCo2). All cell lines were induced with 2 µg/ml doxycycline at the time of seeding. The number of colonies was quantified using a custom pipeline in CellProfiler^80^.

### Protein lysate preparation and analysis

Cell lines were seeded at low confluency on 10 cm plates. Doxycycline was added at different times according to desired end points. All cell lines in a given experiment were harvested at the same time. For whole-cell extracts, cell pellets were lysed using M-PER buffer (Thermo Fisher) with PhosSTOP and cOmplete protease inhibitor cocktail (Roche) for 10 min on ice. For chromatin fractionation, the cell pellets were first lysed in CSK buffer (10 mM Hepes pH 7.4, 300 mM Sucrose, 100 mM NaCl, 3 mM MgCl_2_, 0.5% Triton-X-100 + PhosSTOP and cOmplete) for 15 min on ice before centrifuging the samples at 5000 × *g* at 4 °C for 5 min. The supernatant containing the soluble protein fraction was collected and snap-frozen in liquid N_2_. The pellets were washed twice with CSK buffer and snap-frozen in liquid N_2_. After rethawing, the pellets were lysed in RIPA buffer (50 mM Tris-HCI pH 7.4, 150 mM NaCl, 1% NP-40, 1 mM EDTA, 0.5% Na-deoxycholate + PhosSTOP and cOmplete) for 10 min on ice. Next, the lysate was sonicated twice for 10 min in intervals of 30 s (Diagenode Bioruptor) before centrifuging the lysate at max. speed for 10 min and collecting the supernatant containing the chromatin-bound fraction. Protein concentrations were quantified using BCA protein quantification kit (Pierce) and separated by SDS-PAGE using Mini-PROTEAN Tetra Handcast Systems (Bio-Rad). Western blots were quantified with ImageJ (https://imagej.nih.gov/ij/).

### Cell cycle analysis

Cells were seeded at low confluency and induced with 2 µg/ml doxycycline for 7 days. Prior to harvesting, the cells were treated with 20 µM BrdU (Sigma) for 30 min. Afterwards, cells were trypsinized, washed twice with PBS and fixed with ice-cold 70% ethanol. Next, cells were washed with 1 ml washing buffer (1% BSA, 0.2% Triton X-100 in PBS) twice and denatured in 2 M HCl + 0.2% Triton-X for 30 min at ambient temperature for 30 min under gentle agitation. Thereafter, cells were washed twice with washing buffer and blocked with blocking buffer (5% BSA, 0.2% Triton X-100 in PBS) for 1 h at ambient temperature under gentle agitation. The blocking buffer was replaced with anti-BrdU antibody (BD Bioscience #347580; 0.5 µg/ml) in blocking buffer and incubated for 1 h at ambient temperature under gentle agitation. Cells were washed twice with washing buffer and incubated with anti-mouse Alexa Fluor 488 (Invitrogen A-21204; 2 µg/ml) in blocking buffer under gentle agitation. Lastly, cells were washed with washing buffer and stained with PI staining solution (50 µg/ml Propidium iodide, 50 µg/ml RNase, 0.1% Triton X-100 in PBS) for 10 min at ambient temperature. Samples were analyzed with the BD Accuri™ C6 flow cytometer (BD Bioscience). Data were analyzed with FlowJo v10.

### Cleaved caspase 3 flow cytometry analysis

Cells were cultured in 10 cm plates for 3 days with 2 µg/ml doxycycline. Cells were then split and cultured for 4 more days with doxycycline to a total of 7 days. Medium and adherent cells were collected and fixed in 1 ml 2% formaldehyde for 10 min at ambient temperature before chilling the cells for 1 min on ice. Next, the cells were washed with 1 ml PBS and permeabilized in ice-cold 90% methanol on ice for 30 min, before storing the cells for up to several weeks at −20 °C. Subsequently, the fixed cells were washed twice with incubation buffer (0.5% BSA in PBS) and stained for 1 h with anti-Cleaved Caspase-3 Alexa Fluor® 647 (CST #9602) antibody at ambient temperature. Next, cells were washed in 500 µl incubation buffer, resuspended in 200 µl PBS, and analyzed with the BD Accuri™ C6 flow cytometer (BD Bioscience). Data were analyzed with FlowJo v10.

### pHH3 and *γ*H2AX flow cytometry analysis

All steps until the addition of the first antibody were performed identically to cleaved caspase 3 flow cytometry analysis. The cells were then incubated in 100 µl *γ*H2AX antibody solution (Millipore, 05-636, 1:250 in incubation buffer [0.5% BSA in PBS]) at ambient temperature for 1 h. The cells were then washed in 500 µl incubation buffer by centrifugation before they were incubated in 100 µl anti-mouse antibody conjugated to Alexa Fluor 488 (Invitrogen, A-21204, 1:1,000 in incubation buffer) at ambient temperature for 1 h. This was followed by three washing steps in 500 µl incubation buffer. After centrifugation, the cells were incubated in an 1:50 pHH3-PE (CST, 5764) solution at ambient temperature for 1 h. Finally, the cells were washed in 500 µl incubation buffer, centrifuged, resuspended in 200 µl PBS, and analyzed with the BD Accuri™ C6 flow cytometer. Data were analyzed with FlowJo v10.

### Immunofluorescence and confocal microscopy

Cells were seeded and induced with doxycycline 4 days prior fixation on poly-L-lysine (Sigma-Aldrich) coated coverslips. For the 7-day time point, cells were pre-induced 3 days before seeding. On the day of the staining, cells were washed once with PBS and fixed with 3% PFA in PBS + 0.1% Triton-X100 (Sigma-Aldrich) for 10 min. (In case of the in situ chromatin fractionation of adherent cells for the MCM2 staining, cells were, prior to fixation, incubated in ice-cold CSK buffer [protein lysate preparation section] for 15 min and then washed 2× with CSK buffer and 1× with PBS.) The fixation buffer was washed off 3× briefly and 2× for 5 min with PBS. Next, the cells were permeabilized with 0.3% Triton-X100 in PBS for 2 min before washing the cells again 3× briefly and 2× for 5 min with PBS. The cells were subsequently incubated in blocking buffer (3% BSA in PBS + 0.1% Tween-20) for 1 h before washing the cells briefly 2× with PBS-T (PBS + 0.1% Tween-20). Next, the cells were incubated with the primary antibody in blocking buffer for 1 h, washed 3× for 5 min with PBS-T, incubated in the secondary antibody in blocking buffer for 1 h, and washed again 3× for 5 min with PBS-T. In order to stain for DNA, the cells were incubated in 2 µg/ml DAPI (in PBS) for 15 min and washed 2× with dH_2_O. The coverslips were mounted in VECTASHIELD HardSet Mounting Medium and stored at 4 °C. All steps were carried out at ambient temperature. Pictures were taken with a Leica TCS SPE confocal system.

### cDNA synthesis and quantitative polymerase chain reaction (qPCR)

Cells were seeded in 6-well plates and induced for two days with doxycycline before RNA was collected using a QIAGEN RNeasy Mini kit according to the manufacturer’s protocol. The collected RNA was transcribed to cDNA using the High-Capacity cDNA ReverseTranscription Kit (Roche) according to the manufacturer’s protocol. Quantitative PCR (qPCR) was performed using the Promega GoTaq qPCR Master Mix. First, the transcribed cDNA was diluted 1:100 in nuclease-free H_2_O. Then 1 µl of diluted cDNA was added to 7.5 µl SybrGreen Master Mix and 5.5 µl nuclease-free H_2_O. The total 14 µl were pipetted on a 96-well PCR plate, before adding 1 µl of the primer mix to reach a final primer concentration of 333 nM per primer (MCM7 fwd: GTTTGACCTCCTCTGGCTGA - rev: AGAGGTTCAAACTGGGAGGG; UBE2D2 fwd ATGGCGCTGAAGAGGATTC - rev CTTGCCAGTGGAACAAGTCA). The qPCR was run on an Applied Biosystems StepOnePlus Real-Time PCR System using the cycle conditions: initial denaturation 95 °C for 2 min, denaturation 95 °C for 15 s, primer annealing 60 °C for 30 s (40 cycles). The Ct values (threshold cycle value) were normalized to the values of the endogenous control UBE2D2 (∆Ct values). Next, the ∆Ct values were normalized to the control sample (∆∆Ct values).

### Statistical analysis

Unpaired, two-sided Student’s *t*-tests, Fisher’s exact test, and nonlinear regressions were performed using GraphPad Prism 6. To assess statistically significant differences in drug sensitivity, the drug concentrations were first transformed on a log_10_ scale. All data points were then normalized in a way that the smallest and highest value in each data set represents 0% and 100%, respectively. On these data, a nonlinear regression was performed using the corresponding “log(inhibitor) vs. normalized response - Variable slope” setting. The curves were compared using the extra sum-of-squares *F* test. Alternatively, the IC_50_ values of every single replicate were calculated individually and subsequently compared via an unpaired, two-sided Student’s *t*-test.

### shRNA sequences

97meres scrbl: TGCTGTTGACAGTGAGCGAGCGAAGATGCTCAGATGAATATAGTGAAGCCACAGATGTATATTCATCTGAGCATCTTCGCATGCCTACTGCCTCGGA. MCM7.1879 TGCTGTTGACAGTGAGCGCCAGGCTAATGGAGATGTCAAATAGTGAAGCCACAGATGTATTTGACATCTCCATTAGCCTGATGCCTACTGCCTCGGA. MCM7.1534v TGCTGTTGACAGTGAGCGACCCGACCGAGACAATGACCTATAGTGAAGCCACAGATGTATAGGTCATTGTCTCGGTCGGGCTGCCTACTGCCTCGGA. MCM7.sh3 TGCTGTTGACAGTGAGCGAAGAGGAGGATTTCTACGAAAATAGTGAAGCCACAGATGTATTTTCGTAGAAATCCTCCTCTGTGCCTACTGCCTCGGA.

## Supplementary information

Suppl Figure 1

Supplementary Figure 2

Supplementary Figure 3

Supplementary Figure 4

Supplementary Figure 5

Table S1 - shRNA library

Table S2 - cell lines and culture media

Table S3 - Antibodies

Supplementary Figure Legends and Supplementary Tables
