# Supplementary figures and images for: Reduced replication origin licensing selectively kills KRAS-mutant colorectal cancer cells via mitotic catastrophe
Source: Cell Death Dis. 2020 Jul 1;11(7):499. doi: 10.1038/s41419-020-2704-9 (PMC7330027; doi:10.1038/s41419-020-2704-9)

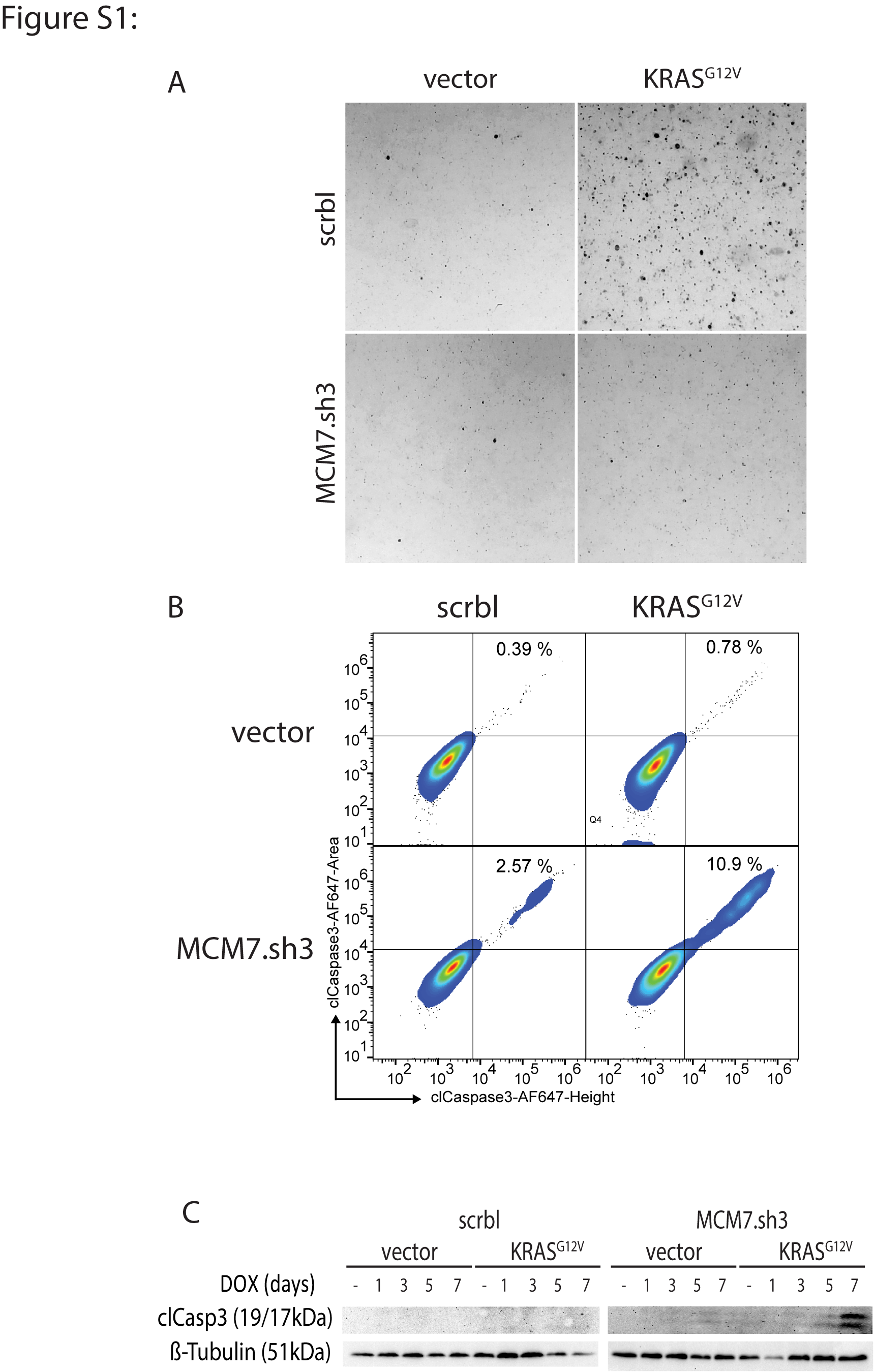

Supplement: Supplementary file 1 — Suppl Figure 1 [file 41419_2020_2704_MOESM1_ESM.tif]

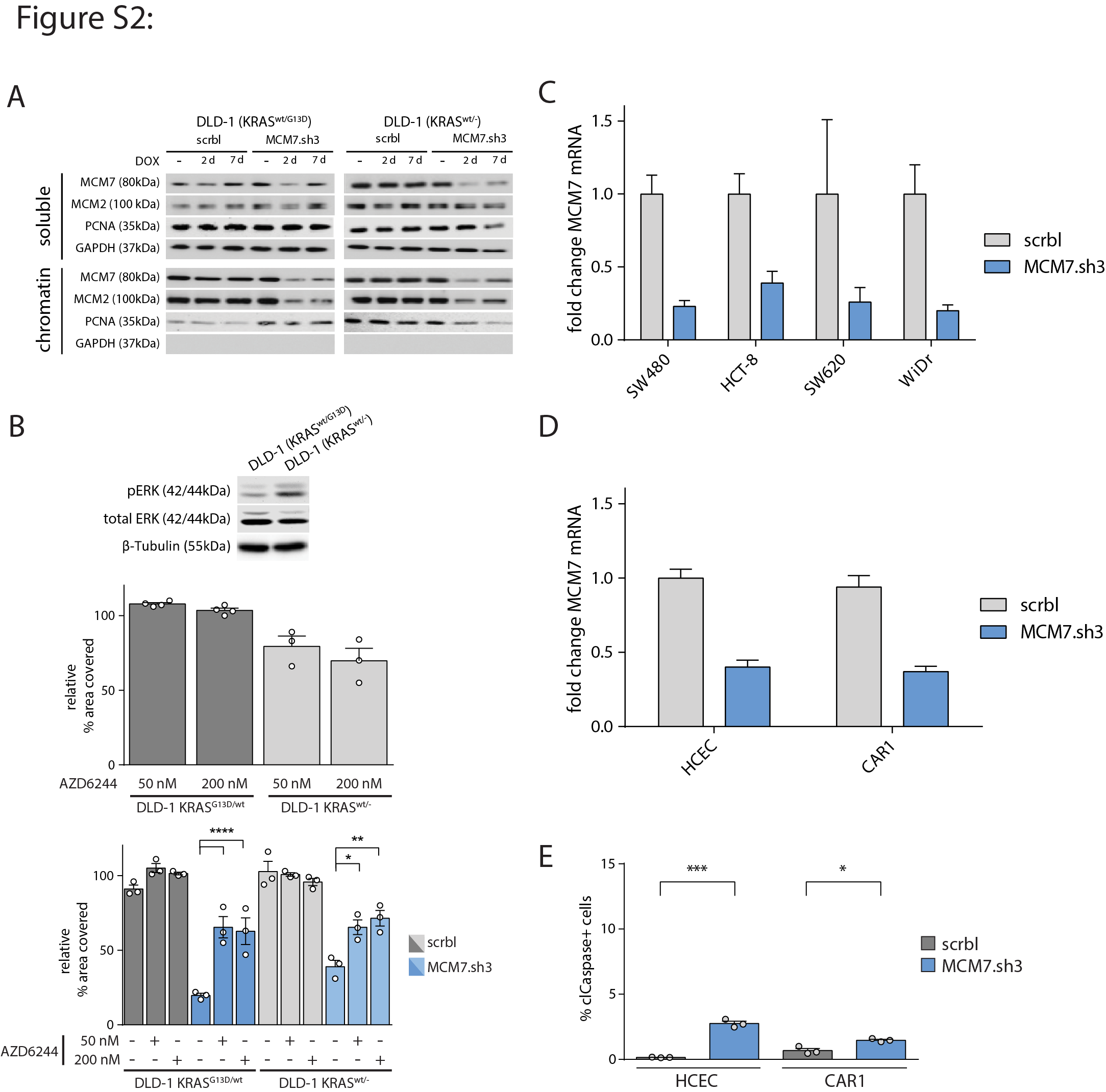

Supplement: Supplementary file 2 — Supplementary Figure 2 [file 41419_2020_2704_MOESM2_ESM.png]

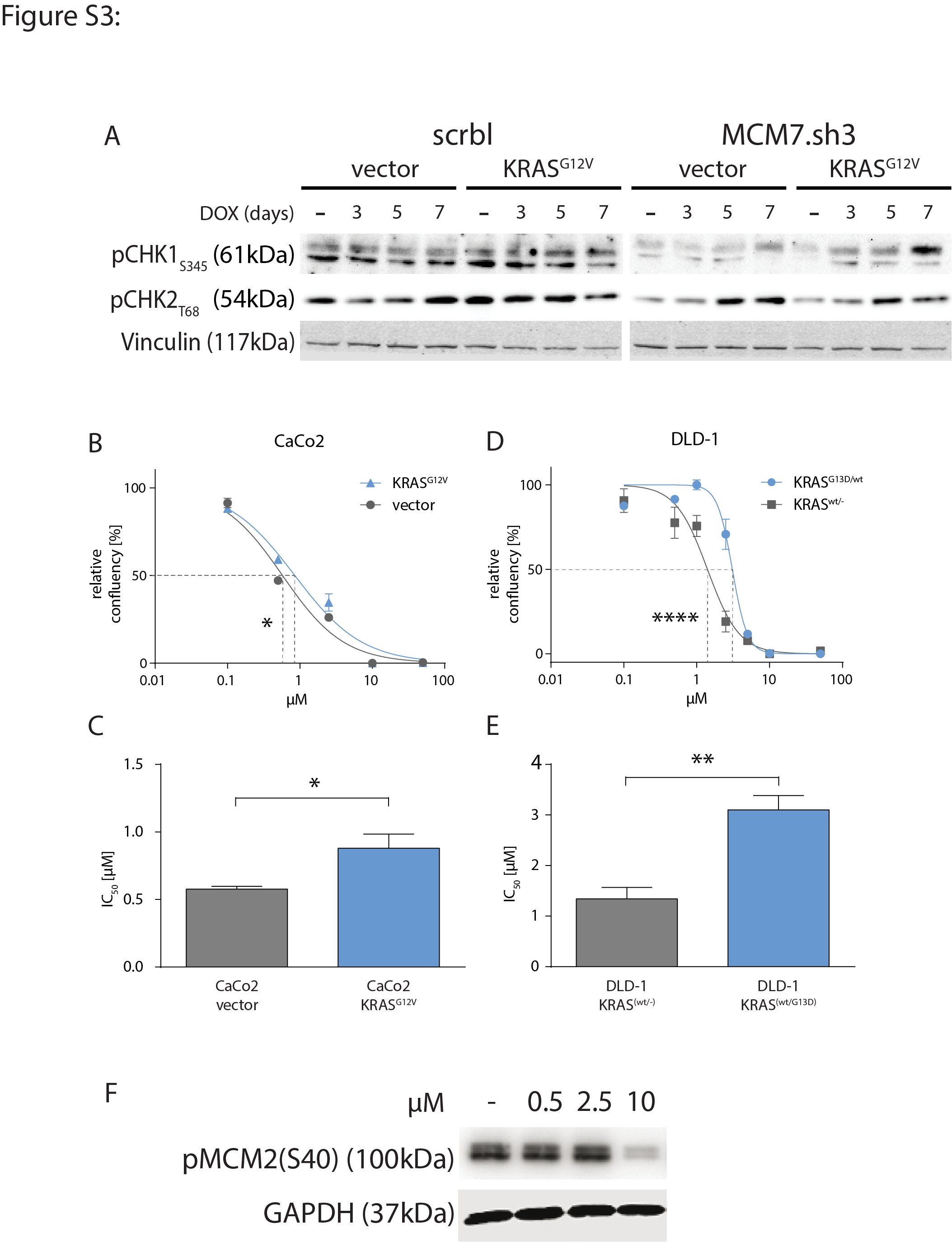

Supplement: Supplementary file 3 — Supplementary Figure 3 [file 41419_2020_2704_MOESM3_ESM.png]

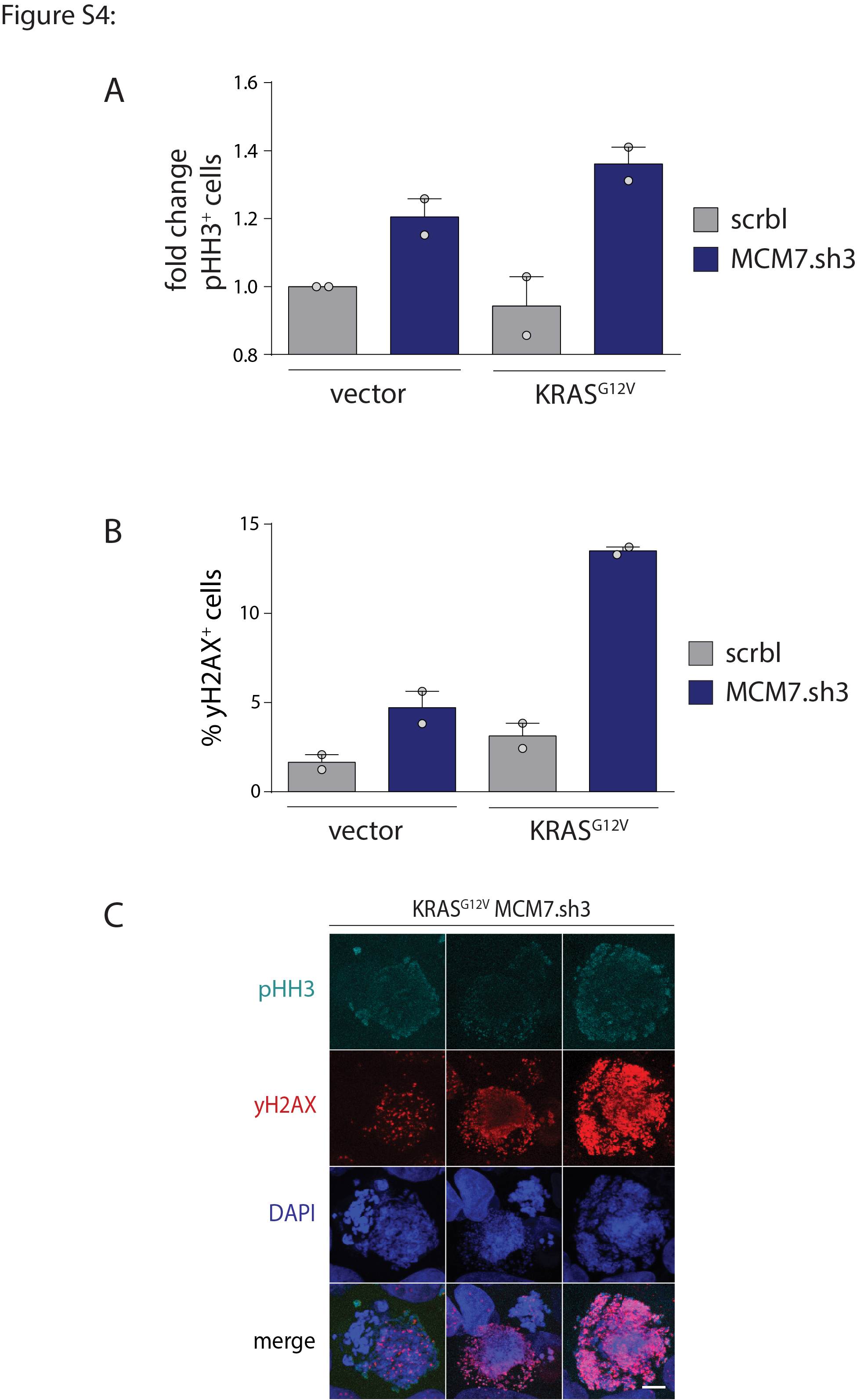

Supplement: Supplementary file 4 — Supplementary Figure 4 [file 41419_2020_2704_MOESM4_ESM.png]

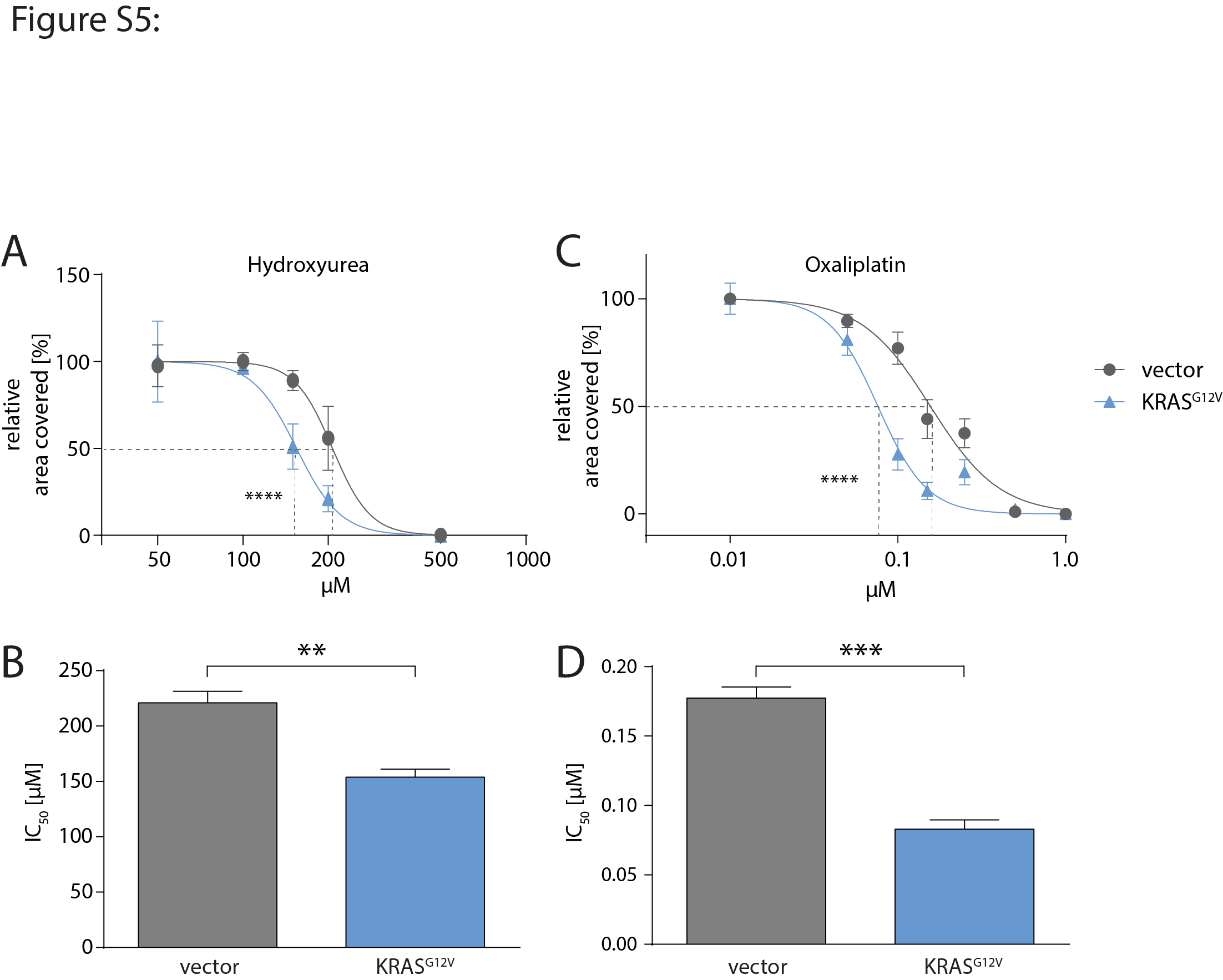

Supplement: Supplementary file 5 — Supplementary Figure 5 [file 41419_2020_2704_MOESM5_ESM.png]
