# Supplementary material for: Reduced replication origin licensing selectively kills KRAS-mutant colorectal cancer cells via mitotic catastrophe
Source: Cell Death Dis. 2020 Jul 1;11(7):499. doi: 10.1038/s41419-020-2704-9 (PMC7330027; doi:10.1038/s41419-020-2704-9)
Supplement: Supplementary file 6 — Table S1 - shRNA library [file 41419_2020_2704_MOESM6_ESM.pdf]

Gene Symbol	shRNA Name	Guide	97mer
**DUSP4**	DUSP4.1283	TTAAGCATAATTATTCATCTGT	TGCTGTTGACAGTGAGCGCCAGATGAATAATTATGCTTAATAGTGAAGCCACAGATGTATTAAGCATAATTATTCATCTGTTGCCTACTGCCTCGGA
**DUSP4**	DUSP4.31	TTTTAGAACAGAATTCTGGGTA	TGCTGTTGACAGTGAGCGCACCCAGAATTCTGTTCTAAAATAGTGAAGCCACAGATGTATTTTAGAACAGAATTCTGGGTATGCCTACTGCCTCGGA
**DUSP4**	DUSP4.1193	TACAACAACGACAACAAAGGGA	TGCTGTTGACAGTGAGCGCCCCTTTGTTGTCGTTGTTGTATAGTGAAGCCACAGATGTATACAACAACGACAACAAAGGGATGCCTACTGCCTCGGA
**DUSP4**	DUSP4.1545	TTAAAAAGCAATTCAAACCTAA	TGCTGTTGACAGTGAGCGCTAGGTTTGAATTGCTTTTTAATAGTGAAGCCACAGATGTATTAAAAAGCAATTCAAACCTAATGCCTACTGCCTCGGA
**DUSP4**	DUSP4.2934	TATTTCTAGAGGAAGCAGGGAG	TGCTGTTGACAGTGAGCGATCCCTGCTTCCTCTAGAAATATAGTGAAGCCACAGATGTATATTTCTAGAGGAAGCAGGGAGTGCCTACTGCCTCGGA
**DUSP6**	DUSP6.1299	TTGTCTAGTACAGACAGCTGGT	TGCTGTTGACAGTGAGCGCCCAGCTGTCTGTACTAGACAATAGTGAAGCCACAGATGTATTGTCTAGTACAGACAGCTGGTTGCCTACTGCCTCGGA
**DUSP6**	DUSP6.1902	TTAGTGATAATAGTGTCCGTAA	TGCTGTTGACAGTGAGCGCTACGGACACTATTATCACTAATAGTGAAGCCACAGATGTATTAGTGATAATAGTGTCCGTAATGCCTACTGCCTCGGA
**DUSP6**	DUSP6.1330	TTAGTATTAACCAATTCCGCAC	TGCTGTTGACAGTGAGCGATGCGGAATTGGTTAATACTAATAGTGAAGCCACAGATGTATTAGTATTAACCAATTCCGCACTGCCTACTGCCTCGGA
**DUSP6**	DUSP6.1382	TATCTATACAGCATGTCCTGTT	TGCTGTTGACAGTGAGCGCACAGGACATGCTGTATAGATATAGTGAAGCCACAGATGTATATCTATACAGCATGTCCTGTTTGCCTACTGCCTCGGA
**DUSP6**	DUSP6.2099	TATACTGTTTAGCACAGCTGAA	TGCTGTTGACAGTGAGCGCTCAGCTGTGCTAAACAGTATATAGTGAAGCCACAGATGTATATACTGTTTAGCACAGCTGAATGCCTACTGCCTCGGA
**ELK1**	ELK1.447	TTGTAGACGAACTTCTGGCCGC	TGCTGTTGACAGTGAGCGACGGCCAGAAGTTCGTCTACAATAGTGAAGCCACAGATGTATTGTAGACGAACTTCTGGCCGCTGCCTACTGCCTCGGA
**ELK1**	ELK1.1078	TAACTCTTCTACTCACATCCAA	TGCTGTTGACAGTGAGCGCTGGATGTGAGTAGAAGAGTTATAGTGAAGCCACAGATGTATAACTCTTCTACTCACATCCAATGCCTACTGCCTCGGA
**ELK1**	ELK1.408	TTCTTGTCATAGTAGTACCGCA	TGCTGTTGACAGTGAGCGCGCGGTACTACTATGACAAGAATAGTGAAGCCACAGATGTATTCTTGTCATAGTAGTACCGCATGCCTACTGCCTCGGA
**ELK1**	ELK1.1708	TTGCCTAGAATAGAGACAGGAC	TGCTGTTGACAGTGAGCGATCCTGTCTCTATTCTAGGCAATAGTGAAGCCACAGATGTATTGCCTAGAATAGAGACAGGACTGCCTACTGCCTCGGA
**ELK1**	ELK1.1488	TATAGGAAGAGATGACTCCCTC	TGCTGTTGACAGTGAGCGAAGGGAGTCATCTCTTCCTATATAGTGAAGCCACAGATGTATATAGGAAGAGATGACTCCCTCTGCCTACTGCCTCGGA
**MAPK1**	MAPK1.1316	TTTAAGATCTGTATCCTGGCTG	TGCTGTTGACAGTGAGCGAAGCCAGGATACAGATCTTAAATAGTGAAGCCACAGATGTATTTAAGATCTGTATCCTGGCTGTGCCTACTGCCTCGGA
**MAPK1**	MAPK1.1233	TCCATGTCGAACTTGAATGGTG	TGCTGTTGACAGTGAGCGAACCATTCAAGTTCGACATGGATAGTGAAGCCACAGATGTATCCATGTCGAACTTGAATGGTGTGCCTACTGCCTCGGA
**MAPK1**	MAPK1.1121	TGAATGTCAACATTTTGTCCAA	TGCTGTTGACAGTGAGCGCTGGACAAAATGTTGACATTCATAGTGAAGCCACAGATGTATGAATGTCAACATTTTGTCCAATGCCTACTGCCTCGGA
**MAPK1**	MAPK1.528	TTCATTTGCTCGATGGTTGGTG	TGCTGTTGACAGTGAGCGAACCAACCATCGAGCAAATGAATAGTGAAGCCACAGATGTATTCATTTGCTCGATGGTTGGTGTGCCTACTGCCTCGGA
**MAPK1**	MAPK1.537	TATACATCTTTCATTTGCTCGA	TGCTGTTGACAGTGAGCGCCGAGCAAATGAAAGATGTATATAGTGAAGCCACAGATGTATATACATCTTTCATTTGCTCGATGCCTACTGCCTCGGA
**MAPK3**	MAPK3.1741	TATATTTATATATTAGACGGGT	TGCTGTTGACAGTGAGCGCCCCGTCTAATATATAAATATATAGTGAAGCCACAGATGTATATATTTATATATTAGACGGGTTGCCTACTGCCTCGGA
**MAPK3**	MAPK3.1200	TTTTCTAACAGTCTGGCGGGAG	TGCTGTTGACAGTGAGCGATCCCGCCAGACTGTTAGAAAATAGTGAAGCCACAGATGTATTTTCTAACAGTCTGGCGGGAGTGCCTACTGCCTCGGA
**MAPK3**	MAPK3.912	TAAAGGTTAACATCCGGTCCAG	TGCTGTTGACAGTGAGCGATGGACCGGATGTTAACCTTTATAGTGAAGCCACAGATGTATAAAGGTTAACATCCGGTCCAGTGCCTACTGCCTCGGA
**MAPK3**	MAPK3.751	TTGGAGTTCAGCATGATCTCTG	TGCTGTTGACAGTGAGCGAAGAGATCATGCTGAACTCCAATAGTGAAGCCACAGATGTATTGGAGTTCAGCATGATCTCTGTGCCTACTGCCTCGGA
**MAPK3**	MAPK3.616	TTGATGAGCAGGTTGGAGGGCT	TGCTGTTGACAGTGAGCGCGCCCTCCAACCTGCTCATCAATAGTGAAGCCACAGATGTATTGATGAGCAGGTTGGAGGGCTTGCCTACTGCCTCGGA
**SRF**	SRF.2720	TTTGTTTTCACTTCGTCTCCTC	TGCTGTTGACAGTGAGCGAAGGAGACGAAGTGAAAACAAATAGTGAAGCCACAGATGTATTTGTTTTCACTTCGTCTCCTCTGCCTACTGCCTCGGA
**SRF**	SRF.901	TCCATCTTGATCTTCACGCGGC	TGCTGTTGACAGTGAGCGACCGCGTGAAGATCAAGATGGATAGTGAAGCCACAGATGTATCCATCTTGATCTTCACGCGGCTGCCTACTGCCTCGGA

Gene Symbol	shRNA Name	Guide	97mer
**SRF**	SRF.85	TAAAGAGATACAATGTTTCCTT	TGCTGTTGACAGTGAGCGCAGGAAACATTGTATCTCTTTATAGTGAAGCCACAGATGTATAAAGAGATACAATGTTTCCTTTGCCTACTGCCTCGGA
**SRF**	SRF.3603	TTCACCTAATCACAGAAGCCAG	TGCTGTTGACAGTGAGCGATGGCTTCTGTGATTAGGTGAATAGTGAAGCCACAGATGTATTCACCTAATCACAGAAGCCAGTGCCTACTGCCTCGGA
**SRF**	SRF.1868	TGAAACAGGGATCTGCACTGTC	TGCTGTTGACAGTGAGCGAACAGTGCAGATCCCTGTTTCATAGTGAAGCCACAGATGTATGAAACAGGGATCTGCACTGTCTGCCTACTGCCTCGGA
**AATF**	AATF.1732	TTTTGCTTCGTAACTTCTGGAT	TGCTGTTGACAGTGAGCGCTCCAGAAGTTACGAAGCAAAATAGTGAAGCCACAGATGTATTTTGCTTCGTAACTTCTGGATTGCCTACTGCCTCGGA
**AATF**	AATF.1150	TAGATATCTAGTGTCTGGGTAC	TGCTGTTGACAGTGAGCGATACCCAGACACTAGATATCTATAGTGAAGCCACAGATGTATAGATATCTAGTGTCTGGGTACTGCCTACTGCCTCGGA
**AATF**	AATF.968	TTTTGTAGTTTGATCCTTCCTT	TGCTGTTGACAGTGAGCGCAGGAAGGATCAAACTACAAAATAGTGAAGCCACAGATGTATTTTGTAGTTTGATCCTTCCTTTGCCTACTGCCTCGGA
**AATF**	AATF.1152	TACTAGATATCTAGTGTCTGGG	TGCTGTTGACAGTGAGCGACCAGACACTAGATATCTAGTATAGTGAAGCCACAGATGTATACTAGATATCTAGTGTCTGGGTGCCTACTGCCTCGGA
**AATF**	AATF.1223	TTCTTCTCTTCTACCAGCTCAT	TGCTGTTGACAGTGAGCGCTGAGCTGGTAGAAGAGAAGAATAGTGAAGCCACAGATGTATTCTTCTCTTCTACCAGCTCATTGCCTACTGCCTCGGA
**ANKRD57**	ANKRD57.2246	TTGCATTTCAACTTTGATCTGA	TGCTGTTGACAGTGAGCGCCAGATCAAAGTTGAAATGCAATAGTGAAGCCACAGATGTATTGCATTTCAACTTTGATCTGATGCCTACTGCCTCGGA
**ANKRD57**	ANKRD57.2974	TTAAATATAGAAATAGTCCCAT	TGCTGTTGACAGTGAGCGCTGGGACTATTTCTATATTTAATAGTGAAGCCACAGATGTATTAAATATAGAAATAGTCCCATTGCCTACTGCCTCGGA
**ANKRD57**	ANKRD57.3667	TTTTGAGACAGAGTCTCCGTTG	TGCTGTTGACAGTGAGCGAAACGGAGACTCTGTCTCAAAATAGTGAAGCCACAGATGTATTTTGAGACAGAGTCTCCGTTGTGCCTACTGCCTCGGA
**AREG**	AREG.517	TTAACTACCTGTTCAACTCTGA	TGCTGTTGACAGTGAGCGCCAGAGTTGAACAGGTAGTTAATAGTGAAGCCACAGATGTATTAACTACCTGTTCAACTCTGATGCCTACTGCCTCGGA
**AREG**	AREG.677	TATATATTTGCATTCTCCGTGA	TGCTGTTGACAGTGAGCGCCACGGAGAATGCAAATATATATAGTGAAGCCACAGATGTATATATATTTGCATTCTCCGTGATGCCTACTGCCTCGGA
**AREG**	AREG.868	TTGTCTTCTAAGCTGGACTGTA	TGCTGTTGACAGTGAGCGCACAGTCCAGCTTAGAAGACAATAGTGAAGCCACAGATGTATTGTCTTCTAAGCTGGACTGTATGCCTACTGCCTCGGA
**AREG**	AREG.850	TGTAATAACAGCAACAGCTGTG	TGCTGTTGACAGTGAGCGAACAGCTGTTGCTGTTATTACATAGTGAAGCCACAGATGTATGTAATAACAGCAACAGCTGTGTGCCTACTGCCTCGGA
**AREG**	AREG.460	TTCGTTATCATACTCTTCTGAG	TGCTGTTGACAGTGAGCGATCAGAAGAGTATGATAACGAATAGTGAAGCCACAGATGTATTCGTTATCATACTCTTCTGAGTGCCTACTGCCTCGGA
**ARNTL2**	ARNTL2.1018	TTGTCTTTCTTACTGTTCCTTT	TGCTGTTGACAGTGAGCGCAAGGAACAGTAAGAAAGACAATAGTGAAGCCACAGATGTATTGTCTTTCTTACTGTTCCTTTTGCCTACTGCCTCGGA
**ARNTL2**	ARNTL2.674	TTGTCCAGTCAAACTAGCCTGA	TGCTGTTGACAGTGAGCGCCAGGCTAGTTTGACTGGACAATAGTGAAGCCACAGATGTATTGTCCAGTCAAACTAGCCTGATGCCTACTGCCTCGGA
**ARNTL2**	ARNTL2.111	TAGCTGTTGGTCTTGTCCCTGG	TGCTGTTGACAGTGAGCGACAGGGACAAGACCAACAGCTATAGTGAAGCCACAGATGTATAGCTGTTGGTCTTGTCCCTGGTGCCTACTGCCTCGGA
**ARNTL2**	ARNTL2.2192	TATGTAAGTAGTATTCTTGGTT	TGCTGTTGACAGTGAGCGCACCAAGAATACTACTTACATATAGTGAAGCCACAGATGTATATGTAAGTAGTATTCTTGGTTTGCCTACTGCCTCGGA
**ARNTL2**	ARNTL2.3342	TTTTGTTTTATTACCTTTGGTT	TGCTGTTGACAGTGAGCGCACCAAAGGTAATAAAACAAAATAGTGAAGCCACAGATGTATTTTGTTTTATTACCTTTGGTTTGCCTACTGCCTCGGA
**ATAD2**	ATAD2.1342	TTGCATTGGATCAACATCGGCA	TGCTGTTGACAGTGAGCGCGCCGATGTTGATCCAATGCAATAGTGAAGCCACAGATGTATTGCATTGGATCAACATCGGCATGCCTACTGCCTCGGA
**ATAD2**	ATAD2.569	TAATCCTACAACTTCGACGCAC	TGCTGTTGACAGTGAGCGATGCGTCGAAGTTGTAGGATTATAGTGAAGCCACAGATGTATAATCCTACAACTTCGACGCACTGCCTACTGCCTCGGA
**ATAD2**	ATAD2.1599	TACTTAGACAATCAGCACCTTT	TGCTGTTGACAGTGAGCGCAAGGTGCTGATTGTCTAAGTATAGTGAAGCCACAGATGTATACTTAGACAATCAGCACCTTTTGCCTACTGCCTCGGA
**ATAD2**	ATAD2.4380	TACTATTTTAATCTTTTCCTTA	TGCTGTTGACAGTGAGCGCAAGGAAAAGATTAAAATAGTATAGTGAAGCCACAGATGTATACTATTTTAATCTTTTCCTTATGCCTACTGCCTCGGA
**ATAD2**	ATAD2.3499	TTTATCACCAACAAGAGTGGAA	TGCTGTTGACAGTGAGCGCTCCACTCTTGTTGGTGATAAATAGTGAAGCCACAGATGTATTTATCACCAACAAGAGTGGAATGCCTACTGCCTCGGA
**BARD1**	BARD1.548	TTTAATTGAATTCTTCTTGTTT	TGCTGTTGACAGTGAGCGCAACAAGAAGAATTCAATTAAATAGTGAAGCCACAGATGTATTTAATTGAATTCTTCTTGTTTTGCCTACTGCCTCGGA

Gene Symbol	shRNA Name	Guide	97mer
**BARD1**	BARD1.1690	TATATTAACAGCATTTCTGGAG	TGCTGTTGACAGTGAGCGATCCAGAAATGCTGTTAATATATAGTGAAGCCACAGATGTATATATTAACAGCATTTCTGGAGTGCCTACTGCCTCGGA
**BARD1**	BARD1.543	TTGAATTCTTCTTGTTTCCTGC	TGCTGTTGACAGTGAGCGACAGGAAACAAGAAGAATTCAATAGTGAAGCCACAGATGTATTGAATTCTTCTTGTTTCCTGCTGCCTACTGCCTCGGA
**BARD1**	BARD1.582	TATCTGACTTTCTTACTTCGAG	TGCTGTTGACAGTGAGCGATCGAAGTAAGAAAGTCAGATATAGTGAAGCCACAGATGTATATCTGACTTTCTTACTTCGAGTGCCTACTGCCTCGGA
**BARD1**	BARD1.1647	TTGACTATATCCACATGCCCAT	TGCTGTTGACAGTGAGCGCTGGGCATGTGGATATAGTCAATAGTGAAGCCACAGATGTATTGACTATATCCACATGCCCATTGCCTACTGCCTCGGA
**BAZ1A**	BAZ1A.1434	TTTATTAGCAACATTGTCCTCT	TGCTGTTGACAGTGAGCGCGAGGACAATGTTGCTAATAAATAGTGAAGCCACAGATGTATTTATTAGCAACATTGTCCTCTTGCCTACTGCCTCGGA
**BAZ1A**	BAZ1A.5651	TAAACTTGGACTTAATACCTGC	TGCTGTTGACAGTGAGCGACAGGTATTAAGTCCAAGTTTATAGTGAAGCCACAGATGTATAAACTTGGACTTAATACCTGCTGCCTACTGCCTCGGA
**BAZ1A**	BAZ1A.4264	TAACTTGTGGTCTTCCTCGTTT	TGCTGTTGACAGTGAGCGCAACGAGGAAGACCACAAGTTATAGTGAAGCCACAGATGTATAACTTGTGGTCTTCCTCGTTTTGCCTACTGCCTCGGA
**BAZ1A**	BAZ1A.5297	TATCTTTCCTACATTCTCCTGA	TGCTGTTGACAGTGAGCGCCAGGAGAATGTAGGAAAGATATAGTGAAGCCACAGATGTATATCTTTCCTACATTCTCCTGATGCCTACTGCCTCGGA
**BAZ1A**	BAZ1A.4218	TTGAGAGTCATCTTCATCTTGT	TGCTGTTGACAGTGAGCGCCAAGATGAAGATGACTCTCAATAGTGAAGCCACAGATGTATTGAGAGTCATCTTCATCTTGTTGCCTACTGCCTCGGA
**BAZ1B**	BAZ1B.5150	TAAACATTTAAATTCTTCCTGC	TGCTGTTGACAGTGAGCGACAGGAAGAATTTAAATGTTTATAGTGAAGCCACAGATGTATAAACATTTAAATTCTTCCTGCTGCCTACTGCCTCGGA
**BAZ1B**	BAZ1B.3381	TTGACTTTCTCTTATTCCCTGA	TGCTGTTGACAGTGAGCGCCAGGGAATAAGAGAAAGTCAATAGTGAAGCCACAGATGTATTGACTTTCTCTTATTCCCTGATGCCTACTGCCTCGGA
**BAZ1B**	BAZ1B.2041	TTTCTTTCTCTCTTCGTTCTTT	TGCTGTTGACAGTGAGCGCAAGAACGAAGAGAGAAAGAAATAGTGAAGCCACAGATGTATTTCTTTCTCTCTTCGTTCTTTTGCCTACTGCCTCGGA
**BAZ1B**	BAZ1B.2230	TAATAGGATACTGAGCATCTGG	TGCTGTTGACAGTGAGCGACAGATGCTCAGTATCCTATTATAGTGAAGCCACAGATGTATAATAGGATACTGAGCATCTGGTGCCTACTGCCTCGGA
**BAZ1B**	BAZ1B.3695	TTTTCTTCTCTTTTGCTTGGGA	TGCTGTTGACAGTGAGCGCCCAAGCAAAAGAGAAGAAAATAGTGAAGCCACAGATGTATTTTCTTCTCTTTTGCTTGGGATGCCTACTGCCTCGGA
**BRCA1**	BRCA1.656	TTCAGTATTTGTTACATCCGTC	TGCTGTTGACAGTGAGCGAACGGATGTAACAAATACTGAATAGTGAAGCCACAGATGTATTCAGTATTTGTTACATCCGTCTGCCTACTGCCTCGGA
**BRCA1**	BRCA1.2561	TAAACTTAGGGAAACCAGCTAT	TGCTGTTGACAGTGAGCGCTAGCTGGTTTCCCTAAGTTTATAGTGAAGCCACAGATGTATAAACTTAGGGAAACCAGCTATTGCCTACTGCCTCGGA
**BRCA1**	BRCA1.1108	TTTTACTGGTAGAACTATCTGC	TGCTGTTGACAGTGAGCGACAGATAGTTCTACCAGTAAAATAGTGAAGCCACAGATGTATTTTACTGGTAGAACTATCTGCTGCCTACTGCCTCGGA
**BRCA1**	BRCA1.37	TTTTGTACTTCTTCAACGCGAA	TGCTGTTGACAGTGAGCGCTCGCGTTGAAGAAGTACAAAATAGTGAAGCCACAGATGTATTTTGTACTTCTTCAACGCGAATGCCTACTGCCTCGGA
**BRCA1**	BRCA1.190	TAAATCTCGTACTTTCTTGTAG	TGCTGTTGACAGTGAGCGATACAAGAAAGTACGAGATTTATAGTGAAGCCACAGATGTATAAATCTCGTACTTTCTTGTAGTGCCTACTGCCTCGGA
**CBFB**	CBFB.3057	TAAACAAACAAACACACAGTAT	TGCTGTTGACAGTGAGCGCTACTGTGTGTTTGTTTGTTTATAGTGAAGCCACAGATGTATAAACAAACAAACACACAGTATTGCCTACTGCCTCGGA
**CBFB**	CBFB.2451	TAACAATTTAAACACACTCCTT	TGCTGTTGACAGTGAGCGCAGGAGTGTGTTTAAATTGTTATAGTGAAGCCACAGATGTATAACAATTTAAACACACTCCTTTGCCTACTGCCTCGGA
**CBFB**	CBFB.507	TCTAAGTCGACATACTCTCGGC	TGCTGTTGACAGTGAGCGACCGAGAGTATGTCGACTTAGATAGTGAAGCCACAGATGTATCTAAGTCGACATACTCTCGGCTGCCTACTGCCTCGGA
**CBFB**	CBFB.665	TTGTGCTAATGCATCCTCCTGC	TGCTGTTGACAGTGAGCGACAGGAGGATGCATTAGCACAATAGTGAAGCCACAGATGTATTGTGCTAATGCATCCTCCTGCTGCCTACTGCCTCGGA
**CBFB**	CBFB.2457	AACAATTTAAACACACTCCTTT	TGCTGTTGACAGTGAGCGCAAGGAGTGTGTTTAAATTGTTTAGTGAAGCCACAGATGTAAACAATTTAAACACACTCCTTTTGCCTACTGCCTCGGA
**CBX3**	CBX3.1941	TTTAATGAGACAATTGACCCTA	TGCTGTTGACAGTGAGCGCAGGGTCAATTGTCTCATTAAATAGTGAAGCCACAGATGTATTTAATGAGACAATTGACCCTATGCCTACTGCCTCGGA
**CBX3**	CBX3.560	TTATTGAGCTTCATCTTCTGGA	TGCTGTTGACAGTGAGCGCCCAGAAGATGAAGCTCAATAATAGTGAAGCCACAGATGTATTATTGAGCTTCATCTTCTGGATGCCTACTGCCTCGGA
**CBX3**	CBX3.1278	TTGTCTAGTTTCCTCATCTGGA	TGCTGTTGACAGTGAGCGCCCAGATGAGGAAACTAGACAATAGTGAAGCCACAGATGTATTGTCTAGTTTCCTCATCTGGATGCCTACTGCCTCGGA

Gene Symbol	shRNA Name	Guide	97mer
**CBX3**	CBX3.1081	TTATCTCTTATTTTGCTTGGAA	TGCTGTTGACAGTGAGCGCTCCAAGCAAAATAAGAGATAATAGTGAAGCCACAGATGTATTATCTCTTATTTTGCTTGGAATGCCTACTGCCTCGGA
**CBX3**	CBX3.628	TAAATCAAAATCTAAGACCCAA	TGCTGTTGACAGTGAGCGCTGGGTCTTAGATTTTGATTTATAGTGAAGCCACAGATGTATAAATCAAAATCTAAGACCCAATGCCTACTGCCTCGGA
**CHAF1A**	CHAF1A.1387	TTCTCTTCTTCTCTTAACCGTT	TGCTGTTGACAGTGAGCGCACGGTTAAGAGAAGAAGAGAATAGTGAAGCCACAGATGTATTCTCTTCTTCTCTTAACCGTTTGCCTACTGCCTCGGA
**CHAF1A**	CHAF1A.3082	TAGAACTCTGCACACTTTGGGG	TGCTGTTGACAGTGAGCGACCCAAAGTGTGCAGAGTTCTATAGTGAAGCCACAGATGTATAGAACTCTGCACACTTTGGGGTGCCTACTGCCTCGGA
**CHAF1A**	CHAF1A.182	TTGTATTAACTTCTTAACTGGA	TGCTGTTGACAGTGAGCGCCCAGTTAAGAAGTTAATACAATAGTGAAGCCACAGATGTATTGTATTAACTTCTTAACTGGATGCCTACTGCCTCGGA
**CHAF1A**	CHAF1A.1374	TAACCGTTTCTCTTCTTCCTTT	TGCTGTTGACAGTGAGCGCAAGGAAGAAGAGAAACGGTTATAGTGAAGCCACAGATGTATAACCGTTTCTCTTCTTCCTTTTGCCTACTGCCTCGGA
**CHAF1A**	CHAF1A.3031	TTACACAGGAATTGAGTCGGTT	TGCTGTTGACAGTGAGCGCACCGACTCAATTCCTGTGTAATAGTGAAGCCACAGATGTATTACACAGGAATTGAGTCGGTTTGCCTACTGCCTCGGA
**CTNNBL1**	CTNNBL1.388	TTGTCTGGAAACTTAATCCGCA	TGCTGTTGACAGTGAGCGCGCGGATTAAGTTTCCAGACAATAGTGAAGCCACAGATGTATTGTCTGGAAACTTAATCCGCATGCCTACTGCCTCGGA
**CTNNBL1**	CTNNBL1.961	TTAAACACGGATAACTGCTGAA	TGCTGTTGACAGTGAGCGCTCAGCAGTTATCCGTGTTTAATAGTGAAGCCACAGATGTATTAAACACGGATAACTGCTGAATGCCTACTGCCTCGGA
**CTNNBL1**	CTNNBL1.1828	TTGTGTAGAAACTGATCCTGGG	TGCTGTTGACAGTGAGCGACCAGGATCAGTTTCTACACAATAGTGAAGCCACAGATGTATTGTGTAGAAACTGATCCTGGGTGCCTACTGCCTCGGA
**CTNNBL1**	CTNNBL1.1653	TTTTGATGGAGCTTCCTCGCAT	TGCTGTTGACAGTGAGCGCTGCGAGGAAGCTCCATCAAAATAGTGAAGCCACAGATGTATTTTGATGGAGCTTCCTCGCATTGCCTACTGCCTCGGA
**CTNNBL1**	CTNNBL1.1495	TTGTCGATGATCTCTCCTCGCC	TGCTGTTGACAGTGAGCGAGCGAGGAGAGATCATCGACAATAGTGAAGCCACAGATGTATTGTCGATGATCTCTCCTCGCCTGCCTACTGCCTCGGA
**DAB2IP**	DAB2IP.2670	TCGTCTTCTAACAACTGCGCGT	TGCTGTTGACAGTGAGCGCCGCGCAGTTGTTAGAAGACGATAGTGAAGCCACAGATGTATCGTCTTCTAACAACTGCGCGTTGCCTACTGCCTCGGA
**DAB2IP**	DAB2IP.468	TTCTTCTTGTCGGTCTCCCGGT	TGCTGTTGACAGTGAGCGCCCGGGAGACCGACAAGAAGAATAGTGAAGCCACAGATGTATTCTTCTTGTCGGTCTCCCGGTTGCCTACTGCCTCGGA
**DAB2IP**	DAB2IP.2316	TTTTCAGTCAGTGACATCTGTC	TGCTGTTGACAGTGAGCGAACAGATGTCACTGACTGAAAATAGTGAAGCCACAGATGTATTTTCAGTCAGTGACATCTGTCTGCCTACTGCCTCGGA
**DAB2IP**	DAB2IP.411	TTGTGGAACTCGAAGTGCTCGC	TGCTGTTGACAGTGAGCGACGAGCACTTCGAGTTCCACAATAGTGAAGCCACAGATGTATTGTGGAACTCGAAGTGCTCGCTGCCTACTGCCTCGGA
**DAB2IP**	DAB2IP.957	TACAGCGCTTTGATGAACTCAC	TGCTGTTGACAGTGAGCGATGAGTTCATCAAAGCGCTGTATAGTGAAGCCACAGATGTATACAGCGCTTTGATGAACTCACTGCCTACTGCCTCGGA
**DDX54**	DDX54.301	TTCTTCTTCTTCTTGTTCTGGG	TGCTGTTGACAGTGAGCGACCAGAACAAGAAGAAGAAGAATAGTGAAGCCACAGATGTATTCTTCTTCTTCTTGTTCTGGGTGCCTACTGCCTCGGA
**DDX54**	DDX54.421	TTGCCATCCAAGATCACCGGGA	TGCTGTTGACAGTGAGCGCCCCGGTGATCTTGGATGGCAATAGTGAAGCCACAGATGTATTGCCATCCAAGATCACCGGGATGCCTACTGCCTCGGA
**DDX54**	DDX54.2274	TAATCTTCTTCTTGTCTTCCTG	TGCTGTTGACAGTGAGCGAAGGAAGACAAGAAGAAGATTATAGTGAAGCCACAGATGTATAATCTTCTTCTTGTCTTCCTGTGCCTACTGCCTCGGA
**DDX54**	DDX54.1756	TTGATCTCAAAGATAGTCGCCC	TGCTGTTGACAGTGAGCGAGGCGACTATCTTTGAGATCAATAGTGAAGCCACAGATGTATTGATCTCAAAGATAGTCGCCCTGCCTACTGCCTCGGA
**DDX54**	DDX54.2269	TTCTTCTTGTCTTCCTGTCCTG	TGCTGTTGACAGTGAGCGAAGGACAGGAAGACAAGAAGAATAGTGAAGCCACAGATGTATTCTTCTTGTCTTCCTGTCCTGTGCCTACTGCCTCGGA
**DEK**	DEK.2225	TTAGTCATAATCGTGAAGCTGG	TGCTGTTGACAGTGAGCGACAGCTTCACGATTATGACTAATAGTGAAGCCACAGATGTATTAGTCATAATCGTGAAGCTGGTGCCTACTGCCTCGGA
**DEK**	DEK.398	TAGATTTCTAAGTTCATCGGTT	TGCTGTTGACAGTGAGCGCACCGATGAACTTAGAAATCTATAGTGAAGCCACAGATGTATAGATTTCTAAGTTCATCGGTTTGCCTACTGCCTCGGA
**DEK**	DEK.448	TTAATGAGGACACAGTGCCTGG	TGCTGTTGACAGTGAGCGACAGGCACTGTGTCCTCATTAATAGTGAAGCCACAGATGTATTAATGAGGACACAGTGCCTGGTGCCTACTGCCTCGGA
**DEK**	DEK.508	TTTTATATTGGACACTTCCTTT	TGCTGTTGACAGTGAGCGCAAGGAAGTGTCCAATATAAAATAGTGAAGCCACAGATGTATTTTATATTGGACACTTCCTTTTGCCTACTGCCTCGGA
**DEK**	DEK.829	TTTCTTTATCTTCATCATCTGA	TGCTGTTGACAGTGAGCGCCAGATGATGAAGATAAAGAAATAGTGAAGCCACAGATGTATTTCTTTATCTTCATCATCTGATGCCTACTGCCTCGGA

Gene Symbol	shRNA Name	Guide	97mer
**DNAJC2**	DNAJC2.1165	TTCTTTAGCTTCTTGCTCCTTC	TGCTGTTGACAGTGAGCGAAAGGAGCAAGAAGCTAAAGAATAGTGAAGCCACAGATGTATTCTTTAGCTTCTTGCTCCTTCTGCCTACTGCCTCGGA
**DNAJC2**	DNAJC2.1455	TAATTGTAGATCATCTTCTGAC	TGCTGTTGACAGTGAGCGATCAGAAGATGATCTACAATTATAGTGAAGCCACAGATGTATAATTGTAGATCATCTTCTGACTGCCTACTGCCTCGGA
**DNAJC2**	DNAJC2.1157	TTAGCTTCTTGCTCCTTCCGTT	TGCTGTTGACAGTGAGCGCACGGAAGGAGCAAGAAGCTAATAGTGAAGCCACAGATGTATTAGCTTCTTGCTCCTTCCGTTTGCCTACTGCCTCGGA
**DNAJC2**	DNAJC2.429	TTCATCTTCTGATTCCTCGGAT	TGCTGTTGACAGTGAGCGCTCCGAGGAATCAGAAGATGAATAGTGAAGCCACAGATGTATTCATCTTCTGATTCCTCGGATTGCCTACTGCCTCGGA
**DNAJC2**	DNAJC2.1466	TTAGTAATTGTAGATCATCTTC	TGCTGTTGACAGTGAGCGAAAGATGATCTACAATTACTAATAGTGAAGCCACAGATGTATTAGTAATTGTAGATCATCTTCTGCCTACTGCCTCGGA
**DNMT1**	DNMT1.884	TTCATCTCTTTCTTCTTCCTTT	TGCTGTTGACAGTGAGCGCAAGGAAGAAGAAAGAGATGAATAGTGAAGCCACAGATGTATTCATCTCTTTCTTCTTCCTTTTGCCTACTGCCTCGGA
**DNMT1**	DNMT1.3991	TTGAAGGAGACAAAGTTCCTGA	TGCTGTTGACAGTGAGCGCCAGGAACTTTGTCTCCTTCAATAGTGAAGCCACAGATGTATTGAAGGAGACAAAGTTCCTGATGCCTACTGCCTCGGA
**DNMT1**	DNMT1.3205	TTTGATGTCAGTCTCATTGGGC	TGCTGTTGACAGTGAGCGACCCAATGAGACTGACATCAAATAGTGAAGCCACAGATGTATTTGATGTCAGTCTCATTGGGCTGCCTACTGCCTCGGA
**DNMT1**	DNMT1.2410	TTATAGTAACTCTTCTTCCCAT	TGCTGTTGACAGTGAGCGCTGGGAAGAAGAGTTACTATAATAGTGAAGCCACAGATGTATTATAGTAACTCTTCTTCCCATTGCCTACTGCCTCGGA
**DNMT1**	DNMT1.2839	TTGAACTTGTTGTCCTCTGTTG	TGCTGTTGACAGTGAGCGAAACAGAGGACAACAAGTTCAATAGTGAAGCCACAGATGTATTGAACTTGTTGTCCTCTGTTGTGCCTACTGCCTCGGA
**DR1**	DR1.1454	ATTGATATTATACTGAACCTTA	TGCTGTTGACAGTGAGCGCAAGGTTCAGTATAATATCAATTAGTGAAGCCACAGATGTAATTGATATTATACTGAACCTTATGCCTACTGCCTCGGA
**DR1**	DR1.1779	TAAACAAGTATATTGATCTGAG	TGCTGTTGACAGTGAGCGATCAGATCAATATACTTGTTTATAGTGAAGCCACAGATGTATAAACAAGTATATTGATCTGAGTGCCTACTGCCTCGGA
**DR1**	DR1.2938	TTAACTTATCGTTCTAGACTAG	TGCTGTTGACAGTGAGCGATAGTCTAGAACGATAAGTTAATAGTGAAGCCACAGATGTATTAACTTATCGTTCTAGACTAGTGCCTACTGCCTCGGA
**DR1**	DR1.2574	TATCTTAAGTGACTTTTCCTTT	TGCTGTTGACAGTGAGCGCAAGGAAAAGTCACTTAAGATATAGTGAAGCCACAGATGTATATCTTAAGTGACTTTTCCTTTTGCCTACTGCCTCGGA
**DR1**	DR1.766	TTTATTGATAGCAGCTCTGGGG	TGCTGTTGACAGTGAGCGACCCAGAGCTGCTATCAATAAATAGTGAAGCCACAGATGTATTTATTGATAGCAGCTCTGGGGTGCCTACTGCCTCGGA
**EGR1**	EGR1.2823	TTTTGTTTTCTTACATTCTGGA	TGCTGTTGACAGTGAGCGCCCAGAATGTAAGAAAACAAAATAGTGAAGCCACAGATGTATTTTGTTTTCTTACATTCTGGATGCCTACTGCCTCGGA
**EGR1**	EGR1.2815	TCTTACATTCTGGAGAACCGAA	TGCTGTTGACAGTGAGCGCTCGGTTCTCCAGAATGTAAGATAGTGAAGCCACAGATGTATCTTACATTCTGGAGAACCGAATGCCTACTGCCTCGGA
**EGR1**	EGR1.2822	TTTGTTTTCTTACATTCTGGAG	TGCTGTTGACAGTGAGCGATCCAGAATGTAAGAAAACAAATAGTGAAGCCACAGATGTATTTGTTTTCTTACATTCTGGAGTGCCTACTGCCTCGGA
**EGR1**	EGR1.2946	TAACATACAAAAATCGCCGCCT	TGCTGTTGACAGTGAGCGCGGCGGCGATTTTTGTATGTTATAGTGAAGCCACAGATGTATAACATACAAAAATCGCCGCCTTGCCTACTGCCTCGGA
**EGR1**	EGR1.2525	TAACGGAACAACACTCTGACAC	TGCTGTTGACAGTGAGCGATGTCAGAGTGTTGTTCCGTTATAGTGAAGCCACAGATGTATAACGGAACAACACTCTGACACTGCCTACTGCCTCGGA
**ENO1**	ENO1.261	TTGATGACATTGAACGCCGGGA	TGCTGTTGACAGTGAGCGCCCCGGCGTTCAATGTCATCAATAGTGAAGCCACAGATGTATTGATGACATTGAACGCCGGGATGCCTACTGCCTCGGA
**ENO1**	ENO1.1440	TTTGAGCACAAAACCACCGGGG	TGCTGTTGACAGTGAGCGACCCGGTGGTTTTGTGCTCAAATAGTGAAGCCACAGATGTATTTGAGCACAAAACCACCGGGGTGCCTACTGCCTCGGA
**ENO1**	ENO1.1437	TTTTGAGCACAAAACCACCGGG	TGCTGTTGACAGTGAGCGACCGGTGGTTTTGTGCTCAAAATAGTGAAGCCACAGATGTATTTTGAGCACAAAACCACCGGGTGCCTACTGCCTCGGA
**ENO1**	ENO1.1441	TTTATTTTGAGCACAAAACCAC	TGCTGTTGACAGTGAGCGATGGTTTTGTGCTCAAAATAAATAGTGAAGCCACAGATGTATTTATTTTGAGCACAAAACCACTGCCTACTGCCTCGGA
**ENO1**	ENO1.681	TCTTCGATAGACACCACTGGGT	TGCTGTTGACAGTGAGCGCCCAGTGGTGTCTATCGAAGATAGTGAAGCCACAGATGTATCTTCGATAGACACCACTGGGTTGCCTACTGCCTCGGA
**EREG**	EREG.1324	TAGATGAGTGACTAGTACCTGT	TGCTGTTGACAGTGAGCGCCAGGTACTAGTCACTCATCTATAGTGAAGCCACAGATGTATAGATGAGTGACTAGTACCTGTTGCCTACTGCCTCGGA
**EREG**	EREG.1188	TAGTGTTTAACACAGGACCTAT	TGCTGTTGACAGTGAGCGCTAGGTCCTGTGTTAAACACTATAGTGAAGCCACAGATGTATAGTGTTTAACACAGGACCTATTGCCTACTGCCTCGGA

Gene Symbol	shRNA Name	Guide	97mer
**EREG**	EREG.3002	TAACCTATTCACATCCTCCTGT	TGCTGTTGACAGTGAGCGCCAGGAGGATGTGAATAGGTTATAGTGAAGCCACAGATGTATAACCTATTCACATCCTCCTGTTGCCTACTGCCTCGGA
**EREG**	EREG.3718	TTATAGAACTTAATATTCCTGG	TGCTGTTGACAGTGAGCGACAGGAATATTAAGTTCTATAATAGTGAAGCCACAGATGTATTATAGAACTTAATATTCCTGGTGCCTACTGCCTCGGA
**EREG**	EREG.993	TTGCTAACAATTCTTGAGCTAT	TGCTGTTGACAGTGAGCGCTAGCTCAAGAATTGTTAGCAATAGTGAAGCCACAGATGTATTGCTAACAATTCTTGAGCTATTGCCTACTGCCTCGGA
**ESPL1**	ESPL1.267	TAGCTTAGCAGTCAGCTGCTGG	TGCTGTTGACAGTGAGCGACAGCAGCTGACTGCTAAGCTATAGTGAAGCCACAGATGTATAGCTTAGCAGTCAGCTGCTGGTGCCTACTGCCTCGGA
**ESPL1**	ESPL1.1362	TACAACTGTCCACTAGTTGGGT	TGCTGTTGACAGTGAGCGCCCCAACTAGTGGACAGTTGTATAGTGAAGCCACAGATGTATACAACTGTCCACTAGTTGGGTTGCCTACTGCCTCGGA
**ESPL1**	ESPL1.1897	TCACAGATGATGTTGAAGCGTT	TGCTGTTGACAGTGAGCGCACGCTTCAACATCATCTGTGATAGTGAAGCCACAGATGTATCACAGATGATGTTGAAGCGTTTGCCTACTGCCTCGGA
**ESPL1**	ESPL1.1771	TTTAGCTGTAGCTCCTTGTCTC	TGCTGTTGACAGTGAGCGAAGACAAGGAGCTACAGCTAAATAGTGAAGCCACAGATGTATTTAGCTGTAGCTCCTTGTCTCTGCCTACTGCCTCGGA
**ESPL1**	ESPL1.2669	TTGACTTCGAAGCAGATCACAG	TGCTGTTGACAGTGAGCGATGTGATCTGCTTCGAAGTCAATAGTGAAGCCACAGATGTATTGACTTCGAAGCAGATCACAGTGCCTACTGCCTCGGA
**ETV4**	ETV4.686	TTCATTTATATGTACACAGGGC	TGCTGTTGACAGTGAGCGACCCTGTGTACATATAAATGAATAGTGAAGCCACAGATGTATTCATTTATATGTACACAGGGCTGCCTACTGCCTCGGA
**ETV4**	ETV4.380	TAATAGTATCGGAGCGAGCGGC	TGCTGTTGACAGTGAGCGACCGCTCGCTCCGATACTATTATAGTGAAGCCACAGATGTATAATAGTATCGGAGCGAGCGGCTGCCTACTGCCTCGGA
**ETV4**	ETV4.806	TTTCCTTCCCAATGACTCCGGT	TGCTGTTGACAGTGAGCGCCCGGAGTCATTGGGAAGGAAATAGTGAAGCCACAGATGTATTTCCTTCCCAATGACTCCGGTTGCCTACTGCCTCGGA
**ETV4**	ETV4.385	TCTCATAATAGTATCGGAGCGA	TGCTGTTGACAGTGAGCGCCGCTCCGATACTATTATGAGATAGTGAAGCCACAGATGTATCTCATAATAGTATCGGAGCGATGCCTACTGCCTCGGA
**ETV4**	ETV4.816	TTTCTCCACTTTTCCTTCCCAA	TGCTGTTGACAGTGAGCGCTGGGAAGGAAAAGTGGAGAAATAGTGAAGCCACAGATGTATTTCTCCACTTTTCCTTCCCAATGCCTACTGCCTCGGA
**ETV5**	ETV5.390	TTGAAGTTGACTGAGATCCTGA	TGCTGTTGACAGTGAGCGCCAGGATCTCAGTCAACTTCAATAGTGAAGCCACAGATGTATTGAAGTTGACTGAGATCCTGATGCCTACTGCCTCGGA
**ETV5**	ETV5.3563	TTTGATTAGAGTACAATGCTAA	TGCTGTTGACAGTGAGCGCTAGCATTGTACTCTAATCAAATAGTGAAGCCACAGATGTATTTGATTAGAGTACAATGCTAATGCCTACTGCCTCGGA
**ETV5**	ETV5.2355	TATGATTTTGAGAACCACGGAG	TGCTGTTGACAGTGAGCGATCCGTGGTTCTCAAAATCATATAGTGAAGCCACAGATGTATATGATTTTGAGAACCACGGAGTGCCTACTGCCTCGGA
**ETV5**	ETV5.3085	TTACCTGTCAGTATCACACGTA	TGCTGTTGACAGTGAGCGCACGTGTGATACTGACAGGTAATAGTGAAGCCACAGATGTATTACCTGTCAGTATCACACGTATGCCTACTGCCTCGGA
**ETV5**	ETV5.2130	TAGTAGTCCATGATCGATGCAG	TGCTGTTGACAGTGAGCGATGCATCGATCATGGACTACTATAGTGAAGCCACAGATGTATAGTAGTCCATGATCGATGCAGTGCCTACTGCCTCGGA
**EZH2**	EZH2.292	TTCAATGAAAGTACCATCCTGA	TGCTGTTGACAGTGAGCGCCAGGATGGTACTTTCATTGAATAGTGAAGCCACAGATGTATTCAATGAAAGTACCATCCTGATGCCTACTGCCTCGGA
**EZH2**	EZH2.578	TTTCCTTTAGTTCTTCTGCTGT	TGCTGTTGACAGTGAGCGCCAGCAGAAGAACTAAAGGAAATAGTGAAGCCACAGATGTATTTCCTTTAGTTCTTCTGCTGTTGCCTACTGCCTCGGA
**EZH2**	EZH2.1100	TTTATTGGTGTTTGACACCGAG	TGCTGTTGACAGTGAGCGATCGGTGTCAAACACCAATAAATAGTGAAGCCACAGATGTATTTATTGGTGTTTGACACCGAGTGCCTACTGCCTCGGA
**EZH2**	EZH2.1112	TTTGGCTTCATCTTTATTGGTG	TGCTGTTGACAGTGAGCGAACCAATAAAGATGAAGCCAAATAGTGAAGCCACAGATGTATTTGGCTTCATCTTTATTGGTGTGCCTACTGCCTCGGA
**EZH2**	EZH2.1700	TATTTATCATACACTTTCCCTC	TGCTGTTGACAGTGAGCGAAGGGAAAGTGTATGATAAATATAGTGAAGCCACAGATGTATATTTATCATACACTTTCCCTCTGCCTACTGCCTCGGA
**FOS**	FOS.1690	TTAATTCCAATAATGAACCCAA	TGCTGTTGACAGTGAGCGCTGGGTTCATTATTGGAATTAATAGTGAAGCCACAGATGTATTAATTCCAATAATGAACCCAATGCCTACTGCCTCGGA
**FOS**	FOS.1804	TTTTCTTAGTATAATATTGGTC	TGCTGTTGACAGTGAGCGAACCAATATTATACTAAGAAAATAGTGAAGCCACAGATGTATTTTCTTAGTATAATATTGGTCTGCCTACTGCCTCGGA
**FOS**	FOS.1894	TAACATTACAATGAACATTGAT	TGCTGTTGACAGTGAGCGCTCAATGTTCATTGTAATGTTATAGTGAAGCCACAGATGTATAACATTACAATGAACATTGATTGCCTACTGCCTCGGA
**FOS**	FOS.2115	TTTTATTGACAATGTCTTGGAA	TGCTGTTGACAGTGAGCGCTCCAAGACATTGTCAATAAAATAGTGAAGCCACAGATGTATTTTATTGACAATGTCTTGGAATGCCTACTGCCTCGGA

Gene Symbol	shRNA Name	Guide	97mer
**FOS**	FOS.703	TCTAGTTGGTCTGTCTCCGCTT	TGCTGTTGACAGTGAGCGCAGCGGAGACAGACCAACTAGATAGTGAAGCCACAGATGTATCTAGTTGGTCTGTCTCCGCTTTGCCTACTGCCTCGGA
**FOSL1**	FOSL1.1280	TAAGGATCTACAAAGTCTCTGG	TGCTGTTGACAGTGAGCGACAGAGACTTTGTAGATCCTTATAGTGAAGCCACAGATGTATAAGGATCTACAAAGTCTCTGGTGCCTACTGCCTCGGA
**FOSL1**	FOSL1.90	TATGAATGAAAAGTTCTCGGGC	TGCTGTTGACAGTGAGCGACCCGAGAACTTTTCATTCATATAGTGAAGCCACAGATGTATATGAATGAAAAGTTCTCGGGCTGCCTACTGCCTCGGA
**FOSL1**	FOSL1.1634	TTTTATTCCATTTTGGTAGGTT	TGCTGTTGACAGTGAGCGCACCTACCAAAATGGAATAAAATAGTGAAGCCACAGATGTATTTTATTCCATTTTGGTAGGTTTGCCTACTGCCTCGGA
**FOSL1**	FOSL1.91	TTATGAATGAAAAGTTCTCGGG	TGCTGTTGACAGTGAGCGACCGAGAACTTTTCATTCATAATAGTGAAGCCACAGATGTATTATGAATGAAAAGTTCTCGGGTGCCTACTGCCTCGGA
**FOSL1**	FOSL1.1405	TGAGTTAGTGTTCTAGGTGGGT	TGCTGTTGACAGTGAGCGCCCCACCTAGAACACTAACTCATAGTGAAGCCACAGATGTATGAGTTAGTGTTCTAGGTGGGTTGCCTACTGCCTCGGA
**FOXM1**	FOXM1.1812	TTGAATCACAAGCATTTCCGAG	TGCTGTTGACAGTGAGCGATCGGAAATGCTTGTGATTCAATAGTGAAGCCACAGATGTATTGAATCACAAGCATTTCCGAGTGCCTACTGCCTCGGA
**FOXM1**	FOXM1.725	TCTAGGAAGATTCACATCCCTA	TGCTGTTGACAGTGAGCGCAGGGATGTGAATCTTCCTAGATAGTGAAGCCACAGATGTATCTAGGAAGATTCACATCCCTATGCCTACTGCCTCGGA
**FOXM1**	FOXM1.1277	TTGATGGTCATGTTCCGGCGGA	TGCTGTTGACAGTGAGCGCCCGCCGGAACATGACCATCAATAGTGAAGCCACAGATGTATTGATGGTCATGTTCCGGCGGATGCCTACTGCCTCGGA
**FOXM1**	FOXM1.2496	TAGCTCAGGAATAAACTGGGAC	TGCTGTTGACAGTGAGCGATCCCAGTTTATTCCTGAGCTATAGTGAAGCCACAGATGTATAGCTCAGGAATAAACTGGGACTGCCTACTGCCTCGGA
**FOXM1**	FOXM1.463	TGGTTAATAATCTTGATCCCAG	TGCTGTTGACAGTGAGCGATGGGATCAAGATTATTAACCATAGTGAAGCCACAGATGTATGGTTAATAATCTTGATCCCAGTGCCTACTGCCTCGGA
**FUBP1**	FUBP1.2714	TATAGCAGCAGTACAGGTCTGA	TGCTGTTGACAGTGAGCGCCAGACCTGTACTGCTGCTATATAGTGAAGCCACAGATGTATATAGCAGCAGTACAGGTCTGATGCCTACTGCCTCGGA
**FUBP1**	FUBP1.1846	TTTCTTGTAGTACTCTTCCCAA	TGCTGTTGACAGTGAGCGCTGGGAAGAGTACTACAAGAAATAGTGAAGCCACAGATGTATTTCTTGTAGTACTCTTCCCAATGCCTACTGCCTCGGA
**FUBP1**	FUBP1.521	TTAACATACAGGACCTTTCTGG	TGCTGTTGACAGTGAGCGACAGAAAGGTCCTGTATGTTAATAGTGAAGCCACAGATGTATTAACATACAGGACCTTTCTGGTGCCTACTGCCTCGGA
**FUBP1**	FUBP1.236	TTGAATTCAGTGATGTCCCTGC	TGCTGTTGACAGTGAGCGACAGGGACATCACTGAATTCAATAGTGAAGCCACAGATGTATTGAATTCAGTGATGTCCCTGCTGCCTACTGCCTCGGA
**FUBP1**	FUBP1.1932	TTGTCTATAATACTCAGCCCAG	TGCTGTTGACAGTGAGCGATGGGCTGAGTATTATAGACAATAGTGAAGCCACAGATGTATTGTCTATAATACTCAGCCCAGTGCCTACTGCCTCGGA
**FUS**	FUS.212	ATAACCACTGTAACTCTGCTGT	TGCTGTTGACAGTGAGCGCCAGCAGAGTTACAGTGGTTATTAGTGAAGCCACAGATGTAATAACCACTGTAACTCTGCTGTTGCCTACTGCCTCGGA
**FUS**	FUS.2807	TTGAGAGGAAAGCACTTCCCAA	TGCTGTTGACAGTGAGCGCTGGGAAGTGCTTTCCTCTCAATAGTGAAGCCACAGATGTATTGAGAGGAAAGCACTTCCCAATGCCTACTGCCTCGGA
**FUS**	FUS.960	TTGTTGTCTGAATTATCCTGTT	TGCTGTTGACAGTGAGCGCACAGGATAATTCAGACAACAATAGTGAAGCCACAGATGTATTGTTGTCTGAATTATCCTGTTTGCCTACTGCCTCGGA
**FUS**	FUS.1050	TTGTTTGTCTTAATAATACCAA	TGCTGTTGACAGTGAGCGCTGGTATTATTAAGACAAACAATAGTGAAGCCACAGATGTATTGTTTGTCTTAATAATACCAATGCCTACTGCCTCGGA
**FUS**	FUS.1028	TCTGCTTGAAGTAATCAGCCAC	TGCTGTTGACAGTGAGCGATGGCTGATTACTTCAAGCAGATAGTGAAGCCACAGATGTATCTGCTTGAAGTAATCAGCCACTGCCTACTGCCTCGGA
**HMGA1**	HMGA1.211	TTTTGCTTCCCTTTGGTCGGCC	TGCTGTTGACAGTGAGCGAGCCGACCAAAGGGAAGCAAAATAGTGAAGCCACAGATGTATTTTGCTTCCCTTTGGTCGGCCTGCCTACTGCCTCGGA
**HMGA1**	HMGA1.155	TCCTTGAATTCCTCGAGCGGAG	TGCTGTTGACAGTGAGCGATCCGCTCGAGGAATTCAAGGATAGTGAAGCCACAGATGTATCCTTGAATTCCTCGAGCGGAGTGCCTACTGCCTCGGA
**HMGA1**	HMGA1.40	TCATCTTCCCTTCTCTAAGGAG	TGCTGTTGACAGTGAGCGATCCTTAGAGAAGGGAAGATGATAGTGAAGCCACAGATGTATCATCTTCCCTTCTCTAAGGAGTGCCTACTGCCTCGGA
**HMGA1**	HMGA1.1061	TATGTACTCAGATCCCAGGCGG	TGCTGTTGACAGTGAGCGACGCCTGGGATCTGAGTACATATAGTGAAGCCACAGATGTATATGTACTCAGATCCCAGGCGGTGCCTACTGCCTCGGA
**HMGA1**	HMGA1.1391	TGAGGATGAACATTTGGCGCTG	TGCTGTTGACAGTGAGCGAAGCGCCAAATGTTCATCCTCATAGTGAAGCCACAGATGTATGAGGATGAACATTTGGCGCTGTGCCTACTGCCTCGGA
**HMGB1**	HMGB1.2309	TTAACTAGTATTTAAAACCTCT	TGCTGTTGACAGTGAGCGCGAGGTTTTAAATACTAGTTAATAGTGAAGCCACAGATGTATTAACTAGTATTTAAAACCTCTTGCCTACTGCCTCGGA

Gene Symbol	shRNA Name	Guide	97mer
**HMGB1**	HMGB1.2683	TACAGTAGAAACTTCCATCTAA	TGCTGTTGACAGTGAGCGCTAGATGGAAGTTTCTACTGTATAGTGAAGCCACAGATGTATACAGTAGAAACTTCCATCTAATGCCTACTGCCTCGGA
**HMGB1**	HMGB1.1726	TTGTATTTTAAGCTCACGCTTT	TGCTGTTGACAGTGAGCGCAAGCGTGAGCTTAAAATACAATAGTGAAGCCACAGATGTATTGTATTTTAAGCTCACGCTTTTGCCTACTGCCTCGGA
**HMGB1**	HMGB1.1534	TTGAGTAGATTGATTACTCTTC	TGCTGTTGACAGTGAGCGAAAGAGTAATCAATCTACTCAATAGTGAAGCCACAGATGTATTGAGTAGATTGATTACTCTTCTGCCTACTGCCTCGGA
**HMGB1**	HMGB1.2936	TTAGACATCCAACTTCTAGGGG	TGCTGTTGACAGTGAGCGACCCTAGAAGTTGGATGTCTAATAGTGAAGCCACAGATGTATTAGACATCCAACTTCTAGGGGTGCCTACTGCCTCGGA
**HMGB2**	HMGB2.635	TTATTCTTCATCTTCATCCTCT	TGCTGTTGACAGTGAGCGCGAGGATGAAGATGAAGAATAATAGTGAAGCCACAGATGTATTATTCTTCATCTTCATCCTCTTGCCTACTGCCTCGGA
**HMGB2**	HMGB2.1295	TTAGCTAATAAACAGAAACGTC	TGCTGTTGACAGTGAGCGAACGTTTCTGTTTATTAGCTAATAGTGAAGCCACAGATGTATTAGCTAATAAACAGAAACGTCTGCCTACTGCCTCGGA
**HMGB2**	HMGB2.102	TTCTTCTTGTGCTCTTCCCGGC	TGCTGTTGACAGTGAGCGACCGGGAAGAGCACAAGAAGAATAGTGAAGCCACAGATGTATTCTTCTTGTGCTCTTCCCGGCTGCCTACTGCCTCGGA
**HMGB2**	HMGB2.952	TTACTATTGATACTAATTCCTA	TGCTGTTGACAGTGAGCGCAGGAATTAGTATCAATAGTAATAGTGAAGCCACAGATGTATTACTATTGATACTAATTCCTATGCCTACTGCCTCGGA
**HMGB2**	HMGB2.849	TCTAACTGTATGAGTAGCCCAT	TGCTGTTGACAGTGAGCGCTGGGCTACTCATACAGTTAGATAGTGAAGCCACAGATGTATCTAACTGTATGAGTAGCCCATTGCCTACTGCCTCGGA
**HMGB3**	HMGB3.2264	TTAACATTGAACATCAATCTAC	TGCTGTTGACAGTGAGCGATAGATTGATGTTCAATGTTAATAGTGAAGCCACAGATGTATTAACATTGAACATCAATCTACTGCCTACTGCCTCGGA
**HMGB3**	HMGB3.1909	TTTGACACACCATACACTCTGA	TGCTGTTGACAGTGAGCGCCAGAGTGTATGGTGTGTCAAATAGTGAAGCCACAGATGTATTTGACACACCATACACTCTGATGCCTACTGCCTCGGA
**HMGB3**	HMGB3.628	TTCATCTTCCTCTTCCACCTTT	TGCTGTTGACAGTGAGCGCAAGGTGGAAGAGGAAGATGAATAGTGAAGCCACAGATGTATTCATCTTCCTCTTCCACCTTTTGCCTACTGCCTCGGA
**HMGB3**	HMGB3.1249	TACAGAAACAAGACAACCTGAA	TGCTGTTGACAGTGAGCGCTCAGGTTGTCTTGTTTCTGTATAGTGAAGCCACAGATGTATACAGAAACAAGACAACCTGAATGCCTACTGCCTCGGA
**HMGB3**	HMGB3.2531	TAATAGCACAAAAACACTCCTG	TGCTGTTGACAGTGAGCGAAGGAGTGTTTTTGTGCTATTATAGTGAAGCCACAGATGTATAATAGCACAAAAACACTCCTGTGCCTACTGCCTCGGA
**HNRNPAB**	HNRNPAB.1243	TTAAATAAGATGCACATGGGAC	TGCTGTTGACAGTGAGCGATCCCATGTGCATCTTATTTAATAGTGAAGCCACAGATGTATTAAATAAGATGCACATGGGACTGCCTACTGCCTCGGA
**HNRNPAB**	HNRNPAB.608	TCTTTGAACAGGATAAACCCAA	TGCTGTTGACAGTGAGCGCTGGGTTTATCCTGTTCAAAGATAGTGAAGCCACAGATGTATCTTTGAACAGGATAAACCCAATGCCTACTGCCTCGGA
**HNRNPAB**	HNRNPAB.829	TTTTGTTCAACTTTGGATCCAT	TGCTGTTGACAGTGAGCGCTGGATCCAAAGTTGAACAAAATAGTGAAGCCACAGATGTATTTTGTTCAACTTTGGATCCATTGCCTACTGCCTCGGA
**HNRNPAB**	HNRNPAB.1575	TTACAATACATTAGATTCCCAA	TGCTGTTGACAGTGAGCGCTGGGAATCTAATGTATTGTAATAGTGAAGCCACAGATGTATTACAATACATTAGATTCCCAATGCCTACTGCCTCGGA
**HNRNPAB**	HNRNPAB.1063	TTGTACTACCGAATTCCTCGAG	TGCTGTTGACAGTGAGCGATCGAGGAATTCGGTAGTACAATAGTGAAGCCACAGATGTATTGTACTACCGAATTCCTCGAGTGCCTACTGCCTCGGA
**HNRNPD**	HNRNPD.1275	TTGAACTGCTATTAGCAGGTGG	TGCTGTTGACAGTGAGCGACACCTGCTAATAGCAGTTCAATAGTGAAGCCACAGATGTATTGAACTGCTATTAGCAGGTGGTGCCTACTGCCTCGGA
**HNRNPD**	HNRNPD.2045	TATATTTCTTTAATCCTCCCTC	TGCTGTTGACAGTGAGCGAAGGGAGGATTAAAGAAATATATAGTGAAGCCACAGATGTATATATTTCTTTAATCCTCCCTCTGCCTACTGCCTCGGA
**HNRNPD**	HNRNPD.774	TTAGGATCAATCACCTTCCCAT	TGCTGTTGACAGTGAGCGCTGGGAAGGTGATTGATCCTAATAGTGAAGCCACAGATGTATTAGGATCAATCACCTTCCCATTGCCTACTGCCTCGGA
**HNRNPD**	HNRNPD.988	TTCCATTATCTTCTTCACTGGT	TGCTGTTGACAGTGAGCGCCCAGTGAAGAAGATAATGGAATAGTGAAGCCACAGATGTATTCCATTATCTTCTTCACTGGTTGCCTACTGCCTCGGA
**HNRNPD**	HNRNPD.1060	TTGCTGATATTGTTCCTTCGAC	TGCTGTTGACAGTGAGCGATCGAAGGAACAATATCAGCAATAGTGAAGCCACAGATGTATTGCTGATATTGTTCCTTCGACTGCCTACTGCCTCGGA
**HNRNPR**	HNRNPR.2400	TTTAAAAACACAGTTAACCTAC	TGCTGTTGACAGTGAGCGATAGGTTAACTGTGTTTTTAAATAGTGAAGCCACAGATGTATTTAAAAACACAGTTAACCTACTGCCTACTGCCTCGGA
**HNRNPR**	HNRNPR.777	TTCTTTAGTCTTATTCTTCGGA	TGCTGTTGACAGTGAGCGCCCGAAGAATAAGACTAAAGAATAGTGAAGCCACAGATGTATTCTTTAGTCTTATTCTTCGGATGCCTACTGCCTCGGA
**HNRNPR**	HNRNPR.1892	TACTTGTCTACTTCCACTGTTG	TGCTGTTGACAGTGAGCGAAACAGTGGAAGTAGACAAGTATAGTGAAGCCACAGATGTATACTTGTCTACTTCCACTGTTGTGCCTACTGCCTCGGA

Gene Symbol	shRNA Name	Guide	97mer
**HNRNPR**	HNRNPR.1919	TTACTTGTCTACTTCCACTGTT	TGCTGTTGACAGTGAGCGCACAGTGGAAGTAGACAAGTAATAGTGAAGCCACAGATGTATTACTTGTCTACTTCCACTGTTTGCCTACTGCCTCGGA
**HNRNPR**	HNRNPR.510	TTTGCCTACAAATACCTCCGTT	TGCTGTTGACAGTGAGCGCACGGAGGTATTTGTAGGCAAATAGTGAAGCCACAGATGTATTTGCCTACAAATACCTCCGTTTGCCTACTGCCTCGGA
**IL17RD**	IL17RD.5166	TTTTGAAGGACAGTCTTCCTGC	TGCTGTTGACAGTGAGCGACAGGAAGACTGTCCTTCAAAATAGTGAAGCCACAGATGTATTTTGAAGGACAGTCTTCCTGCTGCCTACTGCCTCGGA
**IL17RD**	IL17RD.4714	TAACCTATCTACACAAGCCTTT	TGCTGTTGACAGTGAGCGCAAGGCTTGTGTAGATAGGTTATAGTGAAGCCACAGATGTATAACCTATCTACACAAGCCTTTTGCCTACTGCCTCGGA
**IL17RD**	IL17RD.660	TTTACAAGCTAGATTGTCCGGC	TGCTGTTGACAGTGAGCGACCGGACAATCTAGCTTGTAAATAGTGAAGCCACAGATGTATTTACAAGCTAGATTGTCCGGCTGCCTACTGCCTCGGA
**IL17RD**	IL17RD.1052	TTTTCTTGTTGCTTCTTGCGGC	TGCTGTTGACAGTGAGCGACCGCAAGAAGCAACAAGAAAATAGTGAAGCCACAGATGTATTTTCTTGTTGCTTCTTGCGGCTGCCTACTGCCTCGGA
**IL17RD**	IL17RD.493	TTTGAAGCTACTGTTGAGCTGC	TGCTGTTGACAGTGAGCGACAGCTCAACAGTAGCTTCAAATAGTGAAGCCACAGATGTATTTGAAGCTACTGTTGAGCTGCTGCCTACTGCCTCGGA
**ILF2**	ILF2.527	TAAAACTTCAGAAGGATCCTGT	TGCTGTTGACAGTGAGCGCCAGGATCCTTCTGAAGTTTTATAGTGAAGCCACAGATGTATAAAACTTCAGAAGGATCCTGTTGCCTACTGCCTCGGA
**ILF2**	ILF2.45	TAGCAGACAACTGAAGAGGCGT	TGCTGTTGACAGTGAGCGCCGCCTCTTCAGTTGTCTGCTATAGTGAAGCCACAGATGTATAGCAGACAACTGAAGAGGCGTTGCCTACTGCCTCGGA
**ILF2**	ILF2.1584	TTTCACAACATTTCAACAGCAA	TGCTGTTGACAGTGAGCGCTGCTGTTGAAATGTTGTGAAATAGTGAAGCCACAGATGTATTTCACAACATTTCAACAGCAATGCCTACTGCCTCGGA
**ILF2**	ILF2.1851	TGATATTCTAGTTTACTCTGGT	TGCTGTTGACAGTGAGCGCCCAGAGTAAACTAGAATATCATAGTGAAGCCACAGATGTATGATATTCTAGTTTACTCTGGTTGCCTACTGCCTCGGA
**ILF2**	ILF2.785	TCTGATGAGAACTTTAACTGTG	TGCTGTTGACAGTGAGCGAACAGTTAAAGTTCTCATCAGATAGTGAAGCCACAGATGTATCTGATGAGAACTTTAACTGTGTGCCTACTGCCTCGGA
**ILF3**	ILF3.2024	TTTTGTTGGAACCAGCACCTTG	TGCTGTTGACAGTGAGCGAAAGGTGCTGGTTCCAACAAAATAGTGAAGCCACAGATGTATTTTGTTGGAACCAGCACCTTGTGCCTACTGCCTCGGA
**ILF3**	ILF3.1480	TTTCTTCTGAATCTTCTTCTTC	TGCTGTTGACAGTGAGCGAAAGAAGAAGATTCAGAAGAAATAGTGAAGCCACAGATGTATTTCTTCTGAATCTTCTTCTTCTGCCTACTGCCTCGGA
**ILF3**	ILF3.724	TTCTGTTACAGCAGCAAGCTGG	TGCTGTTGACAGTGAGCGACAGCTTGCTGCTGTAACAGAATAGTGAAGCCACAGATGTATTCTGTTACAGCAGCAAGCTGGTGCCTACTGCCTCGGA
**ILF3**	ILF3.299	TTACTTCTTCTGTAGTGTCTGG	TGCTGTTGACAGTGAGCGACAGACACTACAGAAGAAGTAATAGTGAAGCCACAGATGTATTACTTCTTCTGTAGTGTCTGGTGCCTACTGCCTCGGA
**ILF3**	ILF3.534	TTCTTTACTGTCGTCCTCTGGG	TGCTGTTGACAGTGAGCGACCAGAGGACGACAGTAAAGAATAGTGAAGCCACAGATGTATTCTTTACTGTCGTCCTCTGGGTGCCTACTGCCTCGGA
**IQGAP1**	IQGAP1.7193	TTAACATGAACAAATGACCTTT	TGCTGTTGACAGTGAGCGCAAGGTCATTTGTTCATGTTAATAGTGAAGCCACAGATGTATTAACATGAACAAATGACCTTTTGCCTACTGCCTCGGA
**IQGAP1**	IQGAP1.622	TTCTTCTGTGAAGTCAACCTTT	TGCTGTTGACAGTGAGCGCAAGGTTGACTTCACAGAAGAATAGTGAAGCCACAGATGTATTCTTCTGTGAAGTCAACCTTTTGCCTACTGCCTCGGA
**IQGAP1**	IQGAP1.5499	TAACATGACATTTTAGTTGTGT	TGCTGTTGACAGTGAGCGCCACAACTAAAATGTCATGTTATAGTGAAGCCACAGATGTATAACATGACATTTTAGTTGTGTTGCCTACTGCCTCGGA
**IQGAP1**	IQGAP1.4793	TTTCATGTAGTCTTGCTGCTGT	TGCTGTTGACAGTGAGCGCCAGCAGCAAGACTACATGAAATAGTGAAGCCACAGATGTATTTCATGTAGTCTTGCTGCTGTTGCCTACTGCCTCGGA
**IQGAP1**	IQGAP1.3207	TTGAATCTGATCTACCTTCGAC	TGCTGTTGACAGTGAGCGATCGAAGGTAGATCAGATTCAATAGTGAAGCCACAGATGTATTGAATCTGATCTACCTTCGACTGCCTACTGCCTCGGA
**JUNB**	JUNB.308	TAGCTGTGTATGAGTCGTCGTG	TGCTGTTGACAGTGAGCGAACGACGACTCATACACAGCTATAGTGAAGCCACAGATGTATAGCTGTGTATGAGTCGTCGTGTGCCTACTGCCTCGGA
**JUNB**	JUNB.273	TCCATTTTAGTGCACATCCGGG	TGCTGTTGACAGTGAGCGACCGGATGTGCACTAAAATGGATAGTGAAGCCACAGATGTATCCATTTTAGTGCACATCCGGGTGCCTACTGCCTCGGA
**JUNB**	JUNB.1535	TATGAATCGAGTCTGTTTCCAG	TGCTGTTGACAGTGAGCGATGGAAACAGACTCGATTCATATAGTGAAGCCACAGATGTATATGAATCGAGTCTGTTTCCAGTGCCTACTGCCTCGGA
**JUNB**	JUNB.363	TTCAGGAGTTTGTAGTCGTGTA	TGCTGTTGACAGTGAGCGCACACGACTACAAACTCCTGAATAGTGAAGCCACAGATGTATTCAGGAGTTTGTAGTCGTGTATGCCTACTGCCTCGGA
**JUNB**	JUNB.1241	TGACCTTCTGTTTGAGCTGGGC	TGCTGTTGACAGTGAGCGACCCAGCTCAAACAGAAGGTCATAGTGAAGCCACAGATGTATGACCTTCTGTTTGAGCTGGGCTGCCTACTGCCTCGGA

Gene Symbol	shRNA Name	Guide	97mer
**KLF10**	KLF10.622	TTGCTTTCTCATCAACATCTGC	TGCTGTTGACAGTGAGCGACAGATGTTGATGAGAAAGCAATAGTGAAGCCACAGATGTATTGCTTTCTCATCAACATCTGCTGCCTACTGCCTCGGA
**KLF10**	KLF10.1350	TAGCTTGCTCACTTCCATCTGC	TGCTGTTGACAGTGAGCGACAGATGGAAGTGAGCAAGCTATAGTGAAGCCACAGATGTATAGCTTGCTCACTTCCATCTGCTGCCTACTGCCTCGGA
**KLF10**	KLF10.1822	TACATCTCCATGTCTGTACCGT	TGCTGTTGACAGTGAGCGCCGGTACAGACATGGAGATGTATAGTGAAGCCACAGATGTATACATCTCCATGTCTGTACCGTTGCCTACTGCCTCGGA
**KLF10**	KLF10.1084	TATGTCTTGCCACATCCTGGGT	TGCTGTTGACAGTGAGCGCCCCAGGATGTGGCAAGACATATAGTGAAGCCACAGATGTATATGTCTTGCCACATCCTGGGTTGCCTACTGCCTCGGA
**KLF10**	KLF10.1413	TTCTGACTCTTCACTTTCCGGT	TGCTGTTGACAGTGAGCGCCCGGAAAGTGAAGAGTCAGAATAGTGAAGCCACAGATGTATTCTGACTCTTCACTTTCCGGTTGCCTACTGCCTCGGA
**LRRFIP1**	LRRFIP1.595	TCTTTTGTCATCTTGGTAGGAC	TGCTGTTGACAGTGAGCGATCCTACCAAGATGACAAAAGATAGTGAAGCCACAGATGTATCTTTTGTCATCTTGGTAGGACTGCCTACTGCCTCGGA
**LRRFIP1**	LRRFIP1.259	TATTTCTCTTCAACTTCTGCTA	TGCTGTTGACAGTGAGCGCAGCAGAAGTTGAAGAGAAATATAGTGAAGCCACAGATGTATATTTCTCTTCAACTTCTGCTATGCCTACTGCCTCGGA
**LRRFIP1**	LRRFIP1.445	TGAAATTGCAGTATACTGTGGG	TGCTGTTGACAGTGAGCGACCACAGTATACTGCAATTTCATAGTGAAGCCACAGATGTATGAAATTGCAGTATACTGTGGGTGCCTACTGCCTCGGA
**LRRFIP1**	LRRFIP1.592	TTTGTCATCTTGGTAGGACCTT	TGCTGTTGACAGTGAGCGCAGGTCCTACCAAGATGACAAATAGTGAAGCCACAGATGTATTTGTCATCTTGGTAGGACCTTTGCCTACTGCCTCGGA
**LRRFIP1**	LRRFIP1.262	TTATATTTCTCTTCAACTTCTG	TGCTGTTGACAGTGAGCGAAGAAGTTGAAGAGAAATATAATAGTGAAGCCACAGATGTATTATATTTCTCTTCAACTTCTGTGCCTACTGCCTCGGA
**MAFF**	MAFF.970	TTTCACTAGTCTTCCCACCCAC	TGCTGTTGACAGTGAGCGATGGGTGGGAAGACTAGTGAAATAGTGAAGCCACAGATGTATTTCACTAGTCTTCCCACCCACTGCCTACTGCCTCGGA
**MAFF**	MAFF.1993	TAAATAAATTCTAGGAGCCGGG	TGCTGTTGACAGTGAGCGACCGGCTCCTAGAATTTATTTATAGTGAAGCCACAGATGTATAAATAAATTCTAGGAGCCGGGTGCCTACTGCCTCGGA
**MAFF**	MAFF.708	TAAACTTTCAGCCAAGGGCGAT	TGCTGTTGACAGTGAGCGCTCGCCCTTGGCTGAAAGTTTATAGTGAAGCCACAGATGTATAAACTTTCAGCCAAGGGCGATTGCCTACTGCCTCGGA
**MAFF**	MAFF.2	TCGCTTGATCGAATTCCTCGAG	TGCTGTTGACAGTGAGCGATCGAGGAATTCGATCAAGCGATAGTGAAGCCACAGATGTATCGCTTGATCGAATTCCTCGAGTGCCTACTGCCTCGGA
**MAFF**	MAFF.839	TAGCTTTGAATCCTGGGAGGGT	TGCTGTTGACAGTGAGCGCCCCTCCCAGGATTCAAAGCTATAGTGAAGCCACAGATGTATAGCTTTGAATCCTGGGAGGGTTGCCTACTGCCTCGGA
**MCM2**	MCM2.715	TTCTCTTTGCACATGTCGCTGA	TGCTGTTGACAGTGAGCGCCAGCGACATGTGCAAAGAGAATAGTGAAGCCACAGATGTATTCTCTTTGCACATGTCGCTGATGCCTACTGCCTCGGA
**MCM2**	MCM2.1397	TACAGCAACCTTGTTGTCCTTC	TGCTGTTGACAGTGAGCGAAAGGACAACAAGGTTGCTGTATAGTGAAGCCACAGATGTATACAGCAACCTTGTTGTCCTTCTGCCTACTGCCTCGGA
**MCM2**	MCM2.1017	TACTTGACCATGCTGAGCTGGG	TGCTGTTGACAGTGAGCGACCAGCTCAGCATGGTCAAGTATAGTGAAGCCACAGATGTATACTTGACCATGCTGAGCTGGGTGCCTACTGCCTCGGA
**MCM2**	MCM2.1427	TCATCTTCACATCTTCATCGGT	TGCTGTTGACAGTGAGCGCCCGATGAAGATGTGAAGATGATAGTGAAGCCACAGATGTATCATCTTCACATCTTCATCGGTTGCCTACTGCCTCGGA
**MCM2**	MCM2.1302	TAGTTGTTGTGATAGATGCCAG	TGCTGTTGACAGTGAGCGATGGCATCTATCACAACAACTATAGTGAAGCCACAGATGTATAGTTGTTGTGATAGATGCCAGTGCCTACTGCCTCGGA
**MCM3**	MCM3.246	TTCACATTGACAATCAGCCGGT	TGCTGTTGACAGTGAGCGCCCGGCTGATTGTCAATGTGAATAGTGAAGCCACAGATGTATTCACATTGACAATCAGCCGGTTGCCTACTGCCTCGGA
**MCM3**	MCM3.957	TTACTGAACTTCTTGATCTTGG	TGCTGTTGACAGTGAGCGACAAGATCAAGAAGTTCAGTAATAGTGAAGCCACAGATGTATTACTGAACTTCTTGATCTTGGTGCCTACTGCCTCGGA
**MCM3**	MCM3.958	TTTACTGAACTTCTTGATCTTG	TGCTGTTGACAGTGAGCGAAAGATCAAGAAGTTCAGTAAATAGTGAAGCCACAGATGTATTTACTGAACTTCTTGATCTTGTGCCTACTGCCTCGGA
**MCM3**	MCM3.2977	TTTCTTGACCTGCATGACGTGC	TGCTGTTGACAGTGAGCGACACGTCATGCAGGTCAAGAAATAGTGAAGCCACAGATGTATTTCTTGACCTGCATGACGTGCTGCCTACTGCCTCGGA
**MCM3**	MCM3.3080	TAAGTTTATTCAACATCTCGGA	TGCTGTTGACAGTGAGCGCCCGAGATGTTGAATAAACTTATAGTGAAGCCACAGATGTATAAGTTTATTCAACATCTCGGATGCCTACTGCCTCGGA
**MCM4**	MCM4.216	TTATCGAGGAACAAACTTGGAA	TGCTGTTGACAGTGAGCGCTCCAAGTTTGTTCCTCGATAATAGTGAAGCCACAGATGTATTATCGAGGAACAAACTTGGAATGCCTACTGCCTCGGA
**MCM4**	MCM4.3163	TTTTGAGACAGTCTCACTCTAT	TGCTGTTGACAGTGAGCGCTAGAGTGAGACTGTCTCAAAATAGTGAAGCCACAGATGTATTTTGAGACAGTCTCACTCTATTGCCTACTGCCTCGGA

Gene Symbol	shRNA Name	Guide	97mer
**MCM4**	MCM4.86	TAGGTTTCCATGTTGATTCGGG	TGCTGTTGACAGTGAGCGACCGAATCAACATGGAAACCTATAGTGAAGCCACAGATGTATAGGTTTCCATGTTGATTCGGGTGCCTACTGCCTCGGA
**MCM4**	MCM4.1641	TATTCTTAGTCTTCAATGCGTT	TGCTGTTGACAGTGAGCGCACGCATTGAAGACTAAGAATATAGTGAAGCCACAGATGTATATTCTTAGTCTTCAATGCGTTTGCCTACTGCCTCGGA
**MCM4**	MCM4.217	TTTATCGAGGAACAAACTTGGA	TGCTGTTGACAGTGAGCGCCCAAGTTTGTTCCTCGATAAATAGTGAAGCCACAGATGTATTTATCGAGGAACAAACTTGGATGCCTACTGCCTCGGA
**MCM5**	MCM5.2528	TTTACCTGAACACACCGTGGCT	TGCTGTTGACAGTGAGCGCGCCACGGTGTGTTCAGGTAAATAGTGAAGCCACAGATGTATTTACCTGAACACACCGTGGCTTGCCTACTGCCTCGGA
**MCM5**	MCM5.2529	TTTTACCTGAACACACCGTGGC	TGCTGTTGACAGTGAGCGACCACGGTGTGTTCAGGTAAAATAGTGAAGCCACAGATGTATTTTACCTGAACACACCGTGGCTGCCTACTGCCTCGGA
**MCM5**	MCM5.1636	TTGACGATGAAGATCATGTCGA	TGCTGTTGACAGTGAGCGCCGACATGATCTTCATCGTCAATAGTGAAGCCACAGATGTATTGACGATGAAGATCATGTCGATGCCTACTGCCTCGGA
**MCM5**	MCM5.240	TGTATTTGAAGGTGAAGCCCGT	TGCTGTTGACAGTGAGCGCCGGGCTTCACCTTCAAATACATAGTGAAGCCACAGATGTATGTATTTGAAGGTGAAGCCCGTTGCCTACTGCCTCGGA
**MCM5**	MCM5.343	TTGTACAAGTAGTCGGCCAGGT	TGCTGTTGACAGTGAGCGCCCTGGCCGACTACTTGTACAATAGTGAAGCCACAGATGTATTGTACAAGTAGTCGGCCAGGTTGCCTACTGCCTCGGA
**MCM6**	MCM6.1479	ATGAATAGCAACTTGATCCCGC	TGCTGTTGACAGTGAGCGACGGGATCAAGTTGCTATTCATTAGTGAAGCCACAGATGTAATGAATAGCAACTTGATCCCGCTGCCTACTGCCTCGGA
**MCM6**	MCM6.2172	TTGATTTATGTCTTCATTGTAG	TGCTGTTGACAGTGAGCGATACAATGAAGACATAAATCAATAGTGAAGCCACAGATGTATTGATTTATGTCTTCATTGTAGTGCCTACTGCCTCGGA
**MCM6**	MCM6.2798	TTTGGTTCCAACTTCACTGGGA	TGCTGTTGACAGTGAGCGCCCCAGTGAAGTTGGAACCAAATAGTGAAGCCACAGATGTATTTGGTTCCAACTTCACTGGGATGCCTACTGCCTCGGA
**MCM6**	MCM6.2096	TTGATCTAGATTGACATCAGGT	TGCTGTTGACAGTGAGCGCCCTGATGTCAATCTAGATCAATAGTGAAGCCACAGATGTATTGATCTAGATTGACATCAGGTTGCCTACTGCCTCGGA
**MCM6**	MCM6.2807	TTACACATGAAAACAAAGGTAT	TGCTGTTGACAGTGAGCGCTACCTTTGTTTTCATGTGTAATAGTGAAGCCACAGATGTATTACACATGAAAACAAAGGTATTGCCTACTGCCTCGGA
**MCM7**	MCM7.1879	TTTGACATCTCCATTAGCCTGA	TGCTGTTGACAGTGAGCGCCAGGCTAATGGAGATGTCAAATAGTGAAGCCACAGATGTATTTGACATCTCCATTAGCCTGATGCCTACTGCCTCGGA
**MCM7**	MCM7.897	TTGTTCATCTTCACAATCCGAT	TGCTGTTGACAGTGAGCGCTCGGATTGTGAAGATGAACAATAGTGAAGCCACAGATGTATTGTTCATCTTCACAATCCGATTGCCTACTGCCTCGGA
**MCM7**	MCM7.8	TTGTAAGAACTTCTTAACCTTT	TGCTGTTGACAGTGAGCGCAAGGTTAAGAAGTTCTTACAATAGTGAAGCCACAGATGTATTGTAAGAACTTCTTAACCTTTTGCCTACTGCCTCGGA
**MCM7**	MCM7.60	TACTTGAACTGCTTCTTCCCGA	TGCTGTTGACAGTGAGCGCCGGGAAGAAGCAGTTCAAGTATAGTGAAGCCACAGATGTATACTTGAACTGCTTCTTCCCGATGCCTACTGCCTCGGA
**MCM7**	MCM7.674	TTCTTGCATCTTCATCTCCTGG	TGCTGTTGACAGTGAGCGACAGGAGATGAAGATGCAAGAATAGTGAAGCCACAGATGTATTCTTGCATCTTCATCTCCTGGTGCCTACTGCCTCGGA
**MED8**	MED8.2	TTTTCCAGTCGAATTCCTCGAG	TGCTGTTGACAGTGAGCGATCGAGGAATTCGACTGGAAAATAGTGAAGCCACAGATGTATTTTCCAGTCGAATTCCTCGAGTGCCTACTGCCTCGGA
**MED8**	MED8.561	TTAAAGGTCTGCTTGTTCGGCC	TGCTGTTGACAGTGAGCGAGCCGAACAAGCAGACCTTTAATAGTGAAGCCACAGATGTATTAAAGGTCTGCTTGTTCGGCCTGCCTACTGCCTCGGA
**MED8**	MED8.222	TTGTTCAGAGTGTTCAGCTGTC	TGCTGTTGACAGTGAGCGAACAGCTGAACACTCTGAACAATAGTGAAGCCACAGATGTATTGTTCAGAGTGTTCAGCTGTCTGCCTACTGCCTCGGA
**MED8**	MED8.405	TTCTCCTGTTCTTCCACTTCAG	TGCTGTTGACAGTGAGCGATGAAGTGGAAGAACAGGAGAATAGTGAAGCCACAGATGTATTCTCCTGTTCTTCCACTTCAGTGCCTACTGCCTCGGA
**MED8**	MED8.474	TTATTCAAGCTCTGGATCTGCT	TGCTGTTGACAGTGAGCGCGCAGATCCAGAGCTTGAATAATAGTGAAGCCACAGATGTATTATTCAAGCTCTGGATCTGCTTGCCTACTGCCTCGGA
**MYBL2**	MYBL2.2073	TTCTTTGATACCTGACAGGGTG	TGCTGTTGACAGTGAGCGAACCCTGTCAGGTATCAAAGAATAGTGAAGCCACAGATGTATTCTTTGATACCTGACAGGGTGTGCCTACTGCCTCGGA
**MYBL2**	MYBL2.2234	TTCTCCTGCATGAAAAGCTGGT	TGCTGTTGACAGTGAGCGCCCAGCTTTTCATGCAGGAGAATAGTGAAGCCACAGATGTATTCTCCTGCATGAAAAGCTGGTTGCCTACTGCCTCGGA
**MYBL2**	MYBL2.1874	TCGATGATGAGTTCGATGCCAG	TGCTGTTGACAGTGAGCGATGGCATCGAACTCATCATCGATAGTGAAGCCACAGATGTATCGATGATGAGTTCGATGCCAGTGCCTACTGCCTCGGA
**MYBL2**	MYBL2.875	TTGGTCAGAAGACTTCCCTGGC	TGCTGTTGACAGTGAGCGACCAGGGAAGTCTTCTGACCAATAGTGAAGCCACAGATGTATTGGTCAGAAGACTTCCCTGGCTGCCTACTGCCTCGGA

Gene Symbol	shRNA Name	Guide	97mer
**MYBL2**	MYBL2.1535	TTAACAGGTGTGCTCTTGGGCG	TGCTGTTGACAGTGAGCGAGCCCAAGAGCACACCTGTTAATAGTGAAGCCACAGATGTATTAACAGGTGTGCTCTTGGGCGTGCCTACTGCCTCGGA
**NAP1L4**	NAP1L4.362	TATGTGAGCACATCTCACCTGA	TGCTGTTGACAGTGAGCGCCAGGTGAGATGTGCTCACATATAGTGAAGCCACAGATGTATATGTGAGCACATCTCACCTGATGCCTACTGCCTCGGA
**NAP1L4**	NAP1L4.2472	TAGAATATTCTAACTATTCTGT	TGCTGTTGACAGTGAGCGCCAGAATAGTTAGAATATTCTATAGTGAAGCCACAGATGTATAGAATATTCTAACTATTCTGTTGCCTACTGCCTCGGA
**NAP1L4**	NAP1L4.740	TAACACAAAAGACATAGGCTGT	TGCTGTTGACAGTGAGCGCCAGCCTATGTCTTTTGTGTTATAGTGAAGCCACAGATGTATAACACAAAAGACATAGGCTGTTGCCTACTGCCTCGGA
**NAP1L4**	NAP1L4.909	TTGACAGTAACATTCTTTCCTT	TGCTGTTGACAGTGAGCGCAGGAAAGAATGTTACTGTCAATAGTGAAGCCACAGATGTATTGACAGTAACATTCTTTCCTTTGCCTACTGCCTCGGA
**NAP1L4**	NAP1L4.885	TTCCAGTCAATAGTACACCCGT	TGCTGTTGACAGTGAGCGCCGGGTGTACTATTGACTGGAATAGTGAAGCCACAGATGTATTCCAGTCAATAGTACACCCGTTGCCTACTGCCTCGGA
**NF1**	NF1.898	TATATCATGAACATCAACATTG	TGCTGTTGACAGTGAGCGAAATGTTGATGTTCATGATATATAGTGAAGCCACAGATGTATATATCATGAACATCAACATTGTGCCTACTGCCTCGGA
**NF1**	NF1.898	TATATCATGAACATCAACATTG	TGCTGTTGACAGTGAGCGAAATGTTGATGTTCATGATATATAGTGAAGCCACAGATGTATATATCATGAACATCAACATTGTGCCTACTGCCTCGGA
**NF1**	NF1.1588	TAGAAGGTGAATTCTGAGCCAG	TGCTGTTGACAGTGAGCGATGGCTCAGAATTCACCTTCTATAGTGAAGCCACAGATGTATAGAAGGTGAATTCTGAGCCAGTGCCTACTGCCTCGGA
**NF1**	NF1.1588	TAGAAGGTGAATTCTGAGCCAG	TGCTGTTGACAGTGAGCGATGGCTCAGAATTCACCTTCTATAGTGAAGCCACAGATGTATAGAAGGTGAATTCTGAGCCAGTGCCTACTGCCTCGGA
**NF1**	NF1.561	TAAAATAGTAGTGAGGCCGCTT	TGCTGTTGACAGTGAGCGCAGCGGCCTCACTACTATTTTATAGTGAAGCCACAGATGTATAAAATAGTAGTGAGGCCGCTTTGCCTACTGCCTCGGA
**NF1**	NF1.561	TAAAATAGTAGTGAGGCCGCTT	TGCTGTTGACAGTGAGCGCAGCGGCCTCACTACTATTTTATAGTGAAGCCACAGATGTATAAAATAGTAGTGAGGCCGCTTTGCCTACTGCCTCGGA
**NF1**	NF1.1785	TTCTTTAAATGTAAGACTCGGT	TGCTGTTGACAGTGAGCGCCCGAGTCTTACATTTAAAGAATAGTGAAGCCACAGATGTATTCTTTAAATGTAAGACTCGGTTGCCTACTGCCTCGGA
**NF1**	NF1.1785	TTCTTTAAATGTAAGACTCGGT	TGCTGTTGACAGTGAGCGCCCGAGTCTTACATTTAAAGAATAGTGAAGCCACAGATGTATTCTTTAAATGTAAGACTCGGTTGCCTACTGCCTCGGA
**NF1**	NF1.1361	TTACACAGTTTGACACAGGCAA	TGCTGTTGACAGTGAGCGCTGCCTGTGTCAAACTGTGTAATAGTGAAGCCACAGATGTATTACACAGTTTGACACAGGCAATGCCTACTGCCTCGGA
**NF1**	NF1.1361	TTACACAGTTTGACACAGGCAA	TGCTGTTGACAGTGAGCGCTGCCTGTGTCAAACTGTGTAATAGTGAAGCCACAGATGTATTACACAGTTTGACACAGGCAATGCCTACTGCCTCGGA
**NFE2L3**	NFE2L3.943	TTCATCATCATCATTCTGCTGA	TGCTGTTGACAGTGAGCGCCAGCAGAATGATGATGATGAATAGTGAAGCCACAGATGTATTCATCATCATCATTCTGCTGATGCCTACTGCCTCGGA
**NFE2L3**	NFE2L3.1541	TATTATTGTGACTTGAATCTAA	TGCTGTTGACAGTGAGCGCTAGATTCAAGTCACAATAATATAGTGAAGCCACAGATGTATATTATTGTGACTTGAATCTAATGCCTACTGCCTCGGA
**NFE2L3**	NFE2L3.3599	TACTCTACACTGTAGCTCCTAT	TGCTGTTGACAGTGAGCGCTAGGAGCTACAGTGTAGAGTATAGTGAAGCCACAGATGTATACTCTACACTGTAGCTCCTATTGCCTACTGCCTCGGA
**NFE2L3**	NFE2L3.350	TAAAGATCTAGGTCTACGCGGA	TGCTGTTGACAGTGAGCGCCCGCGTAGACCTAGATCTTTATAGTGAAGCCACAGATGTATAAAGATCTAGGTCTACGCGGATGCCTACTGCCTCGGA
**NFE2L3**	NFE2L3.1962	TAATATCTACTTAACATGCTAT	TGCTGTTGACAGTGAGCGCTAGCATGTTAAGTAGATATTATAGTGAAGCCACAGATGTATAATATCTACTTAACATGCTATTGCCTACTGCCTCGGA
**NFKB1**	NFKB1.1378	TTGTCTATGAACATCTGTGGGG	TGCTGTTGACAGTGAGCGACCCACAGATGTTCATAGACAATAGTGAAGCCACAGATGTATTGTCTATGAACATCTGTGGGGTGCCTACTGCCTCGGA
**NFKB1**	NFKB1.2926	TTCTAGTAACTTATACAGCTGC	TGCTGTTGACAGTGAGCGACAGCTGTATAAGTTACTAGAATAGTGAAGCCACAGATGTATTCTAGTAACTTATACAGCTGCTGCCTACTGCCTCGGA
**NFKB1**	NFKB1.3703	TTTAATGACAATAGGAACGTAG	TGCTGTTGACAGTGAGCGATACGTTCCTATTGTCATTAAATAGTGAAGCCACAGATGTATTTAATGACAATAGGAACGTAGTGCCTACTGCCTCGGA
**NFKB1**	NFKB1.2920	TAACTTATACAGCTGCAGCTTC	TGCTGTTGACAGTGAGCGAAAGCTGCAGCTGTATAAGTTATAGTGAAGCCACAGATGTATAACTTATACAGCTGCAGCTTCTGCCTACTGCCTCGGA
**NFKB1**	NFKB1.2367	TTGTGCTTGAGTAAGATACTGA	TGCTGTTGACAGTGAGCGCCAGTATCTTACTCAAGCACAATAGTGAAGCCACAGATGTATTGTGCTTGAGTAAGATACTGATGCCTACTGCCTCGGA
**PA2G4**	PA2G4.706	TTCCTTGAGAATATAATCCTGG	TGCTGTTGACAGTGAGCGACAGGATTATATTCTCAAGGAATAGTGAAGCCACAGATGTATTCCTTGAGAATATAATCCTGGTGCCTACTGCCTCGGA

Gene Symbol	shRNA Name	Guide	97mer
**PA2G4**	PA2G4.994	TTCTCCATCGATGACATGCTGC	TGCTGTTGACAGTGAGCGACAGCATGTCATCGATGGAGAATAGTGAAGCCACAGATGTATTCTCCATCGATGACATGCTGCTGCCTACTGCCTCGGA
**PA2G4**	PA2G4.1492	TTCGACTTGCAGAACTCTGGAG	TGCTGTTGACAGTGAGCGATCCAGAGTTCTGCAAGTCGAATAGTGAAGCCACAGATGTATTCGACTTGCAGAACTCTGGAGTGCCTACTGCCTCGGA
**PA2G4**	PA2G4.1812	TCATTTAAAGTAGTCTTCCGTG	TGCTGTTGACAGTGAGCGAACGGAAGACTACTTTAAATGATAGTGAAGCCACAGATGTATCATTTAAAGTAGTCTTCCGTGTGCCTACTGCCTCGGA
**PA2G4**	PA2G4.1809	TTTAAAGTAGTCTTCCGTGGTT	TGCTGTTGACAGTGAGCGCACCACGGAAGACTACTTTAAATAGTGAAGCCACAGATGTATTTAAAGTAGTCTTCCGTGGTTTGCCTACTGCCTCGGA
**PHTF2**	PHTF2.323	TTACTTGTAACCACCACCGGAA	TGCTGTTGACAGTGAGCGCTCCGGTGGTGGTTACAAGTAATAGTGAAGCCACAGATGTATTACTTGTAACCACCACCGGAATGCCTACTGCCTCGGA
**PHTF2**	PHTF2.269	TACAACTCGAACAATTCCCTTT	TGCTGTTGACAGTGAGCGCAAGGGAATTGTTCGAGTTGTATAGTGAAGCCACAGATGTATACAACTCGAACAATTCCCTTTTGCCTACTGCCTCGGA
**PHTF2**	PHTF2.808	TTGTATTCCATCTTCACCGCTT	TGCTGTTGACAGTGAGCGCAGCGGTGAAGATGGAATACAATAGTGAAGCCACAGATGTATTGTATTCCATCTTCACCGCTTTGCCTACTGCCTCGGA
**PHTF2**	PHTF2.993	TTCTATTCCGAAGAACACCTTC	TGCTGTTGACAGTGAGCGAAAGGTGTTCTTCGGAATAGAATAGTGAAGCCACAGATGTATTCTATTCCGAAGAACACCTTCTGCCTACTGCCTCGGA
**PHTF2**	PHTF2.714	TATCAATTGAATTCTTTGCTTT	TGCTGTTGACAGTGAGCGCAAGCAAAGAATTCAATTGATATAGTGAAGCCACAGATGTATATCAATTGAATTCTTTGCTTTTGCCTACTGCCTCGGA
**PMEPA1**	PMEPA1.4210	TTAACTTGAACAGAGCTTGGGA	TGCTGTTGACAGTGAGCGCCCCAAGCTCTGTTCAAGTTAATAGTGAAGCCACAGATGTATTAACTTGAACAGAGCTTGGGATGCCTACTGCCTCGGA
**PMEPA1**	PMEPA1.3541	TAACGTGACAACTACCATCTAG	TGCTGTTGACAGTGAGCGATAGATGGTAGTTGTCACGTTATAGTGAAGCCACAGATGTATAACGTGACAACTACCATCTAGTGCCTACTGCCTCGGA
**PMEPA1**	PMEPA1.4018	TTTTCTTTCCTTATCGACGGGA	TGCTGTTGACAGTGAGCGCCCCGTCGATAAGGAAAGAAAATAGTGAAGCCACAGATGTATTTTCTTTCCTTATCGACGGGATGCCTACTGCCTCGGA
**PMEPA1**	PMEPA1.2011	TTAAGTGAGAATTGATCCGTGA	TGCTGTTGACAGTGAGCGCCACGGATCAATTCTCACTTAATAGTGAAGCCACAGATGTATTAAGTGAGAATTGATCCGTGATGCCTACTGCCTCGGA
**PMEPA1**	PMEPA1.2873	TTAATCATCTTTACAAGTGCGT	TGCTGTTGACAGTGAGCGCCGCACTTGTAAAGATGATTAATAGTGAAGCCACAGATGTATTAATCATCTTTACAAGTGCGTTGCCTACTGCCTCGGA
**PML**	PML.1271	TTGAACTCGTCGAAGCCATCGG	TGCTGTTGACAGTGAGCGACGATGGCTTCGACGAGTTCAATAGTGAAGCCACAGATGTATTGAACTCGTCGAAGCCATCGGTGCCTACTGCCTCGGA
**PML**	PML.105	TTTAGATCTTGGAGTGCGTGAA	TGCTGTTGACAGTGAGCGCTCACGCACTCCAAGATCTAAATAGTGAAGCCACAGATGTATTTAGATCTTGGAGTGCGTGAATGCCTACTGCCTCGGA
**PML**	PML.109	TCGGTTTAGATCTTGGAGTGCG	TGCTGTTGACAGTGAGCGAGCACTCCAAGATCTAAACCGATAGTGAAGCCACAGATGTATCGGTTTAGATCTTGGAGTGCGTGCCTACTGCCTCGGA
**PML**	PML.1148	TAGCACTTCATCCTCTGCACCA	TGCTGTTGACAGTGAGCGCGGTGCAGAGGATGAAGTGCTATAGTGAAGCCACAGATGTATAGCACTTCATCCTCTGCACCATGCCTACTGCCTCGGA
**PML**	PML.111	TCTCGGTTTAGATCTTGGAGTG	TGCTGTTGACAGTGAGCGAACTCCAAGATCTAAACCGAGATAGTGAAGCCACAGATGTATCTCGGTTTAGATCTTGGAGTGTGCCTACTGCCTCGGA
**PNN**	PNN.401	TTTAACATCATCATCCTCCGGG	TGCTGTTGACAGTGAGCGACCGGAGGATGATGATGTTAAATAGTGAAGCCACAGATGTATTTAACATCATCATCCTCCGGGTGCCTACTGCCTCGGA
**PNN**	PNN.1071	TATTTCTACATCATTGTGCTGA	TGCTGTTGACAGTGAGCGCCAGCACAATGATGTAGAAATATAGTGAAGCCACAGATGTATATTTCTACATCATTGTGCTGATGCCTACTGCCTCGGA
**PNN**	PNN.2904	TACTGTTTAGTTATCTACCGAA	TGCTGTTGACAGTGAGCGCTCGGTAGATAACTAAACAGTATAGTGAAGCCACAGATGTATACTGTTTAGTTATCTACCGAATGCCTACTGCCTCGGA
**PNN**	PNN.2847	TTAGATTAAAATAACAAGGGAG	TGCTGTTGACAGTGAGCGATCCCTTGTTATTTTAATCTAATAGTGAAGCCACAGATGTATTAGATTAAAATAACAAGGGAGTGCCTACTGCCTCGGA
**PNN**	PNN.400	TTAACATCATCATCCTCCGGGT	TGCTGTTGACAGTGAGCGCCCCGGAGGATGATGATGTTAATAGTGAAGCCACAGATGTATTAACATCATCATCCTCCGGGTTGCCTACTGCCTCGGA
**POLR1E**	POLR1E.1199	TTGGCTATCTCCATCATCCTTT	TGCTGTTGACAGTGAGCGCAAGGATGATGGAGATAGCCAATAGTGAAGCCACAGATGTATTGGCTATCTCCATCATCCTTTTGCCTACTGCCTCGGA
**POLR1E**	POLR1E.501	TTCAATACAAGAATCCATCTTT	TGCTGTTGACAGTGAGCGCAAGATGGATTCTTGTATTGAATAGTGAAGCCACAGATGTATTCAATACAAGAATCCATCTTTTGCCTACTGCCTCGGA
**POLR1E**	POLR1E.815	TTCAGTATTTCTTCTGACGTGA	TGCTGTTGACAGTGAGCGCCACGTCAGAAGAAATACTGAATAGTGAAGCCACAGATGTATTCAGTATTTCTTCTGACGTGATGCCTACTGCCTCGGA

Gene Symbol	shRNA Name	Guide	97mer
**POLR1E**	POLR1E.1264	TTGTGATCTTCTTCACTGCCGG	TGCTGTTGACAGTGAGCGACGGCAGTGAAGAAGATCACAATAGTGAAGCCACAGATGTATTGTGATCTTCTTCACTGCCGGTGCCTACTGCCTCGGA
**POLR1E**	POLR1E.467	TTTGGTCTGACTCTCTAGCGCC	TGCTGTTGACAGTGAGCGAGCGCTAGAGAGTCAGACCAAATAGTGAAGCCACAGATGTATTTGGTCTGACTCTCTAGCGCCTGCCTACTGCCTCGGA
**POLR2F**	POLR2F.1308	TCTGACTTAATGTAACACCTGG	TGCTGTTGACAGTGAGCGACAGGTGTTACATTAAGTCAGATAGTGAAGCCACAGATGTATCTGACTTAATGTAACACCTGGTGCCTACTGCCTCGGA
**POLR2F**	POLR2F.1300	TAATGTAACACCTGGAACCCAG	TGCTGTTGACAGTGAGCGATGGGTTCCAGGTGTTACATTATAGTGAAGCCACAGATGTATAATGTAACACCTGGAACCCAGTGCCTACTGCCTCGGA
**POLR2F**	POLR2F.112	TTGTCTGACATGACACCCTCGC	TGCTGTTGACAGTGAGCGACGAGGGTGTCATGTCAGACAATAGTGAAGCCACAGATGTATTGTCTGACATGACACCCTCGCTGCCTACTGCCTCGGA
**POLR2F**	POLR2F.181	TTCTCCAAGTCATCTAGCCCTT	TGCTGTTGACAGTGAGCGCAGGGCTAGATGACTTGGAGAATAGTGAAGCCACAGATGTATTCTCCAAGTCATCTAGCCCTTTGCCTACTGCCTCGGA
**POLR2F**	POLR2F.1305	TGACTTAATGTAACACCTGGAA	TGCTGTTGACAGTGAGCGCTCCAGGTGTTACATTAAGTCATAGTGAAGCCACAGATGTATGACTTAATGTAACACCTGGAATGCCTACTGCCTCGGA
**POLR3K**	POLR3K.166	TTCAGTTTTGGGTACTTCCGAT	TGCTGTTGACAGTGAGCGCTCGGAAGTACCCAAAACTGAATAGTGAAGCCACAGATGTATTCAGTTTTGGGTACTTCCGATTGCCTACTGCCTCGGA
**POLR3K**	POLR3K.726	TAACAGTTAAATATTTAGGGAC	TGCTGTTGACAGTGAGCGATCCCTAAATATTTAACTGTTATAGTGAAGCCACAGATGTATAACAGTTAAATATTTAGGGACTGCCTACTGCCTCGGA
**POLR3K**	POLR3K.313	TTGTAGAAGGTGGTCATCGGCT	TGCTGTTGACAGTGAGCGCGCCGATGACCACCTTCTACAATAGTGAAGCCACAGATGTATTGTAGAAGGTGGTCATCGGCTTGCCTACTGCCTCGGA
**POLR3K**	POLR3K.676	TATTTAAGAGTGTTGATTGGGA	TGCTGTTGACAGTGAGCGCCCAATCAACACTCTTAAATATAGTGAAGCCACAGATGTATATTTAAGAGTGTTGATTGGGATGCCTACTGCCTCGGA
**POLR3K**	POLR3K.151	TTCCGATTTGTTACCTTGCGGG	TGCTGTTGACAGTGAGCGACCGCAAGGTAACAAATCGGAATAGTGAAGCCACAGATGTATTCCGATTTGTTACCTTGCGGGTGCCTACTGCCTCGGA
**PPP1R15A**	PPP1R15A.49	TTGCATAAGATCAACAACTGGG	TGCTGTTGACAGTGAGCGACCAGTTGTTGATCTTATGCAATAGTGAAGCCACAGATGTATTGCATAAGATCAACAACTGGGTGCCTACTGCCTCGGA
**PPP1R15A**	PPP1R15A.963	TTCTGTTCTTTTATCCTCCGTG	TGCTGTTGACAGTGAGCGAACGGAGGATAAAAGAACAGAATAGTGAAGCCACAGATGTATTCTGTTCTTTTATCCTCCGTGTGCCTACTGCCTCGGA
**PPP1R15A**	PPP1R15A.1494	TTCTCTTTCATCCTCGGCTGAT	TGCTGTTGACAGTGAGCGCTCAGCCGAGGATGAAAGAGAATAGTGAAGCCACAGATGTATTCTCTTTCATCCTCGGCTGATTGCCTACTGCCTCGGA
**PPP1R15A**	PPP1R15A.2344	TTTATTCCTTATATAAACCCAC	TGCTGTTGACAGTGAGCGATGGGTTTATATAAGGAATAAATAGTGAAGCCACAGATGTATTTATTCCTTATATAAACCCACTGCCTACTGCCTCGGA
**PPP1R15A**	PPP1R15A.964	TTTCTGTTCTTTTATCCTCCGT	TGCTGTTGACAGTGAGCGCCGGAGGATAAAAGAACAGAAATAGTGAAGCCACAGATGTATTTCTGTTCTTTTATCCTCCGTTGCCTACTGCCTCGGA
**PPP2R5A**	PPP2R5A.694	TACAACACAATATGCTAGCTGA	TGCTGTTGACAGTGAGCGCCAGCTAGCATATTGTGTTGTATAGTGAAGCCACAGATGTATACAACACAATATGCTAGCTGATGCCTACTGCCTCGGA
**PPP2R5A**	PPP2R5A.49	AAACAGTATACAACACTGCTGC	TGCTGTTGACAGTGAGCGACAGCAGTGTTGTATACTGTTTTAGTGAAGCCACAGATGTAAAACAGTATACAACACTGCTGCTGCCTACTGCCTCGGA
**PPP2R5A**	PPP2R5A.730	TTAGTGTTGTATCTTTCTCCAG	TGCTGTTGACAGTGAGCGATGGAGAAAGATACAACACTAATAGTGAAGCCACAGATGTATTAGTGTTGTATCTTTCTCCAGTGCCTACTGCCTCGGA
**PPP2R5A**	PPP2R5A.96	TTCTTTGCTCTTCAAGTCTGAA	TGCTGTTGACAGTGAGCGCTCAGACTTGAAGAGCAAAGAATAGTGAAGCCACAGATGTATTCTTTGCTCTTCAAGTCTGAATGCCTACTGCCTCGGA
**PPP2R5A**	PPP2R5A.183	TACTATATCAGAATACGCTGAT	TGCTGTTGACAGTGAGCGCTCAGCGTATTCTGATATAGTATAGTGAAGCCACAGATGTATACTATATCAGAATACGCTGATTGCCTACTGCCTCGGA
**PPP2R5B**	PPP2R5B.1181	TTTTGATCCACATATCTCTTGG	TGCTGTTGACAGTGAGCGACAAGAGATATGTGGATCAAAATAGTGAAGCCACAGATGTATTTTGATCCACATATCTCTTGGTGCCTACTGCCTCGGA
**PPP2R5B**	PPP2R5B.2733	TTTTCTTGAATTTCTCTCTTTA	TGCTGTTGACAGTGAGCGCAAAGAGAGAAATTCAAGAAAATAGTGAAGCCACAGATGTATTTTCTTGAATTTCTCTCTTTATGCCTACTGCCTCGGA
**PPP2R5B**	PPP2R5B.1342	TCGAATTCATAGATGAACCGGA	TGCTGTTGACAGTGAGCGCCCGGTTCATCTATGAATTCGATAGTGAAGCCACAGATGTATCGAATTCATAGATGAACCGGATGCCTACTGCCTCGGA
**PPP2R5B**	PPP2R5B.1178	TTGATCCACATATCTCTTGGCC	TGCTGTTGACAGTGAGCGAGCCAAGAGATATGTGGATCAATAGTGAAGCCACAGATGTATTGATCCACATATCTCTTGGCCTGCCTACTGCCTCGGA
**PPP2R5B**	PPP2R5B.1879	TTGAGCACATTGTAGATCAGTG	TGCTGTTGACAGTGAGCGAACTGATCTACAATGTGCTCAATAGTGAAGCCACAGATGTATTGAGCACATTGTAGATCAGTGTGCCTACTGCCTCGGA

Gene Symbol	shRNA Name	Guide	97mer
**PPP2R5C**	PPP2R5C.1208	TAGATTTTCTATTTTAACCCAT	TGCTGTTGACAGTGAGCGCTGGGTTAAAATAGAAAATCTATAGTGAAGCCACAGATGTATAGATTTTCTATTTTAACCCATTGCCTACTGCCTCGGA
**PPP2R5C**	PPP2R5C.118	TTTCACTTAAAGCAGCTCGTTT	TGCTGTTGACAGTGAGCGCAACGAGCTGCTTTAAGTGAAATAGTGAAGCCACAGATGTATTTCACTTAAAGCAGCTCGTTTTGCCTACTGCCTCGGA
**PPP2R5C**	PPP2R5C.1159	TTCTCTTTTAGTTTCTCTGCTT	TGCTGTTGACAGTGAGCGCAGCAGAGAAACTAAAAGAGAATAGTGAAGCCACAGATGTATTCTCTTTTAGTTTCTCTGCTTTGCCTACTGCCTCGGA
**PPP2R5C**	PPP2R5C.631	TTGTGCTCTTCTTTTAGTGGTA	TGCTGTTGACAGTGAGCGCACCACTAAAAGAAGAGCACAATAGTGAAGCCACAGATGTATTGTGCTCTTCTTTTAGTGGTATGCCTACTGCCTCGGA
**PPP2R5C**	PPP2R5C.362	TATATTAGGTTGGAAATCTGGA	TGCTGTTGACAGTGAGCGCCCAGATTTCCAACCTAATATATAGTGAAGCCACAGATGTATATATTAGGTTGGAAATCTGGATGCCTACTGCCTCGGA
**PPP2R5E**	PPP2R5E.679	TAAACTGTGAGGAACTTTGCGA	TGCTGTTGACAGTGAGCGCCGCAAAGTTCCTCACAGTTTATAGTGAAGCCACAGATGTATAAACTGTGAGGAACTTTGCGATGCCTACTGCCTCGGA
**PPP2R5E**	PPP2R5E.2683	TAAACTGATGCATGAATCCGAT	TGCTGTTGACAGTGAGCGCTCGGATTCATGCATCAGTTTATAGTGAAGCCACAGATGTATAAACTGATGCATGAATCCGATTGCCTACTGCCTCGGA
**PPP2R5E**	PPP2R5E.1240	TATTGTTAATCTGTTTTCGGAT	TGCTGTTGACAGTGAGCGCTCCGAAAACAGATTAACAATATAGTGAAGCCACAGATGTATATTGTTAATCTGTTTTCGGATTGCCTACTGCCTCGGA
**PPP2R5E**	PPP2R5E.1269	TTCTGTTTCATAAACAAACCTT	TGCTGTTGACAGTGAGCGCAGGTTTGTTTATGAAACAGAATAGTGAAGCCACAGATGTATTCTGTTTCATAAACAAACCTTTGCCTACTGCCTCGGA
**PPP2R5E**	PPP2R5E.2031	TTCTACTACATAAACGTGTGAC	TGCTGTTGACAGTGAGCGATCACACGTTTATGTAGTAGAATAGTGAAGCCACAGATGTATTCTACTACATAAACGTGTGACTGCCTACTGCCTCGGA
**PTTG1**	PTTG1.670	TACAAATACACACAAACTCTGA	TGCTGTTGACAGTGAGCGCCAGAGTTTGTGTGTATTTGTATAGTGAAGCCACAGATGTATACAAATACACACAAACTCTGATGCCTACTGCCTCGGA
**PTTG1**	PTTG1.327	TTTGCTTTAACAGTCTTCTCAG	TGCTGTTGACAGTGAGCGATGAGAAGACTGTTAAAGCAAATAGTGAAGCCACAGATGTATTTGCTTTAACAGTCTTCTCAGTGCCTACTGCCTCGGA
**PTTG1**	PTTG1.18	TATTGCAGGTCTTAACAGCCGC	TGCTGTTGACAGTGAGCGACGGCTGTTAAGACCTGCAATATAGTGAAGCCACAGATGTATATTGCAGGTCTTAACAGCCGCTGCCTACTGCCTCGGA
**PTTG1**	PTTG1.236	TAGCTCTGTTGACAGTTCCCAA	TGCTGTTGACAGTGAGCGCTGGGAACTGTCAACAGAGCTATAGTGAAGCCACAGATGTATAGCTCTGTTGACAGTTCCCAATGCCTACTGCCTCGGA
**PTTG1**	PTTG1.7	TTAACAGCCGCATTCATCTGAG	TGCTGTTGACAGTGAGCGATCAGATGAATGCGGCTGTTAATAGTGAAGCCACAGATGTATTAACAGCCGCATTCATCTGAGTGCCTACTGCCTCGGA
**RASA1**	RASA1.1954	TTATCTTTAAAATAGAGTCTTT	TGCTGTTGACAGTGAGCGCAAGACTCTATTTTAAAGATAATAGTGAAGCCACAGATGTATTATCTTTAAAATAGAGTCTTTTGCCTACTGCCTCGGA
**RASA1**	RASA1.1954	TTATCTTTAAAATAGAGTCTTT	TGCTGTTGACAGTGAGCGCAAGACTCTATTTTAAAGATAATAGTGAAGCCACAGATGTATTATCTTTAAAATAGAGTCTTTTGCCTACTGCCTCGGA
**RASA1**	RASA1.396	TAGAATAGCTCGTACACGCCTT	TGCTGTTGACAGTGAGCGCAGGCGTGTACGAGCTATTCTATAGTGAAGCCACAGATGTATAGAATAGCTCGTACACGCCTTTGCCTACTGCCTCGGA
**RASA1**	RASA1.396	TAGAATAGCTCGTACACGCCTT	TGCTGTTGACAGTGAGCGCAGGCGTGTACGAGCTATTCTATAGTGAAGCCACAGATGTATAGAATAGCTCGTACACGCCTTTGCCTACTGCCTCGGA
**RASA1**	RASA1.573	TTCATGTGGATCTTCTTCCCGG	TGCTGTTGACAGTGAGCGACGGGAAGAAGATCCACATGAATAGTGAAGCCACAGATGTATTCATGTGGATCTTCTTCCCGGTGCCTACTGCCTCGGA
**RASA1**	RASA1.573	TTCATGTGGATCTTCTTCCCGG	TGCTGTTGACAGTGAGCGACGGGAAGAAGATCCACATGAATAGTGAAGCCACAGATGTATTCATGTGGATCTTCTTCCCGGTGCCTACTGCCTCGGA
**RASA1**	RASA1.2153	TAAATATATCTCAATGTCGGTG	TGCTGTTGACAGTGAGCGAACCGACATTGAGATATATTTATAGTGAAGCCACAGATGTATAAATATATCTCAATGTCGGTGTGCCTACTGCCTCGGA
**RASA1**	RASA1.2153	TAAATATATCTCAATGTCGGTG	TGCTGTTGACAGTGAGCGAACCGACATTGAGATATATTTATAGTGAAGCCACAGATGTATAAATATATCTCAATGTCGGTGTGCCTACTGCCTCGGA
**RASA1**	RASA1.3593	TAAAACTATACAGTTGAACTAA	TGCTGTTGACAGTGAGCGCTAGTTCAACTGTATAGTTTTATAGTGAAGCCACAGATGTATAAAACTATACAGTTGAACTAATGCCTACTGCCTCGGA
**RASA1**	RASA1.3593	TAAAACTATACAGTTGAACTAA	TGCTGTTGACAGTGAGCGCTAGTTCAACTGTATAGTTTTATAGTGAAGCCACAGATGTATAAAACTATACAGTTGAACTAATGCCTACTGCCTCGGA
**RASA2**	RASA2.1380	TTGTATTGAATAACTTGTCTAC	TGCTGTTGACAGTGAGCGATAGACAAGTTATTCAATACAATAGTGAAGCCACAGATGTATTGTATTGAATAACTTGTCTACTGCCTACTGCCTCGGA
**RASA2**	RASA2.1380	TTGTATTGAATAACTTGTCTAC	TGCTGTTGACAGTGAGCGATAGACAAGTTATTCAATACAATAGTGAAGCCACAGATGTATTGTATTGAATAACTTGTCTACTGCCTACTGCCTCGGA

Gene Symbol	shRNA Name	Guide	97mer
**RASA2**	RASA2.1884	TTGTTAAGCAGAACCATCGTTT	TGCTGTTGACAGTGAGCGCAACGATGGTTCTGCTTAACAATAGTGAAGCCACAGATGTATTGTTAAGCAGAACCATCGTTTTGCCTACTGCCTCGGA
**RASA2**	RASA2.1884	TTGTTAAGCAGAACCATCGTTT	TGCTGTTGACAGTGAGCGCAACGATGGTTCTGCTTAACAATAGTGAAGCCACAGATGTATTGTTAAGCAGAACCATCGTTTTGCCTACTGCCTCGGA
**RASA2**	RASA2.760	TTGTTCCACAAGTCGATCCTGA	TGCTGTTGACAGTGAGCGCCAGGATCGACTTGTGGAACAATAGTGAAGCCACAGATGTATTGTTCCACAAGTCGATCCTGATGCCTACTGCCTCGGA
**RASA2**	RASA2.760	TTGTTCCACAAGTCGATCCTGA	TGCTGTTGACAGTGAGCGCCAGGATCGACTTGTGGAACAATAGTGAAGCCACAGATGTATTGTTCCACAAGTCGATCCTGATGCCTACTGCCTCGGA
**RASA2**	RASA2.449	TTCAAGGTGAACTTTACCCTGA	TGCTGTTGACAGTGAGCGCCAGGGTAAAGTTCACCTTGAATAGTGAAGCCACAGATGTATTCAAGGTGAACTTTACCCTGATGCCTACTGCCTCGGA
**RASA2**	RASA2.449	TTCAAGGTGAACTTTACCCTGA	TGCTGTTGACAGTGAGCGCCAGGGTAAAGTTCACCTTGAATAGTGAAGCCACAGATGTATTCAAGGTGAACTTTACCCTGATGCCTACTGCCTCGGA
**RASA2**	RASA2.2057	TTCTACACAGTTATTTGCCTGG	TGCTGTTGACAGTGAGCGACAGGCAAATAACTGTGTAGAATAGTGAAGCCACAGATGTATTCTACACAGTTATTTGCCTGGTGCCTACTGCCTCGGA
**RASA2**	RASA2.2057	TTCTACACAGTTATTTGCCTGG	TGCTGTTGACAGTGAGCGACAGGCAAATAACTGTGTAGAATAGTGAAGCCACAGATGTATTCTACACAGTTATTTGCCTGGTGCCTACTGCCTCGGA
**RASA3**	RASA3.2532	TATTTCGTCTTCTTGAACTTGT	TGCTGTTGACAGTGAGCGCCAAGTTCAAGAAGACGAAATATAGTGAAGCCACAGATGTATATTTCGTCTTCTTGAACTTGTTGCCTACTGCCTCGGA
**RASA3**	RASA3.3525	TTACTTTGTATCAAAGAGCTAA	TGCTGTTGACAGTGAGCGCTAGCTCTTTGATACAAAGTAATAGTGAAGCCACAGATGTATTACTTTGTATCAAAGAGCTAATGCCTACTGCCTCGGA
**RASA3**	RASA3.276	TTTTCCACAATTTTGGTCCTGA	TGCTGTTGACAGTGAGCGCCAGGACCAAAATTGTGGAAAATAGTGAAGCCACAGATGTATTTTCCACAATTTTGGTCCTGATGCCTACTGCCTCGGA
**RASA3**	RASA3.4009	TTTGTGACAAGAAAGTTCCTAT	TGCTGTTGACAGTGAGCGCTAGGAACTTTCTTGTCACAAATAGTGAAGCCACAGATGTATTTGTGACAAGAAAGTTCCTATTGCCTACTGCCTCGGA
**RASA3**	RASA3.707	TAAAACACTTCATCGAACTGGG	TGCTGTTGACAGTGAGCGACCAGTTCGATGAAGTGTTTTATAGTGAAGCCACAGATGTATAAAACACTTCATCGAACTGGGTGCCTACTGCCTCGGA
**RASA4**	RASA4.4988	TTAATAATTCCAATCATCCTAT	TGCTGTTGACAGTGAGCGCTAGGATGATTGGAATTATTAATAGTGAAGCCACAGATGTATTAATAATTCCAATCATCCTATTGCCTACTGCCTCGGA
**RASA4**	RASA4.4126	TTTCGTGATCTCTTCTTCCCTT	TGCTGTTGACAGTGAGCGCAGGGAAGAAGAGATCACGAAATAGTGAAGCCACAGATGTATTTCGTGATCTCTTCTTCCCTTTGCCTACTGCCTCGGA
**RASA4**	RASA4.4422	TAAAAATACAAGAATTAGCTGG	TGCTGTTGACAGTGAGCGACAGCTAATTCTTGTATTTTTATAGTGAAGCCACAGATGTATAAAAATACAAGAATTAGCTGGTGCCTACTGCCTCGGA
**RASA4**	RASA4.3049	TTTGAGGAGAGAACAGCTGGTT	TGCTGTTGACAGTGAGCGCACCAGCTGTTCTCTCCTCAAATAGTGAAGCCACAGATGTATTTGAGGAGAGAACAGCTGGTTTGCCTACTGCCTCGGA
**RASA4**	RASA4.3459	TCAAGTTTTGCCAACCATCGGA	TGCTGTTGACAGTGAGCGCCCGATGGTTGGCAAAACTTGATAGTGAAGCCACAGATGTATCAAGTTTTGCCAACCATCGGATGCCTACTGCCTCGGA
**RASAL1**	RASAL1.3203	TTCTAGCAGACATTCTAGCGGG	TGCTGTTGACAGTGAGCGACCGCTAGAATGTCTGCTAGAATAGTGAAGCCACAGATGTATTCTAGCAGACATTCTAGCGGGTGCCTACTGCCTCGGA
**RASAL1**	RASAL1.3203	TTCTAGCAGACATTCTAGCGGG	TGCTGTTGACAGTGAGCGACCGCTAGAATGTCTGCTAGAATAGTGAAGCCACAGATGTATTCTAGCAGACATTCTAGCGGGTGCCTACTGCCTCGGA
**RASAL1**	RASAL1.3202	TCTAGCAGACATTCTAGCGGGT	TGCTGTTGACAGTGAGCGCCCCGCTAGAATGTCTGCTAGATAGTGAAGCCACAGATGTATCTAGCAGACATTCTAGCGGGTTGCCTACTGCCTCGGA
**RASAL1**	RASAL1.3202	TCTAGCAGACATTCTAGCGGGT	TGCTGTTGACAGTGAGCGCCCCGCTAGAATGTCTGCTAGATAGTGAAGCCACAGATGTATCTAGCAGACATTCTAGCGGGTTGCCTACTGCCTCGGA
**RASAL1**	RASAL1.3144	TTCTTGAACTACCTCATTGGAA	TGCTGTTGACAGTGAGCGCTCCAATGAGGTAGTTCAAGAATAGTGAAGCCACAGATGTATTCTTGAACTACCTCATTGGAATGCCTACTGCCTCGGA
**RASAL1**	RASAL1.3144	TTCTTGAACTACCTCATTGGAA	TGCTGTTGACAGTGAGCGCTCCAATGAGGTAGTTCAAGAATAGTGAAGCCACAGATGTATTCTTGAACTACCTCATTGGAATGCCTACTGCCTCGGA
**RASAL1**	RASAL1.2886	TTAGCAGACATGAAAACAGGAA	TGCTGTTGACAGTGAGCGCTCCTGTTTTCATGTCTGCTAATAGTGAAGCCACAGATGTATTAGCAGACATGAAAACAGGAATGCCTACTGCCTCGGA
**RASAL1**	RASAL1.2886	TTAGCAGACATGAAAACAGGAA	TGCTGTTGACAGTGAGCGCTCCTGTTTTCATGTCTGCTAATAGTGAAGCCACAGATGTATTAGCAGACATGAAAACAGGAATGCCTACTGCCTCGGA
**RASAL1**	RASAL1.1181	TAAACTGTTCCATCGACTTGGA	TGCTGTTGACAGTGAGCGCCCAAGTCGATGGAACAGTTTATAGTGAAGCCACAGATGTATAAACTGTTCCATCGACTTGGATGCCTACTGCCTCGGA

Gene Symbol	shRNA Name	Guide	97mer
**RASAL1**	RASAL1.1181	TAAACTGTTCCATCGACTTGGA	TGCTGTTGACAGTGAGCGCCCAAGTCGATGGAACAGTTTATAGTGAAGCCACAGATGTATAAACTGTTCCATCGACTTGGATGCCTACTGCCTCGGA
**RASAL2**	RASAL2.9009	TATAGTAGCAACTTTAGTCTGA	TGCTGTTGACAGTGAGCGCCAGACTAAAGTTGCTACTATATAGTGAAGCCACAGATGTATATAGTAGCAACTTTAGTCTGATGCCTACTGCCTCGGA
**RASAL2**	RASAL2.9009	TATAGTAGCAACTTTAGTCTGA	TGCTGTTGACAGTGAGCGCCAGACTAAAGTTGCTACTATATAGTGAAGCCACAGATGTATATAGTAGCAACTTTAGTCTGATGCCTACTGCCTCGGA
**RASAL2**	RASAL2.6025	TAACTTAACATTTAACATCTAT	TGCTGTTGACAGTGAGCGCTAGATGTTAAATGTTAAGTTATAGTGAAGCCACAGATGTATAACTTAACATTTAACATCTATTGCCTACTGCCTCGGA
**RASAL2**	RASAL2.6025	TAACTTAACATTTAACATCTAT	TGCTGTTGACAGTGAGCGCTAGATGTTAAATGTTAAGTTATAGTGAAGCCACAGATGTATAACTTAACATTTAACATCTATTGCCTACTGCCTCGGA
**RASAL2**	RASAL2.3477	TTGTGAAGTAGCAACATTCTGT	TGCTGTTGACAGTGAGCGCCAGAATGTTGCTACTTCACAATAGTGAAGCCACAGATGTATTGTGAAGTAGCAACATTCTGTTGCCTACTGCCTCGGA
**RASAL2**	RASAL2.3477	TTGTGAAGTAGCAACATTCTGT	TGCTGTTGACAGTGAGCGCCAGAATGTTGCTACTTCACAATAGTGAAGCCACAGATGTATTGTGAAGTAGCAACATTCTGTTGCCTACTGCCTCGGA
**RASAL2**	RASAL2.8696	TATCATTGTAAAATAGTCGTGT	TGCTGTTGACAGTGAGCGCCACGACTATTTTACAATGATATAGTGAAGCCACAGATGTATATCATTGTAAAATAGTCGTGTTGCCTACTGCCTCGGA
**RASAL2**	RASAL2.8696	TATCATTGTAAAATAGTCGTGT	TGCTGTTGACAGTGAGCGCCACGACTATTTTACAATGATATAGTGAAGCCACAGATGTATATCATTGTAAAATAGTCGTGTTGCCTACTGCCTCGGA
**RASAL2**	RASAL2.459	TTAGGTTGAACTGTCCTGCGAA	TGCTGTTGACAGTGAGCGCTCGCAGGACAGTTCAACCTAATAGTGAAGCCACAGATGTATTAGGTTGAACTGTCCTGCGAATGCCTACTGCCTCGGA
**RASAL2**	RASAL2.459	TTAGGTTGAACTGTCCTGCGAA	TGCTGTTGACAGTGAGCGCTCGCAGGACAGTTCAACCTAATAGTGAAGCCACAGATGTATTAGGTTGAACTGTCCTGCGAATGCCTACTGCCTCGGA
**RASGRF1**	RASGRF1.2658	TACATTACTATATTCCTGCTAG	TGCTGTTGACAGTGAGCGATAGCAGGAATATAGTAATGTATAGTGAAGCCACAGATGTATACATTACTATATTCCTGCTAGTGCCTACTGCCTCGGA
**RASGRF1**	RASGRF1.3029	TATGGTGTCCTTGACACTCTGG	TGCTGTTGACAGTGAGCGACAGAGTGTCAAGGACACCATATAGTGAAGCCACAGATGTATATGGTGTCCTTGACACTCTGGTGCCTACTGCCTCGGA
**RASGRF1**	RASGRF1.1738	TACAGTATCATCTAGCACATGT	TGCTGTTGACAGTGAGCGCCATGTGCTAGATGATACTGTATAGTGAAGCCACAGATGTATACAGTATCATCTAGCACATGTTGCCTACTGCCTCGGA
**RASGRF1**	RASGRF1.2342	TAATCCTAGAGCAAGGAGCGAC	TGCTGTTGACAGTGAGCGATCGCTCCTTGCTCTAGGATTATAGTGAAGCCACAGATGTATAATCCTAGAGCAAGGAGCGACTGCCTACTGCCTCGGA
**RASGRF1**	RASGRF1.2771	TTGTCTGTCTGTTGTAGTGGAG	TGCTGTTGACAGTGAGCGATCCACTACAACAGACAGACAATAGTGAAGCCACAGATGTATTGTCTGTCTGTTGTAGTGGAGTGCCTACTGCCTCGGA
**RBM14**	RBM14.1421	TTTAGCTATCATGTCTTCCTGG	TGCTGTTGACAGTGAGCGACAGGAAGACATGATAGCTAAATAGTGAAGCCACAGATGTATTTAGCTATCATGTCTTCCTGGTGCCTACTGCCTCGGA
**RBM14**	RBM14.3781	TAAATCTAGACATAAAACCCAG	TGCTGTTGACAGTGAGCGATGGGTTTTATGTCTAGATTTATAGTGAAGCCACAGATGTATAAATCTAGACATAAAACCCAGTGCCTACTGCCTCGGA
**RBM14**	RBM14.1281	TTTTCTAACACCACCTACTTGG	TGCTGTTGACAGTGAGCGACAAGTAGGTGGTGTTAGAAAATAGTGAAGCCACAGATGTATTTTCTAACACCACCTACTTGGTGCCTACTGCCTCGGA
**RBM14**	RBM14.1437	TTAGCTATCATGTCTTCCTGGA	TGCTGTTGACAGTGAGCGCCCAGGAAGACATGATAGCTAATAGTGAAGCCACAGATGTATTAGCTATCATGTCTTCCTGGATGCCTACTGCCTCGGA
**RBM14**	RBM14.1332	TTGTAGTCCATACAAAAGGGAA	TGCTGTTGACAGTGAGCGCTCCCTTTTGTATGGACTACAATAGTGAAGCCACAGATGTATTGTAGTCCATACAAAAGGGAATGCCTACTGCCTCGGA
**RNF138**	RNF138.759	TTACATTAGTGAAACATCTTTT	TGCTGTTGACAGTGAGCGCAAAGATGTTTCACTAATGTAATAGTGAAGCCACAGATGTATTACATTAGTGAAACATCTTTTTGCCTACTGCCTCGGA
**RNF138**	RNF138.2339	TAACTTGACATATTGGATGTAT	TGCTGTTGACAGTGAGCGCTACATCCAATATGTCAAGTTATAGTGAAGCCACAGATGTATAACTTGACATATTGGATGTATTGCCTACTGCCTCGGA
**RNF138**	RNF138.2861	TACATCTCAAATAAGACTCTGT	TGCTGTTGACAGTGAGCGCCAGAGTCTTATTTGAGATGTATAGTGAAGCCACAGATGTATACATCTCAAATAAGACTCTGTTGCCTACTGCCTCGGA
**RNF138**	RNF138.481	TTGGGTTTCTTCATCTAGCTGA	TGCTGTTGACAGTGAGCGCCAGCTAGATGAAGAAACCCAATAGTGAAGCCACAGATGTATTGGGTTTCTTCATCTAGCTGATGCCTACTGCCTCGGA
**RNF138**	RNF138.2166	TAAAATGACAGAACAAAGGCGT	TGCTGTTGACAGTGAGCGCCGCCTTTGTTCTGTCATTTTATAGTGAAGCCACAGATGTATAAAATGACAGAACAAAGGCGTTGCCTACTGCCTCGGA
**RUVBL1**	RUVBL1.487	TTCACCTTCATAAACTTCCTTG	TGCTGTTGACAGTGAGCGAAAGGAAGTTTATGAAGGTGAATAGTGAAGCCACAGATGTATTCACCTTCATAAACTTCCTTGTGCCTACTGCCTCGGA

Gene Symbol	shRNA Name	Guide	97mer
**RUVBL1**	RUVBL1.907	TTTGTCTGTGATTTCTGTCTTC	TGCTGTTGACAGTGAGCGAAAGACAGAAATCACAGACAAATAGTGAAGCCACAGATGTATTTGTCTGTGATTTCTGTCTTCTGCCTACTGCCTCGGA
**RUVBL1**	RUVBL1.1673	TTATAGAAAACACACCAGGTAA	TGCTGTTGACAGTGAGCGCTACCTGGTGTGTTTTCTATAATAGTGAAGCCACAGATGTATTATAGAAAACACACCAGGTAATGCCTACTGCCTCGGA
**RUVBL1**	RUVBL1.989	TCAACAAACAGCACACCCGGGA	TGCTGTTGACAGTGAGCGCCCCGGGTGTGCTGTTTGTTGATAGTGAAGCCACAGATGTATCAACAAACAGCACACCCGGGATGCCTACTGCCTCGGA
**RUVBL1**	RUVBL1.1338	TTTAGCAAGCAAGTTGGCCGGG	TGCTGTTGACAGTGAGCGACCGGCCAACTTGCTTGCTAAATAGTGAAGCCACAGATGTATTTAGCAAGCAAGTTGGCCGGGTGCCTACTGCCTCGGA
**SFPQ**	SFPQ.2323	TATTAGGTCAATAAACTGCTAA	TGCTGTTGACAGTGAGCGCTAGCAGTTTATTGACCTAATATAGTGAAGCCACAGATGTATATTAGGTCAATAAACTGCTAATGCCTACTGCCTCGGA
**SFPQ**	SFPQ.1022	TAGTCTTTTGAATTCATCCTCC	TGCTGTTGACAGTGAGCGAGAGGATGAATTCAAAAGACTATAGTGAAGCCACAGATGTATAGTCTTTTGAATTCATCCTCCTGCCTACTGCCTCGGA
**SFPQ**	SFPQ.1062	TTGTTGATAAAAACTTCTCCTG	TGCTGTTGACAGTGAGCGAAGGAGAAGTTTTTATCAACAATAGTGAAGCCACAGATGTATTGTTGATAAAAACTTCTCCTGTGCCTACTGCCTCGGA
**SFPQ**	SFPQ.1734	TTGTGAAGTTCTTCCATGCGTC	TGCTGTTGACAGTGAGCGAACGCATGGAAGAACTTCACAATAGTGAAGCCACAGATGTATTGTGAAGTTCTTCCATGCGTCTGCCTACTGCCTCGGA
**SFPQ**	SFPQ.1059	TTGATAAAAACTTCTCCTGGTT	TGCTGTTGACAGTGAGCGCACCAGGAGAAGTTTTTATCAATAGTGAAGCCACAGATGTATTGATAAAAACTTCTCCTGGTTTGCCTACTGCCTCGGA
**SKI**	SKI.3286	TTGACTGCTCTATAAATCGGTA	TGCTGTTGACAGTGAGCGCACCGATTTATAGAGCAGTCAATAGTGAAGCCACAGATGTATTGACTGCTCTATAAATCGGTATGCCTACTGCCTCGGA
**SKI**	SKI.4039	TTGCATAACAAAACCGACCCTT	TGCTGTTGACAGTGAGCGCAGGGTCGGTTTTGTTATGCAATAGTGAAGCCACAGATGTATTGCATAACAAAACCGACCCTTTGCCTACTGCCTCGGA
**SKI**	SKI.5673	TTATTGTACAATGTCATCTGTT	TGCTGTTGACAGTGAGCGCACAGATGACATTGTACAATAATAGTGAAGCCACAGATGTATTATTGTACAATGTCATCTGTTTGCCTACTGCCTCGGA
**SKI**	SKI.4968	TACATGTATAATACAACGGTGA	TGCTGTTGACAGTGAGCGCCACCGTTGTATTATACATGTATAGTGAAGCCACAGATGTATACATGTATAATACAACGGTGATGCCTACTGCCTCGGA
**SKI**	SKI.5667	TACAATGTCATCTGTTTCGGGG	TGCTGTTGACAGTGAGCGACCCGAAACAGATGACATTGTATAGTGAAGCCACAGATGTATACAATGTCATCTGTTTCGGGGTGCCTACTGCCTCGGA
**SMAD6**	SMAD6.865	TACATTGTAAAAATGACTCCAT	TGCTGTTGACAGTGAGCGCTGGAGTCATTTTTACAATGTATAGTGAAGCCACAGATGTATACATTGTAAAAATGACTCCATTGCCTACTGCCTCGGA
**SMAD6**	SMAD6.941	TATTGAAAGAATTATAAGCCAA	TGCTGTTGACAGTGAGCGCTGGCTTATAATTCTTTCAATATAGTGAAGCCACAGATGTATATTGAAAGAATTATAAGCCAATGCCTACTGCCTCGGA
**SMAD6**	SMAD6.82	TTTCAGTGTAAGACAATGTGGA	TGCTGTTGACAGTGAGCGCCCACATTGTCTTACACTGAAATAGTGAAGCCACAGATGTATTTCAGTGTAAGACAATGTGGATGCCTACTGCCTCGGA
**SMAD6**	SMAD6.81	TTCAGTGTAAGACAATGTGGAA	TGCTGTTGACAGTGAGCGCTCCACATTGTCTTACACTGAATAGTGAAGCCACAGATGTATTCAGTGTAAGACAATGTGGAATGCCTACTGCCTCGGA
**SMAD6**	SMAD6.1061	TATCTCAAAAACCATACACCAA	TGCTGTTGACAGTGAGCGCTGGTGTATGGTTTTTGAGATATAGTGAAGCCACAGATGTATATCTCAAAAACCATACACCAATGCCTACTGCCTCGGA
**SMAD7**	SMAD7.390	TTGTGTACCAACAGCGTCCTGG	TGCTGTTGACAGTGAGCGACAGGACGCTGTTGGTACACAATAGTGAAGCCACAGATGTATTGTGTACCAACAGCGTCCTGGTGCCTACTGCCTCGGA
**SMAD7**	SMAD7.1703	TTGAGCTAAGAACAGTGTCGAA	TGCTGTTGACAGTGAGCGCTCGACACTGTTCTTAGCTCAATAGTGAAGCCACAGATGTATTGAGCTAAGAACAGTGTCGAATGCCTACTGCCTCGGA
**SMAD7**	SMAD7.1953	TTCTTGTTTATACACATTGCAC	TGCTGTTGACAGTGAGCGATGCAATGTGTATAAACAAGAATAGTGAAGCCACAGATGTATTCTTGTTTATACACATTGCACTGCCTACTGCCTCGGA
**SMAD7**	SMAD7.2099	TTTAATGGAACATAAACTCCTT	TGCTGTTGACAGTGAGCGCAGGAGTTTATGTTCCATTAAATAGTGAAGCCACAGATGTATTTAATGGAACATAAACTCCTTTGCCTACTGCCTCGGA
**SMAD7**	SMAD7.239	TTGTTGTCCGAATTGAGCTGTC	TGCTGTTGACAGTGAGCGAACAGCTCAATTCGGACAACAATAGTGAAGCCACAGATGTATTGTTGTCCGAATTGAGCTGTCTGCCTACTGCCTCGGA
**SMC3**	SMC3.1337	TTTCTTGTCATTAATAGCCTGA	TGCTGTTGACAGTGAGCGCCAGGCTATTAATGACAAGAAATAGTGAAGCCACAGATGTATTTCTTGTCATTAATAGCCTGATGCCTACTGCCTCGGA
**SMC3**	SMC3.1442	TTTGACTTCATTAAGATCCTGG	TGCTGTTGACAGTGAGCGACAGGATCTTAATGAAGTCAAATAGTGAAGCCACAGATGTATTTGACTTCATTAAGATCCTGGTGCCTACTGCCTCGGA
**SMC3**	SMC3.860	TTCTCCACTAGTCTCTCGCTTA	TGCTGTTGACAGTGAGCGCAAGCGAGAGACTAGTGGAGAATAGTGAAGCCACAGATGTATTCTCCACTAGTCTCTCGCTTATGCCTACTGCCTCGGA

Gene Symbol	shRNA Name	Guide	97mer
**SMC3**	SMC3.905	TTTATCTCTTGCATCCTGCTGA	TGCTGTTGACAGTGAGCGCCAGCAGGATGCAAGAGATAAATAGTGAAGCCACAGATGTATTTATCTCTTGCATCCTGCTGATGCCTACTGCCTCGGA
**SMC3**	SMC3.3106	TAACTTTTCTTTCTGCTCGGAG	TGCTGTTGACAGTGAGCGATCCGAGCAGAAAGAAAAGTTATAGTGAAGCCACAGATGTATAACTTTTCTTTCTGCTCGGAGTGCCTACTGCCTCGGA
**SMURF2**	SMURF2.1967	TAAACTGTTGTGAAGATCCGGA	TGCTGTTGACAGTGAGCGCCCGGATCTTCACAACAGTTTATAGTGAAGCCACAGATGTATAAACTGTTGTGAAGATCCGGATGCCTACTGCCTCGGA
**SMURF2**	SMURF2.1494	TTTCTGAACCAGGTCTCGCTTG	TGCTGTTGACAGTGAGCGAAAGCGAGACCTGGTTCAGAAATAGTGAAGCCACAGATGTATTTCTGAACCAGGTCTCGCTTGTGCCTACTGCCTCGGA
**SMURF2**	SMURF2.3699	TTTAGAGCAAAATTCTTCCTTT	TGCTGTTGACAGTGAGCGCAAGGAAGAATTTTGCTCTAAATAGTGAAGCCACAGATGTATTTAGAGCAAAATTCTTCCTTTTGCCTACTGCCTCGGA
**SMURF2**	SMURF2.3622	TTAATGGAAACTTACACTGTAG	TGCTGTTGACAGTGAGCGATACAGTGTAAGTTTCCATTAATAGTGAAGCCACAGATGTATTAATGGAAACTTACACTGTAGTGCCTACTGCCTCGGA
**SMURF2**	SMURF2.633	TTTCTTATGGATCTTCTTGTGA	TGCTGTTGACAGTGAGCGCCACAAGAAGATCCATAAGAAATAGTGAAGCCACAGATGTATTTCTTATGGATCTTCTTGTGATGCCTACTGCCTCGGA
**SNRPB**	SNRPB.928	TAAAAGGACTATGTACAGCCTT	TGCTGTTGACAGTGAGCGCAGGCTGTACATAGTCCTTTTATAGTGAAGCCACAGATGTATAAAAGGACTATGTACAGCCTTTGCCTACTGCCTCGGA
**SNRPB**	SNRPB.20	TAGAAACCTACTTCCGGTCCAG	TGCTGTTGACAGTGAGCGATGGACCGGAAGTAGGTTTCTATAGTGAAGCCACAGATGTATAGAAACCTACTTCCGGTCCAGTGCCTACTGCCTCGGA
**SNRPB**	SNRPB.391	TCTACTGTCATTGAGACCAGAT	TGCTGTTGACAGTGAGCGCTCTGGTCTCAATGACAGTAGATAGTGAAGCCACAGATGTATCTACTGTCATTGAGACCAGATTGCCTACTGCCTCGGA
**SNRPB**	SNRPB.292	TTTCTGAACTCATCACAGTCAC	TGCTGTTGACAGTGAGCGATGACTGTGATGAGTTCAGAAATAGTGAAGCCACAGATGTATTTCTGAACTCATCACAGTCACTGCCTACTGCCTCGGA
**SNRPB**	SNRPB.329	TTCCCTTTCTGCTTGTTTGGAG	TGCTGTTGACAGTGAGCGATCCAAACAAGCAGAAAGGGAATAGTGAAGCCACAGATGTATTCCCTTTCTGCTTGTTTGGAGTGCCTACTGCCTCGGA
**SNRPD1**	SNRPD1.1118	TATATTGTTAAGTTTAGCCTAA	TGCTGTTGACAGTGAGCGCTAGGCTAAACTTAACAATATATAGTGAAGCCACAGATGTATATATTGTTAAGTTTAGCCTAATGCCTACTGCCTCGGA
**SNRPD1**	SNRPD1.1049	TAGAACTGTAGCCTAACTGGAC	TGCTGTTGACAGTGAGCGATCCAGTTAGGCTACAGTTCTATAGTGAAGCCACAGATGTATAGAACTGTAGCCTAACTGGACTGCCTACTGCCTCGGA
**SNRPD1**	SNRPD1.582	TAAATAGACAGCTCTGTCCCAC	TGCTGTTGACAGTGAGCGATGGGACAGAGCTGTCTATTTATAGTGAAGCCACAGATGTATAAATAGACAGCTCTGTCCCACTGCCTACTGCCTCGGA
**SNRPD1**	SNRPD1.1061	TTTCTCAGTACATAGAACTGTA	TGCTGTTGACAGTGAGCGCACAGTTCTATGTACTGAGAAATAGTGAAGCCACAGATGTATTTCTCAGTACATAGAACTGTATGCCTACTGCCTCGGA
**SNRPD1**	SNRPD1.1469	TAACTGATAATCTAGTGACCAT	TGCTGTTGACAGTGAGCGCTGGTCACTAGATTATCAGTTATAGTGAAGCCACAGATGTATAACTGATAATCTAGTGACCATTGCCTACTGCCTCGGA
**SOWAHC**	SOWAHC.2451	TTAATGTACCTTAACACTGGTG	TGCTGTTGACAGTGAGCGAACCAGTGTTAAGGTACATTAATAGTGAAGCCACAGATGTATTAATGTACCTTAACACTGGTGTGCCTACTGCCTCGGA
**SOWAHC**	SOWAHC.3222	TTAGGTTTCAGCAACTACGCGA	TGCTGTTGACAGTGAGCGCCGCGTAGTTGCTGAAACCTAATAGTGAAGCCACAGATGTATTAGGTTTCAGCAACTACGCGATGCCTACTGCCTCGGA
**SPRED1**	SPRED1.5564	TTCAACACAAGAAATATCCTTA	TGCTGTTGACAGTGAGCGCAAGGATATTTCTTGTGTTGAATAGTGAAGCCACAGATGTATTCAACACAAGAAATATCCTTATGCCTACTGCCTCGGA
**SPRED1**	SPRED1.5123	TAAAATACCAAAACTTAGCTGG	TGCTGTTGACAGTGAGCGACAGCTAAGTTTTGGTATTTTATAGTGAAGCCACAGATGTATAAAATACCAAAACTTAGCTGGTGCCTACTGCCTCGGA
**SPRED1**	SPRED1.1060	TATTCTGACAATCTCATCCTCA	TGCTGTTGACAGTGAGCGCGAGGATGAGATTGTCAGAATATAGTGAAGCCACAGATGTATATTCTGACAATCTCATCCTCATGCCTACTGCCTCGGA
**SPRED1**	SPRED1.6831	TATTACTGTCCTAATAACCTTT	TGCTGTTGACAGTGAGCGCAAGGTTATTAGGACAGTAATATAGTGAAGCCACAGATGTATATTACTGTCCTAATAACCTTTTGCCTACTGCCTCGGA
**SPRED1**	SPRED1.5651	TACACTTGCTTAATGCACCTTT	TGCTGTTGACAGTGAGCGCAAGGTGCATTAAGCAAGTGTATAGTGAAGCCACAGATGTATACACTTGCTTAATGCACCTTTTGCCTACTGCCTCGGA
**SPRED2**	SPRED2.418	TAGAAGAACTGTCTGTAGCTGT	TGCTGTTGACAGTGAGCGCCAGCTACAGACAGTTCTTCTATAGTGAAGCCACAGATGTATAGAAGAACTGTCTGTAGCTGTTGCCTACTGCCTCGGA
**SPRED2**	SPRED2.1853	TAAAGATAGAAACTGCAGCTTA	TGCTGTTGACAGTGAGCGCAAGCTGCAGTTTCTATCTTTATAGTGAAGCCACAGATGTATAAAGATAGAAACTGCAGCTTATGCCTACTGCCTCGGA
**SPRED2**	SPRED2.2948	TAATCTACTAAAGAAAATCGAT	TGCTGTTGACAGTGAGCGCTCGATTTTCTTTAGTAGATTATAGTGAAGCCACAGATGTATAATCTACTAAAGAAAATCGATTGCCTACTGCCTCGGA

Gene Symbol	shRNA Name	Guide	97mer
**SPRED2**	SPRED2.1588	TTGCAGACTCCTTTGAACTGGA	TGCTGTTGACAGTGAGCGCCCAGTTCAAAGGAGTCTGCAATAGTGAAGCCACAGATGTATTGCAGACTCCTTTGAACTGGATGCCTACTGCCTCGGA
**SPRED2**	SPRED2.1496	TAGTACAGGAGTCTGTGGCGAA	TGCTGTTGACAGTGAGCGCTCGCCACAGACTCCTGTACTATAGTGAAGCCACAGATGTATAGTACAGGAGTCTGTGGCGAATGCCTACTGCCTCGGA
**SPRY1**	SPRY1.2179	TAACATATGTACTTTCTTCGTG	TGCTGTTGACAGTGAGCGAACGAAGAAAGTACATATGTTATAGTGAAGCCACAGATGTATAACATATGTACTTTCTTCGTGTGCCTACTGCCTCGGA
**SPRY1**	SPRY1.587	TTCACAAATGAACTTGTGCTGT	TGCTGTTGACAGTGAGCGCCAGCACAAGTTCATTTGTGAATAGTGAAGCCACAGATGTATTCACAAATGAACTTGTGCTGTTGCCTACTGCCTCGGA
**SPRY1**	SPRY1.718	TTGACTAAGCACATGCAGGTTC	TGCTGTTGACAGTGAGCGAAACCTGCATGTGCTTAGTCAATAGTGAAGCCACAGATGTATTGACTAAGCACATGCAGGTTCTGCCTACTGCCTCGGA
**SPRY1**	SPRY1.311	TTATTATTCACATTAATTGGTA	TGCTGTTGACAGTGAGCGCACCAATTAATGTGAATAATAATAGTGAAGCCACAGATGTATTATTATTCACATTAATTGGTATGCCTACTGCCTCGGA
**SPRY1**	SPRY1.137	TCATAGTCTAATCTCTGACGGC	TGCTGTTGACAGTGAGCGACCGTCAGAGATTAGACTATGATAGTGAAGCCACAGATGTATCATAGTCTAATCTCTGACGGCTGCCTACTGCCTCGGA
**SPRY2**	SPRY2.328	TAGAACACATCTGAACTCCGTG	TGCTGTTGACAGTGAGCGAACGGAGTTCAGATGTGTTCTATAGTGAAGCCACAGATGTATAGAACACATCTGAACTCCGTGTGCCTACTGCCTCGGA
**SPRY2**	SPRY2.1747	TAGTATAATATTTTGTGTCTGT	TGCTGTTGACAGTGAGCGCCAGACACAAAATATTATACTATAGTGAAGCCACAGATGTATAGTATAATATTTTGTGTCTGTTGCCTACTGCCTCGGA
**SPRY2**	SPRY2.2060	TATATTGGACATATGCATCTGT	TGCTGTTGACAGTGAGCGCCAGATGCATATGTCCAATATATAGTGAAGCCACAGATGTATATATTGGACATATGCATCTGTTGCCTACTGCCTCGGA
**SPRY2**	SPRY2.1553	TTAGCTTATGCAATACATGGGT	TGCTGTTGACAGTGAGCGCCCCATGTATTGCATAAGCTAATAGTGAAGCCACAGATGTATTAGCTTATGCAATACATGGGTTGCCTACTGCCTCGGA
**SPRY2**	SPRY2.329	TTAGAACACATCTGAACTCCGT	TGCTGTTGACAGTGAGCGCCGGAGTTCAGATGTGTTCTAATAGTGAAGCCACAGATGTATTAGAACACATCTGAACTCCGTTGCCTACTGCCTCGGA
**SPRY3**	SPRY3.6580	TTTAACAGCAGCAACACTGGTA	TGCTGTTGACAGTGAGCGCACCAGTGTTGCTGCTGTTAAATAGTGAAGCCACAGATGTATTTAACAGCAGCAACACTGGTATGCCTACTGCCTCGGA
**SPRY3**	SPRY3.6604	TTCCTTATCTTGTCCATCCTGG	TGCTGTTGACAGTGAGCGACAGGATGGACAAGATAAGGAATAGTGAAGCCACAGATGTATTCCTTATCTTGTCCATCCTGGTGCCTACTGCCTCGGA
**SPRY3**	SPRY3.7990	TTGCATATTATAATCATCTGGG	TGCTGTTGACAGTGAGCGACCAGATGATTATAATATGCAATAGTGAAGCCACAGATGTATTGCATATTATAATCATCTGGGTGCCTACTGCCTCGGA
**SPRY3**	SPRY3.2842	TTCTTTTCCACACTTGAGCTTC	TGCTGTTGACAGTGAGCGAAAGCTCAAGTGTGGAAAAGAATAGTGAAGCCACAGATGTATTCTTTTCCACACTTGAGCTTCTGCCTACTGCCTCGGA
**SPRY3**	SPRY3.7497	TTAGGTGAGAGAAACACACTGA	TGCTGTTGACAGTGAGCGCCAGTGTGTTTCTCTCACCTAATAGTGAAGCCACAGATGTATTAGGTGAGAGAAACACACTGATGCCTACTGCCTCGGA
**SPRY4**	SPRY4.4393	TTCAAGGTGACAGTCATCCGGG	TGCTGTTGACAGTGAGCGACCGGATGACTGTCACCTTGAATAGTGAAGCCACAGATGTATTCAAGGTGACAGTCATCCGGGTGCCTACTGCCTCGGA
**SPRY4**	SPRY4.2099	TAAAACACCGTTAGCATCCGGT	TGCTGTTGACAGTGAGCGCCCGGATGCTAACGGTGTTTTATAGTGAAGCCACAGATGTATAAAACACCGTTAGCATCCGGTTGCCTACTGCCTCGGA
**SPRY4**	SPRY4.2909	TAAGATGTTACTACTAGTCTTC	TGCTGTTGACAGTGAGCGAAAGACTAGTAGTAACATCTTATAGTGAAGCCACAGATGTATAAGATGTTACTACTAGTCTTCTGCCTACTGCCTCGGA
**SPRY4**	SPRY4.4659	TTGTAACATGAAACCAAGCTGT	TGCTGTTGACAGTGAGCGCCAGCTTGGTTTCATGTTACAATAGTGAAGCCACAGATGTATTGTAACATGAAACCAAGCTGTTGCCTACTGCCTCGGA
**SPRY4**	SPRY4.2067	TATAAAAACATAATGACTGGAT	TGCTGTTGACAGTGAGCGCTCCAGTCATTATGTTTTTATATAGTGAAGCCACAGATGTATATAAAAACATAATGACTGGATTGCCTACTGCCTCGGA
**SRSF1**	SRSF1.3300	TTAACATTTAATACTTACCTTT	TGCTGTTGACAGTGAGCGCAAGGTAAGTATTAAATGTTAATAGTGAAGCCACAGATGTATTAACATTTAATACTTACCTTTTGCCTACTGCCTCGGA
**SRSF1**	SRSF1.4112	TAAACTTACACTAAGTACTTAG	TGCTGTTGACAGTGAGCGATAAGTACTTAGTGTAAGTTTATAGTGAAGCCACAGATGTATAAACTTACACTAAGTACTTAGTGCCTACTGCCTCGGA
**SRSF1**	SRSF1.3678	TAATCTTGTGCACTCTTCCCTT	TGCTGTTGACAGTGAGCGCAGGGAAGAGTGCACAAGATTATAGTGAAGCCACAGATGTATAATCTTGTGCACTCTTCCCTTTGCCTACTGCCTCGGA
**SRSF1**	SRSF1.1216	TTACACAATATCACAGTCTGAA	TGCTGTTGACAGTGAGCGCTCAGACTGTGATATTGTGTAATAGTGAAGCCACAGATGTATTACACAATATCACAGTCTGAATGCCTACTGCCTCGGA
**SRSF1**	SRSF1.2656	TTTATCTCCAGGTCCTCGCTTT	TGCTGTTGACAGTGAGCGCAAGCGAGGACCTGGAGATAAATAGTGAAGCCACAGATGTATTTATCTCCAGGTCCTCGCTTTTGCCTACTGCCTCGGA

Gene Symbol	shRNA Name	Guide	97mer
**SRSF10**	SRSF10.613	TAGTTGTAATCAAAAGACCGAC	TGCTGTTGACAGTGAGCGATCGGTCTTTTGATTACAACTATAGTGAAGCCACAGATGTATAGTTGTAATCAAAAGACCGACTGCCTACTGCCTCGGA
**SRSF10**	SRSF10.371	TTGAACATAAGCAAATCCTCTT	TGCTGTTGACAGTGAGCGCAGAGGATTTGCTTATGTTCAATAGTGAAGCCACAGATGTATTGAACATAAGCAAATCCTCTTTGCCTACTGCCTCGGA
**SRSF10**	SRSF10.567	TTCGGCTTCTAGAACGTCTGTA	TGCTGTTGACAGTGAGCGCACAGACGTTCTAGAAGCCGAATAGTGAAGCCACAGATGTATTCGGCTTCTAGAACGTCTGTATGCCTACTGCCTCGGA
**SRSF10**	SRSF10.319	TACACATCAACTATAGGACCAT	TGCTGTTGACAGTGAGCGCTGGTCCTATAGTTGATGTGTATAGTGAAGCCACAGATGTATACACATCAACTATAGGACCATTGCCTACTGCCTCGGA
**SRSF10**	SRSF10.571	TAACTTCGGCTTCTAGAACGTC	TGCTGTTGACAGTGAGCGAACGTTCTAGAAGCCGAAGTTATAGTGAAGCCACAGATGTATAACTTCGGCTTCTAGAACGTCTGCCTACTGCCTCGGA
**SRSF2**	SRSF2.1937	TTAAACTACAGAAACAATGGTT	TGCTGTTGACAGTGAGCGCACCATTGTTTCTGTAGTTTAATAGTGAAGCCACAGATGTATTAAACTACAGAAACAATGGTTTGCCTACTGCCTCGGA
**SRSF2**	SRSF2.1488	TTGTGTTTGATAAACAATCCTT	TGCTGTTGACAGTGAGCGCAGGATTGTTTATCAAACACAATAGTGAAGCCACAGATGTATTGTGTTTGATAAACAATCCTTTGCCTACTGCCTCGGA
**SRSF2**	SRSF2.1919	TAAACTACAGAAACAATGGTTA	TGCTGTTGACAGTGAGCGCAACCATTGTTTCTGTAGTTTATAGTGAAGCCACAGATGTATAAACTACAGAAACAATGGTTATGCCTACTGCCTCGGA
**SRSF2**	SRSF2.1545	TCAACTGCTACACAACTGCGCC	TGCTGTTGACAGTGAGCGAGCGCAGTTGTGTAGCAGTTGATAGTGAAGCCACAGATGTATCAACTGCTACACAACTGCGCCTGCCTACTGCCTCGGA
**SRSF2**	SRSF2.1723	TTATTTATATGCAAGGCCCGGG	TGCTGTTGACAGTGAGCGACCGGGCCTTGCATATAAATAATAGTGAAGCCACAGATGTATTATTTATATGCAAGGCCCGGGTGCCTACTGCCTCGGA
**SRSF3**	SRSF3.2297	TTAACTTTAAGGCTGAACCTTC	TGCTGTTGACAGTGAGCGAAAGGTTCAGCCTTAAAGTTAATAGTGAAGCCACAGATGTATTAACTTTAAGGCTGAACCTTCTGCCTACTGCCTCGGA
**SRSF3**	SRSF3.1983	TTACATTTGAACCATATTGTGA	TGCTGTTGACAGTGAGCGCCACAATATGGTTCAAATGTAATAGTGAAGCCACAGATGTATTACATTTGAACCATATTGTGATGCCTACTGCCTCGGA
**SRSF3**	SRSF3.774	TTGAACAGCTAAAACATCTTAA	TGCTGTTGACAGTGAGCGCTAAGATGTTTTAGCTGTTCAATAGTGAAGCCACAGATGTATTGAACAGCTAAAACATCTTAATGCCTACTGCCTCGGA
**SRSF3**	SRSF3.1204	TTCTTGAAACTTAACATTCTAT	TGCTGTTGACAGTGAGCGCTAGAATGTTAAGTTTCAAGAATAGTGAAGCCACAGATGTATTCTTGAAACTTAACATTCTATTGCCTACTGCCTCGGA
**SRSF3**	SRSF3.941	TAGAGGTTTATTATCAGTCTGT	TGCTGTTGACAGTGAGCGCCAGACTGATAATAAACCTCTATAGTGAAGCCACAGATGTATAGAGGTTTATTATCAGTCTGTTGCCTACTGCCTCGGA
**SSRP1**	SSRP1.2236	TTGCTCTTGAAGCTCTCGCTTA	TGCTGTTGACAGTGAGCGCAAGCGAGAGCTTCAAGAGCAATAGTGAAGCCACAGATGTATTGCTCTTGAAGCTCTCGCTTATGCCTACTGCCTCGGA
**SSRP1**	SSRP1.1950	TCTGACTTGATCTTCTCTCGGC	TGCTGTTGACAGTGAGCGACCGAGAGAAGATCAAGTCAGATAGTGAAGCCACAGATGTATCTGACTTGATCTTCTCTCGGCTGCCTACTGCCTCGGA
**SSRP1**	SSRP1.2168	TTTCTTTTCCATCTTTACCTTT	TGCTGTTGACAGTGAGCGCAAGGTAAAGATGGAAAAGAAATAGTGAAGCCACAGATGTATTTCTTTTCCATCTTTACCTTTTGCCTACTGCCTCGGA
**SSRP1**	SSRP1.2149	TTTACTTTCTTCTTCTTCTTTG	TGCTGTTGACAGTGAGCGAAAAGAAGAAGAAGAAAGTAAATAGTGAAGCCACAGATGTATTTACTTTCTTCTTCTTCTTTGTGCCTACTGCCTCGGA
**SSRP1**	SSRP1.2143	TTCTTCTTCTTCTTTGACTTGT	TGCTGTTGACAGTGAGCGCCAAGTCAAAGAAGAAGAAGAATAGTGAAGCCACAGATGTATTCTTCTTCTTCTTTGACTTGTTGCCTACTGCCTCGGA
**STRAP**	STRAP.683	TAACAAATAATTACTATCCTGC	TGCTGTTGACAGTGAGCGACAGGATAGTAATTATTTGTTATAGTGAAGCCACAGATGTATAACAAATAATTACTATCCTGCTGCCTACTGCCTCGGA
**STRAP**	STRAP.1693	TAAACAGCTACAAGAACCCTAA	TGCTGTTGACAGTGAGCGCTAGGGTTCTTGTAGCTGTTTATAGTGAAGCCACAGATGTATAAACAGCTACAAGAACCCTAATGCCTACTGCCTCGGA
**STRAP**	STRAP.683	TTAACAAATAATTACTATCCTG	TGCTGTTGACAGTGAGCGAAGGATAGTAATTATTTGTTAATAGTGAAGCCACAGATGTATTAACAAATAATTACTATCCTGTGCCTACTGCCTCGGA
**STRAP**	STRAP.859	TGTCATAGTAGCATGATCCCAA	TGCTGTTGACAGTGAGCGCTGGGATCATGCTACTATGACATAGTGAAGCCACAGATGTATGTCATAGTAGCATGATCCCAATGCCTACTGCCTCGGA
**STRAP**	STRAP.1476	TAAGCAGACAGTAACTCTGGAA	TGCTGTTGACAGTGAGCGCTCCAGAGTTACTGTCTGCTTATAGTGAAGCCACAGATGTATAAGCAGACAGTAACTCTGGAATGCCTACTGCCTCGGA
**TCERG1**	TCERG1.2467	TTGATGAACTATCTACTGCTTT	TGCTGTTGACAGTGAGCGCAAGCAGTAGATAGTTCATCAATAGTGAAGCCACAGATGTATTGATGAACTATCTACTGCTTTTGCCTACTGCCTCGGA
**TCERG1**	TCERG1.1866	TTTATTATCGTCTCTCTTCCGT	TGCTGTTGACAGTGAGCGCCGGAAGAGAGACGATAATAAATAGTGAAGCCACAGATGTATTTATTATCGTCTCTCTTCCGTTGCCTACTGCCTCGGA

Gene Symbol	shRNA Name	Guide	97mer
**TCERG1**	TCERG1.3848	TAAACAACAAGACTCGGTCTAT	TGCTGTTGACAGTGAGCGCTAGACCGAGTCTTGTTGTTTATAGTGAAGCCACAGATGTATAAACAACAAGACTCGGTCTATTGCCTACTGCCTCGGA
**TCERG1**	TCERG1.1882	TTATTATCGTCTCTCTTCCGTT	TGCTGTTGACAGTGAGCGCACGGAAGAGAGACGATAATAATAGTGAAGCCACAGATGTATTATTATCGTCTCTCTTCCGTTTGCCTACTGCCTCGGA
**TCERG1**	TCERG1.2725	TATCAGACCATGACACATCTGA	TGCTGTTGACAGTGAGCGCCAGATGTGTCATGGTCTGATATAGTGAAGCCACAGATGTATATCAGACCATGACACATCTGATGCCTACTGCCTCGGA
**TCOF1**	TCOF1.313	TTTCTTAGCTTGCAGTGCCGCA	TGCTGTTGACAGTGAGCGCGCGGCACTGCAAGCTAAGAAATAGTGAAGCCACAGATGTATTTCTTAGCTTGCAGTGCCGCATGCCTACTGCCTCGGA
**TCOF1**	TCOF1.2214	TTTACTGAGGTTTTCACCTGTG	TGCTGTTGACAGTGAGCGAACAGGTGAAAACCTCAGTAAATAGTGAAGCCACAGATGTATTTACTGAGGTTTTCACCTGTGTGCCTACTGCCTCGGA
**TCOF1**	TCOF1.459	TTTTCTTTCATGCTTGATGGCA	TGCTGTTGACAGTGAGCGCGCCATCAAGCATGAAAGAAAATAGTGAAGCCACAGATGTATTTTCTTTCATGCTTGATGGCATGCCTACTGCCTCGGA
**TCOF1**	TCOF1.458	TTTCTTTCATGCTTGATGGCAA	TGCTGTTGACAGTGAGCGCTGCCATCAAGCATGAAAGAAATAGTGAAGCCACAGATGTATTTCTTTCATGCTTGATGGCAATGCCTACTGCCTCGGA
**TCOF1**	TCOF1.312	TTCTTAGCTTGCAGTGCCGCAT	TGCTGTTGACAGTGAGCGCTGCGGCACTGCAAGCTAAGAATAGTGAAGCCACAGATGTATTCTTAGCTTGCAGTGCCGCATTGCCTACTGCCTCGGA
**TFAM**	TFAM.455	TATCTCTTCTTTATATACCTGC	TGCTGTTGACAGTGAGCGACAGGTATATAAAGAAGAGATATAGTGAAGCCACAGATGTATATCTCTTCTTTATATACCTGCTGCCTACTGCCTCGGA
**TFAM**	TFAM.2312	TTCTAAAACAAGAACATCTGAA	TGCTGTTGACAGTGAGCGCTCAGATGTTCTTGTTTTAGAATAGTGAAGCCACAGATGTATTCTAAAACAAGAACATCTGAATGCCTACTGCCTCGGA
**TFAM**	TFAM.465	TAAATCTGCTTATCTCTTCTTT	TGCTGTTGACAGTGAGCGCAAGAAGAGATAAGCAGATTTATAGTGAAGCCACAGATGTATAAATCTGCTTATCTCTTCTTTTGCCTACTGCCTCGGA
**TFAM**	TFAM.674	TTCCTTTACAGTCTTCAGCTTT	TGCTGTTGACAGTGAGCGCAAGCTGAAGACTGTAAAGGAATAGTGAAGCCACAGATGTATTCCTTTACAGTCTTCAGCTTTTGCCTACTGCCTCGGA
**TFAM**	TFAM.3304	TTAAGTATAAAATAATACGGGT	TGCTGTTGACAGTGAGCGCCCCGTATTATTTTATACTTAATAGTGAAGCCACAGATGTATTAAGTATAAAATAATACGGGTTGCCTACTGCCTCGGA
**TFDP1**	TFDP1.1542	TAGCTTACCAATATCTTCTTGG	TGCTGTTGACAGTGAGCGACAAGAAGATATTGGTAAGCTATAGTGAAGCCACAGATGTATAGCTTACCAATATCTTCTTGGTGCCTACTGCCTCGGA
**TFDP1**	TFDP1.865	TTGAAGTTGAGACTGTTTCTGT	TGCTGTTGACAGTGAGCGCCAGAAACAGTCTCAACTTCAATAGTGAAGCCACAGATGTATTGAAGTTGAGACTGTTTCTGTTGCCTACTGCCTCGGA
**TFDP1**	TFDP1.2514	TTCAATAAAAAGACTATCGTTT	TGCTGTTGACAGTGAGCGCAACGATAGTCTTTTTATTGAATAGTGAAGCCACAGATGTATTCAATAAAAAGACTATCGTTTTGCCTACTGCCTCGGA
**TFDP1**	TFDP1.841	TATTCTTTCAAGTCTCCTCTGT	TGCTGTTGACAGTGAGCGCCAGAGGAGACTTGAAAGAATATAGTGAAGCCACAGATGTATATTCTTTCAAGTCTCCTCTGTTGCCTACTGCCTCGGA
**TFDP1**	TFDP1.2064	TTTGTTGCTGCAAAACAGGGAG	TGCTGTTGACAGTGAGCGATCCCTGTTTTGCAGCAACAAATAGTGAAGCCACAGATGTATTTGTTGCTGCAAAACAGGGAGTGCCTACTGCCTCGGA
**TGIF1**	TGIF1.787	TTAAGCTGTAAGTTTTGCCTGA	TGCTGTTGACAGTGAGCGCCAGGCAAAACTTACAGCTTAATAGTGAAGCCACAGATGTATTAAGCTGTAAGTTTTGCCTGATGCCTACTGCCTCGGA
**TGIF1**	TGIF1.962	TTTAAGTTTATAGTTCTTGGAA	TGCTGTTGACAGTGAGCGCTCCAAGAACTATAAACTTAAATAGTGAAGCCACAGATGTATTTAAGTTTATAGTTCTTGGAATGCCTACTGCCTCGGA
**TGIF1**	TGIF1.954	TATAGTTCTTGGAATGACTGTA	TGCTGTTGACAGTGAGCGCACAGTCATTCCAAGAACTATATAGTGAAGCCACAGATGTATATAGTTCTTGGAATGACTGTATGCCTACTGCCTCGGA
**TGIF1**	TGIF1.199	TAGACAGGTGTGTTTGCTGGGA	TGCTGTTGACAGTGAGCGCCCCAGCAAACACACCTGTCTATAGTGAAGCCACAGATGTATAGACAGGTGTGTTTGCTGGGATGCCTACTGCCTCGGA
**TGIF1**	TGIF1.510	TTTCAATGCAGTCACAGTGGTA	TGCTGTTGACAGTGAGCGCACCACTGTGACTGCATTGAAATAGTGAAGCCACAGATGTATTTCAATGCAGTCACAGTGGTATGCCTACTGCCTCGGA
**TIMELESS**	TIMELESS.4012	TAAATCTCCAGAGAGCTGCTGG	TGCTGTTGACAGTGAGCGACAGCAGCTCTCTGGAGATTTATAGTGAAGCCACAGATGTATAAATCTCCAGAGAGCTGCTGGTGCCTACTGCCTCGGA
**TIMELESS**	TIMELESS.4984	TTGCAGTATTTATCCATCCTTT	TGCTGTTGACAGTGAGCGCAAGGATGGATAAATACTGCAATAGTGAAGCCACAGATGTATTGCAGTATTTATCCATCCTTTTGCCTACTGCCTCGGA
**TIMELESS**	TIMELESS.939	TTTTCTGCCATCTCTCGCTGGC	TGCTGTTGACAGTGAGCGACCAGCGAGAGATGGCAGAAAATAGTGAAGCCACAGATGTATTTTCTGCCATCTCTCGCTGGCTGCCTACTGCCTCGGA
**TIMELESS**	TIMELESS.1233	TTTACTGATCCCATGAGCCGGT	TGCTGTTGACAGTGAGCGCCCGGCTCATGGGATCAGTAAATAGTGAAGCCACAGATGTATTTACTGATCCCATGAGCCGGTTGCCTACTGCCTCGGA

Gene Symbol	shRNA Name	Guide	97mer
**TIMELESS**	TIMELESS.2900	TATTCTTCATGATATGACCCAG	TGCTGTTGACAGTGAGCGATGGGTCATATCATGAAGAATATAGTGAAGCCACAGATGTATATTCTTCATGATATGACCCAGTGCCTACTGCCTCGGA
**TOP2B**	TOP2B.232	TACATCTTCATCATACACCCAC	TGCTGTTGACAGTGAGCGATGGGTGTATGATGAAGATGTATAGTGAAGCCACAGATGTATACATCTTCATCATACACCCACTGCCTACTGCCTCGGA
**TOP2B**	TOP2B.849	TAAAGATCTACATAACTGCGAA	TGCTGTTGACAGTGAGCGCTCGCAGTTATGTAGATCTTTATAGTGAAGCCACAGATGTATAAAGATCTACATAACTGCGAATGCCTACTGCCTCGGA
**TOP2B**	TOP2B.1679	TTTTATGTGAGAACCATCTTGA	TGCTGTTGACAGTGAGCGCCAAGATGGTTCTCACATAAAATAGTGAAGCCACAGATGTATTTTATGTGAGAACCATCTTGATGCCTACTGCCTCGGA
**TOP2B**	TOP2B.4436	TTTACTTGGAACTTTATCTGTC	TGCTGTTGACAGTGAGCGAACAGATAAAGTTCCAAGTAAATAGTGAAGCCACAGATGTATTTACTTGGAACTTTATCTGTCTGCCTACTGCCTCGGA
**TOP2B**	TOP2B.2861	TAGCATAGGTTCTAAAACCTGT	TGCTGTTGACAGTGAGCGCCAGGTTTTAGAACCTATGCTATAGTGAAGCCACAGATGTATAGCATAGGTTCTAAAACCTGTTGCCTACTGCCTCGGA
**TRIP13**	TRIP13.858	TTTATCATCAATCAAATCCTGA	TGCTGTTGACAGTGAGCGCCAGGATTTGATTGATGATAAATAGTGAAGCCACAGATGTATTTATCATCAATCAAATCCTGATGCCTACTGCCTCGGA
**TRIP13**	TRIP13.264	TAGCTTTCTAACACTCAGGTTT	TGCTGTTGACAGTGAGCGCAACCTGAGTGTTAGAAAGCTATAGTGAAGCCACAGATGTATAGCTTTCTAACACTCAGGTTTTGCCTACTGCCTCGGA
**TRIP13**	TRIP13.839	TGAATCTTCTGAAACATCTTGG	TGCTGTTGACAGTGAGCGACAAGATGTTTCAGAAGATTCATAGTGAAGCCACAGATGTATGAATCTTCTGAAACATCTTGGTGCCTACTGCCTCGGA
**TRIP13**	TRIP13.989	TTTAATCTGATCAATTTGGGTC	TGCTGTTGACAGTGAGCGAACCCAAATTGATCAGATTAAATAGTGAAGCCACAGATGTATTTAATCTGATCAATTTGGGTCTGCCTACTGCCTCGGA
**TRIP13**	TRIP13.241	TTTATGTCTTCTTTCTTTGCAG	TGCTGTTGACAGTGAGCGATGCAAAGAAAGAAGACATAAATAGTGAAGCCACAGATGTATTTATGTCTTCTTTCTTTGCAGTGCCTACTGCCTCGGA
**WBP11**	WBP11.2579	TAATTGTACATATTCTTCCCAC	TGCTGTTGACAGTGAGCGATGGGAAGAATATGTACAATTATAGTGAAGCCACAGATGTATAATTGTACATATTCTTCCCACTGCCTACTGCCTCGGA
**WBP11**	WBP11.846	TAACATGTCTTCATCTCGCCTA	TGCTGTTGACAGTGAGCGCAGGCGAGATGAAGACATGTTATAGTGAAGCCACAGATGTATAACATGTCTTCATCTCGCCTATGCCTACTGCCTCGGA
**WBP11**	WBP11.236	TTCTTTAATTCTCTCTTCCGGG	TGCTGTTGACAGTGAGCGACCGGAAGAGAGAATTAAAGAATAGTGAAGCCACAGATGTATTCTTTAATTCTCTCTTCCGGGTGCCTACTGCCTCGGA
**WBP11**	WBP11.2271	TTCAATAACTGATCTATTCTGG	TGCTGTTGACAGTGAGCGACAGAATAGATCAGTTATTGAATAGTGAAGCCACAGATGTATTCAATAACTGATCTATTCTGGTGCCTACTGCCTCGGA
**WBP11**	WBP11.1135	TTGAAGAGGAGTCAGTTCCTTC	TGCTGTTGACAGTGAGCGAAAGGAACTGACTCCTCTTCAATAGTGAAGCCACAGATGTATTGAAGAGGAGTCAGTTCCTTCTGCCTACTGCCTCGGA
**WHSC1**	WHSC1.1087	TTGAGGGTTGAGATGAAGCTGG	TGCTGTTGACAGTGAGCGACAGCTTCATCTCAACCCTCAATAGTGAAGCCACAGATGTATTGAGGGTTGAGATGAAGCTGGTGCCTACTGCCTCGGA
**WHSC1**	WHSC1.767	TTAAGTTTGGTATAGCTGTGAA	TGCTGTTGACAGTGAGCGCTCACAGCTATACCAAACTTAATAGTGAAGCCACAGATGTATTAAGTTTGGTATAGCTGTGAATGCCTACTGCCTCGGA
**WHSC1**	WHSC1.67	TTATGCACTTTACAACACTCTG	TGCTGTTGACAGTGAGCGAAGAGTGTTGTAAAGTGCATAATAGTGAAGCCACAGATGTATTATGCACTTTACAACACTCTGTGCCTACTGCCTCGGA
**WHSC1**	WHSC1.777	TTTCTGACCTTTAAGTTTGGTA	TGCTGTTGACAGTGAGCGCACCAAACTTAAAGGTCAGAAATAGTGAAGCCACAGATGTATTTCTGACCTTTAAGTTTGGTATGCCTACTGCCTCGGA
**WHSC1**	WHSC1.279	TTCTCCATTAAACACCCGGGAA	TGCTGTTGACAGTGAGCGCTCCCGGGTGTTTAATGGAGAATAGTGAAGCCACAGATGTATTCTCCATTAAACACCCGGGAATGCCTACTGCCTCGGA
**XRCC6**	XRCC6.842	TTGAGCTTCAGCTTTAACCTGC	TGCTGTTGACAGTGAGCGACAGGTTAAAGCTGAAGCTCAATAGTGAAGCCACAGATGTATTGAGCTTCAGCTTTAACCTGCTGCCTACTGCCTCGGA
**XRCC6**	XRCC6.1682	TTTCTCTTGGTAACTTTCCCTT	TGCTGTTGACAGTGAGCGCAGGGAAAGTTACCAAGAGAAATAGTGAAGCCACAGATGTATTTCTCTTGGTAACTTTCCCTTTGCCTACTGCCTCGGA
**XRCC6**	XRCC6.480	TGAGTGAGTAGTCAGATCCGTG	TGCTGTTGACAGTGAGCGAACGGATCTGACTACTCACTCATAGTGAAGCCACAGATGTATGAGTGAGTAGTCAGATCCGTGTGCCTACTGCCTCGGA
**XRCC6**	XRCC6.828	TTAACCTGCTGAGTGCTCGCTT	TGCTGTTGACAGTGAGCGCAGCGAGCACTCAGCAGGTTAATAGTGAAGCCACAGATGTATTAACCTGCTGAGTGCTCGCTTTGCCTACTGCCTCGGA
**XRCC6**	XRCC6.270	TACTGATGTACACACTTTGGAT	TGCTGTTGACAGTGAGCGCTCCAAAGTGTGTACATCAGTATAGTGAAGCCACAGATGTATACTGATGTACACACTTTGGATTGCCTACTGCCTCGGA
**ZNF207**	ZNF207.1823	TAAGCTTACAGAACTTGCCTTT	TGCTGTTGACAGTGAGCGCAAGGCAAGTTCTGTAAGCTTATAGTGAAGCCACAGATGTATAAGCTTACAGAACTTGCCTTTTGCCTACTGCCTCGGA

Gene Symbol	shRNA Name	Guide	97mer
**ZNF207**	ZNF207.1051	TTGTACTAGTTGTTGAAGCTGT	TGCTGTTGACAGTGAGCGCCAGCTTCAACAACTAGTACAATAGTGAAGCCACAGATGTATTGTACTAGTTGTTGAAGCTGTTGCCTACTGCCTCGGA
**ZNF207**	ZNF207.318	TATTGTTTCTTTATGTACCTGC	TGCTGTTGACAGTGAGCGACAGGTACATAAAGAAACAATATAGTGAAGCCACAGATGTATATTGTTTCTTTATGTACCTGCTGCCTACTGCCTCGGA
**ZNF207**	ZNF207.1042	TTGTTGAAGCTGTAGACTGTGT	TGCTGTTGACAGTGAGCGCCACAGTCTACAGCTTCAACAATAGTGAAGCCACAGATGTATTGTTGAAGCTGTAGACTGTGTTGCCTACTGCCTCGGA
**ZNF207**	ZNF207.257	TTTCTTGTGACATATATGGCAT	TGCTGTTGACAGTGAGCGCTGCCATATATGTCACAAGAAATAGTGAAGCCACAGATGTATTTCTTGTGACATATATGGCATTGCCTACTGCCTCGGA
