# Supplementary material for: Reduced replication origin licensing selectively kills KRAS-mutant colorectal cancer cells via mitotic catastrophe
Source: Cell Death Dis. 2020 Jul 1;11(7):499. doi: 10.1038/s41419-020-2704-9 (PMC7330027; doi:10.1038/s41419-020-2704-9)
Supplement: Supplementary file 7 — Table S2 - cell lines and culture media [file 41419_2020_2704_MOESM7_ESM.pdf]

Cell line	Authentication	Medium	Additions	Hygromycin	Puromycin	G418	Blasticidin
**CaCo2** (ATCC)	13 Feb 2018, CLS GmbH	DMEM	10% FCS, 1% Pen/Strep, 1% Ultraglutamine solution	250 µg/ml	5.0 µg/ml	1 250 µg/ml	---
**HT29** (ATCC)	11 Nov 2018, CLS GmbH	DMEM	10% FCS, 1% Pen/Strep, 1% Ultraglutamine solution	---	1.0 µg/ml	1 000 µg/ml	---
**WiDr**	29 May 2015, CLS GmbH	DMEM	10% FCS, 1% Pen/Strep, 1% Ultraglutamine solution	---	1.0 µg/ml	1 000 µg/ml	---
**SW480** (ATCC)	13 Feb 2018, CLS GmbH	DMEM	10% FCS, 1% Pen/Strep, 1% Ultraglutamine solution	---	5.0 µg/ml	1 250 µg/ml	---
**HCT8** (ATCC)	12 Apr 2016, CLS GmbH	DMEM	10% FCS, 1% Pen/Strep, 1% Ultraglutamine solution, 4g/L glucose	---	5.0 µg/ml	1 250 µg/ml	---
**DLD-1 ($KRAS^{wt/G13D}$)** (Horizon Discovery)	May 2016, freshly purchased	RPMI	10% FCS, 1% Pen/Strep	---	2.5 µg/ml	1 250 µg/ml	---
**DLD-1 ($KRAS^{wt/-}$)** (Horizon Discovery)	May 2016, freshly purchased	RPMI	10% FCS, 1% Pen/Strep	---	2.5 µg/ml	1 250 µg/ml	---
**PlatE (HEK293T)**	---	DMEM	10% FCS, 1% Pen/Strep, 1% Ultraglutamine solution, 4g/L glucose	---	1.0 µg/ml	---	10 µg/ml
**HCET1T** (ATCC)	Oct 2018, freshly purchased	DMEM/ Medium 199 4+1	4mM Glutamax-1, 20ng/ml EGF, 10µg/ml Insulin, 2µg/ml Apo-Transferrin, 5nM Sodium-Selenite, 1µg/ml Hydrocortisone	---	---	---	---
**CaR-1** (JCRB cell bank Japan)	Jan 2019, freshly purchased	EMEM	10% FCS, 1% Pen/Strep	---	---	---	---
