# Supplementary material for: Reduced replication origin licensing selectively kills KRAS-mutant colorectal cancer cells via mitotic catastrophe
Source: Cell Death Dis. 2020 Jul 1;11(7):499. doi: 10.1038/s41419-020-2704-9 (PMC7330027; doi:10.1038/s41419-020-2704-9)
Supplement: Supplementary file 9 — Supplementary Figure Legends and Supplementary Tables [file 41419_2020_2704_MOESM9_ESM.docx]

# SUPPLEMENTARY FIGURE LEGENDS

**Suppl. Figure 1. Characterization of CaCo2 cells after KRAS^G12V^ induction and MCM7 knockdown.**

(A) Representative soft agar assays after 5 weeks of KRAS^G12V^ expression and MCM7 knockdown.

(B) Representative flow cytometry analysis of cleaved Caspase 3 (clCaspase3) stained CaCo2 cells after 7 days of doxycycline induction. Cells that co-express KRAS^G12V^ and MCM7.sh3 show a significantly higher percentage of clCaspase3^+^ cells.

(C) Western blot shows an increase in cleaved Caspase 3 (clCaspase 3) in cells that co-express KRAS^G12V^ and MCM7.sh3 after 7 days of doxycycline induction.

**Suppl. Figure 2. Knockdown efficiency of MCM7 in DLD1, SW480, HCT‑8, HT‑29, and WiDr.**

(A) Western blot of the soluble and chromatin-bound cell fraction after two and seven days of doxycycline induction showed an equally efficient MCM7 knockdown in both DLD 1 cell lines. PCNA serves as a loading control for soluble and chromatin-bound protein. GAPDH serves as a loading control that is only present in the soluble fraction.

(B) Change in mRNA expression of SW480, HCT-8, HT-29, and WiDr cells after induction of MCM7.sh3. Knockdown of MCM7 caused an efficient suppression of MCM7 expression in all four cell lines after 3 days of doxycycline induction. Mean fold change normalized to scrbl control ± SD is shown (n = 2-3 technical replicates per group).

**Suppl. Figure 3. Consequences of mutant KRAS specific replication stress.**

(A) Western blots showed that CHK1 is specifically activated after 7 days of KRAS^G12V^ and MCM7.sh3 co-expression in CaCo2 cells. CHK2 is activated after 5 days of MCM7 knockdown, independently of KRAS^G12V^ expression. Vinculin serves as a loading control.

(B-E) Pharmacological CDC7 inhibition of KRAS mutant and KRAS wild-type cells. KRAS mutant CaCo2 (B, C) and DLD‑1 cells (D,E) showed increased resistance towards PHA 767491 (CDC7 inhibitor). Mean percentage of the relative area covered by cells ± SEM is shown (n = 3‑4 per group). Nonlinear regression - extra sum-of-squares F test (B,D) Mean IC_50_ ± SEM is shown (n = 4). Student’s t-test (two-sided) (C,E); * = p < 0.05; ** = p < 0.01; **** = p < 0.0001.

(F) Origin firing was decreased upon 24 h of CDC7 inhibition with PHA 767491 in CaCo2 cells as marked by decreased phosphorylation of MCM2 at S40. GAPDH serves as a loading control.

**Suppl. Figure 4. Mitotic catastrophe causes chromosomal aberrations.**

(A) FACS analysis showed an increase in mitotic cells after simultaneous KRAS^G12V^ expression and MCM7 knockdown. Mean percentage of pHH3^+^ cells ± SEM is shown (n = 2 per group).

(B) FACS analysis confirmed a specific increase of γH2AX^+^ cells in mitosis after simultaneous knockdown of MCM7 and KRAS^G12V^ expression. Mean percentage of γH2AX^+^ cells (gated on pHH3^+^ cells) ± SEM is shown (n = 2 per group).

(C) CaCo2 cells expression KRAS^G12V^ and MCM7.sh3 simultaneously for 7 days exhibit in part massive chromosomal aberrations and DNA damage during mitosis. Scale bar = 10 µm.

**Suppl. Figure 5. Perturbations of replication fork progression by hydroxyurea and oxaliplatin show increased efficacy in KRAS mutant CaCo2 cells.**

(A-D) KRAS induction caused a shift in IC_50_ from 207.3 µM to 154.7 µM with hydroxyurea (A,B) and 0.157 µM to 0.076 µM with oxaliplatin (C,D). Mean percentage of the relative area covered by cells ± SEM is shown (n = 3‑5 per group). Nonlinear regression - extra sum-of-squares F test (A,C); Mean IC_50_ ± SEM is shown (n = 3). Student’s t-test (two-sided) (B,D); ** = p < 0.01; *** = p < 0.001; **** = p < 0.0001.

# SUPPLEMENTARY TABLE LEGENDS

**Suppl. Table 1**

Sequences and labelling of 625 shRNAs within the library for 121 different target gene

| **GeneSymbol** | **shRNAName** | **Guide** | | **97mer** | |
| --- | --- | --- | --- | --- | --- |
| **DUSP4** | DUSP4.1283 | | TTAAGCATAATTATTCATCTGT | | TGCTGTTGACAGTGAGCGCCAGATGAATAATTATGCTTAATAGTGAAGCCACAGATGTATTAAGCATAATTATTCATCTGTTGCCTACTGCCTCGGA |
| **DUSP4** | DUSP4.31 | | TTTTAGAACAGAATTCTGGGTA | | TGCTGTTGACAGTGAGCGCACCCAGAATTCTGTTCTAAAATAGTGAAGCCACAGATGTATTTTAGAACAGAATTCTGGGTATGCCTACTGCCTCGGA |
| **DUSP4** | DUSP4.1193 | | TACAACAACGACAACAAAGGGA | | TGCTGTTGACAGTGAGCGCCCCTTTGTTGTCGTTGTTGTATAGTGAAGCCACAGATGTATACAACAACGACAACAAAGGGATGCCTACTGCCTCGGA |
| **DUSP4** | DUSP4.1545 | | TTAAAAAGCAATTCAAACCTAA | | TGCTGTTGACAGTGAGCGCTAGGTTTGAATTGCTTTTTAATAGTGAAGCCACAGATGTATTAAAAAGCAATTCAAACCTAATGCCTACTGCCTCGGA |
| **DUSP4** | DUSP4.2934 | | TATTTCTAGAGGAAGCAGGGAG | | TGCTGTTGACAGTGAGCGATCCCTGCTTCCTCTAGAAATATAGTGAAGCCACAGATGTATATTTCTAGAGGAAGCAGGGAGTGCCTACTGCCTCGGA |
| **DUSP6** | DUSP6.1299 | | TTGTCTAGTACAGACAGCTGGT | | TGCTGTTGACAGTGAGCGCCCAGCTGTCTGTACTAGACAATAGTGAAGCCACAGATGTATTGTCTAGTACAGACAGCTGGTTGCCTACTGCCTCGGA |
| **DUSP6** | DUSP6.1902 | | TTAGTGATAATAGTGTCCGTAA | | TGCTGTTGACAGTGAGCGCTACGGACACTATTATCACTAATAGTGAAGCCACAGATGTATTAGTGATAATAGTGTCCGTAATGCCTACTGCCTCGGA |
| **DUSP6** | DUSP6.1330 | | TTAGTATTAACCAATTCCGCAC | | TGCTGTTGACAGTGAGCGATGCGGAATTGGTTAATACTAATAGTGAAGCCACAGATGTATTAGTATTAACCAATTCCGCACTGCCTACTGCCTCGGA |
| **DUSP6** | DUSP6.1382 | | TATCTATACAGCATGTCCTGTT | | TGCTGTTGACAGTGAGCGCACAGGACATGCTGTATAGATATAGTGAAGCCACAGATGTATATCTATACAGCATGTCCTGTTTGCCTACTGCCTCGGA |
| **DUSP6** | DUSP6.2099 | | TATACTGTTTAGCACAGCTGAA | | TGCTGTTGACAGTGAGCGCTCAGCTGTGCTAAACAGTATATAGTGAAGCCACAGATGTATATACTGTTTAGCACAGCTGAATGCCTACTGCCTCGGA |
| **ELK1** | ELK1.447 | | TTGTAGACGAACTTCTGGCCGC | | TGCTGTTGACAGTGAGCGACGGCCAGAAGTTCGTCTACAATAGTGAAGCCACAGATGTATTGTAGACGAACTTCTGGCCGCTGCCTACTGCCTCGGA |
| **ELK1** | ELK1.1078 | | TAACTCTTCTACTCACATCCAA | | TGCTGTTGACAGTGAGCGCTGGATGTGAGTAGAAGAGTTATAGTGAAGCCACAGATGTATAACTCTTCTACTCACATCCAATGCCTACTGCCTCGGA |
| **ELK1** | ELK1.408 | | TTCTTGTCATAGTAGTACCGCA | | TGCTGTTGACAGTGAGCGCGCGGTACTACTATGACAAGAATAGTGAAGCCACAGATGTATTCTTGTCATAGTAGTACCGCATGCCTACTGCCTCGGA |
| **ELK1** | ELK1.1708 | | TTGCCTAGAATAGAGACAGGAC | | TGCTGTTGACAGTGAGCGATCCTGTCTCTATTCTAGGCAATAGTGAAGCCACAGATGTATTGCCTAGAATAGAGACAGGACTGCCTACTGCCTCGGA |
| **ELK1** | ELK1.1488 | | TATAGGAAGAGATGACTCCCTC | | TGCTGTTGACAGTGAGCGAAGGGAGTCATCTCTTCCTATATAGTGAAGCCACAGATGTATATAGGAAGAGATGACTCCCTCTGCCTACTGCCTCGGA |
| **MAPK1** | MAPK1.1316 | | TTTAAGATCTGTATCCTGGCTG | | TGCTGTTGACAGTGAGCGAAGCCAGGATACAGATCTTAAATAGTGAAGCCACAGATGTATTTAAGATCTGTATCCTGGCTGTGCCTACTGCCTCGGA |
| **MAPK1** | MAPK1.1233 | | TCCATGTCGAACTTGAATGGTG | | TGCTGTTGACAGTGAGCGAACCATTCAAGTTCGACATGGATAGTGAAGCCACAGATGTATCCATGTCGAACTTGAATGGTGTGCCTACTGCCTCGGA |
| **MAPK1** | MAPK1.1121 | | TGAATGTCAACATTTTGTCCAA | | TGCTGTTGACAGTGAGCGCTGGACAAAATGTTGACATTCATAGTGAAGCCACAGATGTATGAATGTCAACATTTTGTCCAATGCCTACTGCCTCGGA |
| **MAPK1** | MAPK1.528 | | TTCATTTGCTCGATGGTTGGTG | | TGCTGTTGACAGTGAGCGAACCAACCATCGAGCAAATGAATAGTGAAGCCACAGATGTATTCATTTGCTCGATGGTTGGTGTGCCTACTGCCTCGGA |
| **MAPK1** | MAPK1.537 | | TATACATCTTTCATTTGCTCGA | | TGCTGTTGACAGTGAGCGCCGAGCAAATGAAAGATGTATATAGTGAAGCCACAGATGTATATACATCTTTCATTTGCTCGATGCCTACTGCCTCGGA |
| **MAPK3** | MAPK3.1741 | | TATATTTATATATTAGACGGGT | | TGCTGTTGACAGTGAGCGCCCCGTCTAATATATAAATATATAGTGAAGCCACAGATGTATATATTTATATATTAGACGGGTTGCCTACTGCCTCGGA |
| **MAPK3** | MAPK3.1200 | | TTTTCTAACAGTCTGGCGGGAG | | TGCTGTTGACAGTGAGCGATCCCGCCAGACTGTTAGAAAATAGTGAAGCCACAGATGTATTTTCTAACAGTCTGGCGGGAGTGCCTACTGCCTCGGA |
| **MAPK3** | MAPK3.912 | | TAAAGGTTAACATCCGGTCCAG | | TGCTGTTGACAGTGAGCGATGGACCGGATGTTAACCTTTATAGTGAAGCCACAGATGTATAAAGGTTAACATCCGGTCCAGTGCCTACTGCCTCGGA |
| **MAPK3** | MAPK3.751 | | TTGGAGTTCAGCATGATCTCTG | | TGCTGTTGACAGTGAGCGAAGAGATCATGCTGAACTCCAATAGTGAAGCCACAGATGTATTGGAGTTCAGCATGATCTCTGTGCCTACTGCCTCGGA |
| **MAPK3** | MAPK3.616 | | TTGATGAGCAGGTTGGAGGGCT | | TGCTGTTGACAGTGAGCGCGCCCTCCAACCTGCTCATCAATAGTGAAGCCACAGATGTATTGATGAGCAGGTTGGAGGGCTTGCCTACTGCCTCGGA |
| **SRF** | SRF.2720 | | TTTGTTTTCACTTCGTCTCCTC | | TGCTGTTGACAGTGAGCGAAGGAGACGAAGTGAAAACAAATAGTGAAGCCACAGATGTATTTGTTTTCACTTCGTCTCCTCTGCCTACTGCCTCGGA |
| **SRF** | SRF.901 | | TCCATCTTGATCTTCACGCGGC | | TGCTGTTGACAGTGAGCGACCGCGTGAAGATCAAGATGGATAGTGAAGCCACAGATGTATCCATCTTGATCTTCACGCGGCTGCCTACTGCCTCGGA |
| **SRF** | SRF.85 | | TAAAGAGATACAATGTTTCCTT | | TGCTGTTGACAGTGAGCGCAGGAAACATTGTATCTCTTTATAGTGAAGCCACAGATGTATAAAGAGATACAATGTTTCCTTTGCCTACTGCCTCGGA |
| **SRF** | SRF.3603 | | TTCACCTAATCACAGAAGCCAG | | TGCTGTTGACAGTGAGCGATGGCTTCTGTGATTAGGTGAATAGTGAAGCCACAGATGTATTCACCTAATCACAGAAGCCAGTGCCTACTGCCTCGGA |
| **SRF** | SRF.1868 | | TGAAACAGGGATCTGCACTGTC | | TGCTGTTGACAGTGAGCGAACAGTGCAGATCCCTGTTTCATAGTGAAGCCACAGATGTATGAAACAGGGATCTGCACTGTCTGCCTACTGCCTCGGA |
| **AATF** | AATF.1732 | | TTTTGCTTCGTAACTTCTGGAT | | TGCTGTTGACAGTGAGCGCTCCAGAAGTTACGAAGCAAAATAGTGAAGCCACAGATGTATTTTGCTTCGTAACTTCTGGATTGCCTACTGCCTCGGA |
| **AATF** | AATF.1150 | | TAGATATCTAGTGTCTGGGTAC | | TGCTGTTGACAGTGAGCGATACCCAGACACTAGATATCTATAGTGAAGCCACAGATGTATAGATATCTAGTGTCTGGGTACTGCCTACTGCCTCGGA |
| **AATF** | AATF.968 | | TTTTGTAGTTTGATCCTTCCTT | | TGCTGTTGACAGTGAGCGCAGGAAGGATCAAACTACAAAATAGTGAAGCCACAGATGTATTTTGTAGTTTGATCCTTCCTTTGCCTACTGCCTCGGA |
| **AATF** | AATF.1152 | | TACTAGATATCTAGTGTCTGGG | | TGCTGTTGACAGTGAGCGACCAGACACTAGATATCTAGTATAGTGAAGCCACAGATGTATACTAGATATCTAGTGTCTGGGTGCCTACTGCCTCGGA |
| **AATF** | AATF.1223 | | TTCTTCTCTTCTACCAGCTCAT | | TGCTGTTGACAGTGAGCGCTGAGCTGGTAGAAGAGAAGAATAGTGAAGCCACAGATGTATTCTTCTCTTCTACCAGCTCATTGCCTACTGCCTCGGA |
| **ANKRD57** | ANKRD57.2246 | | TTGCATTTCAACTTTGATCTGA | | TGCTGTTGACAGTGAGCGCCAGATCAAAGTTGAAATGCAATAGTGAAGCCACAGATGTATTGCATTTCAACTTTGATCTGATGCCTACTGCCTCGGA |
| **ANKRD57** | ANKRD57.2974 | | TTAAATATAGAAATAGTCCCAT | | TGCTGTTGACAGTGAGCGCTGGGACTATTTCTATATTTAATAGTGAAGCCACAGATGTATTAAATATAGAAATAGTCCCATTGCCTACTGCCTCGGA |
| **ANKRD57** | ANKRD57.3667 | | TTTTGAGACAGAGTCTCCGTTG | | TGCTGTTGACAGTGAGCGAAACGGAGACTCTGTCTCAAAATAGTGAAGCCACAGATGTATTTTGAGACAGAGTCTCCGTTGTGCCTACTGCCTCGGA |
| **AREG** | AREG.517 | | TTAACTACCTGTTCAACTCTGA | | TGCTGTTGACAGTGAGCGCCAGAGTTGAACAGGTAGTTAATAGTGAAGCCACAGATGTATTAACTACCTGTTCAACTCTGATGCCTACTGCCTCGGA |
| **AREG** | AREG.677 | | TATATATTTGCATTCTCCGTGA | | TGCTGTTGACAGTGAGCGCCACGGAGAATGCAAATATATATAGTGAAGCCACAGATGTATATATATTTGCATTCTCCGTGATGCCTACTGCCTCGGA |
| **AREG** | AREG.868 | | TTGTCTTCTAAGCTGGACTGTA | | TGCTGTTGACAGTGAGCGCACAGTCCAGCTTAGAAGACAATAGTGAAGCCACAGATGTATTGTCTTCTAAGCTGGACTGTATGCCTACTGCCTCGGA |
| **AREG** | AREG.850 | | TGTAATAACAGCAACAGCTGTG | | TGCTGTTGACAGTGAGCGAACAGCTGTTGCTGTTATTACATAGTGAAGCCACAGATGTATGTAATAACAGCAACAGCTGTGTGCCTACTGCCTCGGA |
| **AREG** | AREG.460 | | TTCGTTATCATACTCTTCTGAG | | TGCTGTTGACAGTGAGCGATCAGAAGAGTATGATAACGAATAGTGAAGCCACAGATGTATTCGTTATCATACTCTTCTGAGTGCCTACTGCCTCGGA |
| **ARNTL2** | ARNTL2.1018 | | TTGTCTTTCTTACTGTTCCTTT | | TGCTGTTGACAGTGAGCGCAAGGAACAGTAAGAAAGACAATAGTGAAGCCACAGATGTATTGTCTTTCTTACTGTTCCTTTTGCCTACTGCCTCGGA |
| **ARNTL2** | ARNTL2.674 | | TTGTCCAGTCAAACTAGCCTGA | | TGCTGTTGACAGTGAGCGCCAGGCTAGTTTGACTGGACAATAGTGAAGCCACAGATGTATTGTCCAGTCAAACTAGCCTGATGCCTACTGCCTCGGA |
| **ARNTL2** | ARNTL2.111 | | TAGCTGTTGGTCTTGTCCCTGG | | TGCTGTTGACAGTGAGCGACAGGGACAAGACCAACAGCTATAGTGAAGCCACAGATGTATAGCTGTTGGTCTTGTCCCTGGTGCCTACTGCCTCGGA |
| **ARNTL2** | ARNTL2.2192 | | TATGTAAGTAGTATTCTTGGTT | | TGCTGTTGACAGTGAGCGCACCAAGAATACTACTTACATATAGTGAAGCCACAGATGTATATGTAAGTAGTATTCTTGGTTTGCCTACTGCCTCGGA |
| **ARNTL2** | ARNTL2.3342 | | TTTTGTTTTATTACCTTTGGTT | | TGCTGTTGACAGTGAGCGCACCAAAGGTAATAAAACAAAATAGTGAAGCCACAGATGTATTTTGTTTTATTACCTTTGGTTTGCCTACTGCCTCGGA |
| **ATAD2** | ATAD2.1342 | | TTGCATTGGATCAACATCGGCA | | TGCTGTTGACAGTGAGCGCGCCGATGTTGATCCAATGCAATAGTGAAGCCACAGATGTATTGCATTGGATCAACATCGGCATGCCTACTGCCTCGGA |
| **ATAD2** | ATAD2.569 | | TAATCCTACAACTTCGACGCAC | | TGCTGTTGACAGTGAGCGATGCGTCGAAGTTGTAGGATTATAGTGAAGCCACAGATGTATAATCCTACAACTTCGACGCACTGCCTACTGCCTCGGA |
| **ATAD2** | ATAD2.1599 | | TACTTAGACAATCAGCACCTTT | | TGCTGTTGACAGTGAGCGCAAGGTGCTGATTGTCTAAGTATAGTGAAGCCACAGATGTATACTTAGACAATCAGCACCTTTTGCCTACTGCCTCGGA |
| **ATAD2** | ATAD2.4380 | | TACTATTTTAATCTTTTCCTTA | | TGCTGTTGACAGTGAGCGCAAGGAAAAGATTAAAATAGTATAGTGAAGCCACAGATGTATACTATTTTAATCTTTTCCTTATGCCTACTGCCTCGGA |
| **ATAD2** | ATAD2.3499 | | TTTATCACCAACAAGAGTGGAA | | TGCTGTTGACAGTGAGCGCTCCACTCTTGTTGGTGATAAATAGTGAAGCCACAGATGTATTTATCACCAACAAGAGTGGAATGCCTACTGCCTCGGA |
| **BARD1** | BARD1.548 | | TTTAATTGAATTCTTCTTGTTT | | TGCTGTTGACAGTGAGCGCAACAAGAAGAATTCAATTAAATAGTGAAGCCACAGATGTATTTAATTGAATTCTTCTTGTTTTGCCTACTGCCTCGGA |
| **BARD1** | BARD1.1690 | | TATATTAACAGCATTTCTGGAG | | TGCTGTTGACAGTGAGCGATCCAGAAATGCTGTTAATATATAGTGAAGCCACAGATGTATATATTAACAGCATTTCTGGAGTGCCTACTGCCTCGGA |
| **BARD1** | BARD1.543 | | TTGAATTCTTCTTGTTTCCTGC | | TGCTGTTGACAGTGAGCGACAGGAAACAAGAAGAATTCAATAGTGAAGCCACAGATGTATTGAATTCTTCTTGTTTCCTGCTGCCTACTGCCTCGGA |
| **BARD1** | BARD1.582 | | TATCTGACTTTCTTACTTCGAG | | TGCTGTTGACAGTGAGCGATCGAAGTAAGAAAGTCAGATATAGTGAAGCCACAGATGTATATCTGACTTTCTTACTTCGAGTGCCTACTGCCTCGGA |
| **BARD1** | BARD1.1647 | | TTGACTATATCCACATGCCCAT | | TGCTGTTGACAGTGAGCGCTGGGCATGTGGATATAGTCAATAGTGAAGCCACAGATGTATTGACTATATCCACATGCCCATTGCCTACTGCCTCGGA |
| **BAZ1A** | BAZ1A.1434 | | TTTATTAGCAACATTGTCCTCT | | TGCTGTTGACAGTGAGCGCGAGGACAATGTTGCTAATAAATAGTGAAGCCACAGATGTATTTATTAGCAACATTGTCCTCTTGCCTACTGCCTCGGA |
| **BAZ1A** | BAZ1A.5651 | | TAAACTTGGACTTAATACCTGC | | TGCTGTTGACAGTGAGCGACAGGTATTAAGTCCAAGTTTATAGTGAAGCCACAGATGTATAAACTTGGACTTAATACCTGCTGCCTACTGCCTCGGA |
| **BAZ1A** | BAZ1A.4264 | | TAACTTGTGGTCTTCCTCGTTT | | TGCTGTTGACAGTGAGCGCAACGAGGAAGACCACAAGTTATAGTGAAGCCACAGATGTATAACTTGTGGTCTTCCTCGTTTTGCCTACTGCCTCGGA |
| **BAZ1A** | BAZ1A.5297 | | TATCTTTCCTACATTCTCCTGA | | TGCTGTTGACAGTGAGCGCCAGGAGAATGTAGGAAAGATATAGTGAAGCCACAGATGTATATCTTTCCTACATTCTCCTGATGCCTACTGCCTCGGA |
| **BAZ1A** | BAZ1A.4218 | | TTGAGAGTCATCTTCATCTTGT | | TGCTGTTGACAGTGAGCGCCAAGATGAAGATGACTCTCAATAGTGAAGCCACAGATGTATTGAGAGTCATCTTCATCTTGTTGCCTACTGCCTCGGA |
| **BAZ1B** | BAZ1B.5150 | | TAAACATTTAAATTCTTCCTGC | | TGCTGTTGACAGTGAGCGACAGGAAGAATTTAAATGTTTATAGTGAAGCCACAGATGTATAAACATTTAAATTCTTCCTGCTGCCTACTGCCTCGGA |
| **BAZ1B** | BAZ1B.3381 | | TTGACTTTCTCTTATTCCCTGA | | TGCTGTTGACAGTGAGCGCCAGGGAATAAGAGAAAGTCAATAGTGAAGCCACAGATGTATTGACTTTCTCTTATTCCCTGATGCCTACTGCCTCGGA |
| **BAZ1B** | BAZ1B.2041 | | TTTCTTTCTCTCTTCGTTCTTT | | TGCTGTTGACAGTGAGCGCAAGAACGAAGAGAGAAAGAAATAGTGAAGCCACAGATGTATTTCTTTCTCTCTTCGTTCTTTTGCCTACTGCCTCGGA |
| **BAZ1B** | BAZ1B.2230 | | TAATAGGATACTGAGCATCTGG | | TGCTGTTGACAGTGAGCGACAGATGCTCAGTATCCTATTATAGTGAAGCCACAGATGTATAATAGGATACTGAGCATCTGGTGCCTACTGCCTCGGA |
| **BAZ1B** | BAZ1B.3695 | | TTTTCTTCTCTTTTGCTTGGGA | | TGCTGTTGACAGTGAGCGCCCCAAGCAAAAGAGAAGAAAATAGTGAAGCCACAGATGTATTTTCTTCTCTTTTGCTTGGGATGCCTACTGCCTCGGA |
| **BRCA1** | BRCA1.656 | | TTCAGTATTTGTTACATCCGTC | | TGCTGTTGACAGTGAGCGAACGGATGTAACAAATACTGAATAGTGAAGCCACAGATGTATTCAGTATTTGTTACATCCGTCTGCCTACTGCCTCGGA |
| **BRCA1** | BRCA1.2561 | | TAAACTTAGGGAAACCAGCTAT | | TGCTGTTGACAGTGAGCGCTAGCTGGTTTCCCTAAGTTTATAGTGAAGCCACAGATGTATAAACTTAGGGAAACCAGCTATTGCCTACTGCCTCGGA |
| **BRCA1** | BRCA1.1108 | | TTTTACTGGTAGAACTATCTGC | | TGCTGTTGACAGTGAGCGACAGATAGTTCTACCAGTAAAATAGTGAAGCCACAGATGTATTTTACTGGTAGAACTATCTGCTGCCTACTGCCTCGGA |
| **BRCA1** | BRCA1.37 | | TTTTGTACTTCTTCAACGCGAA | | TGCTGTTGACAGTGAGCGCTCGCGTTGAAGAAGTACAAAATAGTGAAGCCACAGATGTATTTTGTACTTCTTCAACGCGAATGCCTACTGCCTCGGA |
| **BRCA1** | BRCA1.190 | | TAAATCTCGTACTTTCTTGTAG | | TGCTGTTGACAGTGAGCGATACAAGAAAGTACGAGATTTATAGTGAAGCCACAGATGTATAAATCTCGTACTTTCTTGTAGTGCCTACTGCCTCGGA |
| **CBFB** | CBFB.3057 | | TAAACAAACAAACACACAGTAT | | TGCTGTTGACAGTGAGCGCTACTGTGTGTTTGTTTGTTTATAGTGAAGCCACAGATGTATAAACAAACAAACACACAGTATTGCCTACTGCCTCGGA |
| **CBFB** | CBFB.2451 | | TAACAATTTAAACACACTCCTT | | TGCTGTTGACAGTGAGCGCAGGAGTGTGTTTAAATTGTTATAGTGAAGCCACAGATGTATAACAATTTAAACACACTCCTTTGCCTACTGCCTCGGA |
| **CBFB** | CBFB.507 | | TCTAAGTCGACATACTCTCGGC | | TGCTGTTGACAGTGAGCGACCGAGAGTATGTCGACTTAGATAGTGAAGCCACAGATGTATCTAAGTCGACATACTCTCGGCTGCCTACTGCCTCGGA |
| **CBFB** | CBFB.665 | | TTGTGCTAATGCATCCTCCTGC | | TGCTGTTGACAGTGAGCGACAGGAGGATGCATTAGCACAATAGTGAAGCCACAGATGTATTGTGCTAATGCATCCTCCTGCTGCCTACTGCCTCGGA |
| **CBFB** | CBFB.2457 | | AACAATTTAAACACACTCCTTT | | TGCTGTTGACAGTGAGCGCAAGGAGTGTGTTTAAATTGTTTAGTGAAGCCACAGATGTAAACAATTTAAACACACTCCTTTTGCCTACTGCCTCGGA |
| **CBX3** | CBX3.1941 | | TTTAATGAGACAATTGACCCTA | | TGCTGTTGACAGTGAGCGCAGGGTCAATTGTCTCATTAAATAGTGAAGCCACAGATGTATTTAATGAGACAATTGACCCTATGCCTACTGCCTCGGA |
| **CBX3** | CBX3.560 | | TTATTGAGCTTCATCTTCTGGA | | TGCTGTTGACAGTGAGCGCCCAGAAGATGAAGCTCAATAATAGTGAAGCCACAGATGTATTATTGAGCTTCATCTTCTGGATGCCTACTGCCTCGGA |
| **CBX3** | CBX3.1278 | | TTGTCTAGTTTCCTCATCTGGA | | TGCTGTTGACAGTGAGCGCCCAGATGAGGAAACTAGACAATAGTGAAGCCACAGATGTATTGTCTAGTTTCCTCATCTGGATGCCTACTGCCTCGGA |
| **CBX3** | CBX3.1081 | | TTATCTCTTATTTTGCTTGGAA | | TGCTGTTGACAGTGAGCGCTCCAAGCAAAATAAGAGATAATAGTGAAGCCACAGATGTATTATCTCTTATTTTGCTTGGAATGCCTACTGCCTCGGA |
| **CBX3** | CBX3.628 | | TAAATCAAAATCTAAGACCCAA | | TGCTGTTGACAGTGAGCGCTGGGTCTTAGATTTTGATTTATAGTGAAGCCACAGATGTATAAATCAAAATCTAAGACCCAATGCCTACTGCCTCGGA |
| **CHAF1A** | CHAF1A.1387 | | TTCTCTTCTTCTCTTAACCGTT | | TGCTGTTGACAGTGAGCGCACGGTTAAGAGAAGAAGAGAATAGTGAAGCCACAGATGTATTCTCTTCTTCTCTTAACCGTTTGCCTACTGCCTCGGA |
| **CHAF1A** | CHAF1A.3082 | | TAGAACTCTGCACACTTTGGGG | | TGCTGTTGACAGTGAGCGACCCAAAGTGTGCAGAGTTCTATAGTGAAGCCACAGATGTATAGAACTCTGCACACTTTGGGGTGCCTACTGCCTCGGA |
| **CHAF1A** | CHAF1A.182 | | TTGTATTAACTTCTTAACTGGA | | TGCTGTTGACAGTGAGCGCCCAGTTAAGAAGTTAATACAATAGTGAAGCCACAGATGTATTGTATTAACTTCTTAACTGGATGCCTACTGCCTCGGA |
| **CHAF1A** | CHAF1A.1374 | | TAACCGTTTCTCTTCTTCCTTT | | TGCTGTTGACAGTGAGCGCAAGGAAGAAGAGAAACGGTTATAGTGAAGCCACAGATGTATAACCGTTTCTCTTCTTCCTTTTGCCTACTGCCTCGGA |
| **CHAF1A** | CHAF1A.3031 | | TTACACAGGAATTGAGTCGGTT | | TGCTGTTGACAGTGAGCGCACCGACTCAATTCCTGTGTAATAGTGAAGCCACAGATGTATTACACAGGAATTGAGTCGGTTTGCCTACTGCCTCGGA |
| **CTNNBL1** | CTNNBL1.388 | | TTGTCTGGAAACTTAATCCGCA | | TGCTGTTGACAGTGAGCGCGCGGATTAAGTTTCCAGACAATAGTGAAGCCACAGATGTATTGTCTGGAAACTTAATCCGCATGCCTACTGCCTCGGA |
| **CTNNBL1** | CTNNBL1.961 | | TTAAACACGGATAACTGCTGAA | | TGCTGTTGACAGTGAGCGCTCAGCAGTTATCCGTGTTTAATAGTGAAGCCACAGATGTATTAAACACGGATAACTGCTGAATGCCTACTGCCTCGGA |
| **CTNNBL1** | CTNNBL1.1828 | | TTGTGTAGAAACTGATCCTGGG | | TGCTGTTGACAGTGAGCGACCAGGATCAGTTTCTACACAATAGTGAAGCCACAGATGTATTGTGTAGAAACTGATCCTGGGTGCCTACTGCCTCGGA |
| **CTNNBL1** | CTNNBL1.1653 | | TTTTGATGGAGCTTCCTCGCAT | | TGCTGTTGACAGTGAGCGCTGCGAGGAAGCTCCATCAAAATAGTGAAGCCACAGATGTATTTTGATGGAGCTTCCTCGCATTGCCTACTGCCTCGGA |
| **CTNNBL1** | CTNNBL1.1495 | | TTGTCGATGATCTCTCCTCGCC | | TGCTGTTGACAGTGAGCGAGCGAGGAGAGATCATCGACAATAGTGAAGCCACAGATGTATTGTCGATGATCTCTCCTCGCCTGCCTACTGCCTCGGA |
| **DAB2IP** | DAB2IP.2670 | | TCGTCTTCTAACAACTGCGCGT | | TGCTGTTGACAGTGAGCGCCGCGCAGTTGTTAGAAGACGATAGTGAAGCCACAGATGTATCGTCTTCTAACAACTGCGCGTTGCCTACTGCCTCGGA |
| **DAB2IP** | DAB2IP.468 | | TTCTTCTTGTCGGTCTCCCGGT | | TGCTGTTGACAGTGAGCGCCCGGGAGACCGACAAGAAGAATAGTGAAGCCACAGATGTATTCTTCTTGTCGGTCTCCCGGTTGCCTACTGCCTCGGA |
| **DAB2IP** | DAB2IP.2316 | | TTTTCAGTCAGTGACATCTGTC | | TGCTGTTGACAGTGAGCGAACAGATGTCACTGACTGAAAATAGTGAAGCCACAGATGTATTTTCAGTCAGTGACATCTGTCTGCCTACTGCCTCGGA |
| **DAB2IP** | DAB2IP.411 | | TTGTGGAACTCGAAGTGCTCGC | | TGCTGTTGACAGTGAGCGACGAGCACTTCGAGTTCCACAATAGTGAAGCCACAGATGTATTGTGGAACTCGAAGTGCTCGCTGCCTACTGCCTCGGA |
| **DAB2IP** | DAB2IP.957 | | TACAGCGCTTTGATGAACTCAC | | TGCTGTTGACAGTGAGCGATGAGTTCATCAAAGCGCTGTATAGTGAAGCCACAGATGTATACAGCGCTTTGATGAACTCACTGCCTACTGCCTCGGA |
| **DDX54** | DDX54.301 | | TTCTTCTTCTTCTTGTTCTGGG | | TGCTGTTGACAGTGAGCGACCAGAACAAGAAGAAGAAGAATAGTGAAGCCACAGATGTATTCTTCTTCTTCTTGTTCTGGGTGCCTACTGCCTCGGA |
| **DDX54** | DDX54.421 | | TTGCCATCCAAGATCACCGGGA | | TGCTGTTGACAGTGAGCGCCCCGGTGATCTTGGATGGCAATAGTGAAGCCACAGATGTATTGCCATCCAAGATCACCGGGATGCCTACTGCCTCGGA |
| **DDX54** | DDX54.2274 | | TAATCTTCTTCTTGTCTTCCTG | | TGCTGTTGACAGTGAGCGAAGGAAGACAAGAAGAAGATTATAGTGAAGCCACAGATGTATAATCTTCTTCTTGTCTTCCTGTGCCTACTGCCTCGGA |
| **DDX54** | DDX54.1756 | | TTGATCTCAAAGATAGTCGCCC | | TGCTGTTGACAGTGAGCGAGGCGACTATCTTTGAGATCAATAGTGAAGCCACAGATGTATTGATCTCAAAGATAGTCGCCCTGCCTACTGCCTCGGA |
| **DDX54** | DDX54.2269 | | TTCTTCTTGTCTTCCTGTCCTG | | TGCTGTTGACAGTGAGCGAAGGACAGGAAGACAAGAAGAATAGTGAAGCCACAGATGTATTCTTCTTGTCTTCCTGTCCTGTGCCTACTGCCTCGGA |
| **DEK** | DEK.2225 | | TTAGTCATAATCGTGAAGCTGG | | TGCTGTTGACAGTGAGCGACAGCTTCACGATTATGACTAATAGTGAAGCCACAGATGTATTAGTCATAATCGTGAAGCTGGTGCCTACTGCCTCGGA |
| **DEK** | DEK.398 | | TAGATTTCTAAGTTCATCGGTT | | TGCTGTTGACAGTGAGCGCACCGATGAACTTAGAAATCTATAGTGAAGCCACAGATGTATAGATTTCTAAGTTCATCGGTTTGCCTACTGCCTCGGA |
| **DEK** | DEK.448 | | TTAATGAGGACACAGTGCCTGG | | TGCTGTTGACAGTGAGCGACAGGCACTGTGTCCTCATTAATAGTGAAGCCACAGATGTATTAATGAGGACACAGTGCCTGGTGCCTACTGCCTCGGA |
| **DEK** | DEK.508 | | TTTTATATTGGACACTTCCTTT | | TGCTGTTGACAGTGAGCGCAAGGAAGTGTCCAATATAAAATAGTGAAGCCACAGATGTATTTTATATTGGACACTTCCTTTTGCCTACTGCCTCGGA |
| **DEK** | DEK.829 | | TTTCTTTATCTTCATCATCTGA | | TGCTGTTGACAGTGAGCGCCAGATGATGAAGATAAAGAAATAGTGAAGCCACAGATGTATTTCTTTATCTTCATCATCTGATGCCTACTGCCTCGGA |
| **DNAJC2** | DNAJC2.1165 | | TTCTTTAGCTTCTTGCTCCTTC | | TGCTGTTGACAGTGAGCGAAAGGAGCAAGAAGCTAAAGAATAGTGAAGCCACAGATGTATTCTTTAGCTTCTTGCTCCTTCTGCCTACTGCCTCGGA |
| **DNAJC2** | DNAJC2.1455 | | TAATTGTAGATCATCTTCTGAC | | TGCTGTTGACAGTGAGCGATCAGAAGATGATCTACAATTATAGTGAAGCCACAGATGTATAATTGTAGATCATCTTCTGACTGCCTACTGCCTCGGA |
| **DNAJC2** | DNAJC2.1157 | | TTAGCTTCTTGCTCCTTCCGTT | | TGCTGTTGACAGTGAGCGCACGGAAGGAGCAAGAAGCTAATAGTGAAGCCACAGATGTATTAGCTTCTTGCTCCTTCCGTTTGCCTACTGCCTCGGA |
| **DNAJC2** | DNAJC2.429 | | TTCATCTTCTGATTCCTCGGAT | | TGCTGTTGACAGTGAGCGCTCCGAGGAATCAGAAGATGAATAGTGAAGCCACAGATGTATTCATCTTCTGATTCCTCGGATTGCCTACTGCCTCGGA |
| **DNAJC2** | DNAJC2.1466 | | TTAGTAATTGTAGATCATCTTC | | TGCTGTTGACAGTGAGCGAAAGATGATCTACAATTACTAATAGTGAAGCCACAGATGTATTAGTAATTGTAGATCATCTTCTGCCTACTGCCTCGGA |
| **DNMT1** | DNMT1.884 | | TTCATCTCTTTCTTCTTCCTTT | | TGCTGTTGACAGTGAGCGCAAGGAAGAAGAAAGAGATGAATAGTGAAGCCACAGATGTATTCATCTCTTTCTTCTTCCTTTTGCCTACTGCCTCGGA |
| **DNMT1** | DNMT1.3991 | | TTGAAGGAGACAAAGTTCCTGA | | TGCTGTTGACAGTGAGCGCCAGGAACTTTGTCTCCTTCAATAGTGAAGCCACAGATGTATTGAAGGAGACAAAGTTCCTGATGCCTACTGCCTCGGA |
| **DNMT1** | DNMT1.3205 | | TTTGATGTCAGTCTCATTGGGC | | TGCTGTTGACAGTGAGCGACCCAATGAGACTGACATCAAATAGTGAAGCCACAGATGTATTTGATGTCAGTCTCATTGGGCTGCCTACTGCCTCGGA |
| **DNMT1** | DNMT1.2410 | | TTATAGTAACTCTTCTTCCCAT | | TGCTGTTGACAGTGAGCGCTGGGAAGAAGAGTTACTATAATAGTGAAGCCACAGATGTATTATAGTAACTCTTCTTCCCATTGCCTACTGCCTCGGA |
| **DNMT1** | DNMT1.2839 | | TTGAACTTGTTGTCCTCTGTTG | | TGCTGTTGACAGTGAGCGAAACAGAGGACAACAAGTTCAATAGTGAAGCCACAGATGTATTGAACTTGTTGTCCTCTGTTGTGCCTACTGCCTCGGA |
| **DR1** | DR1.1454 | | ATTGATATTATACTGAACCTTA | | TGCTGTTGACAGTGAGCGCAAGGTTCAGTATAATATCAATTAGTGAAGCCACAGATGTAATTGATATTATACTGAACCTTATGCCTACTGCCTCGGA |
| **DR1** | DR1.1779 | | TAAACAAGTATATTGATCTGAG | | TGCTGTTGACAGTGAGCGATCAGATCAATATACTTGTTTATAGTGAAGCCACAGATGTATAAACAAGTATATTGATCTGAGTGCCTACTGCCTCGGA |
| **DR1** | DR1.2938 | | TTAACTTATCGTTCTAGACTAG | | TGCTGTTGACAGTGAGCGATAGTCTAGAACGATAAGTTAATAGTGAAGCCACAGATGTATTAACTTATCGTTCTAGACTAGTGCCTACTGCCTCGGA |
| **DR1** | DR1.2574 | | TATCTTAAGTGACTTTTCCTTT | | TGCTGTTGACAGTGAGCGCAAGGAAAAGTCACTTAAGATATAGTGAAGCCACAGATGTATATCTTAAGTGACTTTTCCTTTTGCCTACTGCCTCGGA |
| **DR1** | DR1.766 | | TTTATTGATAGCAGCTCTGGGG | | TGCTGTTGACAGTGAGCGACCCAGAGCTGCTATCAATAAATAGTGAAGCCACAGATGTATTTATTGATAGCAGCTCTGGGGTGCCTACTGCCTCGGA |
| **EGR1** | EGR1.2823 | | TTTTGTTTTCTTACATTCTGGA | | TGCTGTTGACAGTGAGCGCCCAGAATGTAAGAAAACAAAATAGTGAAGCCACAGATGTATTTTGTTTTCTTACATTCTGGATGCCTACTGCCTCGGA |
| **EGR1** | EGR1.2815 | | TCTTACATTCTGGAGAACCGAA | | TGCTGTTGACAGTGAGCGCTCGGTTCTCCAGAATGTAAGATAGTGAAGCCACAGATGTATCTTACATTCTGGAGAACCGAATGCCTACTGCCTCGGA |
| **EGR1** | EGR1.2822 | | TTTGTTTTCTTACATTCTGGAG | | TGCTGTTGACAGTGAGCGATCCAGAATGTAAGAAAACAAATAGTGAAGCCACAGATGTATTTGTTTTCTTACATTCTGGAGTGCCTACTGCCTCGGA |
| **EGR1** | EGR1.2946 | | TAACATACAAAAATCGCCGCCT | | TGCTGTTGACAGTGAGCGCGGCGGCGATTTTTGTATGTTATAGTGAAGCCACAGATGTATAACATACAAAAATCGCCGCCTTGCCTACTGCCTCGGA |
| **EGR1** | EGR1.2525 | | TAACGGAACAACACTCTGACAC | | TGCTGTTGACAGTGAGCGATGTCAGAGTGTTGTTCCGTTATAGTGAAGCCACAGATGTATAACGGAACAACACTCTGACACTGCCTACTGCCTCGGA |
| **ENO1** | ENO1.261 | | TTGATGACATTGAACGCCGGGA | | TGCTGTTGACAGTGAGCGCCCCGGCGTTCAATGTCATCAATAGTGAAGCCACAGATGTATTGATGACATTGAACGCCGGGATGCCTACTGCCTCGGA |
| **ENO1** | ENO1.1440 | | TTTGAGCACAAAACCACCGGGG | | TGCTGTTGACAGTGAGCGACCCGGTGGTTTTGTGCTCAAATAGTGAAGCCACAGATGTATTTGAGCACAAAACCACCGGGGTGCCTACTGCCTCGGA |
| **ENO1** | ENO1.1437 | | TTTTGAGCACAAAACCACCGGG | | TGCTGTTGACAGTGAGCGACCGGTGGTTTTGTGCTCAAAATAGTGAAGCCACAGATGTATTTTGAGCACAAAACCACCGGGTGCCTACTGCCTCGGA |
| **ENO1** | ENO1.1441 | | TTTATTTTGAGCACAAAACCAC | | TGCTGTTGACAGTGAGCGATGGTTTTGTGCTCAAAATAAATAGTGAAGCCACAGATGTATTTATTTTGAGCACAAAACCACTGCCTACTGCCTCGGA |
| **ENO1** | ENO1.681 | | TCTTCGATAGACACCACTGGGT | | TGCTGTTGACAGTGAGCGCCCCAGTGGTGTCTATCGAAGATAGTGAAGCCACAGATGTATCTTCGATAGACACCACTGGGTTGCCTACTGCCTCGGA |
| **EREG** | EREG.1324 | | TAGATGAGTGACTAGTACCTGT | | TGCTGTTGACAGTGAGCGCCAGGTACTAGTCACTCATCTATAGTGAAGCCACAGATGTATAGATGAGTGACTAGTACCTGTTGCCTACTGCCTCGGA |
| **EREG** | EREG.1188 | | TAGTGTTTAACACAGGACCTAT | | TGCTGTTGACAGTGAGCGCTAGGTCCTGTGTTAAACACTATAGTGAAGCCACAGATGTATAGTGTTTAACACAGGACCTATTGCCTACTGCCTCGGA |
| **EREG** | EREG.3002 | | TAACCTATTCACATCCTCCTGT | | TGCTGTTGACAGTGAGCGCCAGGAGGATGTGAATAGGTTATAGTGAAGCCACAGATGTATAACCTATTCACATCCTCCTGTTGCCTACTGCCTCGGA |
| **EREG** | EREG.3718 | | TTATAGAACTTAATATTCCTGG | | TGCTGTTGACAGTGAGCGACAGGAATATTAAGTTCTATAATAGTGAAGCCACAGATGTATTATAGAACTTAATATTCCTGGTGCCTACTGCCTCGGA |
| **EREG** | EREG.993 | | TTGCTAACAATTCTTGAGCTAT | | TGCTGTTGACAGTGAGCGCTAGCTCAAGAATTGTTAGCAATAGTGAAGCCACAGATGTATTGCTAACAATTCTTGAGCTATTGCCTACTGCCTCGGA |
| **ESPL1** | ESPL1.267 | | TAGCTTAGCAGTCAGCTGCTGG | | TGCTGTTGACAGTGAGCGACAGCAGCTGACTGCTAAGCTATAGTGAAGCCACAGATGTATAGCTTAGCAGTCAGCTGCTGGTGCCTACTGCCTCGGA |
| **ESPL1** | ESPL1.1362 | | TACAACTGTCCACTAGTTGGGT | | TGCTGTTGACAGTGAGCGCCCCAACTAGTGGACAGTTGTATAGTGAAGCCACAGATGTATACAACTGTCCACTAGTTGGGTTGCCTACTGCCTCGGA |
| **ESPL1** | ESPL1.1897 | | TCACAGATGATGTTGAAGCGTT | | TGCTGTTGACAGTGAGCGCACGCTTCAACATCATCTGTGATAGTGAAGCCACAGATGTATCACAGATGATGTTGAAGCGTTTGCCTACTGCCTCGGA |
| **ESPL1** | ESPL1.1771 | | TTTAGCTGTAGCTCCTTGTCTC | | TGCTGTTGACAGTGAGCGAAGACAAGGAGCTACAGCTAAATAGTGAAGCCACAGATGTATTTAGCTGTAGCTCCTTGTCTCTGCCTACTGCCTCGGA |
| **ESPL1** | ESPL1.2669 | | TTGACTTCGAAGCAGATCACAG | | TGCTGTTGACAGTGAGCGATGTGATCTGCTTCGAAGTCAATAGTGAAGCCACAGATGTATTGACTTCGAAGCAGATCACAGTGCCTACTGCCTCGGA |
| **ETV4** | ETV4.686 | | TTCATTTATATGTACACAGGGC | | TGCTGTTGACAGTGAGCGACCCTGTGTACATATAAATGAATAGTGAAGCCACAGATGTATTCATTTATATGTACACAGGGCTGCCTACTGCCTCGGA |
| **ETV4** | ETV4.380 | | TAATAGTATCGGAGCGAGCGGC | | TGCTGTTGACAGTGAGCGACCGCTCGCTCCGATACTATTATAGTGAAGCCACAGATGTATAATAGTATCGGAGCGAGCGGCTGCCTACTGCCTCGGA |
| **ETV4** | ETV4.806 | | TTTCCTTCCCAATGACTCCGGT | | TGCTGTTGACAGTGAGCGCCCGGAGTCATTGGGAAGGAAATAGTGAAGCCACAGATGTATTTCCTTCCCAATGACTCCGGTTGCCTACTGCCTCGGA |
| **ETV4** | ETV4.385 | | TCTCATAATAGTATCGGAGCGA | | TGCTGTTGACAGTGAGCGCCGCTCCGATACTATTATGAGATAGTGAAGCCACAGATGTATCTCATAATAGTATCGGAGCGATGCCTACTGCCTCGGA |
| **ETV4** | ETV4.816 | | TTTCTCCACTTTTCCTTCCCAA | | TGCTGTTGACAGTGAGCGCTGGGAAGGAAAAGTGGAGAAATAGTGAAGCCACAGATGTATTTCTCCACTTTTCCTTCCCAATGCCTACTGCCTCGGA |
| **ETV5** | ETV5.390 | | TTGAAGTTGACTGAGATCCTGA | | TGCTGTTGACAGTGAGCGCCAGGATCTCAGTCAACTTCAATAGTGAAGCCACAGATGTATTGAAGTTGACTGAGATCCTGATGCCTACTGCCTCGGA |
| **ETV5** | ETV5.3563 | | TTTGATTAGAGTACAATGCTAA | | TGCTGTTGACAGTGAGCGCTAGCATTGTACTCTAATCAAATAGTGAAGCCACAGATGTATTTGATTAGAGTACAATGCTAATGCCTACTGCCTCGGA |
| **ETV5** | ETV5.2355 | | TATGATTTTGAGAACCACGGAG | | TGCTGTTGACAGTGAGCGATCCGTGGTTCTCAAAATCATATAGTGAAGCCACAGATGTATATGATTTTGAGAACCACGGAGTGCCTACTGCCTCGGA |
| **ETV5** | ETV5.3085 | | TTACCTGTCAGTATCACACGTA | | TGCTGTTGACAGTGAGCGCACGTGTGATACTGACAGGTAATAGTGAAGCCACAGATGTATTACCTGTCAGTATCACACGTATGCCTACTGCCTCGGA |
| **ETV5** | ETV5.2130 | | TAGTAGTCCATGATCGATGCAG | | TGCTGTTGACAGTGAGCGATGCATCGATCATGGACTACTATAGTGAAGCCACAGATGTATAGTAGTCCATGATCGATGCAGTGCCTACTGCCTCGGA |
| **EZH2** | EZH2.292 | | TTCAATGAAAGTACCATCCTGA | | TGCTGTTGACAGTGAGCGCCAGGATGGTACTTTCATTGAATAGTGAAGCCACAGATGTATTCAATGAAAGTACCATCCTGATGCCTACTGCCTCGGA |
| **EZH2** | EZH2.578 | | TTTCCTTTAGTTCTTCTGCTGT | | TGCTGTTGACAGTGAGCGCCAGCAGAAGAACTAAAGGAAATAGTGAAGCCACAGATGTATTTCCTTTAGTTCTTCTGCTGTTGCCTACTGCCTCGGA |
| **EZH2** | EZH2.1100 | | TTTATTGGTGTTTGACACCGAG | | TGCTGTTGACAGTGAGCGATCGGTGTCAAACACCAATAAATAGTGAAGCCACAGATGTATTTATTGGTGTTTGACACCGAGTGCCTACTGCCTCGGA |
| **EZH2** | EZH2.1112 | | TTTGGCTTCATCTTTATTGGTG | | TGCTGTTGACAGTGAGCGAACCAATAAAGATGAAGCCAAATAGTGAAGCCACAGATGTATTTGGCTTCATCTTTATTGGTGTGCCTACTGCCTCGGA |
| **EZH2** | EZH2.1700 | | TATTTATCATACACTTTCCCTC | | TGCTGTTGACAGTGAGCGAAGGGAAAGTGTATGATAAATATAGTGAAGCCACAGATGTATATTTATCATACACTTTCCCTCTGCCTACTGCCTCGGA |
| **FOS** | FOS.1690 | | TTAATTCCAATAATGAACCCAA | | TGCTGTTGACAGTGAGCGCTGGGTTCATTATTGGAATTAATAGTGAAGCCACAGATGTATTAATTCCAATAATGAACCCAATGCCTACTGCCTCGGA |
| **FOS** | FOS.1804 | | TTTTCTTAGTATAATATTGGTC | | TGCTGTTGACAGTGAGCGAACCAATATTATACTAAGAAAATAGTGAAGCCACAGATGTATTTTCTTAGTATAATATTGGTCTGCCTACTGCCTCGGA |
| **FOS** | FOS.1894 | | TAACATTACAATGAACATTGAT | | TGCTGTTGACAGTGAGCGCTCAATGTTCATTGTAATGTTATAGTGAAGCCACAGATGTATAACATTACAATGAACATTGATTGCCTACTGCCTCGGA |
| **FOS** | FOS.2115 | | TTTTATTGACAATGTCTTGGAA | | TGCTGTTGACAGTGAGCGCTCCAAGACATTGTCAATAAAATAGTGAAGCCACAGATGTATTTTATTGACAATGTCTTGGAATGCCTACTGCCTCGGA |
| **FOS** | FOS.703 | | TCTAGTTGGTCTGTCTCCGCTT | | TGCTGTTGACAGTGAGCGCAGCGGAGACAGACCAACTAGATAGTGAAGCCACAGATGTATCTAGTTGGTCTGTCTCCGCTTTGCCTACTGCCTCGGA |
| **FOSL1** | FOSL1.1280 | | TAAGGATCTACAAAGTCTCTGG | | TGCTGTTGACAGTGAGCGACAGAGACTTTGTAGATCCTTATAGTGAAGCCACAGATGTATAAGGATCTACAAAGTCTCTGGTGCCTACTGCCTCGGA |
| **FOSL1** | FOSL1.90 | | TATGAATGAAAAGTTCTCGGGC | | TGCTGTTGACAGTGAGCGACCCGAGAACTTTTCATTCATATAGTGAAGCCACAGATGTATATGAATGAAAAGTTCTCGGGCTGCCTACTGCCTCGGA |
| **FOSL1** | FOSL1.1634 | | TTTTATTCCATTTTGGTAGGTT | | TGCTGTTGACAGTGAGCGCACCTACCAAAATGGAATAAAATAGTGAAGCCACAGATGTATTTTATTCCATTTTGGTAGGTTTGCCTACTGCCTCGGA |
| **FOSL1** | FOSL1.91 | | TTATGAATGAAAAGTTCTCGGG | | TGCTGTTGACAGTGAGCGACCGAGAACTTTTCATTCATAATAGTGAAGCCACAGATGTATTATGAATGAAAAGTTCTCGGGTGCCTACTGCCTCGGA |
| **FOSL1** | FOSL1.1405 | | TGAGTTAGTGTTCTAGGTGGGT | | TGCTGTTGACAGTGAGCGCCCCACCTAGAACACTAACTCATAGTGAAGCCACAGATGTATGAGTTAGTGTTCTAGGTGGGTTGCCTACTGCCTCGGA |
| **FOXM1** | FOXM1.1812 | | TTGAATCACAAGCATTTCCGAG | | TGCTGTTGACAGTGAGCGATCGGAAATGCTTGTGATTCAATAGTGAAGCCACAGATGTATTGAATCACAAGCATTTCCGAGTGCCTACTGCCTCGGA |
| **FOXM1** | FOXM1.725 | | TCTAGGAAGATTCACATCCCTA | | TGCTGTTGACAGTGAGCGCAGGGATGTGAATCTTCCTAGATAGTGAAGCCACAGATGTATCTAGGAAGATTCACATCCCTATGCCTACTGCCTCGGA |
| **FOXM1** | FOXM1.1277 | | TTGATGGTCATGTTCCGGCGGA | | TGCTGTTGACAGTGAGCGCCCGCCGGAACATGACCATCAATAGTGAAGCCACAGATGTATTGATGGTCATGTTCCGGCGGATGCCTACTGCCTCGGA |
| **FOXM1** | FOXM1.2496 | | TAGCTCAGGAATAAACTGGGAC | | TGCTGTTGACAGTGAGCGATCCCAGTTTATTCCTGAGCTATAGTGAAGCCACAGATGTATAGCTCAGGAATAAACTGGGACTGCCTACTGCCTCGGA |
| **FOXM1** | FOXM1.463 | | TGGTTAATAATCTTGATCCCAG | | TGCTGTTGACAGTGAGCGATGGGATCAAGATTATTAACCATAGTGAAGCCACAGATGTATGGTTAATAATCTTGATCCCAGTGCCTACTGCCTCGGA |
| **FUBP1** | FUBP1.2714 | | TATAGCAGCAGTACAGGTCTGA | | TGCTGTTGACAGTGAGCGCCAGACCTGTACTGCTGCTATATAGTGAAGCCACAGATGTATATAGCAGCAGTACAGGTCTGATGCCTACTGCCTCGGA |
| **FUBP1** | FUBP1.1846 | | TTTCTTGTAGTACTCTTCCCAA | | TGCTGTTGACAGTGAGCGCTGGGAAGAGTACTACAAGAAATAGTGAAGCCACAGATGTATTTCTTGTAGTACTCTTCCCAATGCCTACTGCCTCGGA |
| **FUBP1** | FUBP1.521 | | TTAACATACAGGACCTTTCTGG | | TGCTGTTGACAGTGAGCGACAGAAAGGTCCTGTATGTTAATAGTGAAGCCACAGATGTATTAACATACAGGACCTTTCTGGTGCCTACTGCCTCGGA |
| **FUBP1** | FUBP1.236 | | TTGAATTCAGTGATGTCCCTGC | | TGCTGTTGACAGTGAGCGACAGGGACATCACTGAATTCAATAGTGAAGCCACAGATGTATTGAATTCAGTGATGTCCCTGCTGCCTACTGCCTCGGA |
| **FUBP1** | FUBP1.1932 | | TTGTCTATAATACTCAGCCCAG | | TGCTGTTGACAGTGAGCGATGGGCTGAGTATTATAGACAATAGTGAAGCCACAGATGTATTGTCTATAATACTCAGCCCAGTGCCTACTGCCTCGGA |
| **FUS** | FUS.212 | | ATAACCACTGTAACTCTGCTGT | | TGCTGTTGACAGTGAGCGCCAGCAGAGTTACAGTGGTTATTAGTGAAGCCACAGATGTAATAACCACTGTAACTCTGCTGTTGCCTACTGCCTCGGA |
| **FUS** | FUS.2807 | | TTGAGAGGAAAGCACTTCCCAA | | TGCTGTTGACAGTGAGCGCTGGGAAGTGCTTTCCTCTCAATAGTGAAGCCACAGATGTATTGAGAGGAAAGCACTTCCCAATGCCTACTGCCTCGGA |
| **FUS** | FUS.960 | | TTGTTGTCTGAATTATCCTGTT | | TGCTGTTGACAGTGAGCGCACAGGATAATTCAGACAACAATAGTGAAGCCACAGATGTATTGTTGTCTGAATTATCCTGTTTGCCTACTGCCTCGGA |
| **FUS** | FUS.1050 | | TTGTTTGTCTTAATAATACCAA | | TGCTGTTGACAGTGAGCGCTGGTATTATTAAGACAAACAATAGTGAAGCCACAGATGTATTGTTTGTCTTAATAATACCAATGCCTACTGCCTCGGA |
| **FUS** | FUS.1028 | | TCTGCTTGAAGTAATCAGCCAC | | TGCTGTTGACAGTGAGCGATGGCTGATTACTTCAAGCAGATAGTGAAGCCACAGATGTATCTGCTTGAAGTAATCAGCCACTGCCTACTGCCTCGGA |
| **HMGA1** | HMGA1.211 | | TTTTGCTTCCCTTTGGTCGGCC | | TGCTGTTGACAGTGAGCGAGCCGACCAAAGGGAAGCAAAATAGTGAAGCCACAGATGTATTTTGCTTCCCTTTGGTCGGCCTGCCTACTGCCTCGGA |
| **HMGA1** | HMGA1.155 | | TCCTTGAATTCCTCGAGCGGAG | | TGCTGTTGACAGTGAGCGATCCGCTCGAGGAATTCAAGGATAGTGAAGCCACAGATGTATCCTTGAATTCCTCGAGCGGAGTGCCTACTGCCTCGGA |
| **HMGA1** | HMGA1.40 | | TCATCTTCCCTTCTCTAAGGAG | | TGCTGTTGACAGTGAGCGATCCTTAGAGAAGGGAAGATGATAGTGAAGCCACAGATGTATCATCTTCCCTTCTCTAAGGAGTGCCTACTGCCTCGGA |
| **HMGA1** | HMGA1.1061 | | TATGTACTCAGATCCCAGGCGG | | TGCTGTTGACAGTGAGCGACGCCTGGGATCTGAGTACATATAGTGAAGCCACAGATGTATATGTACTCAGATCCCAGGCGGTGCCTACTGCCTCGGA |
| **HMGA1** | HMGA1.1391 | | TGAGGATGAACATTTGGCGCTG | | TGCTGTTGACAGTGAGCGAAGCGCCAAATGTTCATCCTCATAGTGAAGCCACAGATGTATGAGGATGAACATTTGGCGCTGTGCCTACTGCCTCGGA |
| **HMGB1** | HMGB1.2309 | | TTAACTAGTATTTAAAACCTCT | | TGCTGTTGACAGTGAGCGCGAGGTTTTAAATACTAGTTAATAGTGAAGCCACAGATGTATTAACTAGTATTTAAAACCTCTTGCCTACTGCCTCGGA |
| **HMGB1** | HMGB1.2683 | | TACAGTAGAAACTTCCATCTAA | | TGCTGTTGACAGTGAGCGCTAGATGGAAGTTTCTACTGTATAGTGAAGCCACAGATGTATACAGTAGAAACTTCCATCTAATGCCTACTGCCTCGGA |
| **HMGB1** | HMGB1.1726 | | TTGTATTTTAAGCTCACGCTTT | | TGCTGTTGACAGTGAGCGCAAGCGTGAGCTTAAAATACAATAGTGAAGCCACAGATGTATTGTATTTTAAGCTCACGCTTTTGCCTACTGCCTCGGA |
| **HMGB1** | HMGB1.1534 | | TTGAGTAGATTGATTACTCTTC | | TGCTGTTGACAGTGAGCGAAAGAGTAATCAATCTACTCAATAGTGAAGCCACAGATGTATTGAGTAGATTGATTACTCTTCTGCCTACTGCCTCGGA |
| **HMGB1** | HMGB1.2936 | | TTAGACATCCAACTTCTAGGGG | | TGCTGTTGACAGTGAGCGACCCTAGAAGTTGGATGTCTAATAGTGAAGCCACAGATGTATTAGACATCCAACTTCTAGGGGTGCCTACTGCCTCGGA |
| **HMGB2** | HMGB2.635 | | TTATTCTTCATCTTCATCCTCT | | TGCTGTTGACAGTGAGCGCGAGGATGAAGATGAAGAATAATAGTGAAGCCACAGATGTATTATTCTTCATCTTCATCCTCTTGCCTACTGCCTCGGA |
| **HMGB2** | HMGB2.1295 | | TTAGCTAATAAACAGAAACGTC | | TGCTGTTGACAGTGAGCGAACGTTTCTGTTTATTAGCTAATAGTGAAGCCACAGATGTATTAGCTAATAAACAGAAACGTCTGCCTACTGCCTCGGA |
| **HMGB2** | HMGB2.102 | | TTCTTCTTGTGCTCTTCCCGGC | | TGCTGTTGACAGTGAGCGACCGGGAAGAGCACAAGAAGAATAGTGAAGCCACAGATGTATTCTTCTTGTGCTCTTCCCGGCTGCCTACTGCCTCGGA |
| **HMGB2** | HMGB2.952 | | TTACTATTGATACTAATTCCTA | | TGCTGTTGACAGTGAGCGCAGGAATTAGTATCAATAGTAATAGTGAAGCCACAGATGTATTACTATTGATACTAATTCCTATGCCTACTGCCTCGGA |
| **HMGB2** | HMGB2.849 | | TCTAACTGTATGAGTAGCCCAT | | TGCTGTTGACAGTGAGCGCTGGGCTACTCATACAGTTAGATAGTGAAGCCACAGATGTATCTAACTGTATGAGTAGCCCATTGCCTACTGCCTCGGA |
| **HMGB3** | HMGB3.2264 | | TTAACATTGAACATCAATCTAC | | TGCTGTTGACAGTGAGCGATAGATTGATGTTCAATGTTAATAGTGAAGCCACAGATGTATTAACATTGAACATCAATCTACTGCCTACTGCCTCGGA |
| **HMGB3** | HMGB3.1909 | | TTTGACACACCATACACTCTGA | | TGCTGTTGACAGTGAGCGCCAGAGTGTATGGTGTGTCAAATAGTGAAGCCACAGATGTATTTGACACACCATACACTCTGATGCCTACTGCCTCGGA |
| **HMGB3** | HMGB3.628 | | TTCATCTTCCTCTTCCACCTTT | | TGCTGTTGACAGTGAGCGCAAGGTGGAAGAGGAAGATGAATAGTGAAGCCACAGATGTATTCATCTTCCTCTTCCACCTTTTGCCTACTGCCTCGGA |
| **HMGB3** | HMGB3.1249 | | TACAGAAACAAGACAACCTGAA | | TGCTGTTGACAGTGAGCGCTCAGGTTGTCTTGTTTCTGTATAGTGAAGCCACAGATGTATACAGAAACAAGACAACCTGAATGCCTACTGCCTCGGA |
| **HMGB3** | HMGB3.2531 | | TAATAGCACAAAAACACTCCTG | | TGCTGTTGACAGTGAGCGAAGGAGTGTTTTTGTGCTATTATAGTGAAGCCACAGATGTATAATAGCACAAAAACACTCCTGTGCCTACTGCCTCGGA |
| **HNRNPAB** | HNRNPAB.1243 | | TTAAATAAGATGCACATGGGAC | | TGCTGTTGACAGTGAGCGATCCCATGTGCATCTTATTTAATAGTGAAGCCACAGATGTATTAAATAAGATGCACATGGGACTGCCTACTGCCTCGGA |
| **HNRNPAB** | HNRNPAB.608 | | TCTTTGAACAGGATAAACCCAA | | TGCTGTTGACAGTGAGCGCTGGGTTTATCCTGTTCAAAGATAGTGAAGCCACAGATGTATCTTTGAACAGGATAAACCCAATGCCTACTGCCTCGGA |
| **HNRNPAB** | HNRNPAB.829 | | TTTTGTTCAACTTTGGATCCAT | | TGCTGTTGACAGTGAGCGCTGGATCCAAAGTTGAACAAAATAGTGAAGCCACAGATGTATTTTGTTCAACTTTGGATCCATTGCCTACTGCCTCGGA |
| **HNRNPAB** | HNRNPAB.1575 | | TTACAATACATTAGATTCCCAA | | TGCTGTTGACAGTGAGCGCTGGGAATCTAATGTATTGTAATAGTGAAGCCACAGATGTATTACAATACATTAGATTCCCAATGCCTACTGCCTCGGA |
| **HNRNPAB** | HNRNPAB.1063 | | TTGTACTACCGAATTCCTCGAG | | TGCTGTTGACAGTGAGCGATCGAGGAATTCGGTAGTACAATAGTGAAGCCACAGATGTATTGTACTACCGAATTCCTCGAGTGCCTACTGCCTCGGA |
| **HNRNPD** | HNRNPD.1275 | | TTGAACTGCTATTAGCAGGTGG | | TGCTGTTGACAGTGAGCGACACCTGCTAATAGCAGTTCAATAGTGAAGCCACAGATGTATTGAACTGCTATTAGCAGGTGGTGCCTACTGCCTCGGA |
| **HNRNPD** | HNRNPD.2045 | | TATATTTCTTTAATCCTCCCTC | | TGCTGTTGACAGTGAGCGAAGGGAGGATTAAAGAAATATATAGTGAAGCCACAGATGTATATATTTCTTTAATCCTCCCTCTGCCTACTGCCTCGGA |
| **HNRNPD** | HNRNPD.774 | | TTAGGATCAATCACCTTCCCAT | | TGCTGTTGACAGTGAGCGCTGGGAAGGTGATTGATCCTAATAGTGAAGCCACAGATGTATTAGGATCAATCACCTTCCCATTGCCTACTGCCTCGGA |
| **HNRNPD** | HNRNPD.988 | | TTCCATTATCTTCTTCACTGGT | | TGCTGTTGACAGTGAGCGCCCAGTGAAGAAGATAATGGAATAGTGAAGCCACAGATGTATTCCATTATCTTCTTCACTGGTTGCCTACTGCCTCGGA |
| **HNRNPD** | HNRNPD.1060 | | TTGCTGATATTGTTCCTTCGAC | | TGCTGTTGACAGTGAGCGATCGAAGGAACAATATCAGCAATAGTGAAGCCACAGATGTATTGCTGATATTGTTCCTTCGACTGCCTACTGCCTCGGA |
| **HNRNPR** | HNRNPR.2400 | | TTTAAAAACACAGTTAACCTAC | | TGCTGTTGACAGTGAGCGATAGGTTAACTGTGTTTTTAAATAGTGAAGCCACAGATGTATTTAAAAACACAGTTAACCTACTGCCTACTGCCTCGGA |
| **HNRNPR** | HNRNPR.777 | | TTCTTTAGTCTTATTCTTCGGA | | TGCTGTTGACAGTGAGCGCCCGAAGAATAAGACTAAAGAATAGTGAAGCCACAGATGTATTCTTTAGTCTTATTCTTCGGATGCCTACTGCCTCGGA |
| **HNRNPR** | HNRNPR.1892 | | TACTTGTCTACTTCCACTGTTG | | TGCTGTTGACAGTGAGCGAAACAGTGGAAGTAGACAAGTATAGTGAAGCCACAGATGTATACTTGTCTACTTCCACTGTTGTGCCTACTGCCTCGGA |
| **HNRNPR** | HNRNPR.1919 | | TTACTTGTCTACTTCCACTGTT | | TGCTGTTGACAGTGAGCGCACAGTGGAAGTAGACAAGTAATAGTGAAGCCACAGATGTATTACTTGTCTACTTCCACTGTTTGCCTACTGCCTCGGA |
| **HNRNPR** | HNRNPR.510 | | TTTGCCTACAAATACCTCCGTT | | TGCTGTTGACAGTGAGCGCACGGAGGTATTTGTAGGCAAATAGTGAAGCCACAGATGTATTTGCCTACAAATACCTCCGTTTGCCTACTGCCTCGGA |
| **IL17RD** | IL17RD.5166 | | TTTTGAAGGACAGTCTTCCTGC | | TGCTGTTGACAGTGAGCGACAGGAAGACTGTCCTTCAAAATAGTGAAGCCACAGATGTATTTTGAAGGACAGTCTTCCTGCTGCCTACTGCCTCGGA |
| **IL17RD** | IL17RD.4714 | | TAACCTATCTACACAAGCCTTT | | TGCTGTTGACAGTGAGCGCAAGGCTTGTGTAGATAGGTTATAGTGAAGCCACAGATGTATAACCTATCTACACAAGCCTTTTGCCTACTGCCTCGGA |
| **IL17RD** | IL17RD.660 | | TTTACAAGCTAGATTGTCCGGC | | TGCTGTTGACAGTGAGCGACCGGACAATCTAGCTTGTAAATAGTGAAGCCACAGATGTATTTACAAGCTAGATTGTCCGGCTGCCTACTGCCTCGGA |
| **IL17RD** | IL17RD.1052 | | TTTTCTTGTTGCTTCTTGCGGC | | TGCTGTTGACAGTGAGCGACCGCAAGAAGCAACAAGAAAATAGTGAAGCCACAGATGTATTTTCTTGTTGCTTCTTGCGGCTGCCTACTGCCTCGGA |
| **IL17RD** | IL17RD.493 | | TTTGAAGCTACTGTTGAGCTGC | | TGCTGTTGACAGTGAGCGACAGCTCAACAGTAGCTTCAAATAGTGAAGCCACAGATGTATTTGAAGCTACTGTTGAGCTGCTGCCTACTGCCTCGGA |
| **ILF2** | ILF2.527 | | TAAAACTTCAGAAGGATCCTGT | | TGCTGTTGACAGTGAGCGCCAGGATCCTTCTGAAGTTTTATAGTGAAGCCACAGATGTATAAAACTTCAGAAGGATCCTGTTGCCTACTGCCTCGGA |
| **ILF2** | ILF2.45 | | TAGCAGACAACTGAAGAGGCGT | | TGCTGTTGACAGTGAGCGCCGCCTCTTCAGTTGTCTGCTATAGTGAAGCCACAGATGTATAGCAGACAACTGAAGAGGCGTTGCCTACTGCCTCGGA |
| **ILF2** | ILF2.1584 | | TTTCACAACATTTCAACAGCAA | | TGCTGTTGACAGTGAGCGCTGCTGTTGAAATGTTGTGAAATAGTGAAGCCACAGATGTATTTCACAACATTTCAACAGCAATGCCTACTGCCTCGGA |
| **ILF2** | ILF2.1851 | | TGATATTCTAGTTTACTCTGGT | | TGCTGTTGACAGTGAGCGCCCAGAGTAAACTAGAATATCATAGTGAAGCCACAGATGTATGATATTCTAGTTTACTCTGGTTGCCTACTGCCTCGGA |
| **ILF2** | ILF2.785 | | TCTGATGAGAACTTTAACTGTG | | TGCTGTTGACAGTGAGCGAACAGTTAAAGTTCTCATCAGATAGTGAAGCCACAGATGTATCTGATGAGAACTTTAACTGTGTGCCTACTGCCTCGGA |
| **ILF3** | ILF3.2024 | | TTTTGTTGGAACCAGCACCTTG | | TGCTGTTGACAGTGAGCGAAAGGTGCTGGTTCCAACAAAATAGTGAAGCCACAGATGTATTTTGTTGGAACCAGCACCTTGTGCCTACTGCCTCGGA |
| **ILF3** | ILF3.1480 | | TTTCTTCTGAATCTTCTTCTTC | | TGCTGTTGACAGTGAGCGAAAGAAGAAGATTCAGAAGAAATAGTGAAGCCACAGATGTATTTCTTCTGAATCTTCTTCTTCTGCCTACTGCCTCGGA |
| **ILF3** | ILF3.724 | | TTCTGTTACAGCAGCAAGCTGG | | TGCTGTTGACAGTGAGCGACAGCTTGCTGCTGTAACAGAATAGTGAAGCCACAGATGTATTCTGTTACAGCAGCAAGCTGGTGCCTACTGCCTCGGA |
| **ILF3** | ILF3.299 | | TTACTTCTTCTGTAGTGTCTGG | | TGCTGTTGACAGTGAGCGACAGACACTACAGAAGAAGTAATAGTGAAGCCACAGATGTATTACTTCTTCTGTAGTGTCTGGTGCCTACTGCCTCGGA |
| **ILF3** | ILF3.534 | | TTCTTTACTGTCGTCCTCTGGG | | TGCTGTTGACAGTGAGCGACCAGAGGACGACAGTAAAGAATAGTGAAGCCACAGATGTATTCTTTACTGTCGTCCTCTGGGTGCCTACTGCCTCGGA |
| **IQGAP1** | IQGAP1.7193 | | TTAACATGAACAAATGACCTTT | | TGCTGTTGACAGTGAGCGCAAGGTCATTTGTTCATGTTAATAGTGAAGCCACAGATGTATTAACATGAACAAATGACCTTTTGCCTACTGCCTCGGA |
| **IQGAP1** | IQGAP1.622 | | TTCTTCTGTGAAGTCAACCTTT | | TGCTGTTGACAGTGAGCGCAAGGTTGACTTCACAGAAGAATAGTGAAGCCACAGATGTATTCTTCTGTGAAGTCAACCTTTTGCCTACTGCCTCGGA |
| **IQGAP1** | IQGAP1.5499 | | TAACATGACATTTTAGTTGTGT | | TGCTGTTGACAGTGAGCGCCACAACTAAAATGTCATGTTATAGTGAAGCCACAGATGTATAACATGACATTTTAGTTGTGTTGCCTACTGCCTCGGA |
| **IQGAP1** | IQGAP1.4793 | | TTTCATGTAGTCTTGCTGCTGT | | TGCTGTTGACAGTGAGCGCCAGCAGCAAGACTACATGAAATAGTGAAGCCACAGATGTATTTCATGTAGTCTTGCTGCTGTTGCCTACTGCCTCGGA |
| **IQGAP1** | IQGAP1.3207 | | TTGAATCTGATCTACCTTCGAC | | TGCTGTTGACAGTGAGCGATCGAAGGTAGATCAGATTCAATAGTGAAGCCACAGATGTATTGAATCTGATCTACCTTCGACTGCCTACTGCCTCGGA |
| **JUNB** | JUNB.308 | | TAGCTGTGTATGAGTCGTCGTG | | TGCTGTTGACAGTGAGCGAACGACGACTCATACACAGCTATAGTGAAGCCACAGATGTATAGCTGTGTATGAGTCGTCGTGTGCCTACTGCCTCGGA |
| **JUNB** | JUNB.273 | | TCCATTTTAGTGCACATCCGGG | | TGCTGTTGACAGTGAGCGACCGGATGTGCACTAAAATGGATAGTGAAGCCACAGATGTATCCATTTTAGTGCACATCCGGGTGCCTACTGCCTCGGA |
| **JUNB** | JUNB.1535 | | TATGAATCGAGTCTGTTTCCAG | | TGCTGTTGACAGTGAGCGATGGAAACAGACTCGATTCATATAGTGAAGCCACAGATGTATATGAATCGAGTCTGTTTCCAGTGCCTACTGCCTCGGA |
| **JUNB** | JUNB.363 | | TTCAGGAGTTTGTAGTCGTGTA | | TGCTGTTGACAGTGAGCGCACACGACTACAAACTCCTGAATAGTGAAGCCACAGATGTATTCAGGAGTTTGTAGTCGTGTATGCCTACTGCCTCGGA |
| **JUNB** | JUNB.1241 | | TGACCTTCTGTTTGAGCTGGGC | | TGCTGTTGACAGTGAGCGACCCAGCTCAAACAGAAGGTCATAGTGAAGCCACAGATGTATGACCTTCTGTTTGAGCTGGGCTGCCTACTGCCTCGGA |
| **KLF10** | KLF10.622 | | TTGCTTTCTCATCAACATCTGC | | TGCTGTTGACAGTGAGCGACAGATGTTGATGAGAAAGCAATAGTGAAGCCACAGATGTATTGCTTTCTCATCAACATCTGCTGCCTACTGCCTCGGA |
| **KLF10** | KLF10.1350 | | TAGCTTGCTCACTTCCATCTGC | | TGCTGTTGACAGTGAGCGACAGATGGAAGTGAGCAAGCTATAGTGAAGCCACAGATGTATAGCTTGCTCACTTCCATCTGCTGCCTACTGCCTCGGA |
| **KLF10** | KLF10.1822 | | TACATCTCCATGTCTGTACCGT | | TGCTGTTGACAGTGAGCGCCGGTACAGACATGGAGATGTATAGTGAAGCCACAGATGTATACATCTCCATGTCTGTACCGTTGCCTACTGCCTCGGA |
| **KLF10** | KLF10.1084 | | TATGTCTTGCCACATCCTGGGT | | TGCTGTTGACAGTGAGCGCCCCAGGATGTGGCAAGACATATAGTGAAGCCACAGATGTATATGTCTTGCCACATCCTGGGTTGCCTACTGCCTCGGA |
| **KLF10** | KLF10.1413 | | TTCTGACTCTTCACTTTCCGGT | | TGCTGTTGACAGTGAGCGCCCGGAAAGTGAAGAGTCAGAATAGTGAAGCCACAGATGTATTCTGACTCTTCACTTTCCGGTTGCCTACTGCCTCGGA |
| **LRRFIP1** | LRRFIP1.595 | | TCTTTTGTCATCTTGGTAGGAC | | TGCTGTTGACAGTGAGCGATCCTACCAAGATGACAAAAGATAGTGAAGCCACAGATGTATCTTTTGTCATCTTGGTAGGACTGCCTACTGCCTCGGA |
| **LRRFIP1** | LRRFIP1.259 | | TATTTCTCTTCAACTTCTGCTA | | TGCTGTTGACAGTGAGCGCAGCAGAAGTTGAAGAGAAATATAGTGAAGCCACAGATGTATATTTCTCTTCAACTTCTGCTATGCCTACTGCCTCGGA |
| **LRRFIP1** | LRRFIP1.445 | | TGAAATTGCAGTATACTGTGGG | | TGCTGTTGACAGTGAGCGACCACAGTATACTGCAATTTCATAGTGAAGCCACAGATGTATGAAATTGCAGTATACTGTGGGTGCCTACTGCCTCGGA |
| **LRRFIP1** | LRRFIP1.592 | | TTTGTCATCTTGGTAGGACCTT | | TGCTGTTGACAGTGAGCGCAGGTCCTACCAAGATGACAAATAGTGAAGCCACAGATGTATTTGTCATCTTGGTAGGACCTTTGCCTACTGCCTCGGA |
| **LRRFIP1** | LRRFIP1.262 | | TTATATTTCTCTTCAACTTCTG | | TGCTGTTGACAGTGAGCGAAGAAGTTGAAGAGAAATATAATAGTGAAGCCACAGATGTATTATATTTCTCTTCAACTTCTGTGCCTACTGCCTCGGA |
| **MAFF** | MAFF.970 | | TTTCACTAGTCTTCCCACCCAC | | TGCTGTTGACAGTGAGCGATGGGTGGGAAGACTAGTGAAATAGTGAAGCCACAGATGTATTTCACTAGTCTTCCCACCCACTGCCTACTGCCTCGGA |
| **MAFF** | MAFF.1993 | | TAAATAAATTCTAGGAGCCGGG | | TGCTGTTGACAGTGAGCGACCGGCTCCTAGAATTTATTTATAGTGAAGCCACAGATGTATAAATAAATTCTAGGAGCCGGGTGCCTACTGCCTCGGA |
| **MAFF** | MAFF.708 | | TAAACTTTCAGCCAAGGGCGAT | | TGCTGTTGACAGTGAGCGCTCGCCCTTGGCTGAAAGTTTATAGTGAAGCCACAGATGTATAAACTTTCAGCCAAGGGCGATTGCCTACTGCCTCGGA |
| **MAFF** | MAFF.2 | | TCGCTTGATCGAATTCCTCGAG | | TGCTGTTGACAGTGAGCGATCGAGGAATTCGATCAAGCGATAGTGAAGCCACAGATGTATCGCTTGATCGAATTCCTCGAGTGCCTACTGCCTCGGA |
| **MAFF** | MAFF.839 | | TAGCTTTGAATCCTGGGAGGGT | | TGCTGTTGACAGTGAGCGCCCCTCCCAGGATTCAAAGCTATAGTGAAGCCACAGATGTATAGCTTTGAATCCTGGGAGGGTTGCCTACTGCCTCGGA |
| **MCM2** | MCM2.715 | | TTCTCTTTGCACATGTCGCTGA | | TGCTGTTGACAGTGAGCGCCAGCGACATGTGCAAAGAGAATAGTGAAGCCACAGATGTATTCTCTTTGCACATGTCGCTGATGCCTACTGCCTCGGA |
| **MCM2** | MCM2.1397 | | TACAGCAACCTTGTTGTCCTTC | | TGCTGTTGACAGTGAGCGAAAGGACAACAAGGTTGCTGTATAGTGAAGCCACAGATGTATACAGCAACCTTGTTGTCCTTCTGCCTACTGCCTCGGA |
| **MCM2** | MCM2.1017 | | TACTTGACCATGCTGAGCTGGG | | TGCTGTTGACAGTGAGCGACCAGCTCAGCATGGTCAAGTATAGTGAAGCCACAGATGTATACTTGACCATGCTGAGCTGGGTGCCTACTGCCTCGGA |
| **MCM2** | MCM2.1427 | | TCATCTTCACATCTTCATCGGT | | TGCTGTTGACAGTGAGCGCCCGATGAAGATGTGAAGATGATAGTGAAGCCACAGATGTATCATCTTCACATCTTCATCGGTTGCCTACTGCCTCGGA |
| **MCM2** | MCM2.1302 | | TAGTTGTTGTGATAGATGCCAG | | TGCTGTTGACAGTGAGCGATGGCATCTATCACAACAACTATAGTGAAGCCACAGATGTATAGTTGTTGTGATAGATGCCAGTGCCTACTGCCTCGGA |
| **MCM3** | MCM3.246 | | TTCACATTGACAATCAGCCGGT | | TGCTGTTGACAGTGAGCGCCCGGCTGATTGTCAATGTGAATAGTGAAGCCACAGATGTATTCACATTGACAATCAGCCGGTTGCCTACTGCCTCGGA |
| **MCM3** | MCM3.957 | | TTACTGAACTTCTTGATCTTGG | | TGCTGTTGACAGTGAGCGACAAGATCAAGAAGTTCAGTAATAGTGAAGCCACAGATGTATTACTGAACTTCTTGATCTTGGTGCCTACTGCCTCGGA |
| **MCM3** | MCM3.958 | | TTTACTGAACTTCTTGATCTTG | | TGCTGTTGACAGTGAGCGAAAGATCAAGAAGTTCAGTAAATAGTGAAGCCACAGATGTATTTACTGAACTTCTTGATCTTGTGCCTACTGCCTCGGA |
| **MCM3** | MCM3.2977 | | TTTCTTGACCTGCATGACGTGC | | TGCTGTTGACAGTGAGCGACACGTCATGCAGGTCAAGAAATAGTGAAGCCACAGATGTATTTCTTGACCTGCATGACGTGCTGCCTACTGCCTCGGA |
| **MCM3** | MCM3.3080 | | TAAGTTTATTCAACATCTCGGA | | TGCTGTTGACAGTGAGCGCCCGAGATGTTGAATAAACTTATAGTGAAGCCACAGATGTATAAGTTTATTCAACATCTCGGATGCCTACTGCCTCGGA |
| **MCM4** | MCM4.216 | | TTATCGAGGAACAAACTTGGAA | | TGCTGTTGACAGTGAGCGCTCCAAGTTTGTTCCTCGATAATAGTGAAGCCACAGATGTATTATCGAGGAACAAACTTGGAATGCCTACTGCCTCGGA |
| **MCM4** | MCM4.3163 | | TTTTGAGACAGTCTCACTCTAT | | TGCTGTTGACAGTGAGCGCTAGAGTGAGACTGTCTCAAAATAGTGAAGCCACAGATGTATTTTGAGACAGTCTCACTCTATTGCCTACTGCCTCGGA |
| **MCM4** | MCM4.86 | | TAGGTTTCCATGTTGATTCGGG | | TGCTGTTGACAGTGAGCGACCGAATCAACATGGAAACCTATAGTGAAGCCACAGATGTATAGGTTTCCATGTTGATTCGGGTGCCTACTGCCTCGGA |
| **MCM4** | MCM4.1641 | | TATTCTTAGTCTTCAATGCGTT | | TGCTGTTGACAGTGAGCGCACGCATTGAAGACTAAGAATATAGTGAAGCCACAGATGTATATTCTTAGTCTTCAATGCGTTTGCCTACTGCCTCGGA |
| **MCM4** | MCM4.217 | | TTTATCGAGGAACAAACTTGGA | | TGCTGTTGACAGTGAGCGCCCAAGTTTGTTCCTCGATAAATAGTGAAGCCACAGATGTATTTATCGAGGAACAAACTTGGATGCCTACTGCCTCGGA |
| **MCM5** | MCM5.2528 | | TTTACCTGAACACACCGTGGCT | | TGCTGTTGACAGTGAGCGCGCCACGGTGTGTTCAGGTAAATAGTGAAGCCACAGATGTATTTACCTGAACACACCGTGGCTTGCCTACTGCCTCGGA |
| **MCM5** | MCM5.2529 | | TTTTACCTGAACACACCGTGGC | | TGCTGTTGACAGTGAGCGACCACGGTGTGTTCAGGTAAAATAGTGAAGCCACAGATGTATTTTACCTGAACACACCGTGGCTGCCTACTGCCTCGGA |
| **MCM5** | MCM5.1636 | | TTGACGATGAAGATCATGTCGA | | TGCTGTTGACAGTGAGCGCCGACATGATCTTCATCGTCAATAGTGAAGCCACAGATGTATTGACGATGAAGATCATGTCGATGCCTACTGCCTCGGA |
| **MCM5** | MCM5.240 | | TGTATTTGAAGGTGAAGCCCGT | | TGCTGTTGACAGTGAGCGCCGGGCTTCACCTTCAAATACATAGTGAAGCCACAGATGTATGTATTTGAAGGTGAAGCCCGTTGCCTACTGCCTCGGA |
| **MCM5** | MCM5.343 | | TTGTACAAGTAGTCGGCCAGGT | | TGCTGTTGACAGTGAGCGCCCTGGCCGACTACTTGTACAATAGTGAAGCCACAGATGTATTGTACAAGTAGTCGGCCAGGTTGCCTACTGCCTCGGA |
| **MCM6** | MCM6.1479 | | ATGAATAGCAACTTGATCCCGC | | TGCTGTTGACAGTGAGCGACGGGATCAAGTTGCTATTCATTAGTGAAGCCACAGATGTAATGAATAGCAACTTGATCCCGCTGCCTACTGCCTCGGA |
| **MCM6** | MCM6.2172 | | TTGATTTATGTCTTCATTGTAG | | TGCTGTTGACAGTGAGCGATACAATGAAGACATAAATCAATAGTGAAGCCACAGATGTATTGATTTATGTCTTCATTGTAGTGCCTACTGCCTCGGA |
| **MCM6** | MCM6.2798 | | TTTGGTTCCAACTTCACTGGGA | | TGCTGTTGACAGTGAGCGCCCCAGTGAAGTTGGAACCAAATAGTGAAGCCACAGATGTATTTGGTTCCAACTTCACTGGGATGCCTACTGCCTCGGA |
| **MCM6** | MCM6.2096 | | TTGATCTAGATTGACATCAGGT | | TGCTGTTGACAGTGAGCGCCCTGATGTCAATCTAGATCAATAGTGAAGCCACAGATGTATTGATCTAGATTGACATCAGGTTGCCTACTGCCTCGGA |
| **MCM6** | MCM6.2807 | | TTACACATGAAAACAAAGGTAT | | TGCTGTTGACAGTGAGCGCTACCTTTGTTTTCATGTGTAATAGTGAAGCCACAGATGTATTACACATGAAAACAAAGGTATTGCCTACTGCCTCGGA |
| **MCM7** | MCM7.1879 | | TTTGACATCTCCATTAGCCTGA | | TGCTGTTGACAGTGAGCGCCAGGCTAATGGAGATGTCAAATAGTGAAGCCACAGATGTATTTGACATCTCCATTAGCCTGATGCCTACTGCCTCGGA |
| **MCM7** | MCM7.897 | | TTGTTCATCTTCACAATCCGAT | | TGCTGTTGACAGTGAGCGCTCGGATTGTGAAGATGAACAATAGTGAAGCCACAGATGTATTGTTCATCTTCACAATCCGATTGCCTACTGCCTCGGA |
| **MCM7** | MCM7.8 | | TTGTAAGAACTTCTTAACCTTT | | TGCTGTTGACAGTGAGCGCAAGGTTAAGAAGTTCTTACAATAGTGAAGCCACAGATGTATTGTAAGAACTTCTTAACCTTTTGCCTACTGCCTCGGA |
| **MCM7** | MCM7.60 | | TACTTGAACTGCTTCTTCCCGA | | TGCTGTTGACAGTGAGCGCCGGGAAGAAGCAGTTCAAGTATAGTGAAGCCACAGATGTATACTTGAACTGCTTCTTCCCGATGCCTACTGCCTCGGA |
| **MCM7** | MCM7.674 | | TTCTTGCATCTTCATCTCCTGG | | TGCTGTTGACAGTGAGCGACAGGAGATGAAGATGCAAGAATAGTGAAGCCACAGATGTATTCTTGCATCTTCATCTCCTGGTGCCTACTGCCTCGGA |
| **MED8** | MED8.2 | | TTTTCCAGTCGAATTCCTCGAG | | TGCTGTTGACAGTGAGCGATCGAGGAATTCGACTGGAAAATAGTGAAGCCACAGATGTATTTTCCAGTCGAATTCCTCGAGTGCCTACTGCCTCGGA |
| **MED8** | MED8.561 | | TTAAAGGTCTGCTTGTTCGGCC | | TGCTGTTGACAGTGAGCGAGCCGAACAAGCAGACCTTTAATAGTGAAGCCACAGATGTATTAAAGGTCTGCTTGTTCGGCCTGCCTACTGCCTCGGA |
| **MED8** | MED8.222 | | TTGTTCAGAGTGTTCAGCTGTC | | TGCTGTTGACAGTGAGCGAACAGCTGAACACTCTGAACAATAGTGAAGCCACAGATGTATTGTTCAGAGTGTTCAGCTGTCTGCCTACTGCCTCGGA |
| **MED8** | MED8.405 | | TTCTCCTGTTCTTCCACTTCAG | | TGCTGTTGACAGTGAGCGATGAAGTGGAAGAACAGGAGAATAGTGAAGCCACAGATGTATTCTCCTGTTCTTCCACTTCAGTGCCTACTGCCTCGGA |
| **MED8** | MED8.474 | | TTATTCAAGCTCTGGATCTGCT | | TGCTGTTGACAGTGAGCGCGCAGATCCAGAGCTTGAATAATAGTGAAGCCACAGATGTATTATTCAAGCTCTGGATCTGCTTGCCTACTGCCTCGGA |
| **MYBL2** | MYBL2.2073 | | TTCTTTGATACCTGACAGGGTG | | TGCTGTTGACAGTGAGCGAACCCTGTCAGGTATCAAAGAATAGTGAAGCCACAGATGTATTCTTTGATACCTGACAGGGTGTGCCTACTGCCTCGGA |
| **MYBL2** | MYBL2.2234 | | TTCTCCTGCATGAAAAGCTGGT | | TGCTGTTGACAGTGAGCGCCCAGCTTTTCATGCAGGAGAATAGTGAAGCCACAGATGTATTCTCCTGCATGAAAAGCTGGTTGCCTACTGCCTCGGA |
| **MYBL2** | MYBL2.1874 | | TCGATGATGAGTTCGATGCCAG | | TGCTGTTGACAGTGAGCGATGGCATCGAACTCATCATCGATAGTGAAGCCACAGATGTATCGATGATGAGTTCGATGCCAGTGCCTACTGCCTCGGA |
| **MYBL2** | MYBL2.875 | | TTGGTCAGAAGACTTCCCTGGC | | TGCTGTTGACAGTGAGCGACCAGGGAAGTCTTCTGACCAATAGTGAAGCCACAGATGTATTGGTCAGAAGACTTCCCTGGCTGCCTACTGCCTCGGA |
| **MYBL2** | MYBL2.1535 | | TTAACAGGTGTGCTCTTGGGCG | | TGCTGTTGACAGTGAGCGAGCCCAAGAGCACACCTGTTAATAGTGAAGCCACAGATGTATTAACAGGTGTGCTCTTGGGCGTGCCTACTGCCTCGGA |
| **NAP1L4** | NAP1L4.362 | | TATGTGAGCACATCTCACCTGA | | TGCTGTTGACAGTGAGCGCCAGGTGAGATGTGCTCACATATAGTGAAGCCACAGATGTATATGTGAGCACATCTCACCTGATGCCTACTGCCTCGGA |
| **NAP1L4** | NAP1L4.2472 | | TAGAATATTCTAACTATTCTGT | | TGCTGTTGACAGTGAGCGCCAGAATAGTTAGAATATTCTATAGTGAAGCCACAGATGTATAGAATATTCTAACTATTCTGTTGCCTACTGCCTCGGA |
| **NAP1L4** | NAP1L4.740 | | TAACACAAAAGACATAGGCTGT | | TGCTGTTGACAGTGAGCGCCAGCCTATGTCTTTTGTGTTATAGTGAAGCCACAGATGTATAACACAAAAGACATAGGCTGTTGCCTACTGCCTCGGA |
| **NAP1L4** | NAP1L4.909 | | TTGACAGTAACATTCTTTCCTT | | TGCTGTTGACAGTGAGCGCAGGAAAGAATGTTACTGTCAATAGTGAAGCCACAGATGTATTGACAGTAACATTCTTTCCTTTGCCTACTGCCTCGGA |
| **NAP1L4** | NAP1L4.885 | | TTCCAGTCAATAGTACACCCGT | | TGCTGTTGACAGTGAGCGCCGGGTGTACTATTGACTGGAATAGTGAAGCCACAGATGTATTCCAGTCAATAGTACACCCGTTGCCTACTGCCTCGGA |
| **NF1** | NF1.898 | | TATATCATGAACATCAACATTG | | TGCTGTTGACAGTGAGCGAAATGTTGATGTTCATGATATATAGTGAAGCCACAGATGTATATATCATGAACATCAACATTGTGCCTACTGCCTCGGA |
| **NF1** | NF1.898 | | TATATCATGAACATCAACATTG | | TGCTGTTGACAGTGAGCGAAATGTTGATGTTCATGATATATAGTGAAGCCACAGATGTATATATCATGAACATCAACATTGTGCCTACTGCCTCGGA |
| **NF1** | NF1.1588 | | TAGAAGGTGAATTCTGAGCCAG | | TGCTGTTGACAGTGAGCGATGGCTCAGAATTCACCTTCTATAGTGAAGCCACAGATGTATAGAAGGTGAATTCTGAGCCAGTGCCTACTGCCTCGGA |
| **NF1** | NF1.1588 | | TAGAAGGTGAATTCTGAGCCAG | | TGCTGTTGACAGTGAGCGATGGCTCAGAATTCACCTTCTATAGTGAAGCCACAGATGTATAGAAGGTGAATTCTGAGCCAGTGCCTACTGCCTCGGA |
| **NF1** | NF1.561 | | TAAAATAGTAGTGAGGCCGCTT | | TGCTGTTGACAGTGAGCGCAGCGGCCTCACTACTATTTTATAGTGAAGCCACAGATGTATAAAATAGTAGTGAGGCCGCTTTGCCTACTGCCTCGGA |
| **NF1** | NF1.561 | | TAAAATAGTAGTGAGGCCGCTT | | TGCTGTTGACAGTGAGCGCAGCGGCCTCACTACTATTTTATAGTGAAGCCACAGATGTATAAAATAGTAGTGAGGCCGCTTTGCCTACTGCCTCGGA |
| **NF1** | NF1.1785 | | TTCTTTAAATGTAAGACTCGGT | | TGCTGTTGACAGTGAGCGCCCGAGTCTTACATTTAAAGAATAGTGAAGCCACAGATGTATTCTTTAAATGTAAGACTCGGTTGCCTACTGCCTCGGA |
| **NF1** | NF1.1785 | | TTCTTTAAATGTAAGACTCGGT | | TGCTGTTGACAGTGAGCGCCCGAGTCTTACATTTAAAGAATAGTGAAGCCACAGATGTATTCTTTAAATGTAAGACTCGGTTGCCTACTGCCTCGGA |
| **NF1** | NF1.1361 | | TTACACAGTTTGACACAGGCAA | | TGCTGTTGACAGTGAGCGCTGCCTGTGTCAAACTGTGTAATAGTGAAGCCACAGATGTATTACACAGTTTGACACAGGCAATGCCTACTGCCTCGGA |
| **NF1** | NF1.1361 | | TTACACAGTTTGACACAGGCAA | | TGCTGTTGACAGTGAGCGCTGCCTGTGTCAAACTGTGTAATAGTGAAGCCACAGATGTATTACACAGTTTGACACAGGCAATGCCTACTGCCTCGGA |
| **NFE2L3** | NFE2L3.943 | | TTCATCATCATCATTCTGCTGA | | TGCTGTTGACAGTGAGCGCCAGCAGAATGATGATGATGAATAGTGAAGCCACAGATGTATTCATCATCATCATTCTGCTGATGCCTACTGCCTCGGA |
| **NFE2L3** | NFE2L3.1541 | | TATTATTGTGACTTGAATCTAA | | TGCTGTTGACAGTGAGCGCTAGATTCAAGTCACAATAATATAGTGAAGCCACAGATGTATATTATTGTGACTTGAATCTAATGCCTACTGCCTCGGA |
| **NFE2L3** | NFE2L3.3599 | | TACTCTACACTGTAGCTCCTAT | | TGCTGTTGACAGTGAGCGCTAGGAGCTACAGTGTAGAGTATAGTGAAGCCACAGATGTATACTCTACACTGTAGCTCCTATTGCCTACTGCCTCGGA |
| **NFE2L3** | NFE2L3.350 | | TAAAGATCTAGGTCTACGCGGA | | TGCTGTTGACAGTGAGCGCCCGCGTAGACCTAGATCTTTATAGTGAAGCCACAGATGTATAAAGATCTAGGTCTACGCGGATGCCTACTGCCTCGGA |
| **NFE2L3** | NFE2L3.1962 | | TAATATCTACTTAACATGCTAT | | TGCTGTTGACAGTGAGCGCTAGCATGTTAAGTAGATATTATAGTGAAGCCACAGATGTATAATATCTACTTAACATGCTATTGCCTACTGCCTCGGA |
| **NFKB1** | NFKB1.1378 | | TTGTCTATGAACATCTGTGGGG | | TGCTGTTGACAGTGAGCGACCCACAGATGTTCATAGACAATAGTGAAGCCACAGATGTATTGTCTATGAACATCTGTGGGGTGCCTACTGCCTCGGA |
| **NFKB1** | NFKB1.2926 | | TTCTAGTAACTTATACAGCTGC | | TGCTGTTGACAGTGAGCGACAGCTGTATAAGTTACTAGAATAGTGAAGCCACAGATGTATTCTAGTAACTTATACAGCTGCTGCCTACTGCCTCGGA |
| **NFKB1** | NFKB1.3703 | | TTTAATGACAATAGGAACGTAG | | TGCTGTTGACAGTGAGCGATACGTTCCTATTGTCATTAAATAGTGAAGCCACAGATGTATTTAATGACAATAGGAACGTAGTGCCTACTGCCTCGGA |
| **NFKB1** | NFKB1.2920 | | TAACTTATACAGCTGCAGCTTC | | TGCTGTTGACAGTGAGCGAAAGCTGCAGCTGTATAAGTTATAGTGAAGCCACAGATGTATAACTTATACAGCTGCAGCTTCTGCCTACTGCCTCGGA |
| **NFKB1** | NFKB1.2367 | | TTGTGCTTGAGTAAGATACTGA | | TGCTGTTGACAGTGAGCGCCAGTATCTTACTCAAGCACAATAGTGAAGCCACAGATGTATTGTGCTTGAGTAAGATACTGATGCCTACTGCCTCGGA |
| **PA2G4** | PA2G4.706 | | TTCCTTGAGAATATAATCCTGG | | TGCTGTTGACAGTGAGCGACAGGATTATATTCTCAAGGAATAGTGAAGCCACAGATGTATTCCTTGAGAATATAATCCTGGTGCCTACTGCCTCGGA |
| **PA2G4** | PA2G4.994 | | TTCTCCATCGATGACATGCTGC | | TGCTGTTGACAGTGAGCGACAGCATGTCATCGATGGAGAATAGTGAAGCCACAGATGTATTCTCCATCGATGACATGCTGCTGCCTACTGCCTCGGA |
| **PA2G4** | PA2G4.1492 | | TTCGACTTGCAGAACTCTGGAG | | TGCTGTTGACAGTGAGCGATCCAGAGTTCTGCAAGTCGAATAGTGAAGCCACAGATGTATTCGACTTGCAGAACTCTGGAGTGCCTACTGCCTCGGA |
| **PA2G4** | PA2G4.1812 | | TCATTTAAAGTAGTCTTCCGTG | | TGCTGTTGACAGTGAGCGAACGGAAGACTACTTTAAATGATAGTGAAGCCACAGATGTATCATTTAAAGTAGTCTTCCGTGTGCCTACTGCCTCGGA |
| **PA2G4** | PA2G4.1809 | | TTTAAAGTAGTCTTCCGTGGTT | | TGCTGTTGACAGTGAGCGCACCACGGAAGACTACTTTAAATAGTGAAGCCACAGATGTATTTAAAGTAGTCTTCCGTGGTTTGCCTACTGCCTCGGA |
| **PHTF2** | PHTF2.323 | | TTACTTGTAACCACCACCGGAA | | TGCTGTTGACAGTGAGCGCTCCGGTGGTGGTTACAAGTAATAGTGAAGCCACAGATGTATTACTTGTAACCACCACCGGAATGCCTACTGCCTCGGA |
| **PHTF2** | PHTF2.269 | | TACAACTCGAACAATTCCCTTT | | TGCTGTTGACAGTGAGCGCAAGGGAATTGTTCGAGTTGTATAGTGAAGCCACAGATGTATACAACTCGAACAATTCCCTTTTGCCTACTGCCTCGGA |
| **PHTF2** | PHTF2.808 | | TTGTATTCCATCTTCACCGCTT | | TGCTGTTGACAGTGAGCGCAGCGGTGAAGATGGAATACAATAGTGAAGCCACAGATGTATTGTATTCCATCTTCACCGCTTTGCCTACTGCCTCGGA |
| **PHTF2** | PHTF2.993 | | TTCTATTCCGAAGAACACCTTC | | TGCTGTTGACAGTGAGCGAAAGGTGTTCTTCGGAATAGAATAGTGAAGCCACAGATGTATTCTATTCCGAAGAACACCTTCTGCCTACTGCCTCGGA |
| **PHTF2** | PHTF2.714 | | TATCAATTGAATTCTTTGCTTT | | TGCTGTTGACAGTGAGCGCAAGCAAAGAATTCAATTGATATAGTGAAGCCACAGATGTATATCAATTGAATTCTTTGCTTTTGCCTACTGCCTCGGA |
| **PMEPA1** | PMEPA1.4210 | | TTAACTTGAACAGAGCTTGGGA | | TGCTGTTGACAGTGAGCGCCCCAAGCTCTGTTCAAGTTAATAGTGAAGCCACAGATGTATTAACTTGAACAGAGCTTGGGATGCCTACTGCCTCGGA |
| **PMEPA1** | PMEPA1.3541 | | TAACGTGACAACTACCATCTAG | | TGCTGTTGACAGTGAGCGATAGATGGTAGTTGTCACGTTATAGTGAAGCCACAGATGTATAACGTGACAACTACCATCTAGTGCCTACTGCCTCGGA |
| **PMEPA1** | PMEPA1.4018 | | TTTTCTTTCCTTATCGACGGGA | | TGCTGTTGACAGTGAGCGCCCCGTCGATAAGGAAAGAAAATAGTGAAGCCACAGATGTATTTTCTTTCCTTATCGACGGGATGCCTACTGCCTCGGA |
| **PMEPA1** | PMEPA1.2011 | | TTAAGTGAGAATTGATCCGTGA | | TGCTGTTGACAGTGAGCGCCACGGATCAATTCTCACTTAATAGTGAAGCCACAGATGTATTAAGTGAGAATTGATCCGTGATGCCTACTGCCTCGGA |
| **PMEPA1** | PMEPA1.2873 | | TTAATCATCTTTACAAGTGCGT | | TGCTGTTGACAGTGAGCGCCGCACTTGTAAAGATGATTAATAGTGAAGCCACAGATGTATTAATCATCTTTACAAGTGCGTTGCCTACTGCCTCGGA |
| **PML** | PML.1271 | | TTGAACTCGTCGAAGCCATCGG | | TGCTGTTGACAGTGAGCGACGATGGCTTCGACGAGTTCAATAGTGAAGCCACAGATGTATTGAACTCGTCGAAGCCATCGGTGCCTACTGCCTCGGA |
| **PML** | PML.105 | | TTTAGATCTTGGAGTGCGTGAA | | TGCTGTTGACAGTGAGCGCTCACGCACTCCAAGATCTAAATAGTGAAGCCACAGATGTATTTAGATCTTGGAGTGCGTGAATGCCTACTGCCTCGGA |
| **PML** | PML.109 | | TCGGTTTAGATCTTGGAGTGCG | | TGCTGTTGACAGTGAGCGAGCACTCCAAGATCTAAACCGATAGTGAAGCCACAGATGTATCGGTTTAGATCTTGGAGTGCGTGCCTACTGCCTCGGA |
| **PML** | PML.1148 | | TAGCACTTCATCCTCTGCACCA | | TGCTGTTGACAGTGAGCGCGGTGCAGAGGATGAAGTGCTATAGTGAAGCCACAGATGTATAGCACTTCATCCTCTGCACCATGCCTACTGCCTCGGA |
| **PML** | PML.111 | | TCTCGGTTTAGATCTTGGAGTG | | TGCTGTTGACAGTGAGCGAACTCCAAGATCTAAACCGAGATAGTGAAGCCACAGATGTATCTCGGTTTAGATCTTGGAGTGTGCCTACTGCCTCGGA |
| **PNN** | PNN.401 | | TTTAACATCATCATCCTCCGGG | | TGCTGTTGACAGTGAGCGACCGGAGGATGATGATGTTAAATAGTGAAGCCACAGATGTATTTAACATCATCATCCTCCGGGTGCCTACTGCCTCGGA |
| **PNN** | PNN.1071 | | TATTTCTACATCATTGTGCTGA | | TGCTGTTGACAGTGAGCGCCAGCACAATGATGTAGAAATATAGTGAAGCCACAGATGTATATTTCTACATCATTGTGCTGATGCCTACTGCCTCGGA |
| **PNN** | PNN.2904 | | TACTGTTTAGTTATCTACCGAA | | TGCTGTTGACAGTGAGCGCTCGGTAGATAACTAAACAGTATAGTGAAGCCACAGATGTATACTGTTTAGTTATCTACCGAATGCCTACTGCCTCGGA |
| **PNN** | PNN.2847 | | TTAGATTAAAATAACAAGGGAG | | TGCTGTTGACAGTGAGCGATCCCTTGTTATTTTAATCTAATAGTGAAGCCACAGATGTATTAGATTAAAATAACAAGGGAGTGCCTACTGCCTCGGA |
| **PNN** | PNN.400 | | TTAACATCATCATCCTCCGGGT | | TGCTGTTGACAGTGAGCGCCCCGGAGGATGATGATGTTAATAGTGAAGCCACAGATGTATTAACATCATCATCCTCCGGGTTGCCTACTGCCTCGGA |
| **POLR1E** | POLR1E.1199 | | TTGGCTATCTCCATCATCCTTT | | TGCTGTTGACAGTGAGCGCAAGGATGATGGAGATAGCCAATAGTGAAGCCACAGATGTATTGGCTATCTCCATCATCCTTTTGCCTACTGCCTCGGA |
| **POLR1E** | POLR1E.501 | | TTCAATACAAGAATCCATCTTT | | TGCTGTTGACAGTGAGCGCAAGATGGATTCTTGTATTGAATAGTGAAGCCACAGATGTATTCAATACAAGAATCCATCTTTTGCCTACTGCCTCGGA |
| **POLR1E** | POLR1E.815 | | TTCAGTATTTCTTCTGACGTGA | | TGCTGTTGACAGTGAGCGCCACGTCAGAAGAAATACTGAATAGTGAAGCCACAGATGTATTCAGTATTTCTTCTGACGTGATGCCTACTGCCTCGGA |
| **POLR1E** | POLR1E.1264 | | TTGTGATCTTCTTCACTGCCGG | | TGCTGTTGACAGTGAGCGACGGCAGTGAAGAAGATCACAATAGTGAAGCCACAGATGTATTGTGATCTTCTTCACTGCCGGTGCCTACTGCCTCGGA |
| **POLR1E** | POLR1E.467 | | TTTGGTCTGACTCTCTAGCGCC | | TGCTGTTGACAGTGAGCGAGCGCTAGAGAGTCAGACCAAATAGTGAAGCCACAGATGTATTTGGTCTGACTCTCTAGCGCCTGCCTACTGCCTCGGA |
| **POLR2F** | POLR2F.1308 | | TCTGACTTAATGTAACACCTGG | | TGCTGTTGACAGTGAGCGACAGGTGTTACATTAAGTCAGATAGTGAAGCCACAGATGTATCTGACTTAATGTAACACCTGGTGCCTACTGCCTCGGA |
| **POLR2F** | POLR2F.1300 | | TAATGTAACACCTGGAACCCAG | | TGCTGTTGACAGTGAGCGATGGGTTCCAGGTGTTACATTATAGTGAAGCCACAGATGTATAATGTAACACCTGGAACCCAGTGCCTACTGCCTCGGA |
| **POLR2F** | POLR2F.112 | | TTGTCTGACATGACACCCTCGC | | TGCTGTTGACAGTGAGCGACGAGGGTGTCATGTCAGACAATAGTGAAGCCACAGATGTATTGTCTGACATGACACCCTCGCTGCCTACTGCCTCGGA |
| **POLR2F** | POLR2F.181 | | TTCTCCAAGTCATCTAGCCCTT | | TGCTGTTGACAGTGAGCGCAGGGCTAGATGACTTGGAGAATAGTGAAGCCACAGATGTATTCTCCAAGTCATCTAGCCCTTTGCCTACTGCCTCGGA |
| **POLR2F** | POLR2F.1305 | | TGACTTAATGTAACACCTGGAA | | TGCTGTTGACAGTGAGCGCTCCAGGTGTTACATTAAGTCATAGTGAAGCCACAGATGTATGACTTAATGTAACACCTGGAATGCCTACTGCCTCGGA |
| **POLR3K** | POLR3K.166 | | TTCAGTTTTGGGTACTTCCGAT | | TGCTGTTGACAGTGAGCGCTCGGAAGTACCCAAAACTGAATAGTGAAGCCACAGATGTATTCAGTTTTGGGTACTTCCGATTGCCTACTGCCTCGGA |
| **POLR3K** | POLR3K.726 | | TAACAGTTAAATATTTAGGGAC | | TGCTGTTGACAGTGAGCGATCCCTAAATATTTAACTGTTATAGTGAAGCCACAGATGTATAACAGTTAAATATTTAGGGACTGCCTACTGCCTCGGA |
| **POLR3K** | POLR3K.313 | | TTGTAGAAGGTGGTCATCGGCT | | TGCTGTTGACAGTGAGCGCGCCGATGACCACCTTCTACAATAGTGAAGCCACAGATGTATTGTAGAAGGTGGTCATCGGCTTGCCTACTGCCTCGGA |
| **POLR3K** | POLR3K.676 | | TATTTAAGAGTGTTGATTGGGA | | TGCTGTTGACAGTGAGCGCCCCAATCAACACTCTTAAATATAGTGAAGCCACAGATGTATATTTAAGAGTGTTGATTGGGATGCCTACTGCCTCGGA |
| **POLR3K** | POLR3K.151 | | TTCCGATTTGTTACCTTGCGGG | | TGCTGTTGACAGTGAGCGACCGCAAGGTAACAAATCGGAATAGTGAAGCCACAGATGTATTCCGATTTGTTACCTTGCGGGTGCCTACTGCCTCGGA |
| **PPP1R15A** | PPP1R15A.49 | | TTGCATAAGATCAACAACTGGG | | TGCTGTTGACAGTGAGCGACCAGTTGTTGATCTTATGCAATAGTGAAGCCACAGATGTATTGCATAAGATCAACAACTGGGTGCCTACTGCCTCGGA |
| **PPP1R15A** | PPP1R15A.963 | | TTCTGTTCTTTTATCCTCCGTG | | TGCTGTTGACAGTGAGCGAACGGAGGATAAAAGAACAGAATAGTGAAGCCACAGATGTATTCTGTTCTTTTATCCTCCGTGTGCCTACTGCCTCGGA |
| **PPP1R15A** | PPP1R15A.1494 | | TTCTCTTTCATCCTCGGCTGAT | | TGCTGTTGACAGTGAGCGCTCAGCCGAGGATGAAAGAGAATAGTGAAGCCACAGATGTATTCTCTTTCATCCTCGGCTGATTGCCTACTGCCTCGGA |
| **PPP1R15A** | PPP1R15A.2344 | | TTTATTCCTTATATAAACCCAC | | TGCTGTTGACAGTGAGCGATGGGTTTATATAAGGAATAAATAGTGAAGCCACAGATGTATTTATTCCTTATATAAACCCACTGCCTACTGCCTCGGA |
| **PPP1R15A** | PPP1R15A.964 | | TTTCTGTTCTTTTATCCTCCGT | | TGCTGTTGACAGTGAGCGCCGGAGGATAAAAGAACAGAAATAGTGAAGCCACAGATGTATTTCTGTTCTTTTATCCTCCGTTGCCTACTGCCTCGGA |
| **PPP2R5A** | PPP2R5A.694 | | TACAACACAATATGCTAGCTGA | | TGCTGTTGACAGTGAGCGCCAGCTAGCATATTGTGTTGTATAGTGAAGCCACAGATGTATACAACACAATATGCTAGCTGATGCCTACTGCCTCGGA |
| **PPP2R5A** | PPP2R5A.49 | | AAACAGTATACAACACTGCTGC | | TGCTGTTGACAGTGAGCGACAGCAGTGTTGTATACTGTTTTAGTGAAGCCACAGATGTAAAACAGTATACAACACTGCTGCTGCCTACTGCCTCGGA |
| **PPP2R5A** | PPP2R5A.730 | | TTAGTGTTGTATCTTTCTCCAG | | TGCTGTTGACAGTGAGCGATGGAGAAAGATACAACACTAATAGTGAAGCCACAGATGTATTAGTGTTGTATCTTTCTCCAGTGCCTACTGCCTCGGA |
| **PPP2R5A** | PPP2R5A.96 | | TTCTTTGCTCTTCAAGTCTGAA | | TGCTGTTGACAGTGAGCGCTCAGACTTGAAGAGCAAAGAATAGTGAAGCCACAGATGTATTCTTTGCTCTTCAAGTCTGAATGCCTACTGCCTCGGA |
| **PPP2R5A** | PPP2R5A.183 | | TACTATATCAGAATACGCTGAT | | TGCTGTTGACAGTGAGCGCTCAGCGTATTCTGATATAGTATAGTGAAGCCACAGATGTATACTATATCAGAATACGCTGATTGCCTACTGCCTCGGA |
| **PPP2R5B** | PPP2R5B.1181 | | TTTTGATCCACATATCTCTTGG | | TGCTGTTGACAGTGAGCGACAAGAGATATGTGGATCAAAATAGTGAAGCCACAGATGTATTTTGATCCACATATCTCTTGGTGCCTACTGCCTCGGA |
| **PPP2R5B** | PPP2R5B.2733 | | TTTTCTTGAATTTCTCTCTTTA | | TGCTGTTGACAGTGAGCGCAAAGAGAGAAATTCAAGAAAATAGTGAAGCCACAGATGTATTTTCTTGAATTTCTCTCTTTATGCCTACTGCCTCGGA |
| **PPP2R5B** | PPP2R5B.1342 | | TCGAATTCATAGATGAACCGGA | | TGCTGTTGACAGTGAGCGCCCGGTTCATCTATGAATTCGATAGTGAAGCCACAGATGTATCGAATTCATAGATGAACCGGATGCCTACTGCCTCGGA |
| **PPP2R5B** | PPP2R5B.1178 | | TTGATCCACATATCTCTTGGCC | | TGCTGTTGACAGTGAGCGAGCCAAGAGATATGTGGATCAATAGTGAAGCCACAGATGTATTGATCCACATATCTCTTGGCCTGCCTACTGCCTCGGA |
| **PPP2R5B** | PPP2R5B.1879 | | TTGAGCACATTGTAGATCAGTG | | TGCTGTTGACAGTGAGCGAACTGATCTACAATGTGCTCAATAGTGAAGCCACAGATGTATTGAGCACATTGTAGATCAGTGTGCCTACTGCCTCGGA |
| **PPP2R5C** | PPP2R5C.1208 | | TAGATTTTCTATTTTAACCCAT | | TGCTGTTGACAGTGAGCGCTGGGTTAAAATAGAAAATCTATAGTGAAGCCACAGATGTATAGATTTTCTATTTTAACCCATTGCCTACTGCCTCGGA |
| **PPP2R5C** | PPP2R5C.118 | | TTTCACTTAAAGCAGCTCGTTT | | TGCTGTTGACAGTGAGCGCAACGAGCTGCTTTAAGTGAAATAGTGAAGCCACAGATGTATTTCACTTAAAGCAGCTCGTTTTGCCTACTGCCTCGGA |
| **PPP2R5C** | PPP2R5C.1159 | | TTCTCTTTTAGTTTCTCTGCTT | | TGCTGTTGACAGTGAGCGCAGCAGAGAAACTAAAAGAGAATAGTGAAGCCACAGATGTATTCTCTTTTAGTTTCTCTGCTTTGCCTACTGCCTCGGA |
| **PPP2R5C** | PPP2R5C.631 | | TTGTGCTCTTCTTTTAGTGGTA | | TGCTGTTGACAGTGAGCGCACCACTAAAAGAAGAGCACAATAGTGAAGCCACAGATGTATTGTGCTCTTCTTTTAGTGGTATGCCTACTGCCTCGGA |
| **PPP2R5C** | PPP2R5C.362 | | TATATTAGGTTGGAAATCTGGA | | TGCTGTTGACAGTGAGCGCCCAGATTTCCAACCTAATATATAGTGAAGCCACAGATGTATATATTAGGTTGGAAATCTGGATGCCTACTGCCTCGGA |
| **PPP2R5E** | PPP2R5E.679 | | TAAACTGTGAGGAACTTTGCGA | | TGCTGTTGACAGTGAGCGCCGCAAAGTTCCTCACAGTTTATAGTGAAGCCACAGATGTATAAACTGTGAGGAACTTTGCGATGCCTACTGCCTCGGA |
| **PPP2R5E** | PPP2R5E.2683 | | TAAACTGATGCATGAATCCGAT | | TGCTGTTGACAGTGAGCGCTCGGATTCATGCATCAGTTTATAGTGAAGCCACAGATGTATAAACTGATGCATGAATCCGATTGCCTACTGCCTCGGA |
| **PPP2R5E** | PPP2R5E.1240 | | TATTGTTAATCTGTTTTCGGAT | | TGCTGTTGACAGTGAGCGCTCCGAAAACAGATTAACAATATAGTGAAGCCACAGATGTATATTGTTAATCTGTTTTCGGATTGCCTACTGCCTCGGA |
| **PPP2R5E** | PPP2R5E.1269 | | TTCTGTTTCATAAACAAACCTT | | TGCTGTTGACAGTGAGCGCAGGTTTGTTTATGAAACAGAATAGTGAAGCCACAGATGTATTCTGTTTCATAAACAAACCTTTGCCTACTGCCTCGGA |
| **PPP2R5E** | PPP2R5E.2031 | | TTCTACTACATAAACGTGTGAC | | TGCTGTTGACAGTGAGCGATCACACGTTTATGTAGTAGAATAGTGAAGCCACAGATGTATTCTACTACATAAACGTGTGACTGCCTACTGCCTCGGA |
| **PTTG1** | PTTG1.670 | | TACAAATACACACAAACTCTGA | | TGCTGTTGACAGTGAGCGCCAGAGTTTGTGTGTATTTGTATAGTGAAGCCACAGATGTATACAAATACACACAAACTCTGATGCCTACTGCCTCGGA |
| **PTTG1** | PTTG1.327 | | TTTGCTTTAACAGTCTTCTCAG | | TGCTGTTGACAGTGAGCGATGAGAAGACTGTTAAAGCAAATAGTGAAGCCACAGATGTATTTGCTTTAACAGTCTTCTCAGTGCCTACTGCCTCGGA |
| **PTTG1** | PTTG1.18 | | TATTGCAGGTCTTAACAGCCGC | | TGCTGTTGACAGTGAGCGACGGCTGTTAAGACCTGCAATATAGTGAAGCCACAGATGTATATTGCAGGTCTTAACAGCCGCTGCCTACTGCCTCGGA |
| **PTTG1** | PTTG1.236 | | TAGCTCTGTTGACAGTTCCCAA | | TGCTGTTGACAGTGAGCGCTGGGAACTGTCAACAGAGCTATAGTGAAGCCACAGATGTATAGCTCTGTTGACAGTTCCCAATGCCTACTGCCTCGGA |
| **PTTG1** | PTTG1.7 | | TTAACAGCCGCATTCATCTGAG | | TGCTGTTGACAGTGAGCGATCAGATGAATGCGGCTGTTAATAGTGAAGCCACAGATGTATTAACAGCCGCATTCATCTGAGTGCCTACTGCCTCGGA |
| **RASA1** | RASA1.1954 | | TTATCTTTAAAATAGAGTCTTT | | TGCTGTTGACAGTGAGCGCAAGACTCTATTTTAAAGATAATAGTGAAGCCACAGATGTATTATCTTTAAAATAGAGTCTTTTGCCTACTGCCTCGGA |
| **RASA1** | RASA1.1954 | | TTATCTTTAAAATAGAGTCTTT | | TGCTGTTGACAGTGAGCGCAAGACTCTATTTTAAAGATAATAGTGAAGCCACAGATGTATTATCTTTAAAATAGAGTCTTTTGCCTACTGCCTCGGA |
| **RASA1** | RASA1.396 | | TAGAATAGCTCGTACACGCCTT | | TGCTGTTGACAGTGAGCGCAGGCGTGTACGAGCTATTCTATAGTGAAGCCACAGATGTATAGAATAGCTCGTACACGCCTTTGCCTACTGCCTCGGA |
| **RASA1** | RASA1.396 | | TAGAATAGCTCGTACACGCCTT | | TGCTGTTGACAGTGAGCGCAGGCGTGTACGAGCTATTCTATAGTGAAGCCACAGATGTATAGAATAGCTCGTACACGCCTTTGCCTACTGCCTCGGA |
| **RASA1** | RASA1.573 | | TTCATGTGGATCTTCTTCCCGG | | TGCTGTTGACAGTGAGCGACGGGAAGAAGATCCACATGAATAGTGAAGCCACAGATGTATTCATGTGGATCTTCTTCCCGGTGCCTACTGCCTCGGA |
| **RASA1** | RASA1.573 | | TTCATGTGGATCTTCTTCCCGG | | TGCTGTTGACAGTGAGCGACGGGAAGAAGATCCACATGAATAGTGAAGCCACAGATGTATTCATGTGGATCTTCTTCCCGGTGCCTACTGCCTCGGA |
| **RASA1** | RASA1.2153 | | TAAATATATCTCAATGTCGGTG | | TGCTGTTGACAGTGAGCGAACCGACATTGAGATATATTTATAGTGAAGCCACAGATGTATAAATATATCTCAATGTCGGTGTGCCTACTGCCTCGGA |
| **RASA1** | RASA1.2153 | | TAAATATATCTCAATGTCGGTG | | TGCTGTTGACAGTGAGCGAACCGACATTGAGATATATTTATAGTGAAGCCACAGATGTATAAATATATCTCAATGTCGGTGTGCCTACTGCCTCGGA |
| **RASA1** | RASA1.3593 | | TAAAACTATACAGTTGAACTAA | | TGCTGTTGACAGTGAGCGCTAGTTCAACTGTATAGTTTTATAGTGAAGCCACAGATGTATAAAACTATACAGTTGAACTAATGCCTACTGCCTCGGA |
| **RASA1** | RASA1.3593 | | TAAAACTATACAGTTGAACTAA | | TGCTGTTGACAGTGAGCGCTAGTTCAACTGTATAGTTTTATAGTGAAGCCACAGATGTATAAAACTATACAGTTGAACTAATGCCTACTGCCTCGGA |
| **RASA2** | RASA2.1380 | | TTGTATTGAATAACTTGTCTAC | | TGCTGTTGACAGTGAGCGATAGACAAGTTATTCAATACAATAGTGAAGCCACAGATGTATTGTATTGAATAACTTGTCTACTGCCTACTGCCTCGGA |
| **RASA2** | RASA2.1380 | | TTGTATTGAATAACTTGTCTAC | | TGCTGTTGACAGTGAGCGATAGACAAGTTATTCAATACAATAGTGAAGCCACAGATGTATTGTATTGAATAACTTGTCTACTGCCTACTGCCTCGGA |
| **RASA2** | RASA2.1884 | | TTGTTAAGCAGAACCATCGTTT | | TGCTGTTGACAGTGAGCGCAACGATGGTTCTGCTTAACAATAGTGAAGCCACAGATGTATTGTTAAGCAGAACCATCGTTTTGCCTACTGCCTCGGA |
| **RASA2** | RASA2.1884 | | TTGTTAAGCAGAACCATCGTTT | | TGCTGTTGACAGTGAGCGCAACGATGGTTCTGCTTAACAATAGTGAAGCCACAGATGTATTGTTAAGCAGAACCATCGTTTTGCCTACTGCCTCGGA |
| **RASA2** | RASA2.760 | | TTGTTCCACAAGTCGATCCTGA | | TGCTGTTGACAGTGAGCGCCAGGATCGACTTGTGGAACAATAGTGAAGCCACAGATGTATTGTTCCACAAGTCGATCCTGATGCCTACTGCCTCGGA |
| **RASA2** | RASA2.760 | | TTGTTCCACAAGTCGATCCTGA | | TGCTGTTGACAGTGAGCGCCAGGATCGACTTGTGGAACAATAGTGAAGCCACAGATGTATTGTTCCACAAGTCGATCCTGATGCCTACTGCCTCGGA |
| **RASA2** | RASA2.449 | | TTCAAGGTGAACTTTACCCTGA | | TGCTGTTGACAGTGAGCGCCAGGGTAAAGTTCACCTTGAATAGTGAAGCCACAGATGTATTCAAGGTGAACTTTACCCTGATGCCTACTGCCTCGGA |
| **RASA2** | RASA2.449 | | TTCAAGGTGAACTTTACCCTGA | | TGCTGTTGACAGTGAGCGCCAGGGTAAAGTTCACCTTGAATAGTGAAGCCACAGATGTATTCAAGGTGAACTTTACCCTGATGCCTACTGCCTCGGA |
| **RASA2** | RASA2.2057 | | TTCTACACAGTTATTTGCCTGG | | TGCTGTTGACAGTGAGCGACAGGCAAATAACTGTGTAGAATAGTGAAGCCACAGATGTATTCTACACAGTTATTTGCCTGGTGCCTACTGCCTCGGA |
| **RASA2** | RASA2.2057 | | TTCTACACAGTTATTTGCCTGG | | TGCTGTTGACAGTGAGCGACAGGCAAATAACTGTGTAGAATAGTGAAGCCACAGATGTATTCTACACAGTTATTTGCCTGGTGCCTACTGCCTCGGA |
| **RASA3** | RASA3.2532 | | TATTTCGTCTTCTTGAACTTGT | | TGCTGTTGACAGTGAGCGCCAAGTTCAAGAAGACGAAATATAGTGAAGCCACAGATGTATATTTCGTCTTCTTGAACTTGTTGCCTACTGCCTCGGA |
| **RASA3** | RASA3.3525 | | TTACTTTGTATCAAAGAGCTAA | | TGCTGTTGACAGTGAGCGCTAGCTCTTTGATACAAAGTAATAGTGAAGCCACAGATGTATTACTTTGTATCAAAGAGCTAATGCCTACTGCCTCGGA |
| **RASA3** | RASA3.276 | | TTTTCCACAATTTTGGTCCTGA | | TGCTGTTGACAGTGAGCGCCAGGACCAAAATTGTGGAAAATAGTGAAGCCACAGATGTATTTTCCACAATTTTGGTCCTGATGCCTACTGCCTCGGA |
| **RASA3** | RASA3.4009 | | TTTGTGACAAGAAAGTTCCTAT | | TGCTGTTGACAGTGAGCGCTAGGAACTTTCTTGTCACAAATAGTGAAGCCACAGATGTATTTGTGACAAGAAAGTTCCTATTGCCTACTGCCTCGGA |
| **RASA3** | RASA3.707 | | TAAAACACTTCATCGAACTGGG | | TGCTGTTGACAGTGAGCGACCAGTTCGATGAAGTGTTTTATAGTGAAGCCACAGATGTATAAAACACTTCATCGAACTGGGTGCCTACTGCCTCGGA |
| **RASA4** | RASA4.4988 | | TTAATAATTCCAATCATCCTAT | | TGCTGTTGACAGTGAGCGCTAGGATGATTGGAATTATTAATAGTGAAGCCACAGATGTATTAATAATTCCAATCATCCTATTGCCTACTGCCTCGGA |
| **RASA4** | RASA4.4126 | | TTTCGTGATCTCTTCTTCCCTT | | TGCTGTTGACAGTGAGCGCAGGGAAGAAGAGATCACGAAATAGTGAAGCCACAGATGTATTTCGTGATCTCTTCTTCCCTTTGCCTACTGCCTCGGA |
| **RASA4** | RASA4.4422 | | TAAAAATACAAGAATTAGCTGG | | TGCTGTTGACAGTGAGCGACAGCTAATTCTTGTATTTTTATAGTGAAGCCACAGATGTATAAAAATACAAGAATTAGCTGGTGCCTACTGCCTCGGA |
| **RASA4** | RASA4.3049 | | TTTGAGGAGAGAACAGCTGGTT | | TGCTGTTGACAGTGAGCGCACCAGCTGTTCTCTCCTCAAATAGTGAAGCCACAGATGTATTTGAGGAGAGAACAGCTGGTTTGCCTACTGCCTCGGA |
| **RASA4** | RASA4.3459 | | TCAAGTTTTGCCAACCATCGGA | | TGCTGTTGACAGTGAGCGCCCGATGGTTGGCAAAACTTGATAGTGAAGCCACAGATGTATCAAGTTTTGCCAACCATCGGATGCCTACTGCCTCGGA |
| **RASAL1** | RASAL1.3203 | | TTCTAGCAGACATTCTAGCGGG | | TGCTGTTGACAGTGAGCGACCGCTAGAATGTCTGCTAGAATAGTGAAGCCACAGATGTATTCTAGCAGACATTCTAGCGGGTGCCTACTGCCTCGGA |
| **RASAL1** | RASAL1.3203 | | TTCTAGCAGACATTCTAGCGGG | | TGCTGTTGACAGTGAGCGACCGCTAGAATGTCTGCTAGAATAGTGAAGCCACAGATGTATTCTAGCAGACATTCTAGCGGGTGCCTACTGCCTCGGA |
| **RASAL1** | RASAL1.3202 | | TCTAGCAGACATTCTAGCGGGT | | TGCTGTTGACAGTGAGCGCCCCGCTAGAATGTCTGCTAGATAGTGAAGCCACAGATGTATCTAGCAGACATTCTAGCGGGTTGCCTACTGCCTCGGA |
| **RASAL1** | RASAL1.3202 | | TCTAGCAGACATTCTAGCGGGT | | TGCTGTTGACAGTGAGCGCCCCGCTAGAATGTCTGCTAGATAGTGAAGCCACAGATGTATCTAGCAGACATTCTAGCGGGTTGCCTACTGCCTCGGA |
| **RASAL1** | RASAL1.3144 | | TTCTTGAACTACCTCATTGGAA | | TGCTGTTGACAGTGAGCGCTCCAATGAGGTAGTTCAAGAATAGTGAAGCCACAGATGTATTCTTGAACTACCTCATTGGAATGCCTACTGCCTCGGA |
| **RASAL1** | RASAL1.3144 | | TTCTTGAACTACCTCATTGGAA | | TGCTGTTGACAGTGAGCGCTCCAATGAGGTAGTTCAAGAATAGTGAAGCCACAGATGTATTCTTGAACTACCTCATTGGAATGCCTACTGCCTCGGA |
| **RASAL1** | RASAL1.2886 | | TTAGCAGACATGAAAACAGGAA | | TGCTGTTGACAGTGAGCGCTCCTGTTTTCATGTCTGCTAATAGTGAAGCCACAGATGTATTAGCAGACATGAAAACAGGAATGCCTACTGCCTCGGA |
| **RASAL1** | RASAL1.2886 | | TTAGCAGACATGAAAACAGGAA | | TGCTGTTGACAGTGAGCGCTCCTGTTTTCATGTCTGCTAATAGTGAAGCCACAGATGTATTAGCAGACATGAAAACAGGAATGCCTACTGCCTCGGA |
| **RASAL1** | RASAL1.1181 | | TAAACTGTTCCATCGACTTGGA | | TGCTGTTGACAGTGAGCGCCCAAGTCGATGGAACAGTTTATAGTGAAGCCACAGATGTATAAACTGTTCCATCGACTTGGATGCCTACTGCCTCGGA |
| **RASAL1** | RASAL1.1181 | | TAAACTGTTCCATCGACTTGGA | | TGCTGTTGACAGTGAGCGCCCAAGTCGATGGAACAGTTTATAGTGAAGCCACAGATGTATAAACTGTTCCATCGACTTGGATGCCTACTGCCTCGGA |
| **RASAL2** | RASAL2.9009 | | TATAGTAGCAACTTTAGTCTGA | | TGCTGTTGACAGTGAGCGCCAGACTAAAGTTGCTACTATATAGTGAAGCCACAGATGTATATAGTAGCAACTTTAGTCTGATGCCTACTGCCTCGGA |
| **RASAL2** | RASAL2.9009 | | TATAGTAGCAACTTTAGTCTGA | | TGCTGTTGACAGTGAGCGCCAGACTAAAGTTGCTACTATATAGTGAAGCCACAGATGTATATAGTAGCAACTTTAGTCTGATGCCTACTGCCTCGGA |
| **RASAL2** | RASAL2.6025 | | TAACTTAACATTTAACATCTAT | | TGCTGTTGACAGTGAGCGCTAGATGTTAAATGTTAAGTTATAGTGAAGCCACAGATGTATAACTTAACATTTAACATCTATTGCCTACTGCCTCGGA |
| **RASAL2** | RASAL2.6025 | | TAACTTAACATTTAACATCTAT | | TGCTGTTGACAGTGAGCGCTAGATGTTAAATGTTAAGTTATAGTGAAGCCACAGATGTATAACTTAACATTTAACATCTATTGCCTACTGCCTCGGA |
| **RASAL2** | RASAL2.3477 | | TTGTGAAGTAGCAACATTCTGT | | TGCTGTTGACAGTGAGCGCCAGAATGTTGCTACTTCACAATAGTGAAGCCACAGATGTATTGTGAAGTAGCAACATTCTGTTGCCTACTGCCTCGGA |
| **RASAL2** | RASAL2.3477 | | TTGTGAAGTAGCAACATTCTGT | | TGCTGTTGACAGTGAGCGCCAGAATGTTGCTACTTCACAATAGTGAAGCCACAGATGTATTGTGAAGTAGCAACATTCTGTTGCCTACTGCCTCGGA |
| **RASAL2** | RASAL2.8696 | | TATCATTGTAAAATAGTCGTGT | | TGCTGTTGACAGTGAGCGCCACGACTATTTTACAATGATATAGTGAAGCCACAGATGTATATCATTGTAAAATAGTCGTGTTGCCTACTGCCTCGGA |
| **RASAL2** | RASAL2.8696 | | TATCATTGTAAAATAGTCGTGT | | TGCTGTTGACAGTGAGCGCCACGACTATTTTACAATGATATAGTGAAGCCACAGATGTATATCATTGTAAAATAGTCGTGTTGCCTACTGCCTCGGA |
| **RASAL2** | RASAL2.459 | | TTAGGTTGAACTGTCCTGCGAA | | TGCTGTTGACAGTGAGCGCTCGCAGGACAGTTCAACCTAATAGTGAAGCCACAGATGTATTAGGTTGAACTGTCCTGCGAATGCCTACTGCCTCGGA |
| **RASAL2** | RASAL2.459 | | TTAGGTTGAACTGTCCTGCGAA | | TGCTGTTGACAGTGAGCGCTCGCAGGACAGTTCAACCTAATAGTGAAGCCACAGATGTATTAGGTTGAACTGTCCTGCGAATGCCTACTGCCTCGGA |
| **RASGRF1** | RASGRF1.2658 | | TACATTACTATATTCCTGCTAG | | TGCTGTTGACAGTGAGCGATAGCAGGAATATAGTAATGTATAGTGAAGCCACAGATGTATACATTACTATATTCCTGCTAGTGCCTACTGCCTCGGA |
| **RASGRF1** | RASGRF1.3029 | | TATGGTGTCCTTGACACTCTGG | | TGCTGTTGACAGTGAGCGACAGAGTGTCAAGGACACCATATAGTGAAGCCACAGATGTATATGGTGTCCTTGACACTCTGGTGCCTACTGCCTCGGA |
| **RASGRF1** | RASGRF1.1738 | | TACAGTATCATCTAGCACATGT | | TGCTGTTGACAGTGAGCGCCATGTGCTAGATGATACTGTATAGTGAAGCCACAGATGTATACAGTATCATCTAGCACATGTTGCCTACTGCCTCGGA |
| **RASGRF1** | RASGRF1.2342 | | TAATCCTAGAGCAAGGAGCGAC | | TGCTGTTGACAGTGAGCGATCGCTCCTTGCTCTAGGATTATAGTGAAGCCACAGATGTATAATCCTAGAGCAAGGAGCGACTGCCTACTGCCTCGGA |
| **RASGRF1** | RASGRF1.2771 | | TTGTCTGTCTGTTGTAGTGGAG | | TGCTGTTGACAGTGAGCGATCCACTACAACAGACAGACAATAGTGAAGCCACAGATGTATTGTCTGTCTGTTGTAGTGGAGTGCCTACTGCCTCGGA |
| **RBM14** | RBM14.1421 | | TTTAGCTATCATGTCTTCCTGG | | TGCTGTTGACAGTGAGCGACAGGAAGACATGATAGCTAAATAGTGAAGCCACAGATGTATTTAGCTATCATGTCTTCCTGGTGCCTACTGCCTCGGA |
| **RBM14** | RBM14.3781 | | TAAATCTAGACATAAAACCCAG | | TGCTGTTGACAGTGAGCGATGGGTTTTATGTCTAGATTTATAGTGAAGCCACAGATGTATAAATCTAGACATAAAACCCAGTGCCTACTGCCTCGGA |
| **RBM14** | RBM14.1281 | | TTTTCTAACACCACCTACTTGG | | TGCTGTTGACAGTGAGCGACAAGTAGGTGGTGTTAGAAAATAGTGAAGCCACAGATGTATTTTCTAACACCACCTACTTGGTGCCTACTGCCTCGGA |
| **RBM14** | RBM14.1437 | | TTAGCTATCATGTCTTCCTGGA | | TGCTGTTGACAGTGAGCGCCCAGGAAGACATGATAGCTAATAGTGAAGCCACAGATGTATTAGCTATCATGTCTTCCTGGATGCCTACTGCCTCGGA |
| **RBM14** | RBM14.1332 | | TTGTAGTCCATACAAAAGGGAA | | TGCTGTTGACAGTGAGCGCTCCCTTTTGTATGGACTACAATAGTGAAGCCACAGATGTATTGTAGTCCATACAAAAGGGAATGCCTACTGCCTCGGA |
| **RNF138** | RNF138.759 | | TTACATTAGTGAAACATCTTTT | | TGCTGTTGACAGTGAGCGCAAAGATGTTTCACTAATGTAATAGTGAAGCCACAGATGTATTACATTAGTGAAACATCTTTTTGCCTACTGCCTCGGA |
| **RNF138** | RNF138.2339 | | TAACTTGACATATTGGATGTAT | | TGCTGTTGACAGTGAGCGCTACATCCAATATGTCAAGTTATAGTGAAGCCACAGATGTATAACTTGACATATTGGATGTATTGCCTACTGCCTCGGA |
| **RNF138** | RNF138.2861 | | TACATCTCAAATAAGACTCTGT | | TGCTGTTGACAGTGAGCGCCAGAGTCTTATTTGAGATGTATAGTGAAGCCACAGATGTATACATCTCAAATAAGACTCTGTTGCCTACTGCCTCGGA |
| **RNF138** | RNF138.481 | | TTGGGTTTCTTCATCTAGCTGA | | TGCTGTTGACAGTGAGCGCCAGCTAGATGAAGAAACCCAATAGTGAAGCCACAGATGTATTGGGTTTCTTCATCTAGCTGATGCCTACTGCCTCGGA |
| **RNF138** | RNF138.2166 | | TAAAATGACAGAACAAAGGCGT | | TGCTGTTGACAGTGAGCGCCGCCTTTGTTCTGTCATTTTATAGTGAAGCCACAGATGTATAAAATGACAGAACAAAGGCGTTGCCTACTGCCTCGGA |
| **RUVBL1** | RUVBL1.487 | | TTCACCTTCATAAACTTCCTTG | | TGCTGTTGACAGTGAGCGAAAGGAAGTTTATGAAGGTGAATAGTGAAGCCACAGATGTATTCACCTTCATAAACTTCCTTGTGCCTACTGCCTCGGA |
| **RUVBL1** | RUVBL1.907 | | TTTGTCTGTGATTTCTGTCTTC | | TGCTGTTGACAGTGAGCGAAAGACAGAAATCACAGACAAATAGTGAAGCCACAGATGTATTTGTCTGTGATTTCTGTCTTCTGCCTACTGCCTCGGA |
| **RUVBL1** | RUVBL1.1673 | | TTATAGAAAACACACCAGGTAA | | TGCTGTTGACAGTGAGCGCTACCTGGTGTGTTTTCTATAATAGTGAAGCCACAGATGTATTATAGAAAACACACCAGGTAATGCCTACTGCCTCGGA |
| **RUVBL1** | RUVBL1.989 | | TCAACAAACAGCACACCCGGGA | | TGCTGTTGACAGTGAGCGCCCCGGGTGTGCTGTTTGTTGATAGTGAAGCCACAGATGTATCAACAAACAGCACACCCGGGATGCCTACTGCCTCGGA |
| **RUVBL1** | RUVBL1.1338 | | TTTAGCAAGCAAGTTGGCCGGG | | TGCTGTTGACAGTGAGCGACCGGCCAACTTGCTTGCTAAATAGTGAAGCCACAGATGTATTTAGCAAGCAAGTTGGCCGGGTGCCTACTGCCTCGGA |
| **SFPQ** | SFPQ.2323 | | TATTAGGTCAATAAACTGCTAA | | TGCTGTTGACAGTGAGCGCTAGCAGTTTATTGACCTAATATAGTGAAGCCACAGATGTATATTAGGTCAATAAACTGCTAATGCCTACTGCCTCGGA |
| **SFPQ** | SFPQ.1022 | | TAGTCTTTTGAATTCATCCTCC | | TGCTGTTGACAGTGAGCGAGAGGATGAATTCAAAAGACTATAGTGAAGCCACAGATGTATAGTCTTTTGAATTCATCCTCCTGCCTACTGCCTCGGA |
| **SFPQ** | SFPQ.1062 | | TTGTTGATAAAAACTTCTCCTG | | TGCTGTTGACAGTGAGCGAAGGAGAAGTTTTTATCAACAATAGTGAAGCCACAGATGTATTGTTGATAAAAACTTCTCCTGTGCCTACTGCCTCGGA |
| **SFPQ** | SFPQ.1734 | | TTGTGAAGTTCTTCCATGCGTC | | TGCTGTTGACAGTGAGCGAACGCATGGAAGAACTTCACAATAGTGAAGCCACAGATGTATTGTGAAGTTCTTCCATGCGTCTGCCTACTGCCTCGGA |
| **SFPQ** | SFPQ.1059 | | TTGATAAAAACTTCTCCTGGTT | | TGCTGTTGACAGTGAGCGCACCAGGAGAAGTTTTTATCAATAGTGAAGCCACAGATGTATTGATAAAAACTTCTCCTGGTTTGCCTACTGCCTCGGA |
| **SKI** | SKI.3286 | | TTGACTGCTCTATAAATCGGTA | | TGCTGTTGACAGTGAGCGCACCGATTTATAGAGCAGTCAATAGTGAAGCCACAGATGTATTGACTGCTCTATAAATCGGTATGCCTACTGCCTCGGA |
| **SKI** | SKI.4039 | | TTGCATAACAAAACCGACCCTT | | TGCTGTTGACAGTGAGCGCAGGGTCGGTTTTGTTATGCAATAGTGAAGCCACAGATGTATTGCATAACAAAACCGACCCTTTGCCTACTGCCTCGGA |
| **SKI** | SKI.5673 | | TTATTGTACAATGTCATCTGTT | | TGCTGTTGACAGTGAGCGCACAGATGACATTGTACAATAATAGTGAAGCCACAGATGTATTATTGTACAATGTCATCTGTTTGCCTACTGCCTCGGA |
| **SKI** | SKI.4968 | | TACATGTATAATACAACGGTGA | | TGCTGTTGACAGTGAGCGCCACCGTTGTATTATACATGTATAGTGAAGCCACAGATGTATACATGTATAATACAACGGTGATGCCTACTGCCTCGGA |
| **SKI** | SKI.5667 | | TACAATGTCATCTGTTTCGGGG | | TGCTGTTGACAGTGAGCGACCCGAAACAGATGACATTGTATAGTGAAGCCACAGATGTATACAATGTCATCTGTTTCGGGGTGCCTACTGCCTCGGA |
| **SMAD6** | SMAD6.865 | | TACATTGTAAAAATGACTCCAT | | TGCTGTTGACAGTGAGCGCTGGAGTCATTTTTACAATGTATAGTGAAGCCACAGATGTATACATTGTAAAAATGACTCCATTGCCTACTGCCTCGGA |
| **SMAD6** | SMAD6.941 | | TATTGAAAGAATTATAAGCCAA | | TGCTGTTGACAGTGAGCGCTGGCTTATAATTCTTTCAATATAGTGAAGCCACAGATGTATATTGAAAGAATTATAAGCCAATGCCTACTGCCTCGGA |
| **SMAD6** | SMAD6.82 | | TTTCAGTGTAAGACAATGTGGA | | TGCTGTTGACAGTGAGCGCCCACATTGTCTTACACTGAAATAGTGAAGCCACAGATGTATTTCAGTGTAAGACAATGTGGATGCCTACTGCCTCGGA |
| **SMAD6** | SMAD6.81 | | TTCAGTGTAAGACAATGTGGAA | | TGCTGTTGACAGTGAGCGCTCCACATTGTCTTACACTGAATAGTGAAGCCACAGATGTATTCAGTGTAAGACAATGTGGAATGCCTACTGCCTCGGA |
| **SMAD6** | SMAD6.1061 | | TATCTCAAAAACCATACACCAA | | TGCTGTTGACAGTGAGCGCTGGTGTATGGTTTTTGAGATATAGTGAAGCCACAGATGTATATCTCAAAAACCATACACCAATGCCTACTGCCTCGGA |
| **SMAD7** | SMAD7.390 | | TTGTGTACCAACAGCGTCCTGG | | TGCTGTTGACAGTGAGCGACAGGACGCTGTTGGTACACAATAGTGAAGCCACAGATGTATTGTGTACCAACAGCGTCCTGGTGCCTACTGCCTCGGA |
| **SMAD7** | SMAD7.1703 | | TTGAGCTAAGAACAGTGTCGAA | | TGCTGTTGACAGTGAGCGCTCGACACTGTTCTTAGCTCAATAGTGAAGCCACAGATGTATTGAGCTAAGAACAGTGTCGAATGCCTACTGCCTCGGA |
| **SMAD7** | SMAD7.1953 | | TTCTTGTTTATACACATTGCAC | | TGCTGTTGACAGTGAGCGATGCAATGTGTATAAACAAGAATAGTGAAGCCACAGATGTATTCTTGTTTATACACATTGCACTGCCTACTGCCTCGGA |
| **SMAD7** | SMAD7.2099 | | TTTAATGGAACATAAACTCCTT | | TGCTGTTGACAGTGAGCGCAGGAGTTTATGTTCCATTAAATAGTGAAGCCACAGATGTATTTAATGGAACATAAACTCCTTTGCCTACTGCCTCGGA |
| **SMAD7** | SMAD7.239 | | TTGTTGTCCGAATTGAGCTGTC | | TGCTGTTGACAGTGAGCGAACAGCTCAATTCGGACAACAATAGTGAAGCCACAGATGTATTGTTGTCCGAATTGAGCTGTCTGCCTACTGCCTCGGA |
| **SMC3** | SMC3.1337 | | TTTCTTGTCATTAATAGCCTGA | | TGCTGTTGACAGTGAGCGCCAGGCTATTAATGACAAGAAATAGTGAAGCCACAGATGTATTTCTTGTCATTAATAGCCTGATGCCTACTGCCTCGGA |
| **SMC3** | SMC3.1442 | | TTTGACTTCATTAAGATCCTGG | | TGCTGTTGACAGTGAGCGACAGGATCTTAATGAAGTCAAATAGTGAAGCCACAGATGTATTTGACTTCATTAAGATCCTGGTGCCTACTGCCTCGGA |
| **SMC3** | SMC3.860 | | TTCTCCACTAGTCTCTCGCTTA | | TGCTGTTGACAGTGAGCGCAAGCGAGAGACTAGTGGAGAATAGTGAAGCCACAGATGTATTCTCCACTAGTCTCTCGCTTATGCCTACTGCCTCGGA |
| **SMC3** | SMC3.905 | | TTTATCTCTTGCATCCTGCTGA | | TGCTGTTGACAGTGAGCGCCAGCAGGATGCAAGAGATAAATAGTGAAGCCACAGATGTATTTATCTCTTGCATCCTGCTGATGCCTACTGCCTCGGA |
| **SMC3** | SMC3.3106 | | TAACTTTTCTTTCTGCTCGGAG | | TGCTGTTGACAGTGAGCGATCCGAGCAGAAAGAAAAGTTATAGTGAAGCCACAGATGTATAACTTTTCTTTCTGCTCGGAGTGCCTACTGCCTCGGA |
| **SMURF2** | SMURF2.1967 | | TAAACTGTTGTGAAGATCCGGA | | TGCTGTTGACAGTGAGCGCCCGGATCTTCACAACAGTTTATAGTGAAGCCACAGATGTATAAACTGTTGTGAAGATCCGGATGCCTACTGCCTCGGA |
| **SMURF2** | SMURF2.1494 | | TTTCTGAACCAGGTCTCGCTTG | | TGCTGTTGACAGTGAGCGAAAGCGAGACCTGGTTCAGAAATAGTGAAGCCACAGATGTATTTCTGAACCAGGTCTCGCTTGTGCCTACTGCCTCGGA |
| **SMURF2** | SMURF2.3699 | | TTTAGAGCAAAATTCTTCCTTT | | TGCTGTTGACAGTGAGCGCAAGGAAGAATTTTGCTCTAAATAGTGAAGCCACAGATGTATTTAGAGCAAAATTCTTCCTTTTGCCTACTGCCTCGGA |
| **SMURF2** | SMURF2.3622 | | TTAATGGAAACTTACACTGTAG | | TGCTGTTGACAGTGAGCGATACAGTGTAAGTTTCCATTAATAGTGAAGCCACAGATGTATTAATGGAAACTTACACTGTAGTGCCTACTGCCTCGGA |
| **SMURF2** | SMURF2.633 | | TTTCTTATGGATCTTCTTGTGA | | TGCTGTTGACAGTGAGCGCCACAAGAAGATCCATAAGAAATAGTGAAGCCACAGATGTATTTCTTATGGATCTTCTTGTGATGCCTACTGCCTCGGA |
| **SNRPB** | SNRPB.928 | | TAAAAGGACTATGTACAGCCTT | | TGCTGTTGACAGTGAGCGCAGGCTGTACATAGTCCTTTTATAGTGAAGCCACAGATGTATAAAAGGACTATGTACAGCCTTTGCCTACTGCCTCGGA |
| **SNRPB** | SNRPB.20 | | TAGAAACCTACTTCCGGTCCAG | | TGCTGTTGACAGTGAGCGATGGACCGGAAGTAGGTTTCTATAGTGAAGCCACAGATGTATAGAAACCTACTTCCGGTCCAGTGCCTACTGCCTCGGA |
| **SNRPB** | SNRPB.391 | | TCTACTGTCATTGAGACCAGAT | | TGCTGTTGACAGTGAGCGCTCTGGTCTCAATGACAGTAGATAGTGAAGCCACAGATGTATCTACTGTCATTGAGACCAGATTGCCTACTGCCTCGGA |
| **SNRPB** | SNRPB.292 | | TTTCTGAACTCATCACAGTCAC | | TGCTGTTGACAGTGAGCGATGACTGTGATGAGTTCAGAAATAGTGAAGCCACAGATGTATTTCTGAACTCATCACAGTCACTGCCTACTGCCTCGGA |
| **SNRPB** | SNRPB.329 | | TTCCCTTTCTGCTTGTTTGGAG | | TGCTGTTGACAGTGAGCGATCCAAACAAGCAGAAAGGGAATAGTGAAGCCACAGATGTATTCCCTTTCTGCTTGTTTGGAGTGCCTACTGCCTCGGA |
| **SNRPD1** | SNRPD1.1118 | | TATATTGTTAAGTTTAGCCTAA | | TGCTGTTGACAGTGAGCGCTAGGCTAAACTTAACAATATATAGTGAAGCCACAGATGTATATATTGTTAAGTTTAGCCTAATGCCTACTGCCTCGGA |
| **SNRPD1** | SNRPD1.1049 | | TAGAACTGTAGCCTAACTGGAC | | TGCTGTTGACAGTGAGCGATCCAGTTAGGCTACAGTTCTATAGTGAAGCCACAGATGTATAGAACTGTAGCCTAACTGGACTGCCTACTGCCTCGGA |
| **SNRPD1** | SNRPD1.582 | | TAAATAGACAGCTCTGTCCCAC | | TGCTGTTGACAGTGAGCGATGGGACAGAGCTGTCTATTTATAGTGAAGCCACAGATGTATAAATAGACAGCTCTGTCCCACTGCCTACTGCCTCGGA |
| **SNRPD1** | SNRPD1.1061 | | TTTCTCAGTACATAGAACTGTA | | TGCTGTTGACAGTGAGCGCACAGTTCTATGTACTGAGAAATAGTGAAGCCACAGATGTATTTCTCAGTACATAGAACTGTATGCCTACTGCCTCGGA |
| **SNRPD1** | SNRPD1.1469 | | TAACTGATAATCTAGTGACCAT | | TGCTGTTGACAGTGAGCGCTGGTCACTAGATTATCAGTTATAGTGAAGCCACAGATGTATAACTGATAATCTAGTGACCATTGCCTACTGCCTCGGA |
| **SOWAHC** | SOWAHC.2451 | | TTAATGTACCTTAACACTGGTG | | TGCTGTTGACAGTGAGCGAACCAGTGTTAAGGTACATTAATAGTGAAGCCACAGATGTATTAATGTACCTTAACACTGGTGTGCCTACTGCCTCGGA |
| **SOWAHC** | SOWAHC.3222 | | TTAGGTTTCAGCAACTACGCGA | | TGCTGTTGACAGTGAGCGCCGCGTAGTTGCTGAAACCTAATAGTGAAGCCACAGATGTATTAGGTTTCAGCAACTACGCGATGCCTACTGCCTCGGA |
| **SPRED1** | SPRED1.5564 | | TTCAACACAAGAAATATCCTTA | | TGCTGTTGACAGTGAGCGCAAGGATATTTCTTGTGTTGAATAGTGAAGCCACAGATGTATTCAACACAAGAAATATCCTTATGCCTACTGCCTCGGA |
| **SPRED1** | SPRED1.5123 | | TAAAATACCAAAACTTAGCTGG | | TGCTGTTGACAGTGAGCGACAGCTAAGTTTTGGTATTTTATAGTGAAGCCACAGATGTATAAAATACCAAAACTTAGCTGGTGCCTACTGCCTCGGA |
| **SPRED1** | SPRED1.1060 | | TATTCTGACAATCTCATCCTCA | | TGCTGTTGACAGTGAGCGCGAGGATGAGATTGTCAGAATATAGTGAAGCCACAGATGTATATTCTGACAATCTCATCCTCATGCCTACTGCCTCGGA |
| **SPRED1** | SPRED1.6831 | | TATTACTGTCCTAATAACCTTT | | TGCTGTTGACAGTGAGCGCAAGGTTATTAGGACAGTAATATAGTGAAGCCACAGATGTATATTACTGTCCTAATAACCTTTTGCCTACTGCCTCGGA |
| **SPRED1** | SPRED1.5651 | | TACACTTGCTTAATGCACCTTT | | TGCTGTTGACAGTGAGCGCAAGGTGCATTAAGCAAGTGTATAGTGAAGCCACAGATGTATACACTTGCTTAATGCACCTTTTGCCTACTGCCTCGGA |
| **SPRED2** | SPRED2.418 | | TAGAAGAACTGTCTGTAGCTGT | | TGCTGTTGACAGTGAGCGCCAGCTACAGACAGTTCTTCTATAGTGAAGCCACAGATGTATAGAAGAACTGTCTGTAGCTGTTGCCTACTGCCTCGGA |
| **SPRED2** | SPRED2.1853 | | TAAAGATAGAAACTGCAGCTTA | | TGCTGTTGACAGTGAGCGCAAGCTGCAGTTTCTATCTTTATAGTGAAGCCACAGATGTATAAAGATAGAAACTGCAGCTTATGCCTACTGCCTCGGA |
| **SPRED2** | SPRED2.2948 | | TAATCTACTAAAGAAAATCGAT | | TGCTGTTGACAGTGAGCGCTCGATTTTCTTTAGTAGATTATAGTGAAGCCACAGATGTATAATCTACTAAAGAAAATCGATTGCCTACTGCCTCGGA |
| **SPRED2** | SPRED2.1588 | | TTGCAGACTCCTTTGAACTGGA | | TGCTGTTGACAGTGAGCGCCCAGTTCAAAGGAGTCTGCAATAGTGAAGCCACAGATGTATTGCAGACTCCTTTGAACTGGATGCCTACTGCCTCGGA |
| **SPRED2** | SPRED2.1496 | | TAGTACAGGAGTCTGTGGCGAA | | TGCTGTTGACAGTGAGCGCTCGCCACAGACTCCTGTACTATAGTGAAGCCACAGATGTATAGTACAGGAGTCTGTGGCGAATGCCTACTGCCTCGGA |
| **SPRY1** | SPRY1.2179 | | TAACATATGTACTTTCTTCGTG | | TGCTGTTGACAGTGAGCGAACGAAGAAAGTACATATGTTATAGTGAAGCCACAGATGTATAACATATGTACTTTCTTCGTGTGCCTACTGCCTCGGA |
| **SPRY1** | SPRY1.587 | | TTCACAAATGAACTTGTGCTGT | | TGCTGTTGACAGTGAGCGCCAGCACAAGTTCATTTGTGAATAGTGAAGCCACAGATGTATTCACAAATGAACTTGTGCTGTTGCCTACTGCCTCGGA |
| **SPRY1** | SPRY1.718 | | TTGACTAAGCACATGCAGGTTC | | TGCTGTTGACAGTGAGCGAAACCTGCATGTGCTTAGTCAATAGTGAAGCCACAGATGTATTGACTAAGCACATGCAGGTTCTGCCTACTGCCTCGGA |
| **SPRY1** | SPRY1.311 | | TTATTATTCACATTAATTGGTA | | TGCTGTTGACAGTGAGCGCACCAATTAATGTGAATAATAATAGTGAAGCCACAGATGTATTATTATTCACATTAATTGGTATGCCTACTGCCTCGGA |
| **SPRY1** | SPRY1.137 | | TCATAGTCTAATCTCTGACGGC | | TGCTGTTGACAGTGAGCGACCGTCAGAGATTAGACTATGATAGTGAAGCCACAGATGTATCATAGTCTAATCTCTGACGGCTGCCTACTGCCTCGGA |
| **SPRY2** | SPRY2.328 | | TAGAACACATCTGAACTCCGTG | | TGCTGTTGACAGTGAGCGAACGGAGTTCAGATGTGTTCTATAGTGAAGCCACAGATGTATAGAACACATCTGAACTCCGTGTGCCTACTGCCTCGGA |
| **SPRY2** | SPRY2.1747 | | TAGTATAATATTTTGTGTCTGT | | TGCTGTTGACAGTGAGCGCCAGACACAAAATATTATACTATAGTGAAGCCACAGATGTATAGTATAATATTTTGTGTCTGTTGCCTACTGCCTCGGA |
| **SPRY2** | SPRY2.2060 | | TATATTGGACATATGCATCTGT | | TGCTGTTGACAGTGAGCGCCAGATGCATATGTCCAATATATAGTGAAGCCACAGATGTATATATTGGACATATGCATCTGTTGCCTACTGCCTCGGA |
| **SPRY2** | SPRY2.1553 | | TTAGCTTATGCAATACATGGGT | | TGCTGTTGACAGTGAGCGCCCCATGTATTGCATAAGCTAATAGTGAAGCCACAGATGTATTAGCTTATGCAATACATGGGTTGCCTACTGCCTCGGA |
| **SPRY2** | SPRY2.329 | | TTAGAACACATCTGAACTCCGT | | TGCTGTTGACAGTGAGCGCCGGAGTTCAGATGTGTTCTAATAGTGAAGCCACAGATGTATTAGAACACATCTGAACTCCGTTGCCTACTGCCTCGGA |
| **SPRY3** | SPRY3.6580 | | TTTAACAGCAGCAACACTGGTA | | TGCTGTTGACAGTGAGCGCACCAGTGTTGCTGCTGTTAAATAGTGAAGCCACAGATGTATTTAACAGCAGCAACACTGGTATGCCTACTGCCTCGGA |
| **SPRY3** | SPRY3.6604 | | TTCCTTATCTTGTCCATCCTGG | | TGCTGTTGACAGTGAGCGACAGGATGGACAAGATAAGGAATAGTGAAGCCACAGATGTATTCCTTATCTTGTCCATCCTGGTGCCTACTGCCTCGGA |
| **SPRY3** | SPRY3.7990 | | TTGCATATTATAATCATCTGGG | | TGCTGTTGACAGTGAGCGACCAGATGATTATAATATGCAATAGTGAAGCCACAGATGTATTGCATATTATAATCATCTGGGTGCCTACTGCCTCGGA |
| **SPRY3** | SPRY3.2842 | | TTCTTTTCCACACTTGAGCTTC | | TGCTGTTGACAGTGAGCGAAAGCTCAAGTGTGGAAAAGAATAGTGAAGCCACAGATGTATTCTTTTCCACACTTGAGCTTCTGCCTACTGCCTCGGA |
| **SPRY3** | SPRY3.7497 | | TTAGGTGAGAGAAACACACTGA | | TGCTGTTGACAGTGAGCGCCAGTGTGTTTCTCTCACCTAATAGTGAAGCCACAGATGTATTAGGTGAGAGAAACACACTGATGCCTACTGCCTCGGA |
| **SPRY4** | SPRY4.4393 | | TTCAAGGTGACAGTCATCCGGG | | TGCTGTTGACAGTGAGCGACCGGATGACTGTCACCTTGAATAGTGAAGCCACAGATGTATTCAAGGTGACAGTCATCCGGGTGCCTACTGCCTCGGA |
| **SPRY4** | SPRY4.2099 | | TAAAACACCGTTAGCATCCGGT | | TGCTGTTGACAGTGAGCGCCCGGATGCTAACGGTGTTTTATAGTGAAGCCACAGATGTATAAAACACCGTTAGCATCCGGTTGCCTACTGCCTCGGA |
| **SPRY4** | SPRY4.2909 | | TAAGATGTTACTACTAGTCTTC | | TGCTGTTGACAGTGAGCGAAAGACTAGTAGTAACATCTTATAGTGAAGCCACAGATGTATAAGATGTTACTACTAGTCTTCTGCCTACTGCCTCGGA |
| **SPRY4** | SPRY4.4659 | | TTGTAACATGAAACCAAGCTGT | | TGCTGTTGACAGTGAGCGCCAGCTTGGTTTCATGTTACAATAGTGAAGCCACAGATGTATTGTAACATGAAACCAAGCTGTTGCCTACTGCCTCGGA |
| **SPRY4** | SPRY4.2067 | | TATAAAAACATAATGACTGGAT | | TGCTGTTGACAGTGAGCGCTCCAGTCATTATGTTTTTATATAGTGAAGCCACAGATGTATATAAAAACATAATGACTGGATTGCCTACTGCCTCGGA |
| **SRSF1** | SRSF1.3300 | | TTAACATTTAATACTTACCTTT | | TGCTGTTGACAGTGAGCGCAAGGTAAGTATTAAATGTTAATAGTGAAGCCACAGATGTATTAACATTTAATACTTACCTTTTGCCTACTGCCTCGGA |
| **SRSF1** | SRSF1.4112 | | TAAACTTACACTAAGTACTTAG | | TGCTGTTGACAGTGAGCGATAAGTACTTAGTGTAAGTTTATAGTGAAGCCACAGATGTATAAACTTACACTAAGTACTTAGTGCCTACTGCCTCGGA |
| **SRSF1** | SRSF1.3678 | | TAATCTTGTGCACTCTTCCCTT | | TGCTGTTGACAGTGAGCGCAGGGAAGAGTGCACAAGATTATAGTGAAGCCACAGATGTATAATCTTGTGCACTCTTCCCTTTGCCTACTGCCTCGGA |
| **SRSF1** | SRSF1.1216 | | TTACACAATATCACAGTCTGAA | | TGCTGTTGACAGTGAGCGCTCAGACTGTGATATTGTGTAATAGTGAAGCCACAGATGTATTACACAATATCACAGTCTGAATGCCTACTGCCTCGGA |
| **SRSF1** | SRSF1.2656 | | TTTATCTCCAGGTCCTCGCTTT | | TGCTGTTGACAGTGAGCGCAAGCGAGGACCTGGAGATAAATAGTGAAGCCACAGATGTATTTATCTCCAGGTCCTCGCTTTTGCCTACTGCCTCGGA |
| **SRSF10** | SRSF10.613 | | TAGTTGTAATCAAAAGACCGAC | | TGCTGTTGACAGTGAGCGATCGGTCTTTTGATTACAACTATAGTGAAGCCACAGATGTATAGTTGTAATCAAAAGACCGACTGCCTACTGCCTCGGA |
| **SRSF10** | SRSF10.371 | | TTGAACATAAGCAAATCCTCTT | | TGCTGTTGACAGTGAGCGCAGAGGATTTGCTTATGTTCAATAGTGAAGCCACAGATGTATTGAACATAAGCAAATCCTCTTTGCCTACTGCCTCGGA |
| **SRSF10** | SRSF10.567 | | TTCGGCTTCTAGAACGTCTGTA | | TGCTGTTGACAGTGAGCGCACAGACGTTCTAGAAGCCGAATAGTGAAGCCACAGATGTATTCGGCTTCTAGAACGTCTGTATGCCTACTGCCTCGGA |
| **SRSF10** | SRSF10.319 | | TACACATCAACTATAGGACCAT | | TGCTGTTGACAGTGAGCGCTGGTCCTATAGTTGATGTGTATAGTGAAGCCACAGATGTATACACATCAACTATAGGACCATTGCCTACTGCCTCGGA |
| **SRSF10** | SRSF10.571 | | TAACTTCGGCTTCTAGAACGTC | | TGCTGTTGACAGTGAGCGAACGTTCTAGAAGCCGAAGTTATAGTGAAGCCACAGATGTATAACTTCGGCTTCTAGAACGTCTGCCTACTGCCTCGGA |
| **SRSF2** | SRSF2.1937 | | TTAAACTACAGAAACAATGGTT | | TGCTGTTGACAGTGAGCGCACCATTGTTTCTGTAGTTTAATAGTGAAGCCACAGATGTATTAAACTACAGAAACAATGGTTTGCCTACTGCCTCGGA |
| **SRSF2** | SRSF2.1488 | | TTGTGTTTGATAAACAATCCTT | | TGCTGTTGACAGTGAGCGCAGGATTGTTTATCAAACACAATAGTGAAGCCACAGATGTATTGTGTTTGATAAACAATCCTTTGCCTACTGCCTCGGA |
| **SRSF2** | SRSF2.1919 | | TAAACTACAGAAACAATGGTTA | | TGCTGTTGACAGTGAGCGCAACCATTGTTTCTGTAGTTTATAGTGAAGCCACAGATGTATAAACTACAGAAACAATGGTTATGCCTACTGCCTCGGA |
| **SRSF2** | SRSF2.1545 | | TCAACTGCTACACAACTGCGCC | | TGCTGTTGACAGTGAGCGAGCGCAGTTGTGTAGCAGTTGATAGTGAAGCCACAGATGTATCAACTGCTACACAACTGCGCCTGCCTACTGCCTCGGA |
| **SRSF2** | SRSF2.1723 | | TTATTTATATGCAAGGCCCGGG | | TGCTGTTGACAGTGAGCGACCGGGCCTTGCATATAAATAATAGTGAAGCCACAGATGTATTATTTATATGCAAGGCCCGGGTGCCTACTGCCTCGGA |
| **SRSF3** | SRSF3.2297 | | TTAACTTTAAGGCTGAACCTTC | | TGCTGTTGACAGTGAGCGAAAGGTTCAGCCTTAAAGTTAATAGTGAAGCCACAGATGTATTAACTTTAAGGCTGAACCTTCTGCCTACTGCCTCGGA |
| **SRSF3** | SRSF3.1983 | | TTACATTTGAACCATATTGTGA | | TGCTGTTGACAGTGAGCGCCACAATATGGTTCAAATGTAATAGTGAAGCCACAGATGTATTACATTTGAACCATATTGTGATGCCTACTGCCTCGGA |
| **SRSF3** | SRSF3.774 | | TTGAACAGCTAAAACATCTTAA | | TGCTGTTGACAGTGAGCGCTAAGATGTTTTAGCTGTTCAATAGTGAAGCCACAGATGTATTGAACAGCTAAAACATCTTAATGCCTACTGCCTCGGA |
| **SRSF3** | SRSF3.1204 | | TTCTTGAAACTTAACATTCTAT | | TGCTGTTGACAGTGAGCGCTAGAATGTTAAGTTTCAAGAATAGTGAAGCCACAGATGTATTCTTGAAACTTAACATTCTATTGCCTACTGCCTCGGA |
| **SRSF3** | SRSF3.941 | | TAGAGGTTTATTATCAGTCTGT | | TGCTGTTGACAGTGAGCGCCAGACTGATAATAAACCTCTATAGTGAAGCCACAGATGTATAGAGGTTTATTATCAGTCTGTTGCCTACTGCCTCGGA |
| **SSRP1** | SSRP1.2236 | | TTGCTCTTGAAGCTCTCGCTTA | | TGCTGTTGACAGTGAGCGCAAGCGAGAGCTTCAAGAGCAATAGTGAAGCCACAGATGTATTGCTCTTGAAGCTCTCGCTTATGCCTACTGCCTCGGA |
| **SSRP1** | SSRP1.1950 | | TCTGACTTGATCTTCTCTCGGC | | TGCTGTTGACAGTGAGCGACCGAGAGAAGATCAAGTCAGATAGTGAAGCCACAGATGTATCTGACTTGATCTTCTCTCGGCTGCCTACTGCCTCGGA |
| **SSRP1** | SSRP1.2168 | | TTTCTTTTCCATCTTTACCTTT | | TGCTGTTGACAGTGAGCGCAAGGTAAAGATGGAAAAGAAATAGTGAAGCCACAGATGTATTTCTTTTCCATCTTTACCTTTTGCCTACTGCCTCGGA |
| **SSRP1** | SSRP1.2149 | | TTTACTTTCTTCTTCTTCTTTG | | TGCTGTTGACAGTGAGCGAAAAGAAGAAGAAGAAAGTAAATAGTGAAGCCACAGATGTATTTACTTTCTTCTTCTTCTTTGTGCCTACTGCCTCGGA |
| **SSRP1** | SSRP1.2143 | | TTCTTCTTCTTCTTTGACTTGT | | TGCTGTTGACAGTGAGCGCCAAGTCAAAGAAGAAGAAGAATAGTGAAGCCACAGATGTATTCTTCTTCTTCTTTGACTTGTTGCCTACTGCCTCGGA |
| **STRAP** | STRAP.683 | | TAACAAATAATTACTATCCTGC | | TGCTGTTGACAGTGAGCGACAGGATAGTAATTATTTGTTATAGTGAAGCCACAGATGTATAACAAATAATTACTATCCTGCTGCCTACTGCCTCGGA |
| **STRAP** | STRAP.1693 | | TAAACAGCTACAAGAACCCTAA | | TGCTGTTGACAGTGAGCGCTAGGGTTCTTGTAGCTGTTTATAGTGAAGCCACAGATGTATAAACAGCTACAAGAACCCTAATGCCTACTGCCTCGGA |
| **STRAP** | STRAP.683 | | TTAACAAATAATTACTATCCTG | | TGCTGTTGACAGTGAGCGAAGGATAGTAATTATTTGTTAATAGTGAAGCCACAGATGTATTAACAAATAATTACTATCCTGTGCCTACTGCCTCGGA |
| **STRAP** | STRAP.859 | | TGTCATAGTAGCATGATCCCAA | | TGCTGTTGACAGTGAGCGCTGGGATCATGCTACTATGACATAGTGAAGCCACAGATGTATGTCATAGTAGCATGATCCCAATGCCTACTGCCTCGGA |
| **STRAP** | STRAP.1476 | | TAAGCAGACAGTAACTCTGGAA | | TGCTGTTGACAGTGAGCGCTCCAGAGTTACTGTCTGCTTATAGTGAAGCCACAGATGTATAAGCAGACAGTAACTCTGGAATGCCTACTGCCTCGGA |
| **TCERG1** | TCERG1.2467 | | TTGATGAACTATCTACTGCTTT | | TGCTGTTGACAGTGAGCGCAAGCAGTAGATAGTTCATCAATAGTGAAGCCACAGATGTATTGATGAACTATCTACTGCTTTTGCCTACTGCCTCGGA |
| **TCERG1** | TCERG1.1866 | | TTTATTATCGTCTCTCTTCCGT | | TGCTGTTGACAGTGAGCGCCGGAAGAGAGACGATAATAAATAGTGAAGCCACAGATGTATTTATTATCGTCTCTCTTCCGTTGCCTACTGCCTCGGA |
| **TCERG1** | TCERG1.3848 | | TAAACAACAAGACTCGGTCTAT | | TGCTGTTGACAGTGAGCGCTAGACCGAGTCTTGTTGTTTATAGTGAAGCCACAGATGTATAAACAACAAGACTCGGTCTATTGCCTACTGCCTCGGA |
| **TCERG1** | TCERG1.1882 | | TTATTATCGTCTCTCTTCCGTT | | TGCTGTTGACAGTGAGCGCACGGAAGAGAGACGATAATAATAGTGAAGCCACAGATGTATTATTATCGTCTCTCTTCCGTTTGCCTACTGCCTCGGA |
| **TCERG1** | TCERG1.2725 | | TATCAGACCATGACACATCTGA | | TGCTGTTGACAGTGAGCGCCAGATGTGTCATGGTCTGATATAGTGAAGCCACAGATGTATATCAGACCATGACACATCTGATGCCTACTGCCTCGGA |
| **TCOF1** | TCOF1.313 | | TTTCTTAGCTTGCAGTGCCGCA | | TGCTGTTGACAGTGAGCGCGCGGCACTGCAAGCTAAGAAATAGTGAAGCCACAGATGTATTTCTTAGCTTGCAGTGCCGCATGCCTACTGCCTCGGA |
| **TCOF1** | TCOF1.2214 | | TTTACTGAGGTTTTCACCTGTG | | TGCTGTTGACAGTGAGCGAACAGGTGAAAACCTCAGTAAATAGTGAAGCCACAGATGTATTTACTGAGGTTTTCACCTGTGTGCCTACTGCCTCGGA |
| **TCOF1** | TCOF1.459 | | TTTTCTTTCATGCTTGATGGCA | | TGCTGTTGACAGTGAGCGCGCCATCAAGCATGAAAGAAAATAGTGAAGCCACAGATGTATTTTCTTTCATGCTTGATGGCATGCCTACTGCCTCGGA |
| **TCOF1** | TCOF1.458 | | TTTCTTTCATGCTTGATGGCAA | | TGCTGTTGACAGTGAGCGCTGCCATCAAGCATGAAAGAAATAGTGAAGCCACAGATGTATTTCTTTCATGCTTGATGGCAATGCCTACTGCCTCGGA |
| **TCOF1** | TCOF1.312 | | TTCTTAGCTTGCAGTGCCGCAT | | TGCTGTTGACAGTGAGCGCTGCGGCACTGCAAGCTAAGAATAGTGAAGCCACAGATGTATTCTTAGCTTGCAGTGCCGCATTGCCTACTGCCTCGGA |
| **TFAM** | TFAM.455 | | TATCTCTTCTTTATATACCTGC | | TGCTGTTGACAGTGAGCGACAGGTATATAAAGAAGAGATATAGTGAAGCCACAGATGTATATCTCTTCTTTATATACCTGCTGCCTACTGCCTCGGA |
| **TFAM** | TFAM.2312 | | TTCTAAAACAAGAACATCTGAA | | TGCTGTTGACAGTGAGCGCTCAGATGTTCTTGTTTTAGAATAGTGAAGCCACAGATGTATTCTAAAACAAGAACATCTGAATGCCTACTGCCTCGGA |
| **TFAM** | TFAM.465 | | TAAATCTGCTTATCTCTTCTTT | | TGCTGTTGACAGTGAGCGCAAGAAGAGATAAGCAGATTTATAGTGAAGCCACAGATGTATAAATCTGCTTATCTCTTCTTTTGCCTACTGCCTCGGA |
| **TFAM** | TFAM.674 | | TTCCTTTACAGTCTTCAGCTTT | | TGCTGTTGACAGTGAGCGCAAGCTGAAGACTGTAAAGGAATAGTGAAGCCACAGATGTATTCCTTTACAGTCTTCAGCTTTTGCCTACTGCCTCGGA |
| **TFAM** | TFAM.3304 | | TTAAGTATAAAATAATACGGGT | | TGCTGTTGACAGTGAGCGCCCCGTATTATTTTATACTTAATAGTGAAGCCACAGATGTATTAAGTATAAAATAATACGGGTTGCCTACTGCCTCGGA |
| **TFDP1** | TFDP1.1542 | | TAGCTTACCAATATCTTCTTGG | | TGCTGTTGACAGTGAGCGACAAGAAGATATTGGTAAGCTATAGTGAAGCCACAGATGTATAGCTTACCAATATCTTCTTGGTGCCTACTGCCTCGGA |
| **TFDP1** | TFDP1.865 | | TTGAAGTTGAGACTGTTTCTGT | | TGCTGTTGACAGTGAGCGCCAGAAACAGTCTCAACTTCAATAGTGAAGCCACAGATGTATTGAAGTTGAGACTGTTTCTGTTGCCTACTGCCTCGGA |
| **TFDP1** | TFDP1.2514 | | TTCAATAAAAAGACTATCGTTT | | TGCTGTTGACAGTGAGCGCAACGATAGTCTTTTTATTGAATAGTGAAGCCACAGATGTATTCAATAAAAAGACTATCGTTTTGCCTACTGCCTCGGA |
| **TFDP1** | TFDP1.841 | | TATTCTTTCAAGTCTCCTCTGT | | TGCTGTTGACAGTGAGCGCCAGAGGAGACTTGAAAGAATATAGTGAAGCCACAGATGTATATTCTTTCAAGTCTCCTCTGTTGCCTACTGCCTCGGA |
| **TFDP1** | TFDP1.2064 | | TTTGTTGCTGCAAAACAGGGAG | | TGCTGTTGACAGTGAGCGATCCCTGTTTTGCAGCAACAAATAGTGAAGCCACAGATGTATTTGTTGCTGCAAAACAGGGAGTGCCTACTGCCTCGGA |
| **TGIF1** | TGIF1.787 | | TTAAGCTGTAAGTTTTGCCTGA | | TGCTGTTGACAGTGAGCGCCAGGCAAAACTTACAGCTTAATAGTGAAGCCACAGATGTATTAAGCTGTAAGTTTTGCCTGATGCCTACTGCCTCGGA |
| **TGIF1** | TGIF1.962 | | TTTAAGTTTATAGTTCTTGGAA | | TGCTGTTGACAGTGAGCGCTCCAAGAACTATAAACTTAAATAGTGAAGCCACAGATGTATTTAAGTTTATAGTTCTTGGAATGCCTACTGCCTCGGA |
| **TGIF1** | TGIF1.954 | | TATAGTTCTTGGAATGACTGTA | | TGCTGTTGACAGTGAGCGCACAGTCATTCCAAGAACTATATAGTGAAGCCACAGATGTATATAGTTCTTGGAATGACTGTATGCCTACTGCCTCGGA |
| **TGIF1** | TGIF1.199 | | TAGACAGGTGTGTTTGCTGGGA | | TGCTGTTGACAGTGAGCGCCCCAGCAAACACACCTGTCTATAGTGAAGCCACAGATGTATAGACAGGTGTGTTTGCTGGGATGCCTACTGCCTCGGA |
| **TGIF1** | TGIF1.510 | | TTTCAATGCAGTCACAGTGGTA | | TGCTGTTGACAGTGAGCGCACCACTGTGACTGCATTGAAATAGTGAAGCCACAGATGTATTTCAATGCAGTCACAGTGGTATGCCTACTGCCTCGGA |
| **TIMELESS** | TIMELESS.4012 | | TAAATCTCCAGAGAGCTGCTGG | | TGCTGTTGACAGTGAGCGACAGCAGCTCTCTGGAGATTTATAGTGAAGCCACAGATGTATAAATCTCCAGAGAGCTGCTGGTGCCTACTGCCTCGGA |
| **TIMELESS** | TIMELESS.4984 | | TTGCAGTATTTATCCATCCTTT | | TGCTGTTGACAGTGAGCGCAAGGATGGATAAATACTGCAATAGTGAAGCCACAGATGTATTGCAGTATTTATCCATCCTTTTGCCTACTGCCTCGGA |
| **TIMELESS** | TIMELESS.939 | | TTTTCTGCCATCTCTCGCTGGC | | TGCTGTTGACAGTGAGCGACCAGCGAGAGATGGCAGAAAATAGTGAAGCCACAGATGTATTTTCTGCCATCTCTCGCTGGCTGCCTACTGCCTCGGA |
| **TIMELESS** | TIMELESS.1233 | | TTTACTGATCCCATGAGCCGGT | | TGCTGTTGACAGTGAGCGCCCGGCTCATGGGATCAGTAAATAGTGAAGCCACAGATGTATTTACTGATCCCATGAGCCGGTTGCCTACTGCCTCGGA |
| **TIMELESS** | TIMELESS.2900 | | TATTCTTCATGATATGACCCAG | | TGCTGTTGACAGTGAGCGATGGGTCATATCATGAAGAATATAGTGAAGCCACAGATGTATATTCTTCATGATATGACCCAGTGCCTACTGCCTCGGA |
| **TOP2B** | TOP2B.232 | | TACATCTTCATCATACACCCAC | | TGCTGTTGACAGTGAGCGATGGGTGTATGATGAAGATGTATAGTGAAGCCACAGATGTATACATCTTCATCATACACCCACTGCCTACTGCCTCGGA |
| **TOP2B** | TOP2B.849 | | TAAAGATCTACATAACTGCGAA | | TGCTGTTGACAGTGAGCGCTCGCAGTTATGTAGATCTTTATAGTGAAGCCACAGATGTATAAAGATCTACATAACTGCGAATGCCTACTGCCTCGGA |
| **TOP2B** | TOP2B.1679 | | TTTTATGTGAGAACCATCTTGA | | TGCTGTTGACAGTGAGCGCCAAGATGGTTCTCACATAAAATAGTGAAGCCACAGATGTATTTTATGTGAGAACCATCTTGATGCCTACTGCCTCGGA |
| **TOP2B** | TOP2B.4436 | | TTTACTTGGAACTTTATCTGTC | | TGCTGTTGACAGTGAGCGAACAGATAAAGTTCCAAGTAAATAGTGAAGCCACAGATGTATTTACTTGGAACTTTATCTGTCTGCCTACTGCCTCGGA |
| **TOP2B** | TOP2B.2861 | | TAGCATAGGTTCTAAAACCTGT | | TGCTGTTGACAGTGAGCGCCAGGTTTTAGAACCTATGCTATAGTGAAGCCACAGATGTATAGCATAGGTTCTAAAACCTGTTGCCTACTGCCTCGGA |
| **TRIP13** | TRIP13.858 | | TTTATCATCAATCAAATCCTGA | | TGCTGTTGACAGTGAGCGCCAGGATTTGATTGATGATAAATAGTGAAGCCACAGATGTATTTATCATCAATCAAATCCTGATGCCTACTGCCTCGGA |
| **TRIP13** | TRIP13.264 | | TAGCTTTCTAACACTCAGGTTT | | TGCTGTTGACAGTGAGCGCAACCTGAGTGTTAGAAAGCTATAGTGAAGCCACAGATGTATAGCTTTCTAACACTCAGGTTTTGCCTACTGCCTCGGA |
| **TRIP13** | TRIP13.839 | | TGAATCTTCTGAAACATCTTGG | | TGCTGTTGACAGTGAGCGACAAGATGTTTCAGAAGATTCATAGTGAAGCCACAGATGTATGAATCTTCTGAAACATCTTGGTGCCTACTGCCTCGGA |
| **TRIP13** | TRIP13.989 | | TTTAATCTGATCAATTTGGGTC | | TGCTGTTGACAGTGAGCGAACCCAAATTGATCAGATTAAATAGTGAAGCCACAGATGTATTTAATCTGATCAATTTGGGTCTGCCTACTGCCTCGGA |
| **TRIP13** | TRIP13.241 | | TTTATGTCTTCTTTCTTTGCAG | | TGCTGTTGACAGTGAGCGATGCAAAGAAAGAAGACATAAATAGTGAAGCCACAGATGTATTTATGTCTTCTTTCTTTGCAGTGCCTACTGCCTCGGA |
| **WBP11** | WBP11.2579 | | TAATTGTACATATTCTTCCCAC | | TGCTGTTGACAGTGAGCGATGGGAAGAATATGTACAATTATAGTGAAGCCACAGATGTATAATTGTACATATTCTTCCCACTGCCTACTGCCTCGGA |
| **WBP11** | WBP11.846 | | TAACATGTCTTCATCTCGCCTA | | TGCTGTTGACAGTGAGCGCAGGCGAGATGAAGACATGTTATAGTGAAGCCACAGATGTATAACATGTCTTCATCTCGCCTATGCCTACTGCCTCGGA |
| **WBP11** | WBP11.236 | | TTCTTTAATTCTCTCTTCCGGG | | TGCTGTTGACAGTGAGCGACCGGAAGAGAGAATTAAAGAATAGTGAAGCCACAGATGTATTCTTTAATTCTCTCTTCCGGGTGCCTACTGCCTCGGA |
| **WBP11** | WBP11.2271 | | TTCAATAACTGATCTATTCTGG | | TGCTGTTGACAGTGAGCGACAGAATAGATCAGTTATTGAATAGTGAAGCCACAGATGTATTCAATAACTGATCTATTCTGGTGCCTACTGCCTCGGA |
| **WBP11** | WBP11.1135 | | TTGAAGAGGAGTCAGTTCCTTC | | TGCTGTTGACAGTGAGCGAAAGGAACTGACTCCTCTTCAATAGTGAAGCCACAGATGTATTGAAGAGGAGTCAGTTCCTTCTGCCTACTGCCTCGGA |
| **WHSC1** | WHSC1.1087 | | TTGAGGGTTGAGATGAAGCTGG | | TGCTGTTGACAGTGAGCGACAGCTTCATCTCAACCCTCAATAGTGAAGCCACAGATGTATTGAGGGTTGAGATGAAGCTGGTGCCTACTGCCTCGGA |
| **WHSC1** | WHSC1.767 | | TTAAGTTTGGTATAGCTGTGAA | | TGCTGTTGACAGTGAGCGCTCACAGCTATACCAAACTTAATAGTGAAGCCACAGATGTATTAAGTTTGGTATAGCTGTGAATGCCTACTGCCTCGGA |
| **WHSC1** | WHSC1.67 | | TTATGCACTTTACAACACTCTG | | TGCTGTTGACAGTGAGCGAAGAGTGTTGTAAAGTGCATAATAGTGAAGCCACAGATGTATTATGCACTTTACAACACTCTGTGCCTACTGCCTCGGA |
| **WHSC1** | WHSC1.777 | | TTTCTGACCTTTAAGTTTGGTA | | TGCTGTTGACAGTGAGCGCACCAAACTTAAAGGTCAGAAATAGTGAAGCCACAGATGTATTTCTGACCTTTAAGTTTGGTATGCCTACTGCCTCGGA |
| **WHSC1** | WHSC1.279 | | TTCTCCATTAAACACCCGGGAA | | TGCTGTTGACAGTGAGCGCTCCCGGGTGTTTAATGGAGAATAGTGAAGCCACAGATGTATTCTCCATTAAACACCCGGGAATGCCTACTGCCTCGGA |
| **XRCC6** | XRCC6.842 | | TTGAGCTTCAGCTTTAACCTGC | | TGCTGTTGACAGTGAGCGACAGGTTAAAGCTGAAGCTCAATAGTGAAGCCACAGATGTATTGAGCTTCAGCTTTAACCTGCTGCCTACTGCCTCGGA |
| **XRCC6** | XRCC6.1682 | | TTTCTCTTGGTAACTTTCCCTT | | TGCTGTTGACAGTGAGCGCAGGGAAAGTTACCAAGAGAAATAGTGAAGCCACAGATGTATTTCTCTTGGTAACTTTCCCTTTGCCTACTGCCTCGGA |
| **XRCC6** | XRCC6.480 | | TGAGTGAGTAGTCAGATCCGTG | | TGCTGTTGACAGTGAGCGAACGGATCTGACTACTCACTCATAGTGAAGCCACAGATGTATGAGTGAGTAGTCAGATCCGTGTGCCTACTGCCTCGGA |
| **XRCC6** | XRCC6.828 | | TTAACCTGCTGAGTGCTCGCTT | | TGCTGTTGACAGTGAGCGCAGCGAGCACTCAGCAGGTTAATAGTGAAGCCACAGATGTATTAACCTGCTGAGTGCTCGCTTTGCCTACTGCCTCGGA |
| **XRCC6** | XRCC6.270 | | TACTGATGTACACACTTTGGAT | | TGCTGTTGACAGTGAGCGCTCCAAAGTGTGTACATCAGTATAGTGAAGCCACAGATGTATACTGATGTACACACTTTGGATTGCCTACTGCCTCGGA |
| **ZNF207** | ZNF207.1823 | | TAAGCTTACAGAACTTGCCTTT | | TGCTGTTGACAGTGAGCGCAAGGCAAGTTCTGTAAGCTTATAGTGAAGCCACAGATGTATAAGCTTACAGAACTTGCCTTTTGCCTACTGCCTCGGA |
| **ZNF207** | ZNF207.1051 | | TTGTACTAGTTGTTGAAGCTGT | | TGCTGTTGACAGTGAGCGCCAGCTTCAACAACTAGTACAATAGTGAAGCCACAGATGTATTGTACTAGTTGTTGAAGCTGTTGCCTACTGCCTCGGA |
| **ZNF207** | ZNF207.318 | | TATTGTTTCTTTATGTACCTGC | | TGCTGTTGACAGTGAGCGACAGGTACATAAAGAAACAATATAGTGAAGCCACAGATGTATATTGTTTCTTTATGTACCTGCTGCCTACTGCCTCGGA |
| **ZNF207** | ZNF207.1042 | | TTGTTGAAGCTGTAGACTGTGT | | TGCTGTTGACAGTGAGCGCCACAGTCTACAGCTTCAACAATAGTGAAGCCACAGATGTATTGTTGAAGCTGTAGACTGTGTTGCCTACTGCCTCGGA |
| **ZNF207** | ZNF207.257 | | TTTCTTGTGACATATATGGCAT | | TGCTGTTGACAGTGAGCGCTGCCATATATGTCACAAGAAATAGTGAAGCCACAGATGTATTTCTTGTGACATATATGGCATTGCCTACTGCCTCGGA |

**Suppl. Table 2**

**Table S2** Cell lines, source, authentication, culture conditions and selection antibiotics

| **cell line** | **authentication** | **medium** | **additions** | **Hygromycin** | **Puromycin** | **G418** | **Blasticidin** |
| --- | --- | --- | --- | --- | --- | --- | --- |
| **CaCo2** (ATCC) | 13 Feb 2018, CLS GmbH | DMEM | 10% FCS, 1% Pen/Strep, 1% Ultraglutamine solution | 250 µg/ml | 5.0 µg/ml | 1 250 µg/ml | --- |
| **HT29** (ATCC) | 11 Nov 2018, CLS GmbH | DMEM | 10% FCS, 1% Pen/Strep, 1% Ultraglutamine solution | --- | 1.0 µg/ml | 1 000 µg/ml | --- |
| **WiDr** (kindly provided by Prof. Reinhold Schäfer) | 29 May 2015, CLS GmbH | DMEM | 10% FCS, 1% Pen/Strep, 1% Ultraglutamine solution | --- | 1.0 µg/ml | 1 000 µg/ml | --- |
| **SW480** (ATCC) | 13 Feb 2018, CLS GmbH | DMEM | 10% FCS, 1% Pen/Strep, 1% Ultraglutamine solution | --- | 5.0 µg/ml | 1 250 µg/ml | --- |
| **HCT8** (ATCC) | 12 Apr 2016, CLS GmbH | DMEM | 10% FCS, 1% Pen/Strep, 1% Ultraglutamine solution, 4g/L glucose | --- | 5.0 µg/ml | 1 250 µg/ml | --- |
| **DLD-1 (KRAS^wt/G13D^)** (Horizon Discovery) | May 2016, freshly purchased | RPMI | 10% FCS, 1% Pen/Strep | --- | 2.5 µg/ml | 1 250 µg/ml | --- |
| **DLD-1 (KRAS^wt/-^)** (Horizon Discovery) | May 2016, freshly purchased | RPMI | 10% FCS, 1% Pen/Strep | --- | 2.5 µg/ml | 1 250 µg/ml | --- |
| **PlatE (HEK293T)** (kindly provided by Prof. Johannes Zuber, IMP) | --- | DMEM | 10% FCS, 1% Pen/Strep, 1% Ultraglutamine solution, 4g/L glucose | --- | 1.0 µg/ml | --- | 10 µg/ml |
| **HCET1T** (ATCC) | Oct 2018, freshly purchased | DMEM/Medium199 4+1 | 4mM Glutamax-1, 20ng/ml EGF, 10µg/ml Insulin, 2µg/ml Apo-Transferrin, 5nM Sodium-Selenite, 1µg/ml Hydrocortisone | --- | --- | --- | --- |

**Suppl. Table 3**

**Table S3** antibodies

| **Antibody target** | **Manufacturer** | **#** | **dilution WB** | **dilution IF** | **dilution FACS** |
| --- | --- | --- | --- | --- | --- |
| 53BP1 | Biomol Bethyl | A300-272A-M |  | 1:100 |  |
| BrdU | BD Bioscience | #347580 |  |  | 1:50 |
| pCHK1 (S345) | CST | 2341 | 1:1 000 |  |  |
| pCHK2(T68) | CST | 2661 | 1:2 000 |  |  |
| Cleaved Caspase 3 (Asp175) Alexa Fluor® 647 Conjugate | CST | 9602 |  |  | 1:50 |
| cleaved PARP | CST | 9541 | 1:1 000 |  |  |
| Total PARP | CST | 9532 | 1:1000 |  |  |
| pERK1/2 | CST | 4370 | 1:2 000 |  |  |
| γH2AX | Millipore | 05-636 |  | 1:100 | 1:250 |
| γH2AX | CST | 9718 |  | 1:200 |  |
| GAPDH | Invitrogen | AM4300 | 1:10 000 |  |  |
| pHH3 (Ser10) | CST | 9701 |  | 1:200 |  |
| pHH3 (Ser10) | CST | 5764 |  |  | 1:50 |
| KRAS | OriGene | TA801672 | 1:2 000 |  |  |
| MCM2 | BD | 610701 | 1:2 000 | 1:50 |  |
| pMCM2 (S40) | Abcam | ab133243 | 1:1 000 |  |  |
| MCM7 | Santa Cruz | sc-9966 | 1:200 |  |  |
| PCNA | Santa Cruz | sc-56 | 1:200 |  |  |
| RPA32 | Abcam | ab2175 | 1:500 | 1:300 |  |
| pRPA32 (Thr21) | Abcam | ab61065 | 1:500 |  |  |
| ß-Tubulin | CST | 2146 | 1:1 000 |  |  |
| Vinculin | CST | 4650 | 1:1 000 |  |  |
| 53BP1 | Biomol Bethyl | A300-272A-M |  | 1:100 |  |
| BrdU | BD Bioscience | #347580 |  |  | 1:50 |
| pCHK1 (S345) | CST | 2341 | 1:1 000 |  |  |
| pCHK2(T68) | CST | 2661 | 1:2 000 |  |  |
